# Long-term restoration of auditory function in a DFNA2 mouse model by adenine base editing

**DOI:** 10.1038/s44321-026-00433-5

**Published:** 2026-05-20

**Authors:** Yuxuan Kong, Yingjie Zhang, Erjie Xie, Xiao Li, Zhuoxi Peng, Jingyuan Zhang, Yu Zhao, Huijun Yuan

**Affiliations:** 1 Department of Otorhinolaryngology-Head & Neck Surgery, West China Hospital, Sichuan University, Chengdu, 610041 China; 2 Institute of Rare Diseases, West China Hospital, Sichuan University, Chengdu, 610000 China

**Keywords:** Genetics, Gene Therapy & Genetic Disease

## Abstract

Hereditary hearing loss, the most prevalent genetic sensory disorder, lacks approved pharmacological therapies and represents a compelling target for gene correction. Pathogenic variants in *KCNQ4* account for ~9.5% of autosomal dominant nonsyndromic cases. Prior gene-editing strategies disrupting mutant alleles have failed to achieve durable auditory rescue. Here we employed a knock-in mouse model harboring the human *KCNQ4* c.961 G > A (p.G321S) mutation to evaluate precise base editing. Dual-AAV delivery of the adenine base editor ABE8e achieved 21.4–28.9% correction in the organ of Corti—the highest efficiency reported for genetic hearing loss. A dose-dependent therapeutic window emerged: higher doses promoted rapid recovery, whereas optimized lower doses minimized long-term toxicity and sustained functional benefit for at least 32 weeks. Treatment reduced auditory brainstem response thresholds by up to 49.09 dB SPL at optimal frequencies, mitigated degeneration of hair cells, spiral ganglion neurons, and auditory nerve fibers, and partially restored outer hair cell electrophysiology. These findings demonstrate the durability of precise mutation correction over allele-disruptive approaches and support clinical translation for *KCNQ4*-associated hearing loss.

## Synopsis

Auditory function was successfully restored in a *Kcnq4* p.G322S mouse model of DFNA2 following the delivery of the adenine base editor ABE8e via dual-AAV vectors.

- A genomic correction efficiency of approximately 21%-29% was achieved in homozygous mice using the ABE8e base editor.
- Auditory function in heterozygous mice was restored and maintained for a sustained period of at least 32 weeks post-treatment.
- Structural preservation of hair cells, spiral ganglion neurons, and nerve fibers was observed and accompanied by improved electrophysiological function of OHCs.
- A dose-dependent efficacy profile was identified, in which accelerated decline was noted at high doses while durable efficacy was obtained with low-dose treatment.

## Abstract (web summary)

Auditory function was successfully restored in a *Kcnq4* p.G322S mouse model of DFNA2 following the delivery of the adenine base editor ABE8e via dual-AAV vectors.

## Graphical abstract

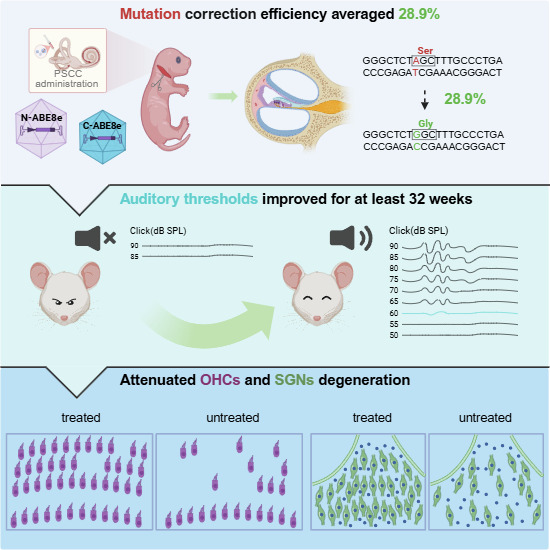

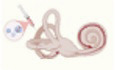

The paper explained **Medical issue**

Gene therapies have emerged as a transformative approach for treating monogenic disorders. Hereditary hearing loss, the most prevalent genetic sensory disorder, currently has no approved pharmacological treatments, making it an ideal target for gene-based interventions. Pathogenic variants in *KCNQ4* underlie roughly 9.5% of autosomal dominant nonsyndromic hearing loss (DFNA2). Although previous attempts have aimed to disrupt mutant *Kcnq4* alleles, long-lasting restoration of auditory function has remained elusive. Here, we explored a mutation-correction strategy to determine whether precise gene editing could achieve more robust and sustained hearing recovery in a DFNA2 mouse model.

**Result**

Using dual-AAV vectors to deliver the ABE8e base editor, we achieved highly efficient correction of *Kcnq4* mutations in mice. This intervention led to substantial and sustained restoration of auditory function for at least 32 weeks, accompanied by preservation of hair cells, spiral ganglion neurons, and nerve fiber structure, as well as enhanced electrophysiological function of outer hair cells. Importantly, we observed a dose-dependent safety profile: mice receiving higher doses exhibited accelerated hearing decline at later stages compared with lower-dose groups. These findings underscore the critical importance of dose optimization for achieving durable efficacy while maintaining safety in inner-ear gene editing.

**Clinical impact**

These results provide compelling proof-of-concept for the use of base editing to treat DFNA2 hereditary hearing loss. They also highlight the crucial role of dose selection in gene therapy, offering key guidance to ensure long-term safety and efficacy, and laying the groundwork for future clinical translation.

## Introduction

Gene therapies offer a transformative approach by precisely correcting specific genetic defects, rendering much of the human genome “druggable” (Kliegman et al, 2024). Rapid advancements in therapeutics for metabolic, immunological, neuromuscular, hematological, and ophthalmological disorders have established gene therapy as a promising strategy to address the needs of over 4,000 rare monogenic diseases, cancers, and other prevalent conditions (Kliegman et al, 2024; Trajanoska et al, 2023). Hereditary hearing loss (HHL), the most prevalent genetic sensory disorder, profoundly impacts early language acquisition and accelerates cognitive decline later in life (Wilson and Tucci, 2021). Genetic mutations account for approximately 60% of congenital hearing loss cases, with over 200 implicated genes, and no pharmacological treatments are currently approved (Jiang et al, 2023; WD et al, 2024). The cochlea is a small, enclosed organ, and localized drug delivery can easily achieve sustained, effective therapeutic concentrations. Additionally, the monogenic nature of HHL makes it an ideal candidate for gene therapy. Recent advances, highlighted by successful clinical trials in *OTOF*-related hearing loss, offer hope for the approval of the first HHL therapeutic (Lv et al, 2024; Qi et al, 2024a; Qi et al, 2025a; Wang et al, 2024). Preclinical studies targeting genes such as *Otof* (Al-Moyed et al, 2019; Cui et al, 2024; Qi et al, 2024b; Tang et al, 2023; Xue et al, 2023; Zhang et al, 2023), *Slc17a8* (Akil et al, 2012), *Tmc1* (Gyorgy et al, 2019; Ratzan et al, 2024; Wu et al, 2021), *Ush1c* (Lentz et al, 2013; Lentz et al, 2020; Pan et al, 2017; Ponnath et al, 2018), *Klhl18* (Gu et al, 2022), and *Mpzl2* (Jiang et al, 2024) have achieved auditory restoration lasting more than 6 months in mice. However, most gene therapy approaches in postnatal murine models demonstrate only transient efficacy, with improvements lasting only weeks(Jiang et al, 2023; Sun et al, 2025; Vona et al, 2025; Zhang et al, 2024). As a result, achieving lasting auditory restoration continues to be a primary goal in gene therapy for HHL.

*KCNQ4* encodes a voltage-gated potassium (K^+^) channel protein that plays a crucial role in K^+^ homeostasis within the cochlear endolymph and perilymph (Kubisch et al, 1999). Pathogenic mutations in *KCNQ4* underlie DFNA2 (OMIM:600101), accounting for ~9.5% of autosomal dominant nonsyndromic hearing loss (NSHL) cases. Most pathogenic variants trigger early-onset progressive hearing loss via dominant-negative effects and impaired surface expression, leading to outer hair cell (OHC) degeneration (Cui et al, 2022; Sloan-Heggen et al, 2016), whereas *KCNQ4* haploinsufficiency results in late-onset high-frequency loss (Kamada et al, 2006). Notably, because *KCNQ4*-related deafness is predominantly driven by dominant-negative mechanisms, universal treatment via gene replacement is theoretically infeasible; in the absence of upstream or downstream universal targets, each pathogenic variant must be individually corrected through precision gene editing.

Knock-in mouse models of *Kcnq4* mutations (p.W276S and p.G229D) have served as critical platforms for therapeutic investigations using CRISPR-based genome editing or antisense oligonucleotides (ASOs). In these models, dual-AAV delivery of SpCas9 with Anc80L65 capsids and single-AAV delivery of SaCas9-KKH via PHP.eB capsids yielded cochlear DNA editing efficiencies of 0.6 and 1.45%, respectively, at 2 weeks post-treatment (Cui et al, 2022; Noh et al, 2022). More recently, engineered virus-like particles (eVLPs) were used to deliver SpCas9 into the inner ear, attaining average editing efficiencies of 7.14% at 1 week and 14.12% at 7 weeks in the *Kcnq4* p.W276S model (Noh et al, 2025a). In parallel, intracochlear ASO administration partially reduced mutant transcript levels in heterozygous *Kcnq4* p.W276S mice (Jang et al, 2025). Collectively, these interventions partially restored OHC Kcnq4 channel function, promoted OHC survival, and improved auditory thresholds for 7–12 weeks post-treatment (Cui et al, 2022; Jang et al, 2025; Noh et al, 2025a; Noh et al, 2022). Although encouraging, the magnitude and durability of hearing recovery remain limited, underscoring the need for more effective and durable therapeutic strategies. Moreover, impaired *Kcnq4* function or expression also contributes to age-related (Jeng et al, 2020), noise-induced (Wang et al, 2021), and ototoxic hearing loss (Leitner et al, 2011). While mutant allele disruption can alleviate dominant-negative effects, it cannot restore normal biallelic expression; incomplete mutant removal or insufficient wild-type expression may therefore limit long-term recovery. Precise mutation correction offers a more powerful strategy to theoretically restore normal biallelic *Kcnq4* expression. Unlike CRISPR nucleases that induce double-strand breaks (DSBs) and rely on error-prone repair or donor templates, CRISPR base editors enable targeted single-nucleotide transitions (e.g., C → T, A → G, C → G, T → G, A → C) without DSBs (Chen et al, 2024; Gaudelli et al, 2017; Komor et al, 2016; Kurt et al, 2021; Ye et al, 2024), thereby minimizing genomic instability and off-target risks (Huang et al, 2021). This high-precision platform holds particular promise for HHL(Cui et al, 2024; Yeh et al, 2020) and other genetic disorders (Musunuru et al, 2021; Suh et al, 2021).

Here, we report ABE8e-mediated correction of the *Kcnq4* c.964 G > A (p.G322S) mutation in a mouse model recapitulating the human *KCNQ4* c.961 G > A (p.G321S) variant. In the cochlear organ of Corti, we observed an average genomic DNA correction efficiency of 21.4–28.9% (up to 39%). This intervention led to a significantly improved auditory threshold for at least 32 weeks, and attenuated degeneration of hair cells (HCs), spiral ganglion neurons (SGNs), afferent and efferent nerve fibers, and partially restored the electrophysiological function of OHCs. These results highlight the potential of precise *Kcnq4* mutation correction for achieving more durable auditory restoration than allele disruption does, establishing a foundational framework for advancing therapies for *KCNQ4*-related hearing loss.

## Results

### Generation of a *Kcnq4* c.964 G>A (p.G322S) mouse model recapitulating the human *KCNQ4* c.961 G>A (p.G321S) variant

Within our deafness cohort (Cheng et al, 2025), we identified seven patients carrying *KCNQ4* mutations linked to hearing impairment, including five pathogenic variants (Table EV1). Four of these variants have been previously reported: *KCNQ4* c.887 G > A (p.G296D), c.853 G > A (p.G285S), c.961 G > A (p.G321S), and c.1251del (p.C418Afs*78). Furthermore, we discovered a novel pathogenic mutation, c.1107 T > A (p.Y369*, classified as PVS1 + PM2 + PP3). These patients presented a spectrum of hearing loss with varying ages of onset (Table EV1).

**Figure EV1:**
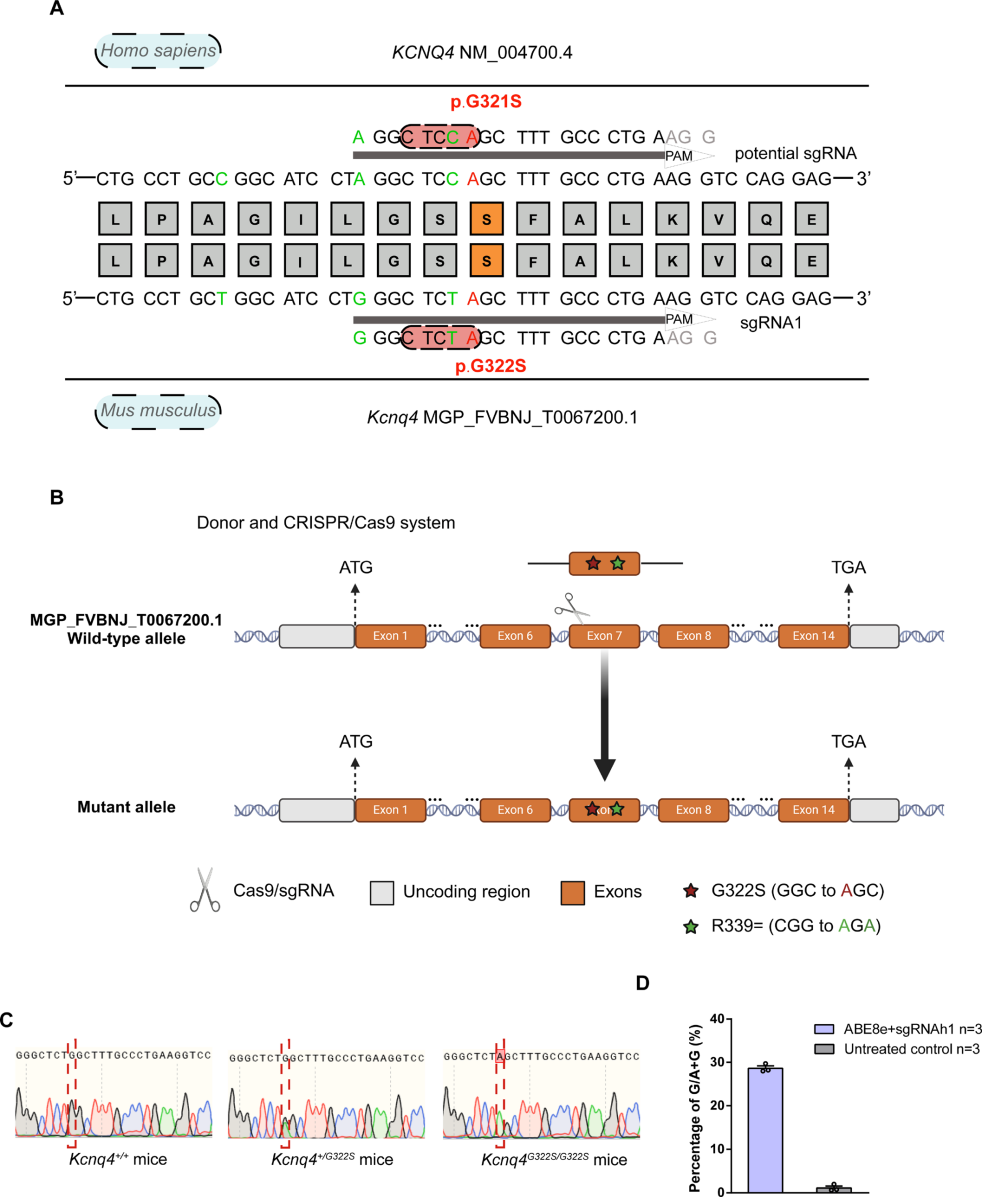
Generation of the *Kcnq4* c.964 G > A (p.G322S) knock-in mouse model and in vitro correction of the human *KCNQ4* c.961 G > A (p.G321S) variant. (**A**) Schematic illustrating the *Kcnq4* c.964 G > A (p.G322S) substitution, recapitulating the pathogenic c.961 G > A (p.G321S) variant identified in DFNA2 patients. Red bases indicate disease-associated mutations; green bases represent synonymous substitutions; gray bases represent PAM sequence (NGG). The light red dashed box marks the optimal editing window of ABE8e. The sgRNA targeting the murine locus and the corresponding sgRNA for the human KCNQ4 allele are shown. (**B**) CRISPR/Cas9-mediated knock-in strategy for generating the *Kcnq4* c.964 G > A (p.G322S) model, incorporating a donor template with a synonymous p.R339= mutation to prevent Cas9 re-cutting. (**C**) Representative Sanger sequencing chromatograms confirming genotypes of *Kcnq4*^*+/+*^, *Kcnq4*^*+/G322S*^, and *Kcnq4*^*G322S/G322S*^ mice. (**D**) HTS-based analysis of editing efficiency in a HEK293T cell line with stable integration of *KCNQ4* p.G321S following transfection with ABE8e and human-specific sgRNA (*n* = 3). Data were presented as mean ± SEM.

**Figure 1:**
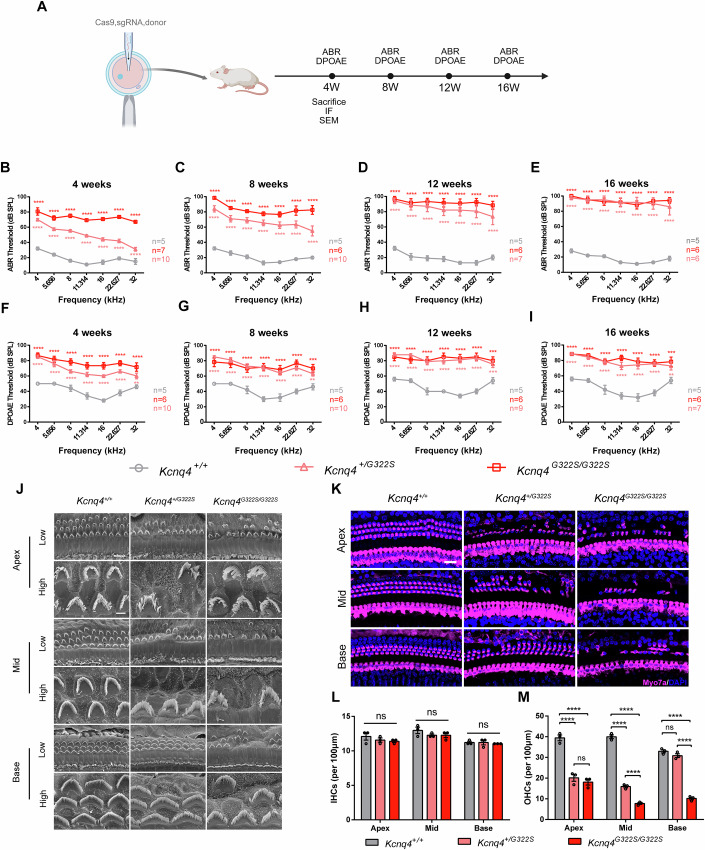
Phenotypic characterization of the *Kcnq4* c.964 G > A (p.G322S) mouse model. (**A**) Schematic overview of auditory assessments. (**B**–**I**) Auditory function was evaluated by ABR (**B**−**E**) and DPOAE (**F**–**I**) in *Kcnq4*^*+/+*^, *Kcnq4*^*+/G322S*^, and *Kcnq4*^*G322S/G322S*^ mice from 4 to 16 weeks of age. Sample sizes (*n*) are indicated for each group. (**J**) Scanning electron microscopy of cochlear structures at 4 weeks of age. Scale bars: 10 μm (low magnification), 2 μm (high magnification). (**K**) Immunofluorescence staining of the whole-mount at 4 weeks of age. Scale bar, 20 μm. (**L**) Quantification of surviving IHCs per 100 μm in the apical, middle, and basal turns of the cochlea of *Kcnq4*^*+/+*^ (*n* = 3), *Kcnq4*^*+/G322S*^ (*n* = 3), and *Kcnq4*^*G322S/G322S*^ (*n* = 3) mice at 4 weeks of age. (**M**) Quantification of surviving OHCs per 100 μm in the apical, middle, and basal turns of the cochlea of *Kcnq4*^*+/+*^ (*n* = 3), *Kcnq4*^*+/G322S*^ (*n* = 3), and *Kcnq4*^*G322S/G322S*^ (*n* = 3) mice at 4 weeks of age. Data were presented as mean ± SEM. Statistical analysis was performed using two-way ANOVA with Bonferroni’s post hoc test. *p < 0.05; **p < 0.01; ***p < 0.001; ****p < 0.0001; ns not significant (Exact *P* values are provided in Appendix Table S2). Source data are available online for this figure.

**Figure EV2:**
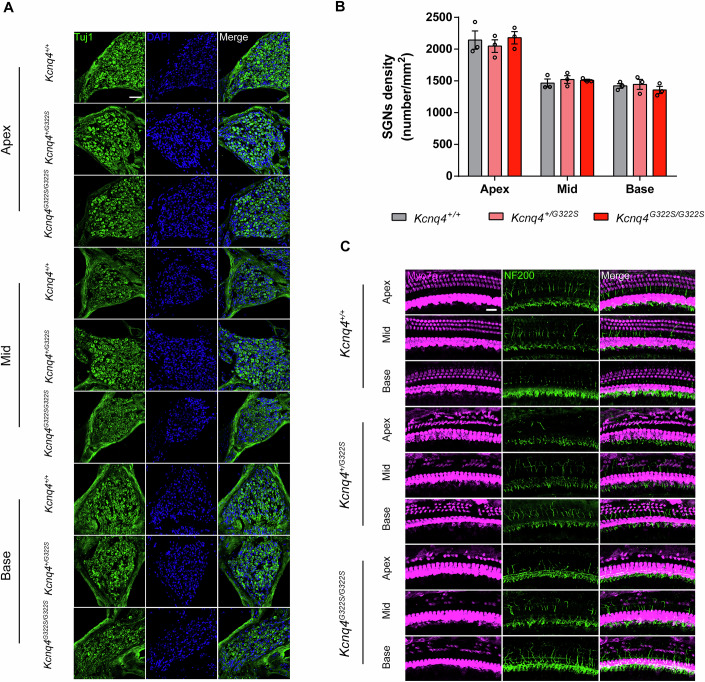
Morphological analysis of SGNs and afferent/efferent nerve fibers by immunofluorescence staining in the *Kcnq4* p.G322S mouse model at 4 weeks of age. (**A**) Representative immunofluorescence images of SGNs in the apical, middle, and basal cochlear turns from *Kcnq4*^*+/+*^, *Kcnq4*^*+/G322S*^, and *Kcnq4*^*G322S/G322S*^ mice. Scale bar, 50 μm. (**B**) Quantification of SGN density in (**A**) from each genotype (*n* = 3). (**C**) Representative immunofluorescence images of afferent/efferent nerve fibers in the apical, middle, and basal cochlear turns from *Kcnq4*^*+/+*^, *Kcnq4*^*+/G322S*^, and *Kcnq4*^*G322S/G322S*^ mice (*n* = 3). Scale bar, 20 μm. Data were presented as mean ± SEM.

After evaluating factors such as age of onset, base editability, bystander editing, reported base editor efficiency, and the potential for small-molecule agonist activation, we generated a mouse model carrying the *Kcnq4* c.964 G > A (p.G322S) mutation, which mirrors the human *KCNQ4* c.961 G > A (p.G321S) variant identified here and has been previously reported to be associated with early-onset hearing loss (Fig. EV1A–C; Table EV1) (Coucke et al, 1999). Auditory brainstem response (ABR) and distortion product otoacoustic emission (DPOAE) testing revealed significant hearing impairment in *Kcnq4*^*+/G322S*^ mice from 4 weeks of age, with pronounced deficits in low- and mid-frequency ranges, progressing to profound hearing loss at all frequency ranges by 16 weeks. Homozygous *Kcnq4*^*G322S/G322S*^ mice presented more severe deficits across all frequencies (Fig. 1A–I). Immunofluorescence analysis demonstrated that hair cell integrity was preserved at P8. By P14, however, the mutant cochleae exhibited the onset of OHC loss (Appendix Fig. S2A,B), which progressed to a pronounced depletion by 4 weeks of age. Quantitative analysis revealed that, in heterozygous mice, OHC numbers were reduced by 48.9 and 60.0% in the apical and middle turns, respectively, whereas the basal turn remained largely unaffected. In homozygous *Kcnq4*^*G322S/G322S*^ cochleae at 4 weeks, OHC loss was more severe, with reductions of 54.2, 80.7, and 69.1% observed in the apical, middle, and basal turns, respectively (Fig. 1K,M). Concomitant with OHC loss, efferent innervation in the apical OHC region was markedly diminished (Fig. EV2C), indicating disrupted cochlear neural circuitry. This neuronal fiber loss may reflect secondary degeneration following OHC death or impaired trophic interactions, and likely contributes to the functional deficits observed in mutant animals. In contrast, the IHC counts remained unaltered at this stage (Fig. 1K,L). Scanning electron microscopy confirmed the preservation of stereociliary architecture and polarity in surviving hair cells at 4 weeks (Fig. 1J). Similarly, no morphological or density changes were observed in the SGNs of mutant mice, suggesting intact SGN survival at 4 weeks (Fig. EV2A,B). These findings establish *Kcnq4*^*+/G322S*^ mice as a robust model for progressive hearing loss and provide a valuable platform for evaluating gene therapy efficacy.

### In vitro screening ABE systems for *Kcnq4* c.964 G > A (p.G322S) mutation correction

**Figure 2:**
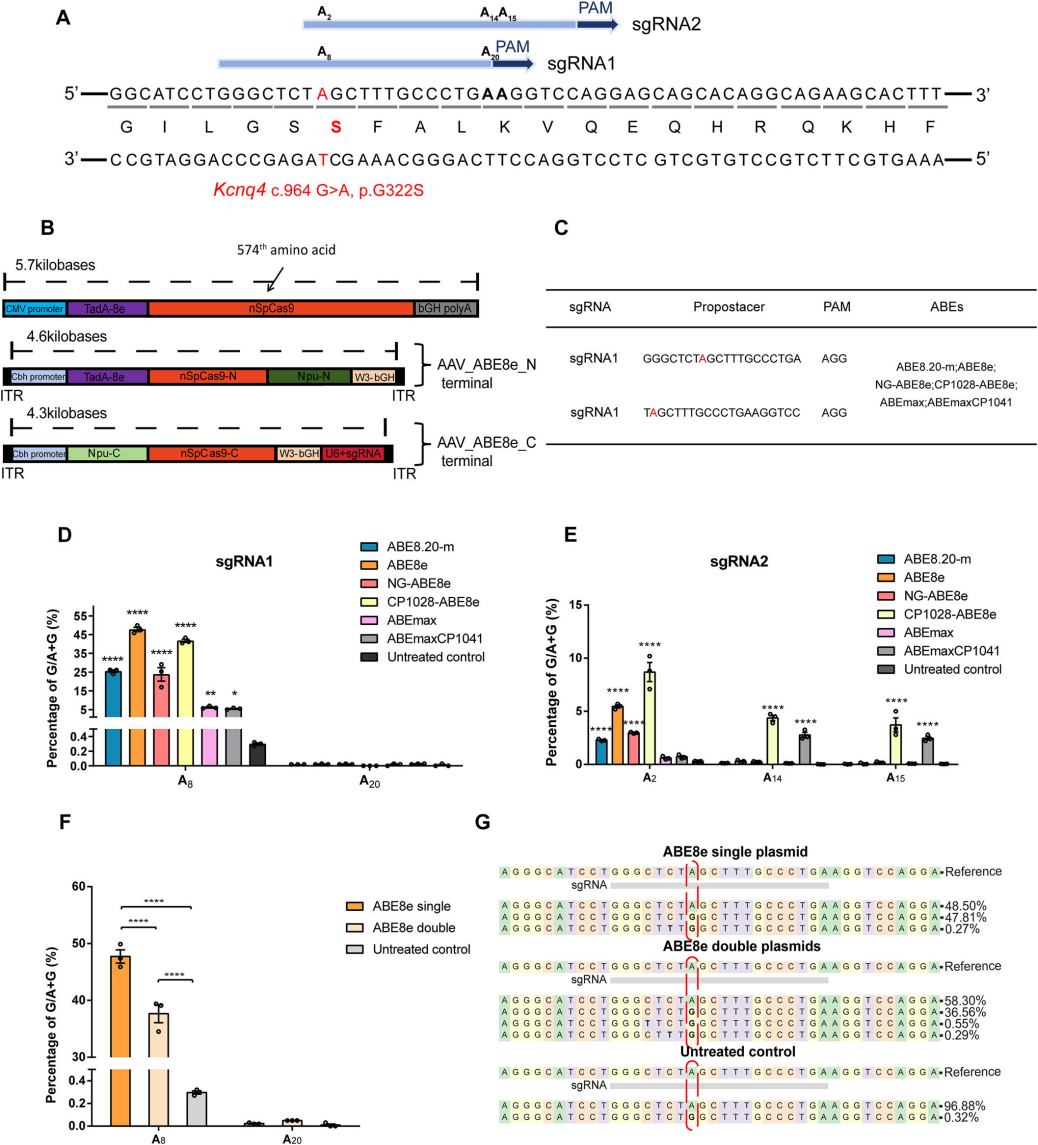
Optimization and screening of ABE systems for correcting the *Kcnq4* c.964 G > A (p.G322S) mutation. (**A**) Schematic of the target sequence at the mouse *Kcnq4* c.964 G > A (p.G322S) locus. (**B**) Schematic of the full-length ABE8e construct and the dual AAV-compatible, intein-mediated split-ABE system. (**C**) Sequences of sgRNA1 and sgRNA2 alongside the corresponding ABE variants tested in this study. (**D**,** E**) Quantification of the post-editing G/(A + G) ratio for six ABE variants using HTS (*n* = 3). Statistical comparisons were performed relative to untreated cell line controls. (**F**) Comparative evaluation of the post-editing G/(A + G) ratio with sgRNA1 delivered via full-length ABE8e or dual AAV-compatible plasmids (*n* = 3). Statistical comparisons were made across all groups. (**G**) Representative CRISPResso2 output from panel (**F**), with target adenines highlighted by red dashed circles. Data were presented as mean ± SEM. Statistical analysis was performed using two-way ANOVA with Bonferroni’s post hoc test. *p < 0.05; **p < 0.01; ****p < 0.0001 (Exact *P* values are provided in Appendix Table S1). Source data are available online for this figure.

Target sequence analysis (Fig. 2A) guided the selection of six ABEs (NGG or NG PAM) and two sgRNAs for evaluation (Fig. 2C). These were co-transfected into a lentiviral *Kcnq4*^G322S^ stable HEK293T cell line (HEK293T*-Kcnq4*^G322S^) to assess editing efficiency (Appendix Fig. S1). High-throughput sequencing (HTS) revealed superior activity of sgRNA1 over sgRNA2, resulting in efficient adenine editing without bystander effects. The sgRNA1 + ABE8e combination exhibited the highest editing efficiency, reaching 47.4 ± 1.2%, significantly surpassing that of the sgRNA2 + CP1028-ABE8e pair (8.4 ± 0.9%) by fivefold (Fig. 2D,E). Consequently, sgRNA1 + ABE8e was selected for further studies. To overcome the ~4.7 kb payload limitation of AAV vectors, an intein-mediated split approach was employed to divide ABE8e into N- and C-terminal components (Fig. 2B) (Levy et al, 2020). Validation experiments demonstrated a ~21% reduction in editing efficiency for split ABE8e (37.4 ± 1.6%) compared with the full-length enzyme (Fig. 2F,G). Collectively, these findings establish ABE8e as a potent tool for correcting the *Kcnq4*^G322S^ mutation in vitro, with the split variant prioritized for subsequent in vivo applications.

### In vivo editing efficiency and off-target profiling of the AAV_ABE8e_N+C_322 system

**Figure EV3:**
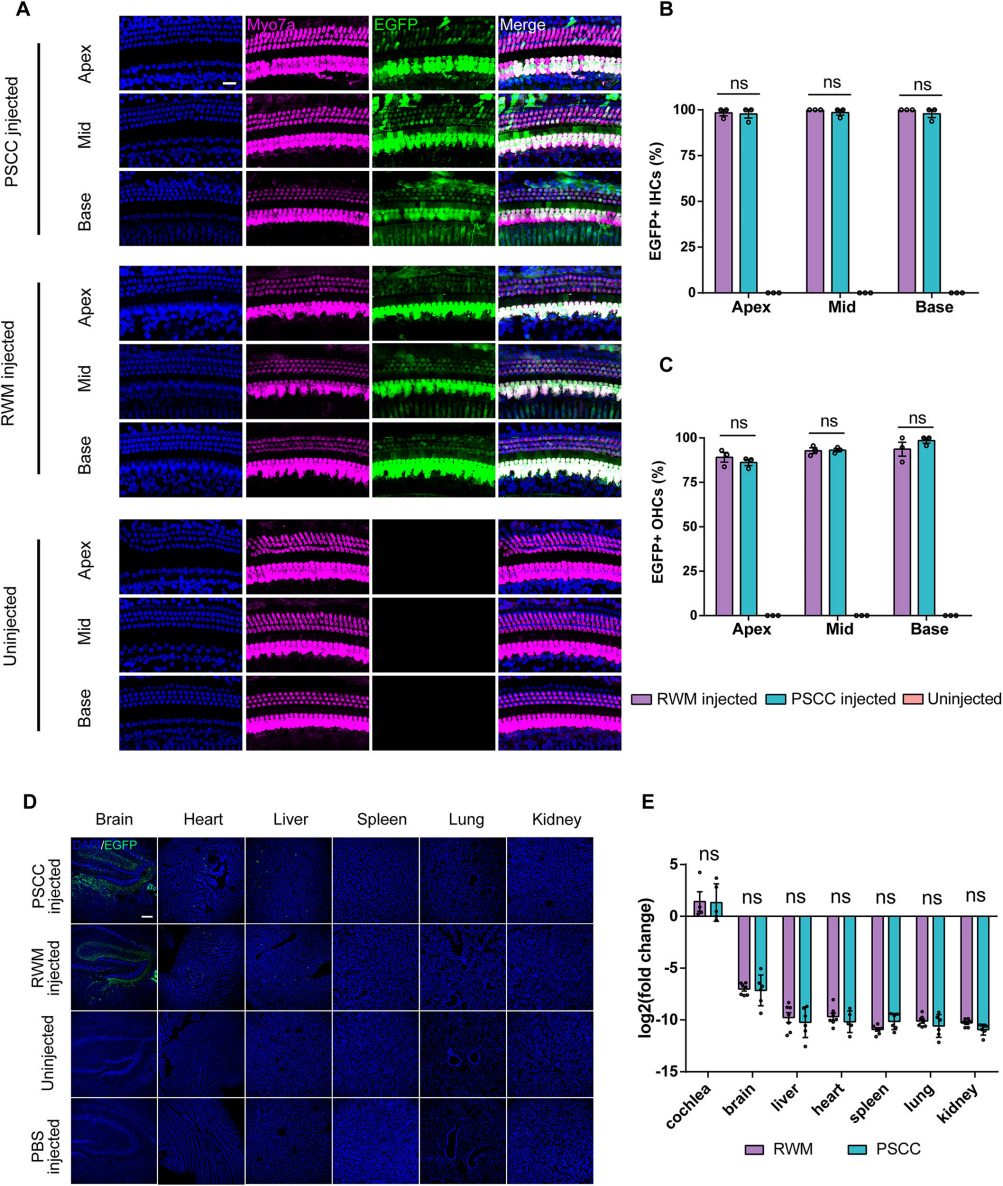
Assessment of cochlear and major organ transduction following AAV-ie delivery via PSCC or RWM injection. (**A**) Representative immunofluorescence images of the whole-mount from *Kcnq4*^*+/+*^ mice three weeks after AAV-ie-CMV-EGFP administration via PSCC or RWM (1 × 10^10^ gc/mouse). (**B**,** C**) Transduction efficiency was quantified as the percentage of EGFP⁺ IHCs (**B**) and OHCs (**C**) across the apical, middle, and basal cochlear turns (*n* = 3). (**D**) Representative immunofluorescence images of major organs from *Kcnq4*^*+/+*^ mice three weeks after AAV-ie-CMV-EGFP administration via PSCC or RWM injection (1 × 10^10^ gc/mouse), along with PBS-injected and uninjected controls. (**E**) RT-qPCR quantification of EGFP expression in major organs (**D**), normalized to cochlear levels (PSCC, cochlea, heart *n* = 5, others *n* = 6; RWM, cochlea *n* = 4, others *n* = 7). Data were presented as mean ± SEM. Statistical analysis was performed using two-way ANOVA with Bonferroni’s post hoc test. ns not significant.

The AAV-ie vector exhibits robust transduction across cochlear cell types, including HCs, supporting cells, and SGNs (Tan et al, 2019). In P2–P3 wild-type (WT) mice injected with AAV-ie-CMV-EGFP (1 × 10^10^ genome copies), over 98% of IHCs were transduced within three weeks, regardless of whether the virus was delivered via the posterior semicircular canal (PSCC) or the round window membrane (RWM). In the PSCC group, OHC transduction efficiency reached 86.2 ± 1.8, 93.2 ± 0.9, and 98.5 ± 1.5% in the apical, middle, and basal turns, respectively. Comparable efficiencies were observed with RWM injection, achieving 89.1 ± 2.8, 92.8 ± 1.6, and 93.7 ± 3.9% transduction in the respective cochlear turns (Fig. EV3A–C). Furthermore, both administration routes exhibited similar systemic biodistribution patterns (Fig. EV3D,E). As no statistically significant difference was detected between the two delivery routes, PSCC injection was used in subsequent experiments.

**Figure 3:**
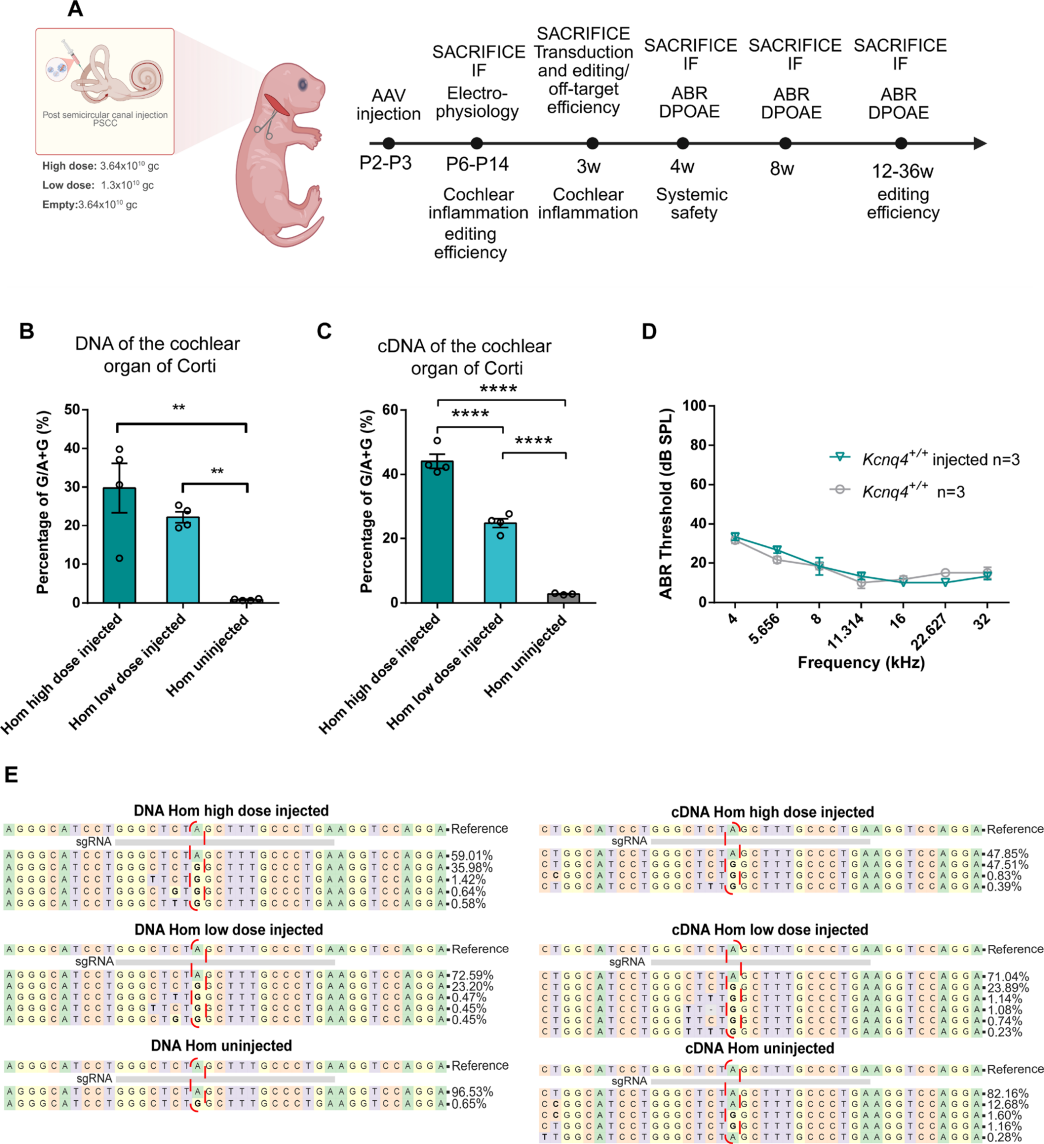
Schematic of the in vivo treatment protocol and assessment of editing efficacy. (**A**) Schematic of the in vivo treatment strategy for *Kcnq4* c.964 G > A (p.G322S) mice. (**B**) Post-editing G/(A + G) ratio in genomic DNA from the cochlear organ of Corti of *Kcnq4*^*G322S/G322S*^ mice, assessed by HTS three weeks post- AAV_ABE8e_N + C_322 injection (high dose: 3.64 × 10^10^ gc/mouse; low dose: 1.3 × 10^10^ gc/mouse, *n* = 4). (**C**) Post-editing G/(A + G) ratio in cDNA from the cochlear organ of Corti of *Kcnq4*^*G322S/G322S*^ mice, assessed by HTS 3 weeks post- AAV_ABE8e_N + C_322 injection (injected *n* = 4; uninjected *n* = 3). (**D**) Auditory safety evaluation in *Kcnq4*^*+/+*^ mice four weeks after AAV_ABE8e_N + C_322 administration (3.64 × 10^10^ gc/mouse, *n* = 3). (**E**) Representative CRISPResso2 output corresponding to panels (**B**, **C**), with the edited adenine highlighted by red dashed circles. Data were presented as mean ± SEM. Statistical analysis was performed using one-way ANOVA with Bonferroni’s post hoc test. **p < 0.01; ****p < 0.0001 (Exact *P* values are provided in Appendix Table S1). Source data are available online for this figure.

**Figure EV4:**
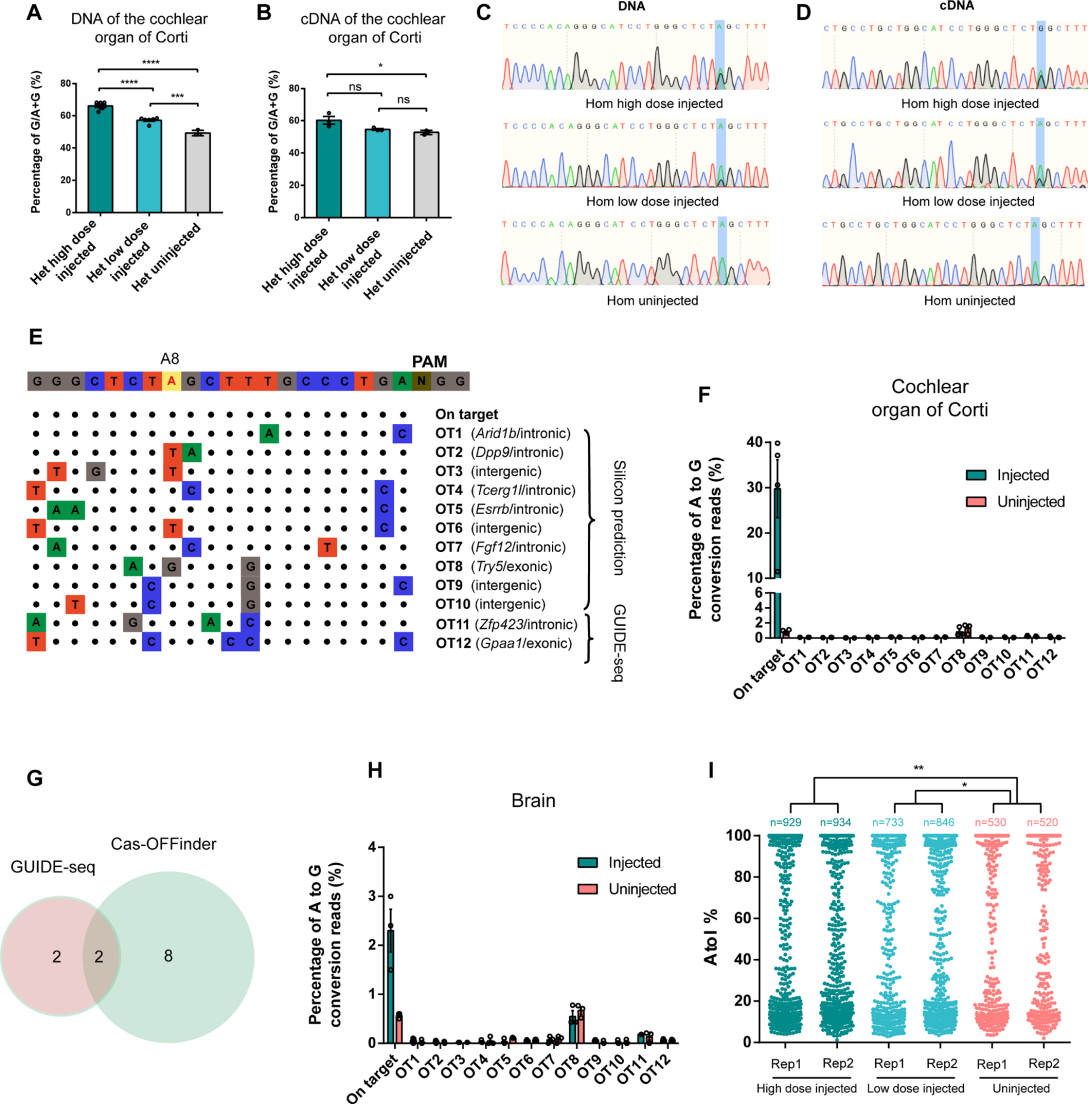
Quantitative assessment of on-target editing efficiency and off-target effects. (**A**) Post-editing G/(A + G) ratio in genomic DNA from the cochlear organ of Corti of *Kcnq4*^*+/G322S*^ mice, assessed by HTS three weeks post-AAV_ABE8e_N + C_322 administration (high dose: 3.64 × 10^10^ gc/mouse, *n* = 6; low dose: 1.3 × 10^10^ gc/mouse, *n* = 6; uninjected *n* = 3). (**B**) Post-editing G/(A + G) ratio in cDNA from the cochlear organ of Corti of *Kcnq4*^*+/G322S*^ mice, assessed by HTS three weeks post-AAV_ABE8e_N + C_322 administration (*n* = 3). (**C**, **D**) Representative Sanger sequencing chromatograms showing A-to-G conversion at the target site in genomic DNA (**C**) and cDNA (**D**) from the cochlear organ of Corti of *Kcnq4*^*G322S/G322S*^ mice three weeks after AAV_ABE8e_N + C_322 treatment. (**E**) Predicted top ten off-target sites (≤3 mismatches, NGG PAM) identified by Cas-OFFinder and two sites detected by GUIDE-seq. (**F**) Off-target A-to-G editing efficiencies assessed by HTS in the cochlear organ of Corti of AAV_ABE8e_N + C_322 treated (3.64 × 10^10^ gc/mouse) *Kcnq4*^*G322S/G322S*^ mice, 3 weeks post-injection (*n* = 3). (**G**) Venn diagram of the top ten Cas-OFFinder–predicted off-target sites and four GUIDE-seq–detected sites. (**H**) Off-target A-to-G editing efficiencies assessed by HTS in the brain of AAV_ABE8e_N + C_322-treated (3.64 × 10^10^ gc/mouse) *Kcnq4*^*G322S/G322S*^ mice, 3 weeks post-injection (*n* = 3). (**I**) Transcriptome-wide A-to-I RNA editing in the cochlea evaluated by RNA-seq in *Kcnq4*^*G322S/G322S*^ mice treated with AAV_ABE8e_N + C_322 (high-dose: 3.64 × 10^10^ gc/mouse; low-dose: 1.3 × 10^10^ gc/mouse; *n* = 2). Data were presented as mean ± SEM. Statistical analysis was performed using one-way or two-way ANOVA with Bonferroni’s post hoc test. *p < 0.05; **p < 0.01; ***p < 0.001; ****p < 0.0001; ns not significant (Exact *P* values are provided in Appendix Table S1).

After confirming that dual-AAV injection (3.64 × 10^10^ gc/mouse) does not induce acute hearing loss in WT mice (Fig. 3D), the split ABE8e components (AAV_ABE8e_N + C_322) were delivered using AAV-ie to *Kcnq4*^G322S/G322S^ mice (Fig. 3A). Three weeks post-delivery, the high-dose group (3.64 × 10^10^ gc/mouse) and low-dose group (1.30 × 10^10^ gc/mouse) achieved average DNA correction efficiencies of 28.9 ± 6.4% (up to 39%) and 21.4 ± 1.5% (up to 24.5%), respectively, in the cochlear organ of Corti, as determined by HTS (Figs. 3B,E and EV4C). These efficiencies substantially exceed previously reported inner ear editing rates within 3 weeks after treatment (0.6–7.14%) (Cui et al, 2022; Cui et al, 2024; Gao et al, 2018; Gyorgy et al, 2019; Hu et al, 2025; Noh et al, 2025a; Noh et al, 2022; Wu et al, 2021; Xue et al, 2022a; Yeh et al, 2020; Zheng et al, 2022). The cDNA correction efficiency reached 41.3 ± 2.2% (up to 47.7%) in the high-dose group and 22.1 ± 1.4% (up to 24.9%) in the low-dose group (Figs. 3C,E and EV4D), corresponding to the correction of ~22–41% of *Kcnq4* mRNA transcripts in cochlear cells endogenously expressing the gene. We further evaluated the editing efficiency of AAV_ABE8e_N + C_322 in *Kcnq4*^+/G322S^ mice. In this model, DNA correction efficiencies ranged from 7.9 ± 1.18% to 16.7 ± 1.38% in the low- and high-dose groups, while cDNA correction efficiencies ranged from 1.9 ± 0.9 to 7.5 ± 2.5%, calculated as the percentage of G/(A + G) at the target site above the control’s baseline (Fig. EV4A,B). Importantly, no bystander A → G conversions were detected at either the genomic or transcriptomic level. To further resolve spatial patterns of editing, we assessed turn-specific DNA correction in 24-week-old *Kcnq4*^*+/G322S*^ cochleae. This analysis revealed a clear dose-dependent effect accompanied by a pronounced tonotopic gradient: in both the high- and low-dose cohorts, the basal turn consistently displayed the highest editing efficiency (27.0 ± 1.9 vs. 18.1 ± 0.7%), followed sequentially by the middle (19.9 ± 1.9 vs. 13.7 ± 3.0%) and apical turns (19.1 ± 0.9 vs. 10.8 ± 1.7%). The aggregate editing efficiency across the organ of Corti reached 22.2 ± 0.3% in the high-dose group and 16.5 ± 2.1% in the low-dose group (Appendix Fig. S5A), representing a progressive increase relative to measurements obtained at three weeks post-injection.

In vivo off-target profiling of the top ten Cas-OFFinder–predicted sites (≤3 mismatches) (Bae et al, 2014) together with four GUIDE-seq–identified loci (Malinin et al, 2021) revealed no detectable DNA off-target editing in the cochlear organ of Corti (Fig. EV4E–G). To assess systemic exposure following cochlear delivery, immunofluorescence and RT-qPCR analyses were performed after injection of AAV-ie–CMV-EGFP. Robust EGFP expression was detected in the hippocampus, with lower-level expression observed in the heart and liver (Fig. EV3D,E; Table EV2). Consistent with these findings, RT-qPCR quantification of ABE8e transcripts and corresponding target-site editing analyses across major organs—including the gonads—demonstrated detectable editor expression outside the cochlea, with the liver exhibiting the highest editing efficiency (~15%) (Appendix Fig. S5B,C). This peripheral activity is likely attributable to the immaturity of the blood–labyrinth barrier in neonatal mice (Xu et al, 2024), permitting transient vector leakage into the systemic circulation and preferential hepatic uptake; however, rapid hepatocyte turnover and immune-mediated clearance may contribute to the subsequent decline in editor expression at later time points (Kumar et al, 2024). Importantly, we did not observe overt hepatotoxicity (Appendix Fig. S7C). Given the potential safety implications of sustained editor expression in the central nervous system, we further interrogated DNA off-target activity at all twelve candidate sites in whole-brain tissue and again detected no significant off-target editing (Fig. EV4H). In addition, unbiased high-depth whole-genome sequencing (WGS; 100× coverage) of cochleae from high-dose–treated mice revealed no significant increase in either the frequency of A/T-to-G/C substitutions or the mutational spectrum compared with uninjected controls (Appendix Fig. S6). At the transcriptomic level, RNA-seq analysis identified an increase in A-to-I RNA editing events in the cochlea following AAV_ABE8e_N + C_322 administration (Fig. EV4I). Sequence-context analysis demonstrated a dose-dependent enrichment of these edits at endogenous ADAR-preferred motifs (Picardi et al, 2017). Notably, the majority of treatment-associated A-to-I events were intronic and functionally annotated as modifier variants (Appendix Fig. S5D–F), suggesting limited biological impact. Collectively, these data support a favorable genome- and transcriptome-wide safety profile of AAV-ie–mediated ABE8e delivery, while demonstrating efficient and precise in vivo correction of the pathogenic *Kcnq4* p.G322S allele. These findings establish a robust preclinical foundation for the development of gene-editing–based therapeutic strategies for hearing restoration.

### Restoration of hearing in *Kcnq4*^*+/G322S*^ mice by AAV_ABE8e_N+C_322

**Figure 4:**
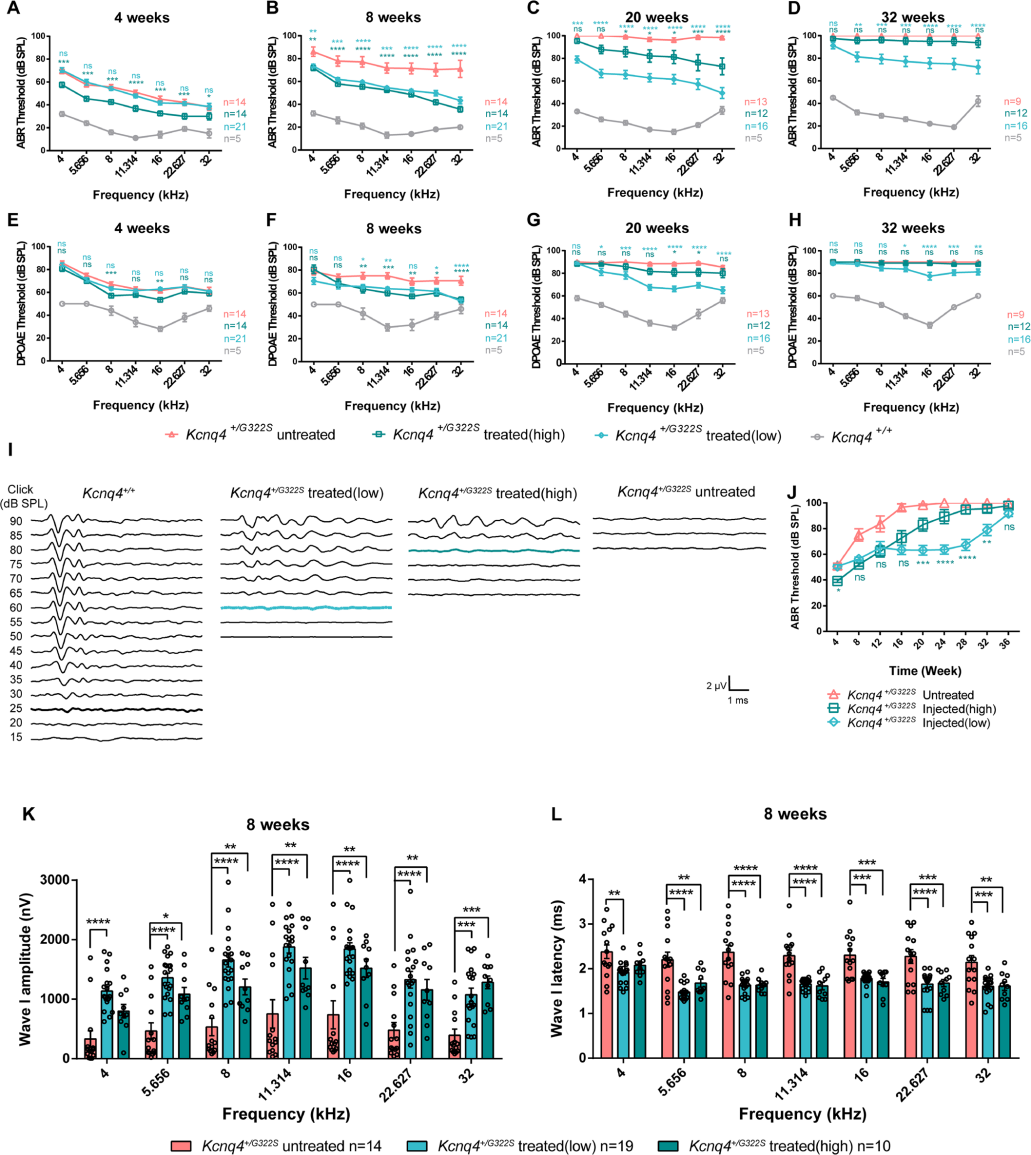
Restoration of auditory function in *Kcnq4*^*+/G322S*^ mice following mutation correction. (**A**–**H**) Auditory function was assessed at 4, 8, 20, and 32 weeks post-treatment with AAV_ABE8e_N + C_322 in high-dose (3.64 × 10^10^ gc/mouse), low-dose (1.3 × 10^10^ gc/mouse), and untreated *Kcnq4*^*+/G322S*^ mice, as well as *Kcnq4*^*+/+*^ controls. Panels (**A**–**D**) show ABR thresholds and panels (**E**–**H**) display DPOAE thresholds. Sample sizes (*n*) are indicated beside each graph. Statistical comparisons were performed between the low-dose treated and untreated group (light teal) and between the high-dose treated and untreated group (dark teal). (**I**) Representative click-evoked ABR waveforms at 20 weeks for each group. Black, light teal, and dark teal lines represent the thresholds of *Kcnq4*^*+/+*^ control, low-dose, and high-dose treated *Kcnq4*^*+/G322S*^ mice, respectively. (**J**) Longitudinal ABR threshold analysis across treatment groups (high-dose, 4–8 weeks *n* = 14, 12–16 weeks *n* = 13, 20–36 weeks *n* = 12; low-dose, 4–8 weeks *n* = 21, 12 weeks *n* = 20, 16 weeks *n* = 18, 20–36 weeks *n* = 16; untreated, 4–8 weeks *n* = 14, 12–20 weeks *n* = 13, 24 weeks *n* = 10, 28–36 weeks *n* = 9). Statistical comparisons highlight differences between high- and low-dose groups. (**K**) Analysis of ABR wave I amplitude at 90 dB SPL in high-dose (*n* = 10), low-dose (*n* = 19), and untreated (*n* = 14) *Kcnq4*^*+/G322S*^ mice at 8 weeks. (**L**) Analysis of ABR wave I latency at 90 dB SPL at 8 weeks in the same cohorts. Data are presented as mean ± SEM. Statistical analysis was performed using mixed-effects model (**J**) or two-way ANOVA (**A**–**H** and **K**, ** L**) with Bonferroni’s post hoc test. *p < 0.05; **p < 0.01; ***p < 0.001; ****p < 0.0001; ns not significant (Exact *P* values are provided in Appendix Table S1). Source data are available online for this figure.

**Figure EV5:**
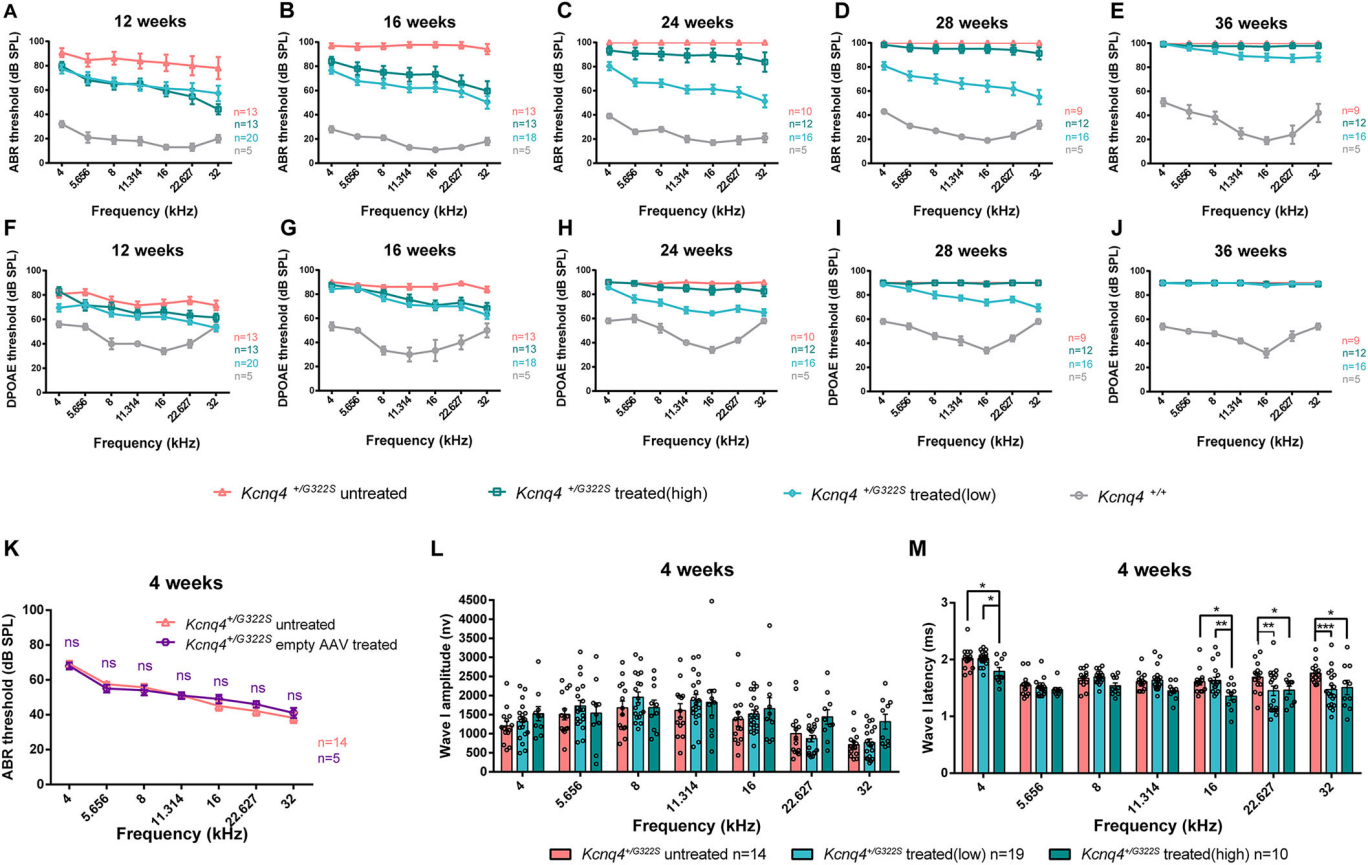
Extended auditory evaluations following AAV_ABE8e_N + C_322 treatment. (**A**–**J**) ABR and DPOAE measurements were performed at 12, 16, 24, 28, and 36 weeks post-treatment of AAV_ABE8e_N + C_322 in high-dose (3.64 × 10^10^ gc/mouse), low-dose (1.3 × 10^10^ gc/mouse), or in untreated *Kcnq4*^*+/G322S*^ mice, as well as *Kcnq4*^*+/+*^ controls. ABR thresholds are shown in (**A**–**E**); DPOAE thresholds in (**F**–**J**). Sample sizes (*n*) are indicated alongside each graph. (**K**) ABR thresholds in *Kcnq4*^*+/G322S*^ mice treated with AAV_ABE8e_N + AAV_ABE8e_C_empty (3.64 × 10^10^ gc/mouse, *n* = 5) compared to untreated *Kcnq4*^*+/G322S*^ controls (*n* = 14) at 4 weeks post-treatment. (**L**, **M**) Quantification of ABR wave I amplitude (L) and latency (**M**) at 90 dB SPL in high-dose (*n* = 10), low-dose (*n* = 19), and untreated (*n* = 14) *Kcnq4*^*+/G322S*^ mice at 4 weeks post-injection of AAV_ABE8e_N + C_322. Data were presented as mean ± SEM. Statistical analysis was performed using two-way ANOVA with Bonferroni’s post hoc test. *p < 0.05; **p < 0.01; ***p < 0.001; ns not significant (Exact *P* values are provided in Appendix Table S1).

Neonatal *Kcnq4*^*+/G322S*^ mice (P2–P3) were then treated with high- or low-dose AAV_ABE8e_N + C_322 or an empty AAV (AAV_ABE8e_N + C_nosgRNA, 3.64 × 10^10^ gc/mouse) as controls (Fig. 3A). The ABR thresholds were recorded from 4–32 kHz. High-dose treatment led to a significant improvement in hearing thresholds, with a shift of 8.2–14.3 decibels (dB) sound pressure level (SPL) at 4 weeks, whereas the low-dose and empty control groups showed no significant changes compared with untreated *Kcnq4*^*+/G322S*^ mice (Figs. 4A and EV5K). By 8 weeks, hearing recovery persisted, with 14.3–35.4 dB SPL shifts in the high-dose group and 12.5–27.7 dB SPL shifts in the low-dose group (Fig. 4B). Long-term auditory preservation was maintained for up to 32 weeks in the low-dose group (Fig. 4D). This sustained functional benefit was accompanied by morphological preservation, as only low-dose–treated mice retained a subset of inner hair cells and their associated afferent innervation at this late stage (Appendix Fig. S4). The maximal auditory threshold improvement peaked at 32 kHz, with a reduction of 49.09 dB SPL (thresholds >90 dB SPL were capped at 100 dB SPL for analysis) by 20 weeks post-treatment (Fig. 4C,I). The pronounced high-frequency rescue is consistent with the preferential transduction and higher on-target editing efficiencies observed in the basal turn of the cochlea (Fig. EV3A,B; Appendix Fig. S5A). Notably, the high-dose group exhibited accelerated auditory threshold deterioration beginning at 16 weeks (Figs. 4C,D and EV5B–E), with an average decline rate of 1.83 dB SPL/week across frequencies, compared with 1.29 dB SPL/week in the low-dose group (Fig. 4J). By 28 weeks, thresholds in the high-dose group surpassed 90 dB SPL across all frequencies (Fig. EV5D). DPOAE corroborated these findings. At 4 weeks, high-dose-treated mice presented a significant threshold shift of 10 dB SPL at 8 kHz and 7.9 dB SPL at 16 kHz (Fig. 4E). By 8 weeks, the high-dose group presented 10.7–16.4 dB SPL shifts across 8–32 kHz, whereas the low-dose group outperformed the high-dose group by 12 weeks (Fig. 4F and EV5F). Notably, DPOAEs in the low-dose group remained detectable for up to 32 weeks, 12 weeks beyond those in the high-dose group (Figs. 4G,H and EV5G–J).

We quantified click ABR wave I amplitudes and latencies at 90 dB SPL to further characterize hearing recovery. At 4 weeks, no significant amplitude improvements were observed in either group (Fig. EV5L). However, partial frequency-specific latency reductions were observed in the treatment groups (Fig. EV5M). At 8 weeks, significant increases in amplitude were observed in both the low-dose (679.5–1124 nV) and high-dose groups (617.9–888.6 nV) across nearly all the tested frequencies, reflecting a significant recovery in the number of neurons firing (Fig. 4K). Latency reductions were also notable in both groups, with the low-dose group showing a range of 0.46–0.73 ms and the high-dose group 0.52–0.74 ms, indicating restored signal transmission velocity (Fig. 4L). In conclusion, our systematic evaluation highlights the efficacy of AAV_ABE8e_N + C_322 in restoring hearing in *Kcnq4*^*+/G322S*^ mice, with lower viral titers yielding superior long-term outcomes. These results underscore the importance of balancing therapeutic efficacy with cellular tolerance to optimize gene-editing interventions.

### Rescue of HCs, SGNs, and cochlear nerve fibers in *Kcnq4*^*+/G322S*^ mice by AAV_ABE8e_N+C_322

**Figure 5:**
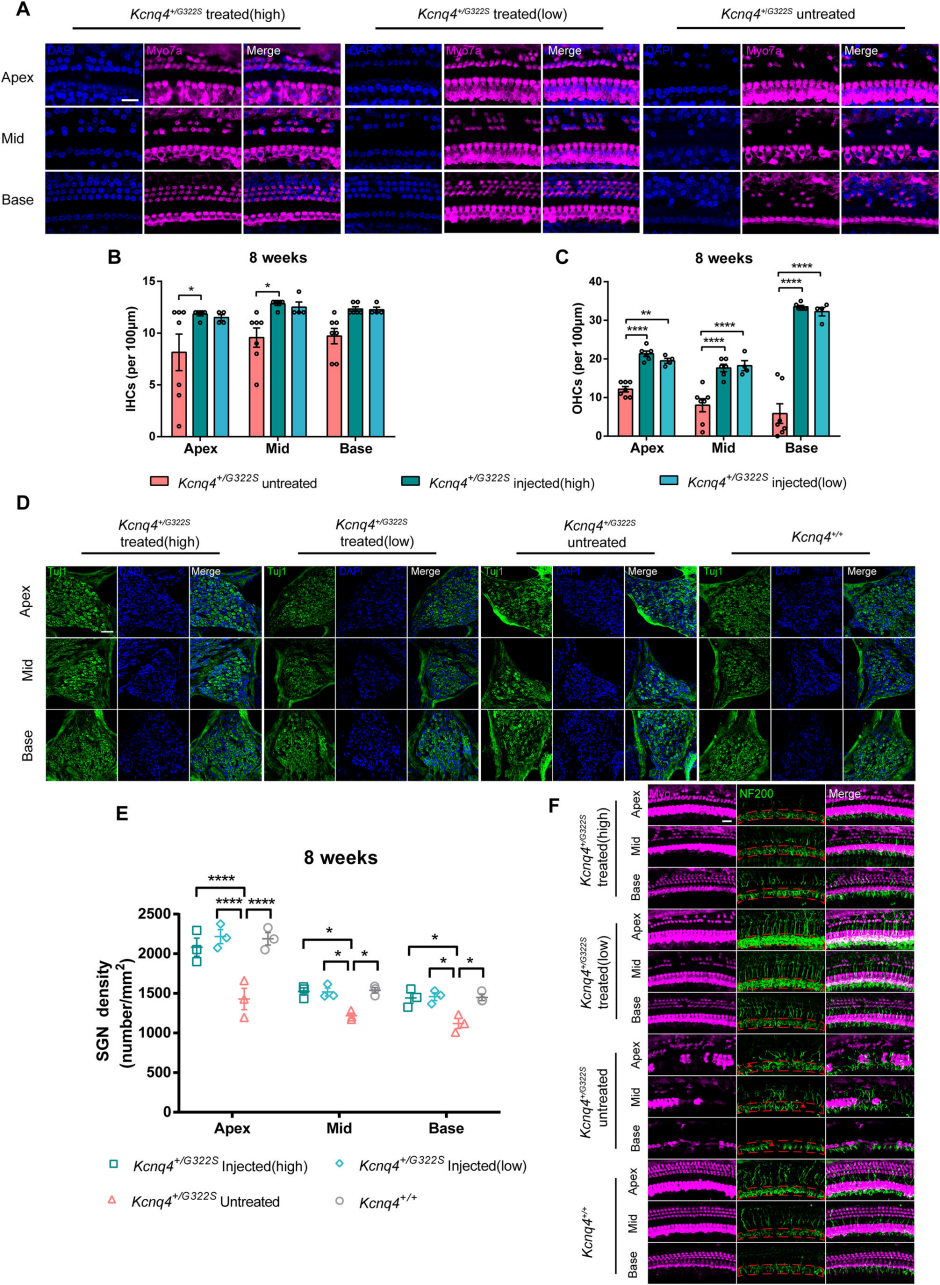
Rescue of HCs, SGNs, and afferent/efferent nerve fibers in 8-week-old *Kcnq4*^*+/G322S*^ mice by AAV_ABE8e_N + C_322. (**A**) Representative immunofluorescence images of HCs in the apical, middle, and basal turns of the cochleae from treated (high-dose: 3.64 × 10^10^ gc/mouse; low-dose: 1.3 × 10^10^ gc/mouse) and untreated *Kcnq4*^*+/G322S*^ mice. Scale bar, 20 µm. (**B**,** C**) Quantification of IHCs (**B**) and OHCs (**C**) per 100 μm in treated (high dose, *n* = 6; low dose, *n* = 4) and untreated (*n* = 7) *Kcnq4*^*+/G322S*^ mice. (**D**) Representative immunofluorescence images of SGNs in the apical, middle, and basal turns of the cochleae sections from *Kcnq4*^*+/+*^, treated (high-dose: 3.64 × 10^10^ gc/mouse; low-dose: 1.3 × 10^10^ gc/mouse), and untreated *Kcnq4*^*+/G322S*^ mice. Scale bar, 50 µm. (**E**) Quantification of SGN density in cochlear sections in (**D**) from *Kcnq4*^*+/+*^ (*n* = 3), treated (high dose, *n* = 3; low dose, *n* = 3), and untreated (*n* = 3) *Kcnq4*^*+/G322S*^ mice. (**F**) Representative immunofluorescence images of afferent/efferent nerve fibers from *Kcnq4*^*+/+*^, treated (high-dose: 3.64 × 10^10^ gc/mouse; low-dose: 1.3 × 10^10^ gc/mouse), and untreated *Kcnq4*^*+/G322S*^ mice. The red dashed box demarcates the region of afferent nerve fibers innervated by IHCs, while the red arrow indicates a focal loss of innervation. Scale bar, 20 μm. Data were presented as mean ± SEM. Statistical analysis was performed using two-way ANOVA with Bonferroni’s post hoc test. *p < 0.05; **p < 0.01; ***p < 0.001; ****p < 0.0001; ns not significant (Exact *P* values are provided in Appendix Table S1). Source data are available online for this figure.

Restoration of auditory function prompted an assessment of morphological alterations in cochlear structures. To this end, cochlear whole mounts from *Kcnq4*^*+/G322S*^ mice treated with high- and low-dose AAV_ABE8e_N + C_322 were examined 8 weeks after injection. Both high- and low-dose regimens comparably preserved OHC survival in mutant mice (Fig. 5A,C). The most robust rescue was observed in the basal turn, where OHC numbers increased by 4.67-fold and 4.3-fold in the high- and low-dose groups, respectively. This spatial bias is consistent with the preferential AAV transduction and higher editing efficiencies typically achieved in the cochlear base (Fig. EV3A,B; Appendix Fig. S5A). In the middle turn, OHC counts increased by ~1.25-fold in both treatment groups, whereas more modest improvements were observed in the apical turn (0.75-fold in the high-dose group and 0.67-fold in the low-dose group). In contrast, IHC survival exhibited a mild decline overall, with statistically significant protection confined to the apical and middle turns in the high-dose group (Fig. 5A,B). Notably, the tonotopic gradient of hair cell preservation closely mirrored the auditory brainstem response (ABR) outcomes, with the greatest rescue at high frequencies, intermediate effects at mid frequencies, and minimal preservation at low frequencies (Figs. 4B,F and 5A–C). SGNs, which are essential for auditory signal transmission, were subsequently evaluated. While SGN degeneration was not detectable in 4-week-old mutant mice (Fig. EV2A,B), a significant reduction in SGN density became evident by 8 weeks, with decreases of 35, 21, and 23% in the apical, middle, and basal turns, respectively, in untreated *Kcnq4*^*+/G322S*^ mice relative to WT controls. AAV_ABE8e N + C_322 treatments markedly preserved SGN density across all cochlear turns in both dose groups, with values approaching those observed in WT mice (Fig. 5D,E). Furthermore, at 8 weeks of age, untreated *Kcnq4*^*+/G322S*^ mice exhibited pronounced abnormalities in both the density and architecture of afferent and efferent nerve fibers, characterized by pathological swelling, aberrant sprouting, and severe disorganization. In regions of extensive IHC loss, nerve fibers were entirely absent. By contrast, treated mutant mice displayed substantially improved neural organization, with afferent and efferent fibers forming more continuous and structurally integrated connections with the surviving HCs (Fig. 5F), consistent with the observed recovery of ABR Wave I responses (Fig. 4K,L). Collectively, these findings demonstrate that AAV_ABE8e_N + C_322 treatment effectively preserves HCs, SGNs, and cochlear neurofilament innervation in *Kcnq4*^*+/G322S*^ mice—structural and neural improvements that are closely associated with the restoration of auditory function.

### Restoration of K^+^ current properties in OHCs of AAV_ABE8e_N+C_322-treated *Kcnq4*^*+/G322S*^ mice

**Figure 6:**
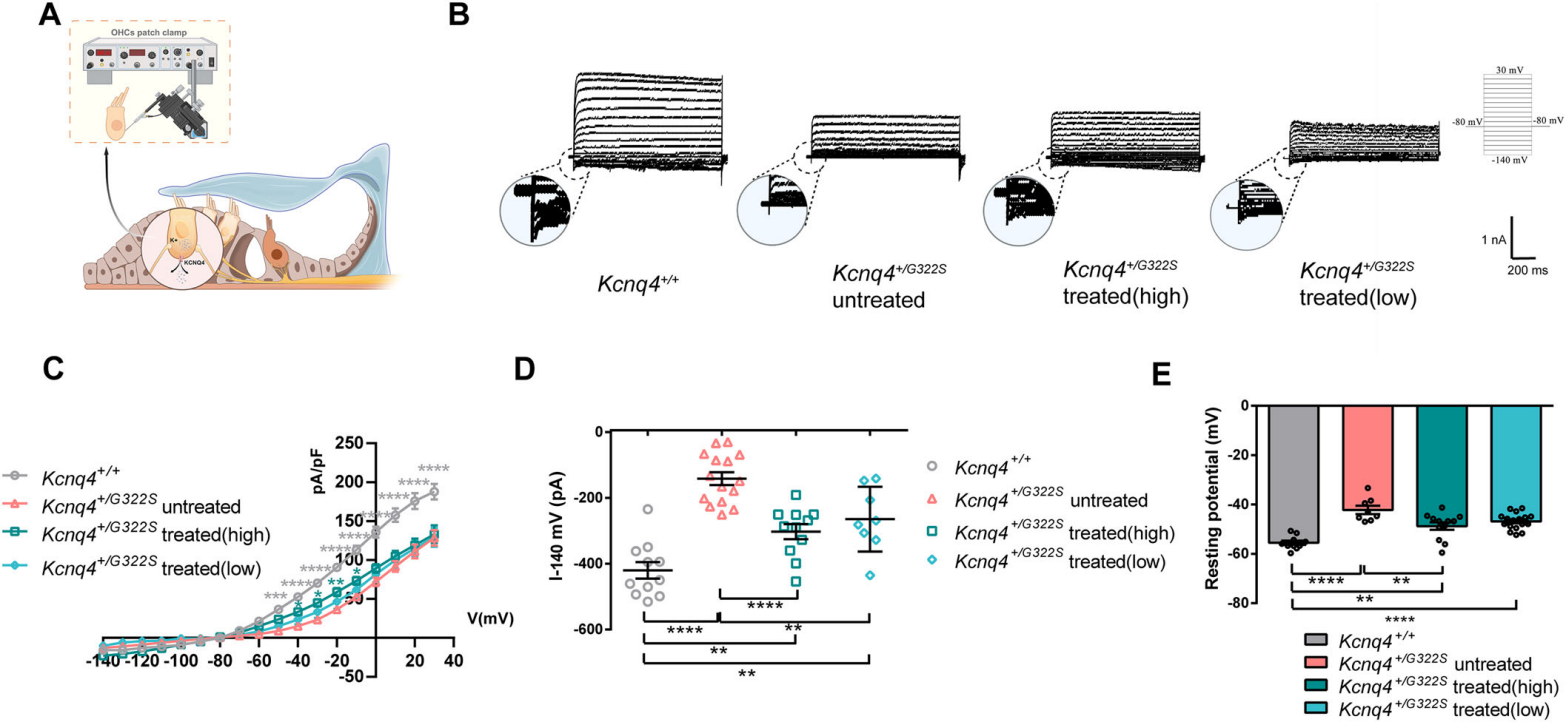
Restoration of K^+^ current in OHCs of *Kcnq4*^*+/G322S*^ mice by AAV_ABE8e_N + C_322. (**A**) Schematic of whole-cell voltage−clamp recordings in OHCs from *Kcnq4*^*+/G322S*^ and *Kcnq4*^*+/+*^ mice. (**B**) Representative current traces from *Kcnq4*^*+/+*^controls, treated (high-dose: 3.64 × 10^10^ gc/mouse; low-dose: 1.3 × 10^10^ gc/mouse), and untreated *Kcnq4*^*+/G322S*^ mice. The transient inward current peak at −140 mV is highlighted. (**C**) Peak current−voltage (I /V) curves illustrating KCNQ4 channel function in *Kcnq4*^*+/+*^controls (*n* = 11 cells from 8 mice), treated (high dose, *n* = 11 cells from 8 mice; low dose, *n* = 8 cells from 7 mice), and untreated (*n* = 15 cells from 10 mice) *Kcnq4*^*+/G322S*^ mice. Significance denotes difference between Treated vs. Untreated (dark teal for high-dose group) and WT vs. Untreated mice (gray). (**D**) Quantification of peak transient inward current at −140 mV in *Kcnq4*^*+/+*^controls (*n* = 11 cells from 8 mice), treated (high dose, *n* = 11 cells from 8 mice; low dose, *n* = 8 cells from 7 mice), and untreated (*n* = 15 cells from 10 mice) *Kcnq4*^*+/G322S*^ mice. (**E**) Quantification of rest membrane potential in *Kcnq4*^*+/+*^controls (*n* = 10 cells from 5 mice), treated (high dose, *n* = 12 cells from 6 mice; low dose, *n* = 17 cells from 10 mice) and untreated (*n* = 8 cells from 4 mice) *Kcnq4*^*+/G322S*^ mice. Data were presented as mean ± SEM. Statistical analysis was performed using one-way or two-way ANOVA with Bonferroni’s post hoc test. *p < 0.05; **p < 0.01; ***p < 0.001; ****p < 0.0001 (Exact *P* values are provided in Appendix Table S1). Source data are available online for this figure.

To evaluate functional recovery, whole-cell voltage‒clamp recordings were performed on OHCs from the apical cochlear turn of ex vivo preparations from P9–P14 mice (Fig. 6A). Current‒voltage (I/V) relationships were analyzed to characterize the K^+^ current (I_K,n_). *Kcnq4*^*+/G322S*^ mutants presented significant reductions in I_K,n_ density compared with WT controls from −60 to +30 mV (Fig. 6B,C). Mutant OHCs also lacked the characteristic transient inward K^+^ current during hyperpolarization, with a marked reduction in peak inward currents at −140 mV, indicating profound functional impairment (Fig. 6B,D) (Kharkovets et al, 2006; Kimitsuki et al, 2010). Resting membrane potential (RMP) measurements further revealed a pronounced depolarization in mutant OHCs, with RMP values elevated by ~25% relative to WT controls (Fig. 6E). This depolarized membrane state is consistent with impaired KCNQ4-mediated potassium conductance and underscores a fundamental disruption of OHC electrophysiological homeostasis caused by the G322S mutation.

Following AAV_ABE8e_N + C_322 treatment, high-dose–treated *Kcnq4*^*+/G322S*^ mice exhibited a significant increase in I_K,n_ current density within the physiologically relevant voltage range (–40 to –10 mV) compared with untreated mutants, whereas the low-dose group showed a similar but statistically non-significant trend (Fig. 6B,C). Notably, both treatment regimens robustly restored the characteristic transient inward K⁺ currents, with current amplitudes at –140 mV increased by 113 and 87% in the high- and low-dose groups, respectively (Fig. 6B,D). Consistent with these improvements, AAV_ABE8e_N + C_322 treatment partially normalized the membrane polarization of mutant OHCs, as reflected by restoration of RMP by 17% (*P* = 0.0061) in the high-dose group and 12% (*P* = 0.0630) in the low-dose group relative to untreated mutants (Fig. 6E). Together, these electrophysiological restorations indicate that base editing–mediated correction of *Kcnq4* partially rescues KCNQ4 channel function and re-establishes key biophysical properties of OHCs, thereby providing a mechanistic basis for the observed recovery of auditory function in vivo.

### Safety evaluation of AAV_ABE8e_N+C_322 therapy

**Figure 7:**
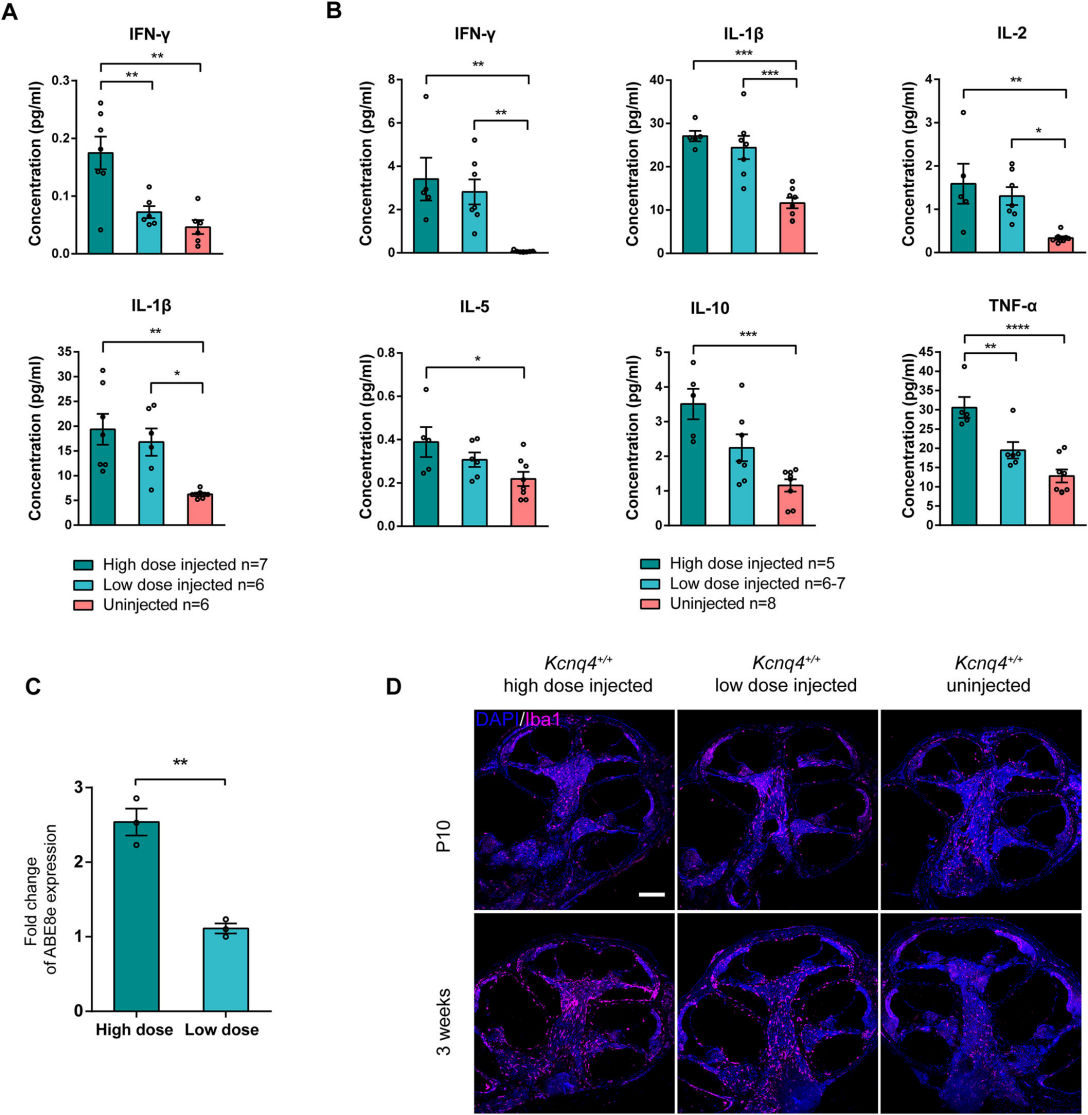
Assessment of cochlear inflammatory cytokines and macrophage recruitment. (**A**) Significantly upregulated cochlear inflammatory cytokines at P10 following low- and high-dose AAV_ABE8e_N + C_322 administration in *Kcnq4*^*+/+*^ mice (high-dose, *n* = 7; low-dose, *n* = 6; uninjected, *n* = 6). (**B**) Inflammatory cytokines significantly elevated in the cochlea at 3 weeks post-injection in *Kcnq4*^*+/+*^ mice (high-dose, *n* = 5; low-dose, IL-5, TNF-α*n* = 6, others *n* = 7; uninjected, *n* = 8). (**C**) Relative quantification of cochlear ABE8e mRNA by RT-qPCR at 3 weeks after AAV_ABE8e_N + C_322 administration, normalized to the low-dose group (*n* = 3). (**D**) Representative immunofluorescence images of cochlear macrophages at P10 and 3 weeks post-injection in *Kcnq4*^*+/+*^ mice receiving low or high doses of AAV_ABE8e_N + C_322. Data were presented as mean ± SEM. Statistical analysis was performed using unpaired *t*-tests or one-way ANOVA with Bonferroni’s post hoc test. *p < 0.05; **p < 0.01; ***p < 0.001; ****p < 0.0001 (Exact *P* values are provided in Appendix Table S1). Source data are available online for this figure.

We first evaluated the impact of AAV_ABE8e_N + C_322 administration on cochlear inflammation and macrophage recruitment in *Kcnq4*^*+/+*^ mice. The high-dose group exhibited an ~2.54-fold increase in ABE8e expression in the cochlea compared to the low-dose group (Fig. 7C). At P10, only two proinflammatory cytokines—IFN-γ and IL-1β—were significantly upregulated in the cochlea following low- or high-dose AAV administration, with no apparent increase in cochlear macrophage accumulation at this stage (Fig. 7A,D; Appendix Fig. S7A). However, by 3 weeks of age, six out of ten profiled inflammatory cytokines/chemokines were markedly elevated in treated cochleae, accompanied by a significant increase in macrophage infiltration compared to uninjected controls. Notably, the extent of cytokine/chemokine upregulation and macrophage accumulation was greater in the high-dose group than in the low-dose group (Fig. 7B,D; Appendix Fig. S7B).

The systemic safety of AAV_ABE8e_N + C_322 was also assessed through comprehensive serum biochemical and immunological analyses of *Kcnq4*^*+/+*^ mice four weeks after high-dose administration. No significant hepatic or renal function alterations were detected compared with those in uninjected controls (Appendix Fig. S7C). The evaluation of six inflammatory markers revealed no evidence of systemic immune activation (Appendix Fig. S7D). Survival analysis revealed comparable survival rates across high-dose, low-dose, and untreated *Kcnq4*^*+/G322S*^ mice (Appendix Fig. S7E), highlighting the tolerability of the treatment. These findings collectively underscore that, despite localized cochlear inflammation and immune activation, AAV_ABE8e_N + C_322 administration is well tolerated in mice, supporting its potential as a viable therapeutic strategy for *KCNQ4*-related hearing loss.

## Discussion

Durable and substantial auditory restoration remains the primary goal of HHL therapy. In this study, we report dual AAV-mediated delivery of ABE8e to correct the *Kcnq4* c.964 G > A (p.G322S) mutation in a murine model, achieving unprecedented editing efficiencies in the cochlear organ of Corti. These outcomes substantially surpass previous benchmarks of 0.6–7.14% for *Kcnq4* disruption (Cui et al, 2022; Noh et al, 2025a; Noh et al, 2022) or 0.82–4.05% for the correction or disruption of other deafness genes within 3 weeks after treatment (Cui et al, 2024; Gao et al, 2018; Gyorgy et al, 2019; Hu et al, 2025; Wu et al, 2021; Xue et al, 2022a; Yeh et al, 2020; Zheng et al, 2022). This exceptional efficiency can be attributed to the high transduction capability of AAV-ie combined with the enhanced editing efficiency of ABE8e (Richter et al, 2020; Tan et al, 2019). Although the broad transduction profile of AAV-ie may present challenges for traditional gene replacement therapies owing to ectopic transgene expression, its extensive mutation-correction capacity across diverse cell types offers significant advantages in the context of precision gene editing. This is particularly relevant for *Kcnq4*, which is expressed in HCs of the cochlea and vestibule, SGNs, cochlear nuclei, and inferior colliculus (Kharkovets et al, 2000; Peixoto Pinheiro et al, 2021), making widespread correction of pathogenic mutations highly beneficial.

Given the functional and mechanistic heterogeneity among deafness-associated genes (Jiang et al, 2023), direct comparisons of auditory outcomes across gene targets may be inappropriate when evaluating therapeutic efficacy. Previous studies utilizing the *Kcnq4* disruption strategy reported auditory recovery within 7–12 weeks, achieving maximum ABR threshold reductions of 20 dB SPL at 6 kHz and 18.5 dB SPL at 32 kHz (Cui et al, 2022; Jang et al, 2025; Noh et al, 2025a; Noh et al, 2022). In targeting the *Kcnq4* p.W276S mutation, eVLP-mediated delivery markedly enhanced SpCas9-induced indel formation at the mutant DNA locus within the inner ear. However, despite increased editing activity, this strategy failed to confer greater therapeutic benefit in terms of hearing restoration (Noh et al, 2025a; Noh et al, 2022). In contrast, our approach yielded sustained hearing restoration for more than 32 weeks, with a maximum average threshold reduction of 49.09 dB SPL at 32 kHz, which was observed at 20 weeks post-treatment (Fig. 4C). This markedly prolonged functional rescue is attributable to precise mutation correction, enabled by the substantially improved editing efficiency achieved in this study. Moreover, *Kcnq4* contributes to both hereditary and age-related hearing loss (Jeng et al, 2020). While gene disruption strategies mitigate dominant-negative effects, they fail to restore normal biallelic expression. In contrast, precise mutation correction, as demonstrated here, has the potential to restore Kcnq4 function and biallelic expression, which may underlie the more robust and sustained auditory recovery observed. Furthermore, as loss-of-function mutations account for a substantial fraction of HHL (Jung et al, 2017), precision gene correction strategies hold promise for broader therapeutic applications. Enhancing editing efficiency and implementing a precise mutation correction strategy may offer advantages for dominant-negative mutations, especially those involving a combination of dominant-negative and haploinsufficiency pathogenic pathways (Kamada et al, 2006).

Dose-dependent differences in therapeutic outcomes emerged within 12 weeks post-injection (Figs. 4A,B and EV5A). Although the high-dose group exhibited accelerated early auditory recovery, functional decline became evident from 16 weeks, with ABR thresholds approaching untreated control levels by 28 weeks. In contrast, the low-dose group sustained auditory improvements over the same interval (Fig. EV5D,I). The accelerated late-stage deterioration observed in the high-dose cohort may reflect dose-dependent liabilities arising from the cumulative burden of editing-associated toxicity, AAV-driven immunogenicity, and prolonged editor expression. Consistent with this, the TadA module mediates transcriptome-wide RNA A-to-I editing beyond on-target DNA correction (Richter et al, 2020; Zhou et al, 2019), and the high-dose cohort exhibited an increased RNA off-target burden (Fig. EV4I; Appendix Fig. S5D,E), which may impose chronic stress on hair cell survival (Zhang et al, 2026). In parallel, AAV delivery and sustained transgene expression may activate innate and adaptive immune pathways (Chan et al, 2022; Hosel et al, 2012; Ishibashi et al, 2023; Muhuri et al, 2021; Chandler et al, 2019; Liu and Xu, 2023; Rai et al, 2020) and disturb proteostasis (Kim et al, 2006; Lange et al, 2016; Yasuno et al, 2024), together fostering a hostile microenvironment within cochlea. Early benefit in the high-dose group likely reflects superior editing efficiency, whereas sustained AAV burden and editor expression may ultimately cross a biological “tipping point”, accelerating hair cell loss. Consistently, our data reveal enhanced macrophage accumulation and elevated inflammatory cytokine expression in the high-dose group (Fig. 7). These results underscore the importance of optimizing the AAV dosage to balance therapeutic efficacy with long-term safety and durability in base-editing strategies for inner-ear gene correction.

Deafness-associated genes play critical roles in the development and maintenance of the auditory system (Jiang et al, 2023; Zhang et al, 2024). Most human *KCNQ4* pathogenic mutations lead to progressive hearing loss (Cui et al, 2022; Sloan-Heggen et al, 2016), creating a therapeutic window for intervention. In mice, *Kcnq4* expression in OHCs begins at P8 (Kharkovets et al, 2000), coinciding with the onset of I_K,n_ currents—which are critical for OHC function (Marcotti and Kros, 1999). In this study, both high- and low-dose AAV administration comparably restored hyperpolarization-evoked inward K⁺ currents and partially normalized RMP in OHCs. High-dose treatment produced a significant recovery of I_K,n_ density across defined voltage ranges (Fig. 6C,D), whereas the low-dose group showed a consistent upward trend in I_K,n_ density. Given that P9–P14 represents an early developmental window, the full functional impact of the low-dose regimen may not yet be realized, raising the possibility of greater recovery at later stages. Notably, the anatomical complexity and fragility of the mature cochlea pose substantial technical barriers to isolating intact basilar membranes with viable hair cells, thereby limiting direct electrophysiological assessment of OHC K⁺ channel activity at peak hearing restoration. Future work will aim to address these limitations.

Another limitation is the incomplete and spatially heterogeneous functional rescue despite near-saturating reporter transduction, pointing to an efficacy-threshold constraint imposed by post-entry bottlenecks rather than limited AAV access. In post-mitotic cochlear hair cells, productive base editing requires multiple rate-limiting steps beyond entry—including endosomal escape, capsid uncoating, nuclear import, second-strand synthesis (for ssAAV), and sustained nuclear availability of editor and gRNA—further restricted by chromatin accessibility and local DNA repair context (Nonnenmacher and Weber, 2012; Schep et al, 2024; Yeh et al, 2019). Additionally, the dual-AAV split-ABE design adds complexity: We selected an Npu intein-mediated strategy due to its superior kinetics and enhanced genomic and transcriptomic safety profiles compared to overlap-based DNA reconstitution and RNA trans-splicing. However, the reconstitution efficiency (75-80%) may still limit the overall editing outcomes despite efficient co-transduction (>90% OHCs; ~100% IHCs, Appendix Fig. S3) (Brovin et al, 2024); it’s noteworthy that even in single-plasmid-transfected cells, the editing efficiency is rarely ~50% (Fig. 2D), underscoring intrinsic limits of editor potency and locus accessibility (Ferreira et al, 2023; Tang et al, 2023; Tornabene et al, 2019). A second constraint arises from the temporal mismatch between disease progression and base editing pharmacodynamics. Although editing efficiency continued to accumulate over time (Appendix Fig. S2C,D; Fig. EV4A; Appendix Fig. S5A), substantial OHC loss had already occurred by P14 (53% apex; 42% mid, Appendix Fig. S2B). At this stage, the low-dose heterozygous group—which ultimately exhibited more durable long-term outcomes—had not yet reached the higher editing efficiencies observed in the high-dose cohort (Fig. EV4A). In contrast, the early restoration of auditory function in the high-dose group (~17% cochlear correction at 3 weeks) likely represents a practical editing threshold required to initiate functional rescue in this model. The late-stage hearing decline in the low-dose group appears to be driven by two interrelated processes. First, the progressive loss of unedited HCs likely triggers chronic inflammation and recruitment of macrophages, creating a deleterious microenvironment that secondarily compromises the survival of neighboring rescued cells. Second, maladaptive remodeling of supporting cells following OHC loss destabilizes the intricate architecture of the organ of Corti, ultimately undermining cochlear structural integrity (Kaur et al, 2015; Pan et al, 2024; Taylor et al, 2012).

Notably, many HHL-associated genes, including *Kcnq4* and *Myo6*, directly predispose hair cells to degeneration (Cui et al, 2022; Xue et al, 2022b), limiting the robustness of auditory restoration compared with genes such as *Otof* or *Slc17a8*, which are not coupled to hair cell loss (Akil et al, 2012; Cui et al, 2024). These observations underscore that early, comprehensive, and spatially uniform base editing—maximizing hair cell preservation during the initial therapeutic window—is critical for sustaining long-term cochlear homeostasis and durable hearing. To mitigate the intrinsic risks of AAV-mediated base editing, future strategies should prioritize (i) lowering effective vector dose via editor optimization, including compact or hypercompact Cas9 orthologs or enhanced catalytic potency, while acknowledging residual off-target and chronic stress liabilities (Davis et al, 2022); and (ii) adopting transient “hit-and-run” delivery modalities such as mRNA-LNP, RNP, or VLPs to minimize prolonged off-target exposure and immunogenicity (Hollidge et al, 2022; Pardi et al, 2015). Supporting this approach, LNP-based delivery demonstrated safety advantages in our hereditary angioedema studies (Wang et al, 2025b), and engineered VLP systems have recently shown improved in vivo editing efficiency relative to conventional AAVs, although inner-ear translation remains constrained by cell-type specificity and transduction efficiency (Noh et al, 2025b; Noh et al, 2022).

The sgRNA used here differs from the human-targeting guide by only two synonymous nucleotides, and the human-specific sgRNA corrected 28.6 ± 0.5% of *KCNQ4* p.G321S in HEK293T cells (Fig. EV1A,D), underscoring strong translational potential. Clinical translation, however, hinges on three interdependent factors: vector dose, editor design, and immune control. Dose selection must balance therapeutic efficacy with systemic exposure, immunogenicity, and editor-associated toxicity, as evidenced by human and NHP AAV studies (Lv et al, 2024; Qi et al, 2024a; Qi et al, 2025b; Valayannopoulos et al, 2025; Wang et al, 2024). Editor optimization—through compact Cas orthologs, enhanced catalytic potency, and narrowed activity windows—can reduce vector load and off-target risks while facilitating single-AAV implementation (Bamidele et al, 2024; Davis et al, 2022; Kim et al, 2022; Kweon et al, 2023; Valdez et al, 2025). Immune management, including baseline NAb screening, longitudinal monitoring, and timely immunomodulation, is essential to mitigate inflammatory or complement-driven adverse events (Guillou et al, 2022; Wang et al, 2025a). Collectively, these integrated strategies provide a rational framework to achieve both enhanced safety and long-term efficacy in clinical translation.

In summary, we established a DFNA2 mouse model harboring the human *KCNQ4* p.G321S mutation. Dual AAV-mediated ABE8e delivery achieved precise mutation correction with exceptional editing efficiency in the cochlear organ of Corti, yielding auditory restoration that exceeded prior *Kcnq4* disruption strategies in both magnitude and durability. These results demonstrate the potential of precise *Kcnq4* mutation correction for sustained hearing recovery and provide a conceptual framework for base editor-based therapeutics in *KCNQ4*-related hearing loss.

## Methods

**Table:** Reagents and tools table

Reagent/resource	Reference or source	Identifier or catalog number
**Experimental models**		
FVB/NJ-*Kcnq4*^*G322S*^ mice	GemPharmatech	N/A
FVB/NJ mice	GemPharmatech	#N000026
HEK293T(*H. sapiens*)	ATCC	#CRL-3216
HEK293T–*Kcnq4*^*G322S*^ cell line	This study	N/A
HEK293T–*KCNQ4*^*G321S*^ cell line	This study	N/A
Neuro-2a(*M. musculus*)	ATCC	#CCL-131
**Recombinant DNA**		
ABE/sgRNA expression plasmids	Addgene	#136300; #138489; #138491; #138492; #152989; #152990; #47511
Cbh_v5 AAV-ABE N-terminal/C-terminal	Addgene	#137177; #137178
AAV plasmids: AAV_ABE8e_N; AAV_ABE8e_C_322; AAV_ABE8e_C_nosgRNA	This study	N/A
**Antibodies**		
Myosin-VIIa rabbit polyclonal antibody	Proteus	cat# 25-6790
Chicken anti-NeurofilamentH polyclonal antibody	Sigma-Aldrich	cat# AB5539
Rabbit anti-Iba1 antibody	Abcam	cat# AB178847
Mouse anti-Tubulin β3 antibody	BioLegend	cat# 657401
Alexa Fluor Plus 647 donkey anti-rabbit IgG	Invitrogen	cat# A32795
Alexa Fluor 488 donkey anti-mouse IgG	Invitrogen	cat# A-21202
Alexa Fluor 488 goat anti-chicken IgY	Invitrogen	cat# A-11039
Alexa Fluor 555 donkey anti-mouse IgG	Invitrogen	cat# A-31570
**Oligonucleotides and other sequence-based reagents**		
Detailed in Tables EV2–5	This study	Tables EV2–5
**Chemicals, enzymes and other reagents**		
Animal Genomic DNA Quick Extraction Kit	Beyotime	cat# D0065S
2× KeyPo Master Mix (high-fidelity)	Vazyme	cat# PK511-01
Agarose	BBI LIFE SCIENCES	cat# A600014
NovoRec® Plus One-Step PCR Cloning Kit	Novoprotein	cat# NR006-R040
KLD Enzyme Mix	New England Biolabs	cat# M0554
Polybrene	Beyotime	cat# C0351
Puromycin	Beyotime	cat# ST551
DMEM	Gibco	cat# 11965092
Fetal bovine serum (FBS)	cellbox	cat# AUS-01S-02
Penicillin/streptomycin	Gibco	cat# 15140122
OriFect Transfection Reagent	Oriscience	cat# CC101
Blasticidin	Beyotime	cat# ST018
PBS	Gibco	cat# 10010023
RNA isolator Total RNA Extraction Reagent	Vazyme	cat# R401-01
PrimeScript™ FAST RT Reagent Kit with gDNA Eraser	Takara	cat# RR092A
Tissue genome extraction kit	TIANGEN	cat# GDP304
Paraformaldehyde (PFA) 4%	Biosharp	cat# BL539A
EDTA 10% decalcifying solution	Zhonghui Hecai Biomedicine Technology	cat# PI014
Triton X-100	BBI LIFE SCIENCES	cat# A110694-0100
DAPI	Beyotime	cat# C1006
Antifade Mounting Medium	Beyotime	cat# P0128
Glutaraldehyde 2.5%	biosharp	cat# BL1964A
Osmium tetroxide 1%	Zhongjingkeyi Technology	N/A
Tannic acid 2%	Macklin	cat# T834649
V-PLEX Proinflammatory Panel 1 mouse kit	MSD (USA)	cat# K15048D
Tissue cell lysis buffer	Absin (China)	cat# abs9225
Multiplex ELISA Kit for Mouse Cytokine Panel 1 (6-Plex)	BOSTER (USA)	cat# MEK1011
**Software**		
LAS_X	Leica Microsystems	v.3.5.7
BioSigRZ software	Tucker-Davis Technologies, Alachua, FL	v.5.7.6
ImageJ	https://imagej.net/ij/	
CRISPResso2	Clement et al.	v2.3.3
Cas-OFFinder	Bae et al.	v2.4.1
GATK HaplotypeCaller	Broad Institute	v4.1.2.0
SOAPnuke	BGI	
R	R Project	v4.4.1
**Other**		
Leica STELLARIS 5	Leica Microsystems	
JSM-IT700HR	JEOL	
K850 Critical point drying system	Quorum (UK)	
Smart Coater	JEOL (Japan)	
RZ6 acoustic system	Tucker-Davis Technologies (USA)	
MF1 magnetic speaker(s)	Tucker-Davis Technologies	
ER 10B+ microphone	Etymotic Research	
NL2020 Nanoliter injection system	World Precision Instruments (USA)	
Eyelid retractor	World Precision Instruments (USA)	cat# 501897
Glass micropipette	World Precision Instruments (USA)	cat# 504949
EPC10 Patch-clamp amplifier	HEKA Elektronik (Germany)	
Illumina NovaSeq X Plus (PE150)	Illumina/Novogene service	
DNBSEQ platform (PE150)	BGI	
AAV production	OBiO Technology	
RNA-seq sequencing	BGI Genomics	
WGS service	BGI-tech	
Amplicon HTS sequencing	Novogene	

### Construction of the *Kcnq4* p.G322S mouse model

This study complied with NIH guidelines and was approved by the Institutional Animal Care and Use Committee (IACUC) of West China Hospital, Sichuan University (Approval No. 2020134A). Given prior reports on the use of gene editing to disrupt mutant alleles, the aim of this study is to investigate whether precise correction using base editors can restore hearing in mice more effectively. CRISPR/Cas9-mediated knock-in technology was employed to generate *Kcnq4* c.964 G > A (p.G322S) mutant mice on an FVB/NJ background (MGP_FVBNJ_T0067200.1) by GemPharmatech Co., Ltd. (Nanjing, China). The mutation, located in exon 7, replaces GGC with AGC, and a synonymous mutation (CGG > AGA) at codon 339 is introduced to prevent re-cleavage (Fig. EV1). Briefly, targeted sgRNAs, Cas9 mRNA, and the donor template were injected into fertilized FVB/NJ eggs to generate F0 *Kcnq4*^*G322S*^ mice, which were then bred with wild-type mice to produce stable F1 progeny for further study. The mice were housed in a specific pathogen-free (SPF) facility at West China Hospital, with controlled temperature, humidity, and a 12-h light/dark cycle. For genotyping, tissue samples (e.g., tail or toe clips) were collected, and genomic DNA was extracted using the Animal Genomic DNA Quick Extraction Kit (Beyotime, China). PCR was performed with high-fidelity 2×KeyPo Master Mix (Vazyme, China) with forward (5′-CGTGAGCATCTGTGCAG-3′) and reverse (5′-GCTCCCTTTTCAGCTGTC-3′) primers. The PCR products were purified by 1% agarose gel electrophoresis and validated by Sanger sequencing.

### Plasmid cloning

Expression plasmids for ABEs and sgRNAs were obtained from Addgene (https://www.addgene.org/, #136300, #138489, #138491, #138492, #152989, #152990, and #47511). The blasticidin resistance gene was incorporated into the ABE plasmids using the NovoRec® Plus One-Step PCR Cloning Kit (Novoprotein, China). The sgRNA constructs were subsequently cloned and inserted into the pFYF1320 vector using the KLD Enzyme Mix (New England Biolabs, USA) according to the manufacturer’s instructions, with the cloning primers listed in Table EV3. The dual-AAV backbones Cbh_v5 AAV-ABE N-terminal and Cbh_v5 AAV-ABE C-terminal were also sourced from Addgene (#137177 and #137178) (Levy et al, 2020). The ABE8e protein was cleaved at the 574th amino acid of nSpCas9 to generate N- and C-terminal fragments, which were subsequently cloned and inserted into the respective vectors by homologous recombination (Novoprotein). Targeting and non-targeting sgRNAs were inserted into the C-terminal vectors using KLD Enzyme Mix. All the plasmids were verified by Sanger sequencing to confirm their correct construction.

### Construction of the HEK293T- *Kcnq4*^*G322S*^ and HEK293T-*KCNQ4*^*G321S*^ cell lines

For in vitro screening of ABE systems and sgRNAs, a partial mouse *Kcnq4* sequence (Table EV3) harboring the p.G322S mutation (GGC to AGC), corresponding to the *Kcnq4* p.G322S mouse model, was cloned and inserted into a lentiviral vector. Further, another HEK293T-*KCNQ4* p.G321S cell line which carried the human p.G321S mutation (GGC to AGC) was constructed using the same strategy to validate its potential value on clinical translation. The lentivirus was packaged using a four-plasmid system. HEK293T cells were seeded in 10 cm dishes, and upon reaching ~70% confluence, they were transfected with the transfer plasmid containing *Kcnq4* p.G322S, along with the packaging plasmids PLP1, PLP2, and VSV-G at mass ratios of 6.5 µg:5 µg:3.5 µg:5 µg. After 72 h, the culture supernatant was harvested, centrifuged at 500×*g* for 15 min to remove debris, and filtered through a 0.45 µm PVDF membrane. The filtered viral supernatant was used to infect HEK293T cells pre-seeded in 12-well plates, with polybrene (5 µg/ml) to enhance transduction. After 72 h, puromycin (2 µg/ml) was added to select transduced cells. A polyclonal cell line was established, and then serially diluted in 96-well plates to isolate single-cell wells. Monoclonal HEK293T cells carrying the *Kcnq4* p.G322S target sequence were expanded for further use.

### Cell culture and transfection

HEK293T cells were maintained in Dulbecco’s modified Eagle Medium (DMEM, Gibco) supplemented with 10% fetal bovine serum (FBS) and 1× penicillin/streptomycin under standard conditions (5% CO₂ at 37 °C). Transfections were performed using OriFect Transfection Reagent (Oriscience, China) following the manufacturer’s protocol. The cells were seeded in 24-well plates 12 h before transfection and transfected with 1 µg of plasmid DNA, maintaining the following ratios: ABE:sgRNA = 750:250 ng, ABE8e_N: ABE8e_C = 500:500 ng. After 8 h, the medium was replaced with fresh complete medium (DMEM + 10% FBS) to promote cell viability. At 24 h post-transfection, blasticidin (10 µg/mL) was added to select the transfected cells. At 96 h post-transfection, the supernatant was discarded, and the cells were lysed with cell lysis buffer (1 M Tris-HCl, pH 7.5, 10% SDS, 0.8 U/mL Protease K) and incubated at 37 °C for 1 h. The lysates were heat-inactivated at 80 °C for 20 min, vortexed, and allowed to return to room temperature for subsequent analysis.

### DNA and RNA extraction from cochlear tissues

The cochleae were dissected and placed in ice-cold PBS immediately. The cochlear capsule was carefully removed to expose the soft tissue, which was then separated from the modiolus. For DNA extraction, the cochlear organ of Corti was lysed in 30–50 µL of cell lysis buffer and incubated at 37 °C for 1 h, with vortexing every 15 min. After incubation, the samples were heat-inactivated at 80 °C for 20 min and stored at −80 °C. For RNA extraction, the RNA isolator Total RNA Extraction Reagent (Vazyme) was used. Fresh tissue was immersed in lysis buffer, and RNA was extracted according to the manufacturer’s instructions. The extracted RNA was reverse transcribed into complementary DNA (cDNA) using the PrimeScript™ FAST RT Reagent Kit with gDNA Eraser (Takara) and stored at −80 °C. The DNA of the mouse brain was extracted using a tissue genome extraction kit (TIANGEN, China) according to the manufacturer’s instructions.

### AAV packaging

The plasmids AAV_ABE8e_N, AAV_ABE8e_C_322, and AAV_ABE8e_C_nosgRNA were packaged into AAV vectors by co-transfection of HEK293T cells with Rep-Cap (AAV-ie) and Helper plasmids. Viral titers were quantified by quantitative PCR using primers specific to the ITRs. For the low- and high-dose groups, AAV_ABE8e_N and AAV_ABE8e_C_322 were co-administered at total doses of 1.30 × 10^10^ and 3.64 × 10^10^ genome copies per mouse, respectively, at an N-to-C terminal vector ratio of 1:1, in a total injection volume of 1 µL. All the AAVs were obtained from OBiO Technology (Shanghai, China).

### Microinjection of neonatal mice

Inner ear injections were performed on P2–P3 mice by the posterior semicircular canal (PSCC) and round window membrane (RWM) approach, as previously described (Kim et al, 2024). Briefly, AAV vectors were injected into the inner ears of *Kcnq4*^*+/+*^, *Kcnq4*^*+/G322S*^, and *Kcnq4*^*G322S/G322S*^ pups. Neonatal mice were anesthetized on ice, and a small incision was made below the right ear using sterilized ophthalmic scissors. The surgical site was exposed with an eyelid retractor (World Precision Instruments, USA), and the surrounding fat and connective tissue was carefully dissected to expose the sternocleidomastoid muscle, which was then incised to reveal the posterior semicircular canal or round window membrane. A glass micropipette (World Precision Instruments) attached to a nanoliter injection system (NL2020, World Precision Instruments) was used to inject 1 µL of the viral mixture containing AAV_ABE8e_N and AAV_ABE8e_C_322/nonsgRNA at a rate of 5 nL/s. After injection, the incision was sealed with 3 M Vetbond Tissue Adhesive (3 M Science, USA), and the pup was placed on a heated pad at 37 °C for recovery before being returned to its cage upon regaining full consciousness.

### Immunofluorescence and confocal imaging

Mouse cochleae were fixed in 4% paraformaldehyde (PFA) at 4 °C overnight, followed by decalcification in 10% EDTA at room temperature for 48–72 h. Post-decalcification, the tissues were processed into cochlear whole mounts (apical, middle, and basal turns) and cochlear sections. The samples were permeabilized and blocked in 10% FBS with 0.1% Triton X-100 for 2 h and then incubated overnight at 4 °C with the following primary antibodies: Myosin-VIIa rabbit polyclonal antibody (1:1000, Proteus) for HCs, chicken anti-NeurofilamentH polyclonal antibody (1:1000, Sigma-Aldrich), rabbit anti-Iba1 antibody (1:500, abcam) for macrophages, mouse anti-Kcnq4 antibody (1:100, abcam) and mouse anti-Tubulin β3 (1:1000, BioLegend) for SGNs. Following primary antibody incubation, the samples were washed with PBS and incubated with secondary antibodies—Alexa Fluor Plus 647-conjugated donkey anti-rabbit IgG (1:500, Invitrogen) and Alexa Fluor 488-conjugated donkey anti-mouse IgG (1:500, Invitrogen), Alexa Fluor 488-conjugated Goat anti-Chicken IgY (1:500, Invitrogen) and Alexa Fluor 555-conjugated donkey anti-mouse IgG (1:500, Invitrogen)—for 2 h at room temperature, followed by nuclear staining with DAPI. Samples were mounted in Antifade Mounting Medium (Beyotime) and imaged on a Leica STELLARIS 5 confocal microscope (Leica Microsystems, Germany) using a ×63/1.40 oil-immersion objective, with or without digital zoom. Z-stack maximum projections were generated to compensate for tissue thickness in whole-mount preparations.

### Quantification of cochlear HCs and SGNs

The quantification of cochlear HCs and SGNs was performed using LAS_X software on *Kcnq4*^*+/+*^, *Kcnq4*^*+/G322S*^, and *Kcnq4*^*G322S/G322S*^ mice post-injection. The number of Myosin-VIIa^+^ and DAPI^+^ IHCs and OHCs per 100 μm were counted at the apical, middle, and basal turns of cochleae. Tubulin β3^+^ and DAPI^+^ SGNs were quantified in cochlear sections according to tonotopic regions to assess the number of cells corresponding to auditory frequencies (Noh et al, 2022).

### Scanning electron microscopy

Mouse cochleae were harvested, punctured at the apex, and perfused with 2.5% glutaraldehyde for overnight fixation at 4 °C. After fixation, the tissues were rinsed in PBS, decalcified in 10% EDTA for 48–72 h, and dissected into apical, middle, and basal turns. The samples were subsequently fixed in 1% osmium tetroxide for 2 h, stained with 2% tannic acid for 1 h, and dehydrated through a graded ethanol series (50–100%). Following critical point drying (Quorum, UK), the samples were mounted on stubs, sputter-coated with gold (Smart Coater, JEOL, Japan), and imaged at ×2000 and ×8000 magnifications using a JSM-IT700HR scanning electron microscope (JEOL, USA).

### Auditory test

ABR and DPOAE measurements were performed using the RZ6 acoustic system (Tucker-Davis Technologies, USA) (Cui et al, 2022). The mice were anesthetized by the intraperitoneal injection of xylazine (10 mg/kg) and ketamine (100 mg/kg) and were placed on a 37 °C heating pad to maintain thermoregulation. Following a 3–5 min stabilization, the animals were transferred to a soundproof chamber with continuous heating. ABR recordings were obtained via a closed-field setup. Reference, recording, and ground electrodes were placed subcutaneously at the cranial vault between the ears, the right pinna near the mastoid, and the dorsal rump, respectively. A sound delivery tube connected to an MF1 magnetic speaker (Tucker-Davis Technologies) was inserted into the external auditory canal. Tone burst stimuli (3 ms) were presented at frequencies from 4 to 32 kHz in half-octave intervals (4, 5.656, 8, 11.314, 16, 22.627, 32 kHz), with intensities ranging from 5 dB SPL to 90 dB SPL in 5 dB steps. Auditory signals were amplified by 10,000× and filtered from 300 Hz to 3 kHz. The ABR threshold was determined as the minimum sound intensity producing a detectable waveform, averaged from 400 tone bursts (ABR thresholds >90 dB SPL were capped at 100 dB SPL for analysis). For DPOAE, a custom probe containing two MF1 magnetic speakers and an ER 10B+ microphone (Etymotic Research) was inserted into the right external auditory canal. Primaries f1 and f2 (f2/f1 = 1.2) were presented with f2 spanning from 4 to 32 kHz in half-octave intervals, with L1−L2 set to 10 dB SPL. For each f2, L2 was incrementally varied from 20 to 80 dB SPL in 10 dB increments. DPOAE thresholds were defined as the f2 amplitude that generated a DPOAE response of 6 dB SPL above the noise floor, which was consistently below 0 dB SPL across all frequencies (DPOAE thresholds >80 dB SPL were capped at 90 dB SPL for analysis). The detailed parameters are shown in Table EV4. Mice were drawn from multiple litters and randomly assigned to experimental or control groups; to minimize litter effects, mice from different litters were distributed across groups. Right-ear (injected ear) hearing thresholds were measured for each mouse, and both sexes were included. All measurements were conducted by a single experimenter blinded to the group assignments to minimize potential bias. Following the experiments, the mice were placed on a 37 °C heating pad for recovery and returned to the breeding room once fully revived.

### Guide RNA-dependent off-target analysis

Putative gRNA-dependent off-target sites were predicted using Cas-OFFinder against the Mus musculus reference genome (GRCm38) (Bae et al, 2014). The top ten candidate loci (Fig. EV5E) were validated by HTS to assess A-to-G editing frequencies. Genome-wide off-target sites were also assessed on mouse-derived N2a cells using GUIDE-seq, as previously described (Malinin et al, 2021). Two candidate off-target sites were identified based on dsODN integration (Fig. EV5E) and validated by HTS to assess A-to-G editing frequencies.

### Transcriptome-wide A-to-I RNA editing in the cochlea evaluated by RNA-seq

Total RNA sequencing libraries were prepared and subjected to paired-end 150 bp sequencing by BGI Genomics (Wuhan, China). Raw sequencing reads were filtered with *fastp* (Chen et al, 2018a) and quality-checked using *FastQC*. Clean reads were aligned to the *Mus musculus* reference genome (GRCm39) using *STAR*, and gene-level quantification was performed with *RSEM* (Dobin et al, 2013; Li and Dewey, 2011). To assess RNA off-target effects induced by base editors, variants were called with *HaplotypeCaller* (GATK v4.1.2.0) on deduplicated, post-alignment reads. For each SNV, only sites with read depth >20 and absent in untreated controls were considered high-confidence variant calls (Cui et al, 2024). To interrogate sequence-context preference, detected A-to-I variants were intersected with a curated set of endogenous ADAR hotspot loci (Picardi et al, 2017). Variant functional consequences were annotated using Ensembl variant effect predictor (VEP) to evaluate genomic distribution and predicted impact (McLaren et al, 2016).

### Whole-genome sequencing

Cochleae from high-dose–injected and uninjected control mice were collected 3 weeks post-administration. Genomic DNA extraction, WGS library preparation, and sequencing were performed by BGI Genomics. Libraries were sequenced on the DNBSEQ platform to generate 150-bp paired-end reads at an average depth of ~100×. Raw reads were processed using SOAPnuke (BGI) to remove adapter contamination and low-quality sequences (-n 0.01 -l 20 -q 0.5 --adaMR 0.25 --polyX 50 --minReadLen 150) (Chen et al, 2018b). Reads were discarded if they contained ≥25% adapter sequence (allowing ≤2 mismatches), were shorter than 150 bp, had ≥1% ambiguous bases (N), contained polyX tracts >50 bp, or had ≥50% of bases with Phred quality <20. The remaining reads were retained as clean reads. Clean reads were aligned to the FVB/NJ reference genome using BWA (v0.7.15) (Li and Durbin, 2009). Germline variants were identified using Sentieon (GATK HaplotypeCaller implementation) and functionally annotated with ANNOVAR (Van der Auwera et al, 2013; Wang et al, 2010). To assess global mutational burden in an unbiased manner, single-nucleotide variant (SNV) frequencies and base-substitution spectra were compared between high-dose–treated and uninjected control samples.

### Analysis of editing and off-target efficiency

HTS was employed to assess gene editing efficiency and off-target effects. Specific primers, each associated with unique barcodes, were designed to amplify HEK293T DNA, cochlear organ of Corti DNA, or complementary DNA (cDNA) (Tables EV5 and EV6). These primers facilitated two-step PCR amplification to generate sequencing-ready libraries for evaluating editing outcomes. In the first PCR step, a 25 µl reaction mixture was prepared containing 0.5 µM each forward and reverse primer, 12.5 µl of 2 × Phanta Flash buffer, 0.5 µl of Phanta Flash Super-Fidelity DNA Polymerase (Vazyme), and 2 µl of sample DNA or cDNA. The PCR conditions were as follows: denaturation at 98 °C for 30 s, followed by 28 cycles of 98 °C for 10 s, 60 °C for 5 s, and 72 °C for 10 s, with a final extension at 72 °C for 2 min. The PCR products were purified using 2% agarose gel electrophoresis and extracted with the TIANgel Purification Kit (TIANGEN). In the second PCR step, indexed HTS adapters were added to the purified first-round PCR products using a 25 µl reaction mixture containing adapter-specific primers, 2 × Phanta Flash buffer, Phanta Flash Super-Fidelity DNA Polymerase, and 1 µl of the mixed purified first-round PCR product. The PCR program consisted of denaturation at 98 °C for 30 s, followed by ten cycles of 98 °C for 10 s, 60 °C for 5 s, and 72 °C for 10 s, with a final extension at 72 °C for 2 min. The final PCR products were again purified by gel electrophoresis and extracted with the TIANgel Purification Kit. The purified libraries were sequenced using the Illumina NovaSeq X Plus PE150 strategy at Novogene Co., Ltd. (Beijing, China). Sequencing data were demultiplexed based on barcodes and analyzed using CRISPResso2 software to quantify on-target editing efficiency and off-target modifications (Clement et al, 2019). On-target editing efficiency was calculated as the absolute correction rate, defined by subtracting the background G allele frequency in untreated controls from the G allele frequency in treated samples: (G/(A + G))%_Treated_ − (G/(A + G))%_Control_.

### Patch-clamp analysis of OHCs

Whole-cell voltage‒clamp recordings of K^+^ currents in OHCs were conducted using an EPC10 amplifier (HEKA Elektronik, Lambrecht/Pfalz, Germany). Patch pipettes were pulled from borosilicate glass capillaries and heat-polished to achieve tip resistances of 3–4 MΩ. The cochleae were acutely isolated and maintained in an ice-cold dissection solution containing (in mM): 144.6 NaCl, 5.5 KCl, 1 MgCl₂, 0.1 CaCl₂, 0.5 MgSO₄, 10.2 HEPES, and 3.5 L-glutamine, pH 7.2 (adjusted with NaOH). The apical turn of the cochlea was dissected, mounted onto a coverslip with a nylon net, and perfused with an external bath solution containing (in mM): 145 NaCl, 5.8 KCl, 0.9 MgCl₂, 1.3 CaCl₂, 0.7 NaH₂PO₄, 10 HEPES, and 5.6 D-glucose, pH 7.4 (adjusted with NaOH). The pipette solution consisted of (in mM): 135 KCl, 3.5 MgCl₂, 0.1 CaCl₂, 2.5 Na₂ATP, 5 HEPES, and 5 EGTA, pH 7.4 (adjusted with KOH). To record K^+^ currents in OHCs in the whole-cell configuration, the cells were held at −80 mV, with step voltages ranging from −140 to +30 mV in 10-mV increments. After breaking through the cell membrane to establish a giga-ohm seal, the series resistance (R_s) and membrane capacitance (C_m) were compensated. The current density‒voltage relationship was derived by normalizing the current to the cell capacitance. The cell capacitance was estimated using capacitive transients as an indirect measure of the cell size. Liquid junction potentials were measured and appropriately corrected.

### Cochlear inflammatory cytokine/chemokine assessment

Ten inflammatory parameters, including IFN-γ, IL-10, IL-12p70, IL-1β, IL-2, IL-4, IL-5, IL-6, KC/GRO, and TNF-α, were measured using electrochemiluminescence V-PLEX proinflammatory panel 1 mouse kit (MSD, USA). Briefly, Cochlear samples were lysed using tissue cell lysis buffer (Absin, China), yielding ~25 μL of supernatant. This supernatant was subsequently diluted threefold and subjected to further processing following the manufacturer’s instructions. Final concentrations were calculated using the MSD Discovery Workbench 4.0 software (MSD, USA).

### Serum biochemical and immune parameter measurement and survival analysis

Serum biochemical parameters in high-dose-injected and control *Kcnq4*^*+/+*^ mice were quantified by Lilai Biotechnology Co., Ltd. (Chengdu, China) using a BS-460 biochemical analyzer (Mindray, China). The parameters measured included ALT, AST, ALP, CREA-S, TP, ALB, LDH, UREA, and Glo. Serum immune markers (IFN-γ, IL-1α, IL-1β, IL-6, IL-10, and TNF-α) in these mice were assessed using the Multiplex ELISA Kit for Mouse Cytokine Panel 1 (6-Plex) (MEK1011, BOSTER, USA) according to the manufacturer’s instructions. Survival analysis was performed on high-dose, low-dose, and untreated *Kcnq4*^*+/G322S*^ mice over a 32-week period.

### Statistical analysis

Statistical analyses and data visualization were performed using R v4.4.1. The sample sizes, exact *p* values and the specific statistical tests employed are detailed in the figure legends. All replicates are biological unless stated otherwise. Randomization and blinding were used whenever applicable to minimize the effects of subjective bias.

### Graphics

The schematics in Figs. 1, 3, 6 and EV1; Appendix Fig. S1 and synopsis graphics were created with BioRender.com

## Supplementary information

Appendix

Table EV1

Table EV2

Table EV3

Table EV4

Table EV5

Table EV6

Peer Review File

Source data Fig. 1

Source data Fig. 2

Source data Fig. 3

Source data Fig. 4

Source data Fig. 5

Source data Fig. 6

Source data Fig. 7

Expanded View Figures

## Data Availability

The datasets produced in this study are available in the following database: Amplicon sequencing, RNA-seq and GUIDE-seq data: the NCBI sequence read archive PRJNA1420045. The source data of this paper are collected in the following database record: biostudies:S-SCDT-10_1038-S44321-026-00433-5.
