# Supplementary material for: Long-term restoration of auditory function in a DFNA2 mouse model by adenine base editing
Source: EMBO Mol Med. 2026 May 20;18(6):2293–321. doi: 10.1038/s44321-026-00433-5 (PMC13270111; doi:10.1038/s44321-026-00433-5)
Supplement: Supplementary file 1 — Appendix [file 44321_2026_433_MOESM1_ESM.pdf]

Appendix

Table of contents

Figures/Tables	Pages
Appendix Figure S1. Schematic overview of lentiviral *Kcnq4*^{G322S} stable monoclonal cell line generation and *in vitro* screening ABE-sgRNA systems.	2
Appendix Figure S2. Disease kinetics and editing pharmacodynamics.	3
Appendix Figure S3. Co-transduction evaluation of dual-AAV administration.	4
Appendix Figure S4. Morphological degeneration at later endpoint.	5
Appendix Figure S5. Editing efficiency, AAV distribution and advanced analysis of RNA off-target effects.	6-7
Appendix Figure S6. DNA off-target effects evaluated by WGS.	8
Appendix Figure S7. Safety assessment following AAV_ABE8e_N+C_322 administration.	9-10
Appendix Table S1. Exact *P* values	11-19

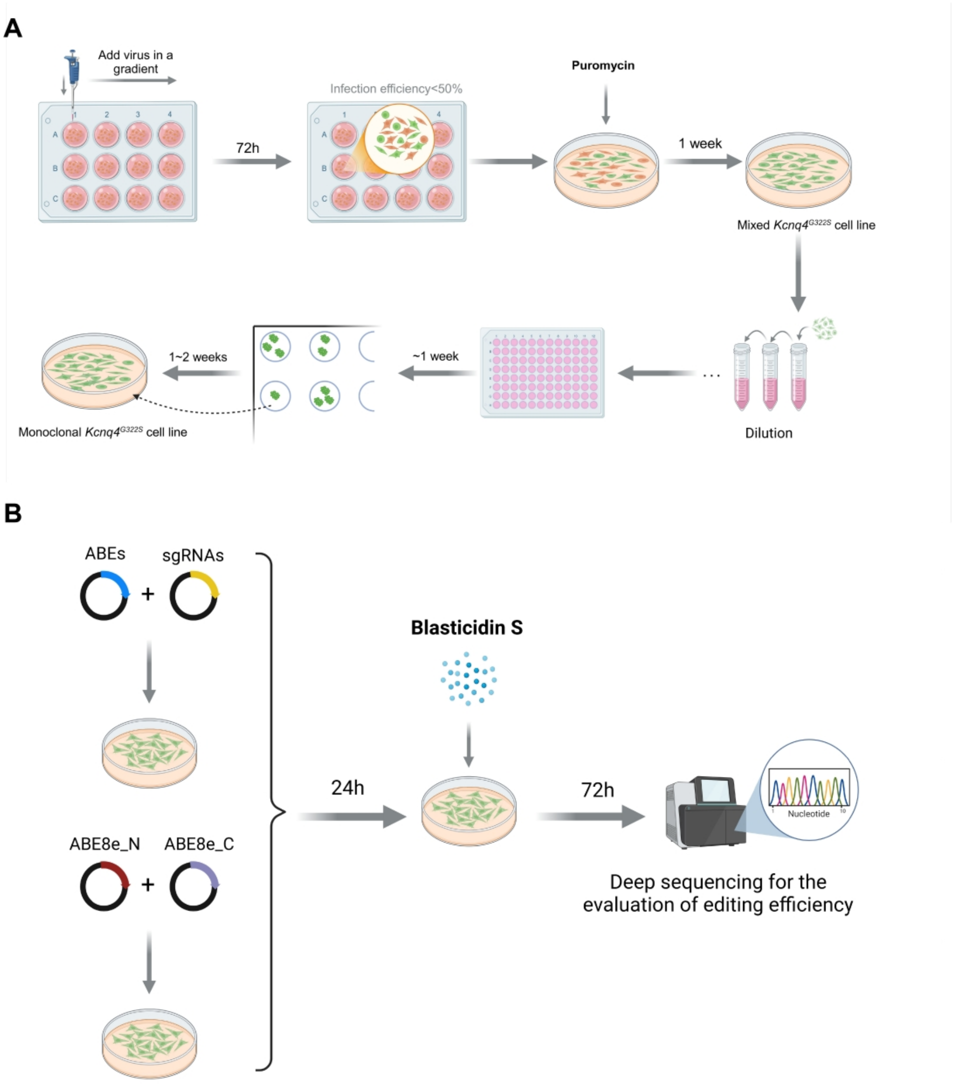

Appendix Figure S1. Schematic overview of lentiviral *Kcnq4*^{G322S} stable monoclonal cell line generation and *in vitro* screening ABE-sgRNA systems.

(A) Schematic illustrating the generation of a lentiviral-based *Kcnq4*^{G322S} stable monoclonal cell line in HEK293T cells. (B) Workflow for *in vitro* screening of ABE-sgRNA combinations, comparing editing efficiencies between full-length and split-intein ABE8e plasmids co-delivered with sgRNA1.

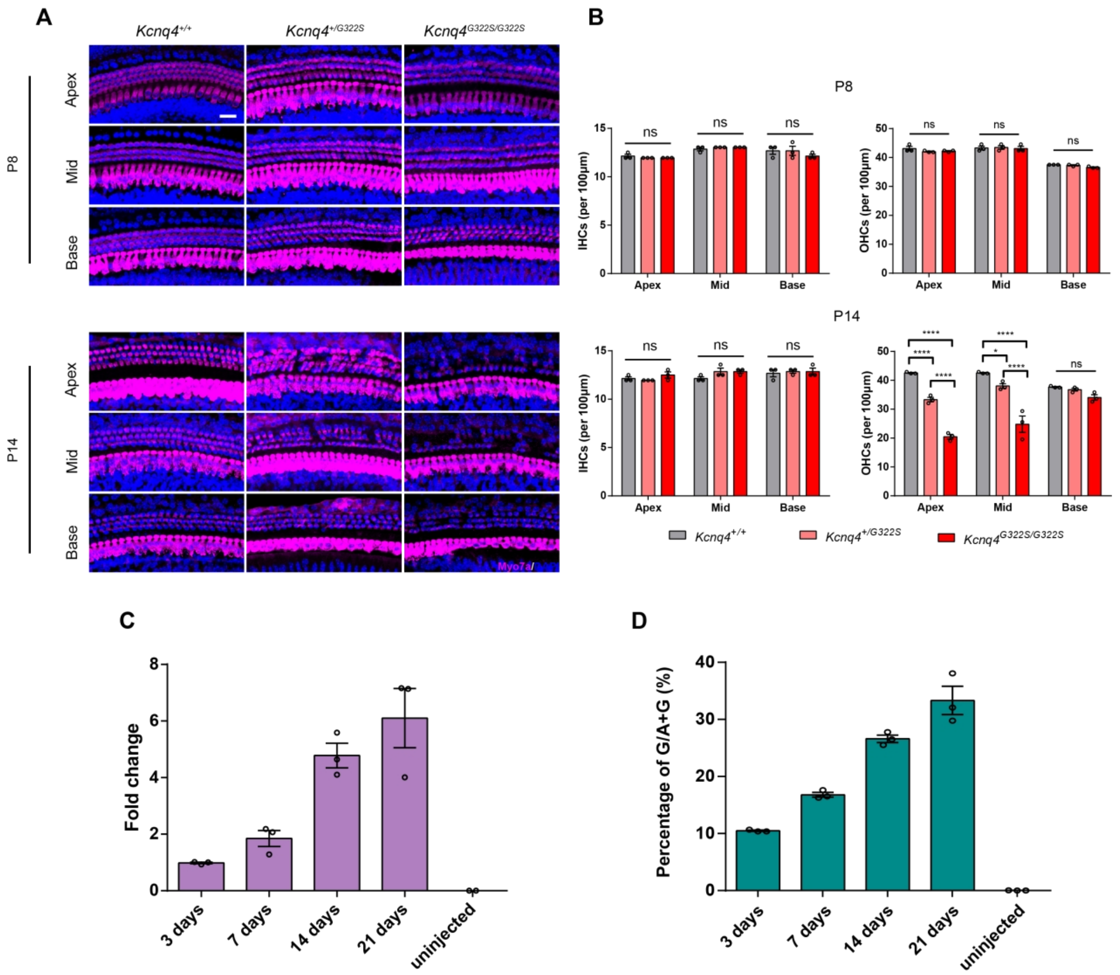

Appendix Figure S2. Disease kinetics and editing pharmacodynamics.

(A) Immunofluoresence images of the organ of corti in $Kcnq4^{+/+}$, $Kcnq4^{+/G322S}$ and $Kcnq4^{G322S/G322S}$ mice at P8 and P14. Scale bar, 20 μ m (B) quantification of IHCs and OHCs in $Kcnq4^{+/+}$, $Kcnq4^{+/G322S}$ and $Kcnq4^{G322S/G322S}$ mice at P8 and P14 (*n* = 3 per group at each time point). (C) Time course of ABE8e expression in $Kcnq4^{+/+}$ mice at different time points following AAV injection (*n* = 3 per group, 3.64 × 10^10 gc/mouse) and uninjected controls (*n* = 2). (D) The editing efficiency of $Kcnq4^{G322S/G322S}$ mice at time points corresponding to (C) after AAV injection (*n* = 3 per group, 3.64 × 10^10 gc/mouse) and uninjected controls (*n* = 3). Data are presented as mean ± SEM. Statistical analysis was performed using two-way ANOVA with Bonferroni's post hoc test. $*p < 0.05$; $**p < 0.01$; $****p < 0.0001$.

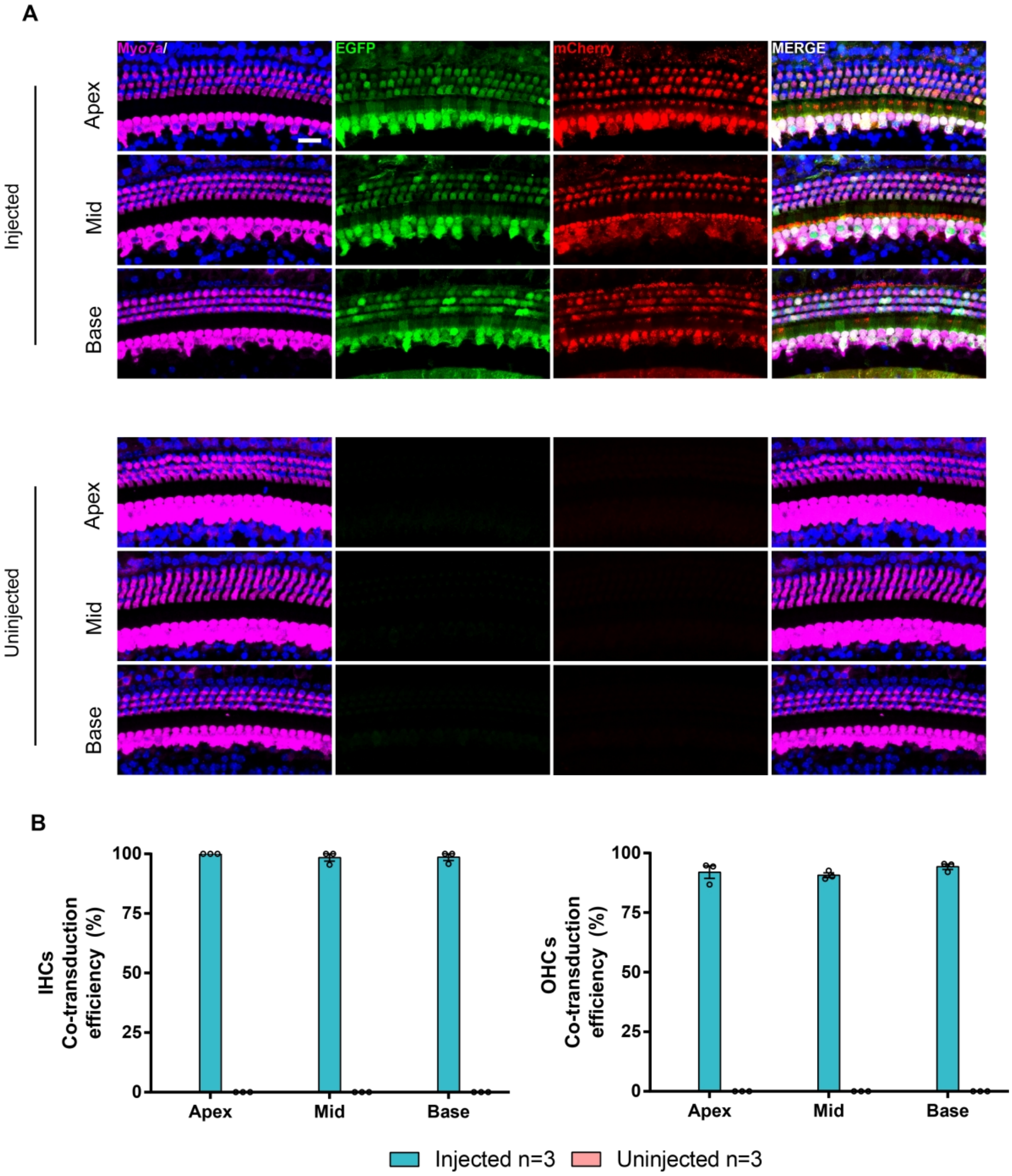

Appendix Figure S3. Co-transduction evaluation of dual-AAV administration.

(A) Immunofluorescence images *Kcnq4*$^{+/+}$ mice injected with AAV-ie-EGFP and AAV-ie-mcherry and uninjected controls. (B) Quantification of co-transduction efficiency of IHCs and OHCs in the injected (n = 3) and uninjected (n = 3) *Kcnq4*$^{+/+}$ mice.

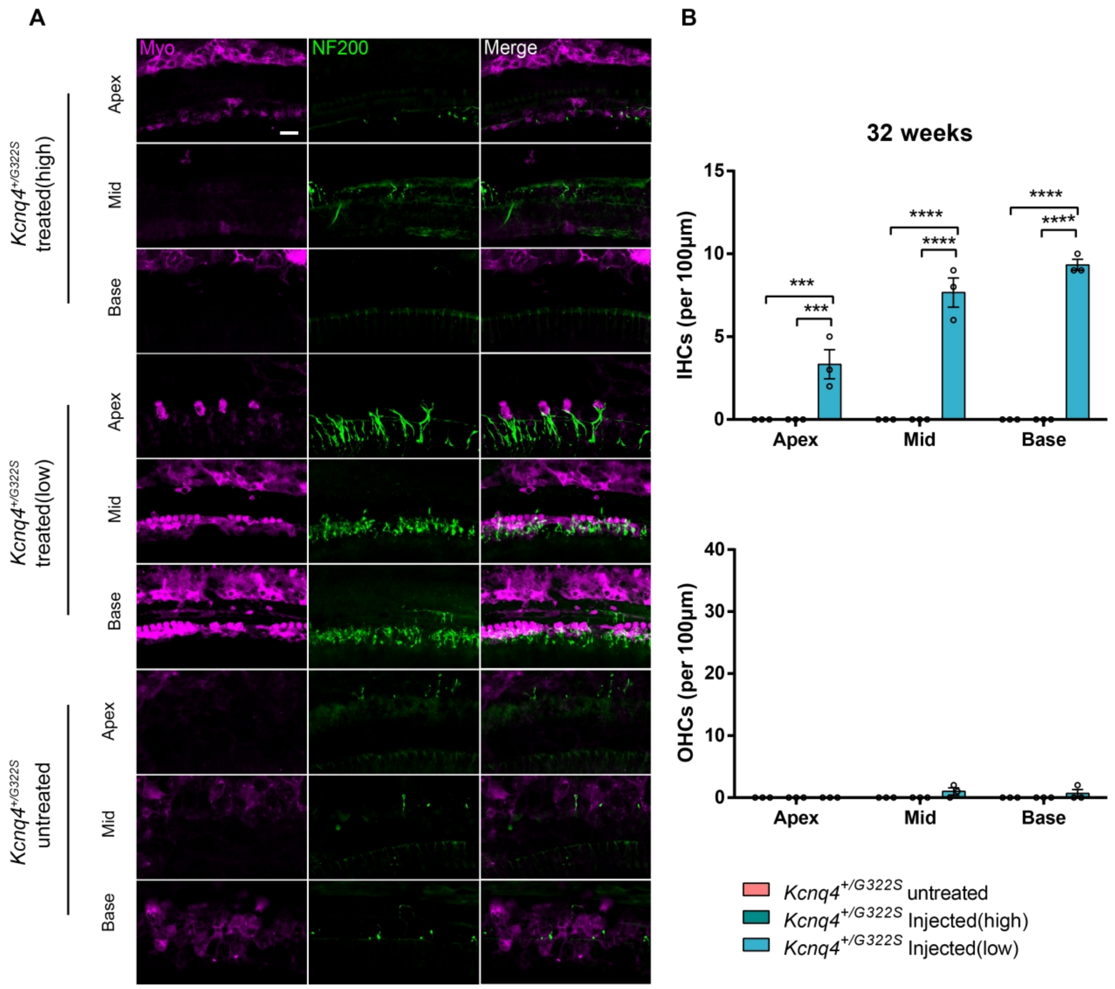

Appendix Figure S4. Morphological degeneration at later endpoint.

(A) Immunofluoresence images of the organ of corti in high-, low-dose treated *Kcnq4*$^{+/G322S}$ mice and untreated controls at 32 weeks of age(the magenta represents HCs and the green represents efferent and afferent neural fibers). Scale bar, 20 μ m. (B) Quantification of HCs in two regimens treated *Kcnq4*$^{+/G322S}$ mice and untreated controls at 32 weeks of age (n = 3 per group). Data are presented as mean ± SEM. Statistical analysis was performed using two-way ANOVA with Bonferroni ' s post hoc test. *p < 0.05; **p < 0.01; ***p < 0.001; ****p < 0.0001; ns: not significant.

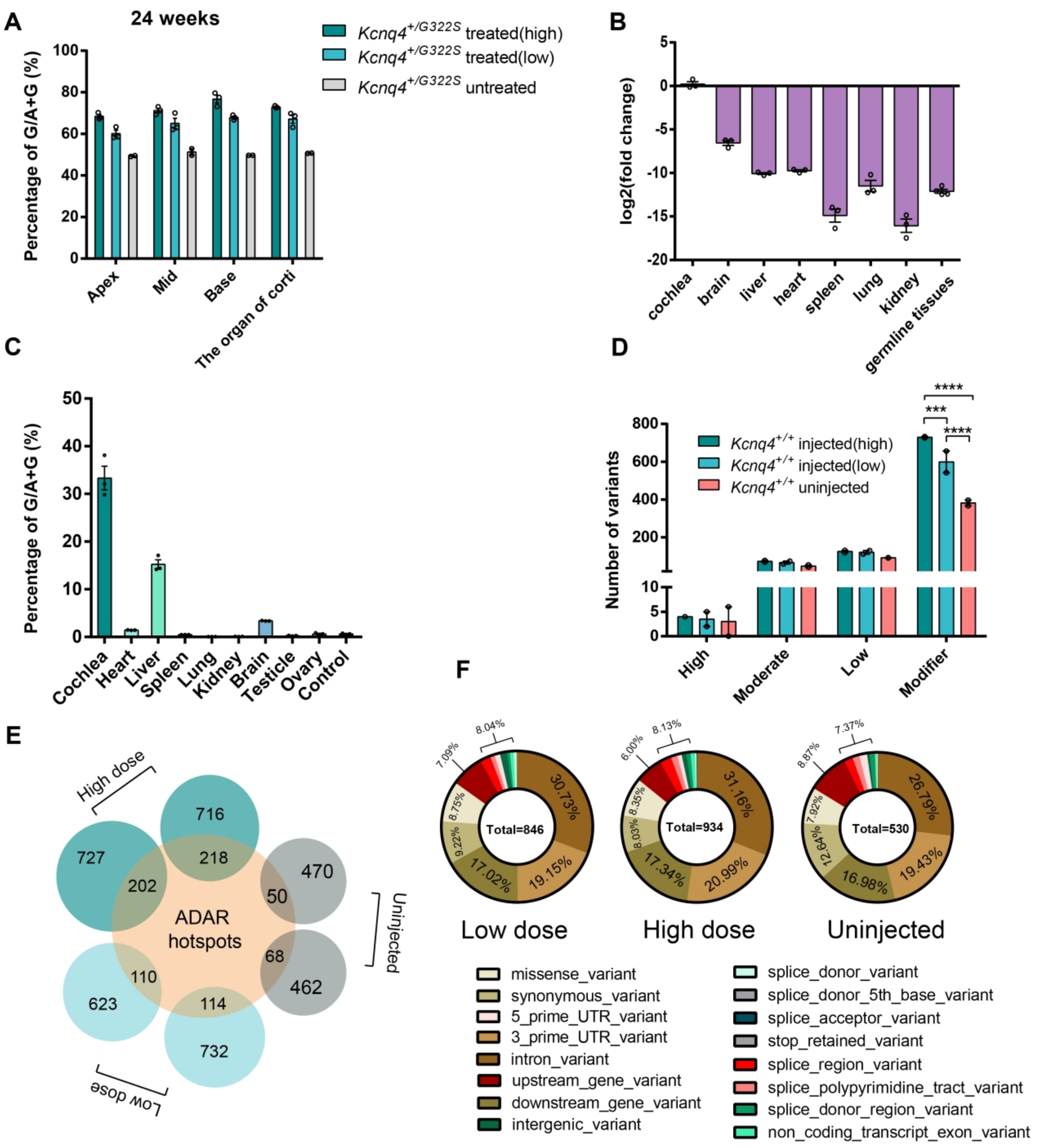

Appendix Figure S5. Editing efficiency, AAV distribution and advanced analysis of RNA off-target effects.

(A) The percentage of G/A+G across apical, middle and basal turns, as well as in the whole organ of Corti at DNA level in high-dose treated, low-dose treated (n = 3 per group) and untreated *Kncq4*$^{+/G322S}$ mice at 24 weeks of age (n = 2). (B) RT-qPCR quantification of ABE8e expression across major organs in high-dose treated *Kcnq4*$^{G322S/G322S}$ mice 3 weeks post injection, normalized to cochlear levels (ovary, n = 2; testicle, n = 2; other organs, n = 3). (C) A to G

editing efficiency across major organs in high-dose treated *Kcnq4*$^{G322S/G322S}$ mice 3 weeks post injection (n = 3 per group). (D) Analysis of A-to-I editing impact in high-dose, low-dose and uninjected *Kcnq4*$^{+/+}$ mice. (E) Overlap between A-to-I editing sites and ADAR hotspots across the high-dose, low-dose, and uninjected *Kcnq4*$^{+/+}$ mice. (F) Distribution of A-to-I editing sites across functions and regions in high-dose, low-dose and uninjected *Kcnq4*$^{+/+}$ mice. Data are presented as mean ± SEM. Statistical analysis was performed using two-way ANOVA with Bonferroni's post hoc test. *p < 0.05; **p < 0.01; ***p < 0.001; ****p < 0.0001; ns: not significant.

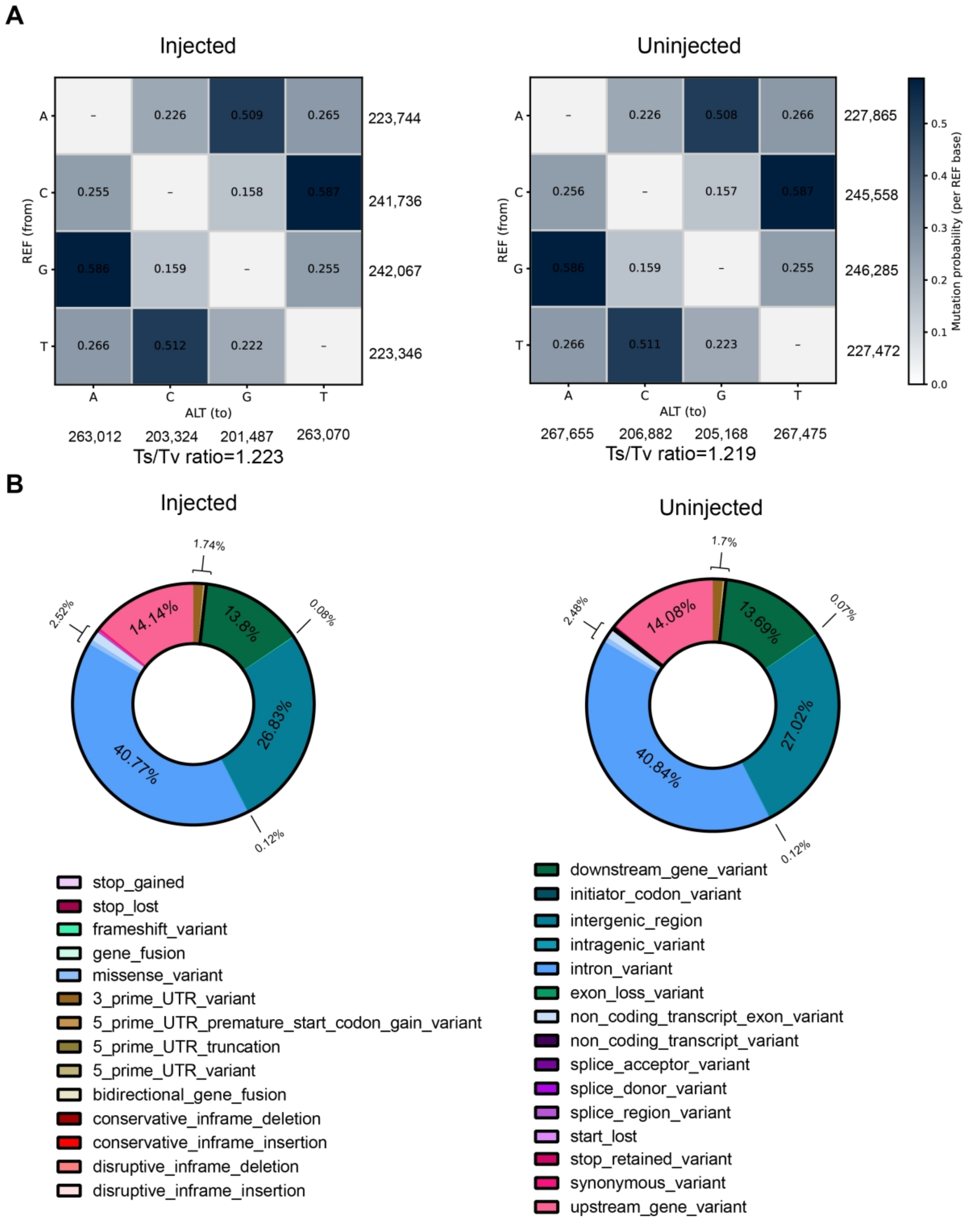

Appendix Figure S6. DNA off-target effects evaluated by WGS.

(A) Base substitution matrix of SNPs between high-dose and uninjected *Kcnq4*$^{+/+}$ mice, Ts and Tv indicates transitions and transversions, respectively. (B) Distribution of SNPs and indels across functions and regions in high-dose, low-dose and uninjected *Kcnq4*$^{+/+}$ mice.

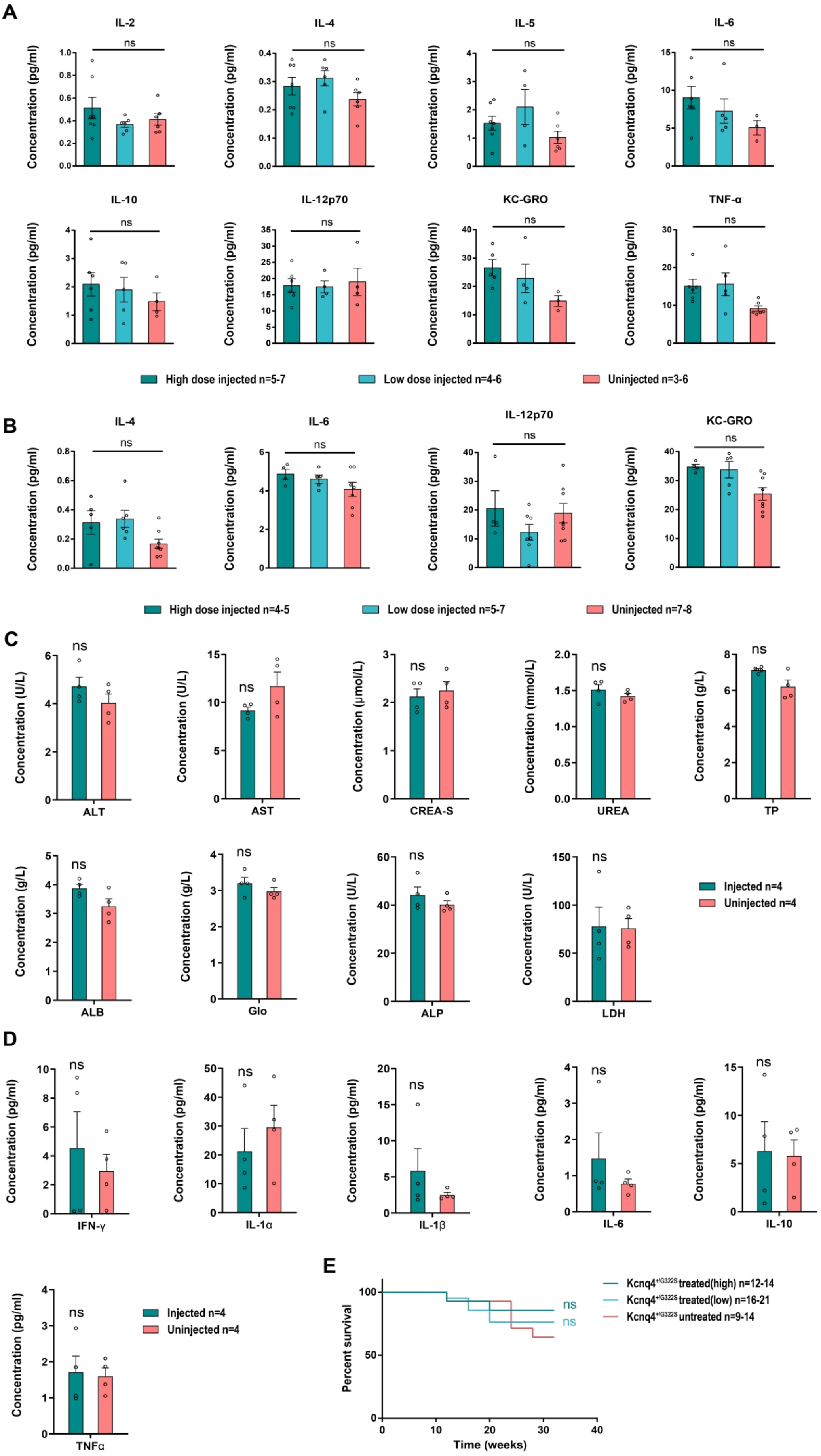
A
IL-2
IL-4
IL-5
IL-6
IL-10
IL-12p70
KC-GRO
TNF-α
ns
Concentration (pg/ml)
High dose injected n=5-7
Low dose injected n=4-6
Uninjected n=3-6
B
IL-4
IL-6
IL-12p70
KC-GRO
High dose injected n=4-5
Low dose injected n=5-7
Uninjected n=7-8
C
ALT
AST
CREA-S
UREA
TP
ALB
Glo
ALP
LDH
Concentration (U/L)
Concentration (μmol/L)
Concentration (mmol/L)
Concentration (g/L)
Injected n=4
Uninjected n=4
D
IFN-γ
IL-1α
IL-1β
IL-6
IL-10
TNFα
Injected n=4
Uninjected n=4
E
Percent survival
Time (weeks)
Kcnq4+/G322S treated(high) n=12-14
Kcnq4+/G322S treated(low) n=16-21
Kcnq4+/G322S untreated n=9-14

Appendix Figure S7. Safety assessment following AAV_ABE8e_N+C_322 administration.

(A, B) Cochlear inflammatory cytokines/chemokine (not significant) were detected in *Kcnq4*$^{+/+}$ mice at P10 (A) or 3 weeks (B) post AAV_ABE8e_N+C_322 injection (high dose: 3.64 × 10^{10} gc/mouse, P10, KC-GRO *n* = 5, IL-2,4,5 *n* = 7, others *n* = 6, 3w, IL-4 *n* = 5, others *n* = 4; low dose: 1.3 × 10^{10} gc/mouse, P10, IL-2,4 *n* = 6, IL-5,12p70, KC-GRO *n* = 4, others *n* = 5, 3w, IL-4 *n* = 6, IL-12p70 *n* = 7, others *n* = 5; uninjected, P10, IL-6, KC-GRO *n* = 3, IL-10,12p70 *n* = 4, others *n* = 6, 3w, IL-6 *n* = 7, others *n* = 8). Data are presented as mean ± SEM. Statistical analysis was performed using one-way ANOVA with Bonferroni's post hoc test. (C, D) Serum biochemical (C) and immunological (D) assessments in *Kcnq4*$^{+/+}$ mice 4 weeks after AAV_ABE8e_N+C_322 injection (3.64 × 10^{10} gc/mouse) compared to uninjected controls (*n* = 4). Data are presented as mean ± SEM. Statistical analysis was performed using unpaired t-tests. (E) Kaplan–Meier survival curves analysis of *Kcnq4*$^{+/G322S}$ mice receiving high-dose, low-dose, or no treatment over 32 weeks. Survival analysis was performed using the log-rank (Mantel–Cox) test. ns: not significant.

Appendix Table S1. Exact *P* values

1B (4w ABR)

Frequency (kHz)	*Kcnq4*$^{+/G322S}$ vs *Kcnq4*$^{+/+}$	*Kcnq4*$^{G322S/G322S}$ vs *Kcnq4*$^{+/+}$
4	<0.0001	<0.0001
5.656	<0.0001	<0.0001
8	<0.0001	<0.0001
11.314	<0.0001	<0.0001
16	<0.0001	<0.0001
22.627	<0.0001	<0.0001
32	<0.0001	<0.0001

1C (8w ABR)

Frequency (kHz)	*Kcnq4*$^{+/G322S}$ vs *Kcnq4*$^{+/+}$	*Kcnq4*$^{G322S/G322S}$ vs *Kcnq4*$^{+/+}$
4	<0.0001	<0.0001
5.656	<0.0001	<0.0001
8	<0.0001	<0.0001
11.314	<0.0001	<0.0001
16	<0.0001	<0.0001
22.627	<0.0001	<0.0001
32	<0.0001	<0.0001

1D (12w ABR)

Frequency (kHz)	*Kcnq4*$^{+/G322S}$ vs *Kcnq4*$^{+/+}$	*Kcnq4*$^{G322S/G322S}$ vs *Kcnq4*$^{+/+}$
4	<0.0001	<0.0001
5.656	<0.0001	<0.0001
8	<0.0001	<0.0001
11.314	<0.0001	<0.0001
16	<0.0001	<0.0001
22.627	<0.0001	<0.0001
32	<0.0001	<0.0001

1E (16w ABR)

Frequency (kHz)	*Kcnq4*$^{+/G322S}$ vs *Kcnq4*$^{+/+}$	*Kcnq4*$^{G322S/G322S}$ vs *Kcnq4*$^{+/+}$
4	<0.0001	<0.0001
5.656	<0.0001	<0.0001
8	<0.0001	<0.0001
11.314	<0.0001	<0.0001

16	<0.0001	<0.0001
22.627	<0.0001	<0.0001
32	<0.0001	<0.0001

1F (4w DPOAE)

**Frequency (kHz)**	$Kcnq4^{+/G322S}$ **vs** $Kcnq4^{+/+}$	$Kcnq4^{G322S/G322S}$ **vs** $Kcnq4^{+/+}$
4	<0.0001	<0.0001
5.656	<0.0001	<0.0001
8	<0.0001	<0.0001
11.314	<0.0001	<0.0001
16	<0.0001	<0.0001
22.627	<0.0001	<0.0001
32	0.0082	<0.0001

1G (8w DPOAE)

**Frequency (kHz)**	$Kcnq4^{+/G322S}$ **vs** $Kcnq4^{+/+}$	$Kcnq4^{G322S/G322S}$ **vs** $Kcnq4^{+/+}$
4	<0.0001	<0.0001
5.656	<0.0001	<0.0001
8	<0.0001	<0.0001
11.314	<0.0001	<0.0001
16	<0.0001	<0.0001
22.627	<0.0001	<0.0001
32	0.0047	0.0002

1H (12w DPOAE)

**Frequency (kHz)**	$Kcnq4^{+/G322S}$ **vs** $Kcnq4^{+/+}$	$Kcnq4^{G322S/G322S}$ **vs** $Kcnq4^{+/+}$
4	<0.0001	<0.0001
5.656	<0.0001	<0.0001
8	<0.0001	<0.0001
11.314	<0.0001	<0.0001
16	<0.0001	<0.0001
22.627	<0.0001	<0.0001
32	0.0006	0.0001

1I (16w DPOAE)

Frequency (kHz)	*Kcnq4*$^{+/G322S}$ vs *Kcnq4*$^{+/+}$	*Kcnq4*$^{G322S/G322S}$ vs *Kcnq4*$^{+/+}$
4	<0.0001	<0.0001
5.656	<0.0001	<0.0001
8	<0.0001	<0.0001
11.314	<0.0001	<0.0001
16	<0.0001	<0.0001
22.627	<0.0001	<0.0001
32	0.0027	0.0002

1L (IHCs)

Turns	*Kcnq4*$^{+/G322S}$vs *Kcnq4*$^{+/+}$	*Kcnq4*$^{G322S/G322S}$vs *Kcnq4*$^{+/+}$	*Kcnq4*$^{+/G322S}$vs *Kcnq4*$^{G322S/G322S}$
Apex	0.7814	0.4162	>0.9999
Mid	0.4162	0.4162	>0.9999
Base	>0.9999	>0.9999	>0.9999

1M (OHCs)

Turns	*Kcnq4*$^{+/G322S}$vs *Kcnq4*$^{+/+}$	*Kcnq4*$^{G322S/G322S}$vs *Kcnq4*$^{+/+}$	*Kcnq4*$^{+/G322S}$vs *Kcnq4*$^{G322S/G322S}$
Apex	<0.0001	<0.0001	0.5250
Mid	<0.0001	<0.0001	<0.0001
Base	0.5250	<0.0001	<0.0001

2D (Editing efficiency with sgRNA1)

Sites	ABE8.20-m vs control	ABE8e vs control	NG-ABE8e vs control	CP1028-ABE 8e vs control	ABEmax vs control	ABEmaxCP1041 vs control
A8	<0.0001	<0.0001	<0.0001	<0.0001	0.0028	0.0103
A20	>0.9999	>0.9999	>0.9999	>0.9999	>0.9999	>0.9999

2E (Editing efficiency with sgRNA2)

Sites	ABE8.20-m vs control	ABE8e vs control	NG-ABE8e vs control	CP1028-ABE 8e vs control	ABEmax vs control	ABEmaxCP1041 vs control
A2	<0.0001	<0.0001	<0.0001	<0.0001	>0.9999	>0.9999
A14	>0.9999	>0.9999	>0.9999	<0.0001	>0.9999	<0.0001
A15	>0.9999	>0.9999	>0.9999	<0.0001	>0.9999	<0.0001

2F (Editing efficiency with single- or dual-plasmid transfected)

Sites	Single vs control	Double vs control	Single vs double

A8	<0.0001	<0.0001	<0.0001
A20	>0.9999	>0.9999	>0.9999

3B (Hom editing efficiency on DNA level)

High dose vs uninjected	Low dose vs uninjected	High dose vs low dose
0.0013	0.0094	0.5732

3C (Hom editing efficiency on cDNA level)

High dose vs uninjected	Low dose vs uninjected	High dose vs low dose
<0.0001	<0.0001	<0.0001

4A (4w ABR after treatment)

Frequency (kHz)	High dose vs untreated	Low dose vs untreated
4	0.0009	>0.9999
5.656	0.0006	0.9130
8	0.0002	>0.9999
11.314	<0.0001	0.5803
16	0.0004	0.5454
22.627	0.0006	>0.9999
32	0.0279	>0.9999

4B (8w ABR after treatment)

Frequency (kHz)	High dose vs untreated	Low dose vs untreated
4	0.0046	0.0070
5.656	<0.0001	0.0004
8	<0.0001	<0.0001
11.314	<0.0001	0.0001
16	<0.0001	<0.0001
22.627	<0.0001	<0.0001
32	<0.0001	<0.0001

4C (20w ABR after treatment)

Frequency (kHz)	High dose vs untreated	Low dose vs untreated
4	0.8555	0.0003
5.656	0.0744	<0.0001
8	0.0420	<0.0001
11.314	0.0213	<0.0001
16	0.0207	<0.0001
22.627	0.0002	<0.0001
32	<0.0001	<0.0001

4D (32w ABR after treatment)

Frequency (kHz)	High dose vs untreated	Low dose vs untreated
4	>0.9999	0.2445
5.656	0.9719	0.0021
8	>0.9999	0.0006
11.314	0.8869	0.0001
16	0.8064	<0.0001
22.627	0.8064	<0.0001
32	0.5925	<0.0001

4E (4w DPOAE after treatment)

Frequency (kHz)	High dose vs untreated	Low dose vs untreated
4	0.1202	>0.9999
5.656	0.1202	0.1906
8	0.0004	0.2326
11.314	0.1202	>0.9999
16	0.0065	>0.9999
22.627	0.2137	>0.9999
32	0.8387	>0.9999

4F (8w DPOAE after treatment)

Frequency (kHz)	High dose vs untreated	Low dose vs untreated
4	>0.9999	0.0516
5.656	0.2998	0.0516
8	0.0083	0.0213
11.314	0.0004	0.0043

16	0.0026	0.0979
22.627	0.0143	0.0213
32	<0.0001	<0.0001

4G (20w DPOAE after treatment)

Frequency (kHz)	High dose vs untreated	Low dose vs untreated
4	>0.9999	>0.9999
5.656	>0.9999	0.0240
8	0.4380	0.0004
11.314	0.0911	<0.0001
16	0.0498	<0.0001
22.627	0.0273	<0.0001
32	0.2251	<0.0001

4H (32w DPOAE after treatment)

Frequency (kHz)	High dose vs untreated	Low dose vs untreated
4	>0.9999	>0.9999
5.656	>0.9999	0.9381
8	>0.9999	0.0611
11.314	>0.9999	0.0328
16	>0.9999	<0.0001
22.627	>0.9999	0.0007
32	>0.9999	0.0017

4J (ABR threshold at different time points)

Time points (weeks)	High dose vs low dose
4	0.0201
8	0.3635
12	0.5192
16	0.1316
20	0.0009
24	<0.0001
28	<0.0001
32	0.0068
36	0.5201

4K (Wave I amplitude at 8 weeks)

Frequency (kHz)	High dose vs untreated	Low dose vs untreated	High dose vs low dose
4	0.0955	<0.0001	0.2958

5.656	0.0138	<0.0001	0.5346
8	0.0063	<0.0001	0.0826
11.314	0.0014	<0.0001	0.2470
16	0.0011	<0.0001	0.3448
22.627	0.0057	<0.0001	>0.9999
32	0.0002	0.0008	0.9189

4L (Wave I latency at 8 weeks)

Frequency (kHz)	High dose vs untreated	Low dose vs untreated	High dose vs low dose
4	0.1391	0.0012	0.8118
5.656	0.0021	<0.0001	0.4885
8	<0.0001	<0.0001	>0.9999
11.314	<0.0001	<0.0001	>0.9999
16	0.0003	0.0003	>0.9999
22.627	0.0004	<0.0001	>0.9999
32	0.0017	0.0001	>0.9999

5B (IHCs)

Turns	High dose vs untreated	Low dose vs untreated	High dose vs low dose
Apex	0.0116	0.0532	>0.9999
Mid	0.0297	0.1112	>0.9999
Base	0.1073	0.2078	>0.9999

5C (OHCs)

Turns	High dose vs untreated	Low dose vs untreated	High dose vs low dose
Apex	<0.0001	0.0044	>0.9999
Mid	<0.0001	<0.0001	>0.9999
Base	<0.0001	<0.0001	>0.9999

5E (SGNs)

Turns	High dose vs low dose	High dose vs untreated	High dose vs wt	Low dose vs untreated	Low dose vs wt	Untreated vs wt
Apex	>0.9999	<0.0001	>0.9999	<0.0001	>0.9999	<0.0001
Mid	>0.9999	0.0424	>0.9999	0.0493	>0.9999	0.0303
Base	>0.9999	0.0292	>0.9999	0.0201	>0.9999	0.0248

6C (I/V curves)

Voltage (mV)	Wt vs untreated	High dose vs untreated	Low dose vs untreated
-140	>0.9999	0.8793	>0.9999
-130	>0.9999	0.9384	>0.9999
-120	>0.9999	>0.9999	>0.9999
-110	>0.9999	>0.9999	>0.9999
-100	>0.9999	>0.9999	>0.9999
-90	>0.9999	>0.9999	>0.9999
-80	>0.9999	>0.9999	>0.9999
-70	>0.9999	>0.9999	>0.9999
-60	0.0978	0.8300	>0.9999
-50	0.0003	0.1688	>0.9999
-40	<0.0001	0.0466	>0.9999
-30	<0.0001	0.0109	>0.9999
-20	<0.0001	0.0070	>0.9999
-10	<0.0001	0.0163	>0.9999
0	<0.0001	0.0747	>0.9999
10	<0.0001	0.3283	>0.9999
20	<0.0001	>0.9999	>0.9999
30	<0.0001	>0.9999	>0.9999

6D (I max at -140 mV)

High dose vs low dose	High dose vs untreated	High dose vs wt	Low dose vs untreated	Low dose vs wt	Untreated vs wt
>0.9999	<0.0001	0.0097	0.0084	0.0012	<0.0001

6E (Resting potential)

High dose vs low dose	High dose vs untreated	High dose vs wt	Low dose vs untreated	Low dose vs wt	Untreated vs wt
>0.9999	0.0061	0.0019	0.0630	<0.0001	<0.0001

7A (Cochlear inflammatory cytokines at P10)

Cytokines	High dose vs untreated	Low dose vs untreated	High dose vs low dose
IFN- γ	0.0010	>0.9999	0.0068
IL-1 β	0.0055	0.0322	>0.9999

7B (Cochlear inflammatory cytokines at 3weeks)

Cytokines	High dose vs	Low dose vs untreated	High dose vs low dose

	untreated		
IFN- γ	0.0019	0.0045	>0.9999
IL-1 β	0.0001	0.0003	>0.9999
IL-2	0.0057	0.0185	>0.9999
IL-5	0.0431	0.4543	0.6968
IL-10	0.0004	0.0690	0.0598
TNF- α	<0.0001	0.0952	0.0090

7C (ABE8e expression between high- and low-dose groups)

High dose vs low dose
0.0018

EV4A (Het editing efficiency on DNA level)

High dose vs uninjected	Low dose vs uninjected	High dose vs low dose
<0.0001	0.0004	<0.0001

EV4B (Het editing efficiency on cDNA level)

High dose vs uninjected	Low dose vs uninjected	High dose vs low dose
0.0366	>0.9999	0.1125

EV4I (RNA A-I editing efficiency)

High dose vs uninjected	Low dose vs uninjected	High dose vs low dose
0.0094	0.0320	0.1646

EV5M (Wave I latency at 4 weeks)

Frequency (kHz)	High dose vs untreated	Low dose vs untreated	High dose vs low dose
4	0.0423	>0.9999	0.0287
5.656	>0.9999	>0.9999	>0.9999
8	0.4387	>0.9999	0.2163
11.314	0.2437	>0.9999	0.0970
16	0.0203	>0.9999	0.0050
22.627	0.0442	0.0080	>0.9999
32	0.0185	0.0008	>0.9999
