# Supplementary figures and images for: Long-term restoration of auditory function in a DFNA2 mouse model by adenine base editing (part 1 of 2)
Source: EMBO Mol Med. 2026 May 20;18(6):2293–321. doi: 10.1038/s44321-026-00433-5 (PMC13270111; doi:10.1038/s44321-026-00433-5)

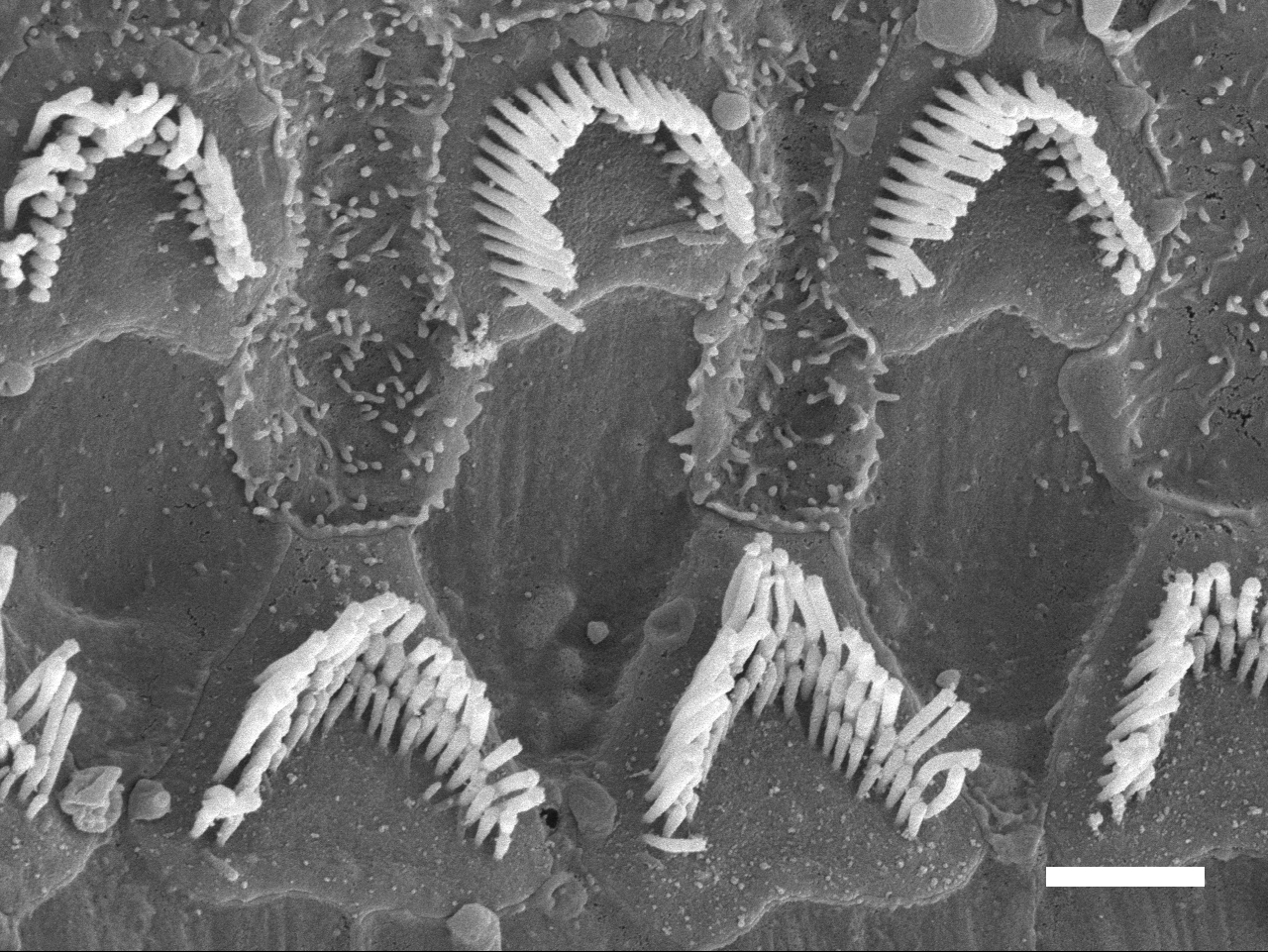

Supplement: Supplementary file 9 — Source data Fig. 1 [file 44321_2026_433_MOESM9_ESM.zip › Figure 1/1J/WT-APEX-high-bar.tif]

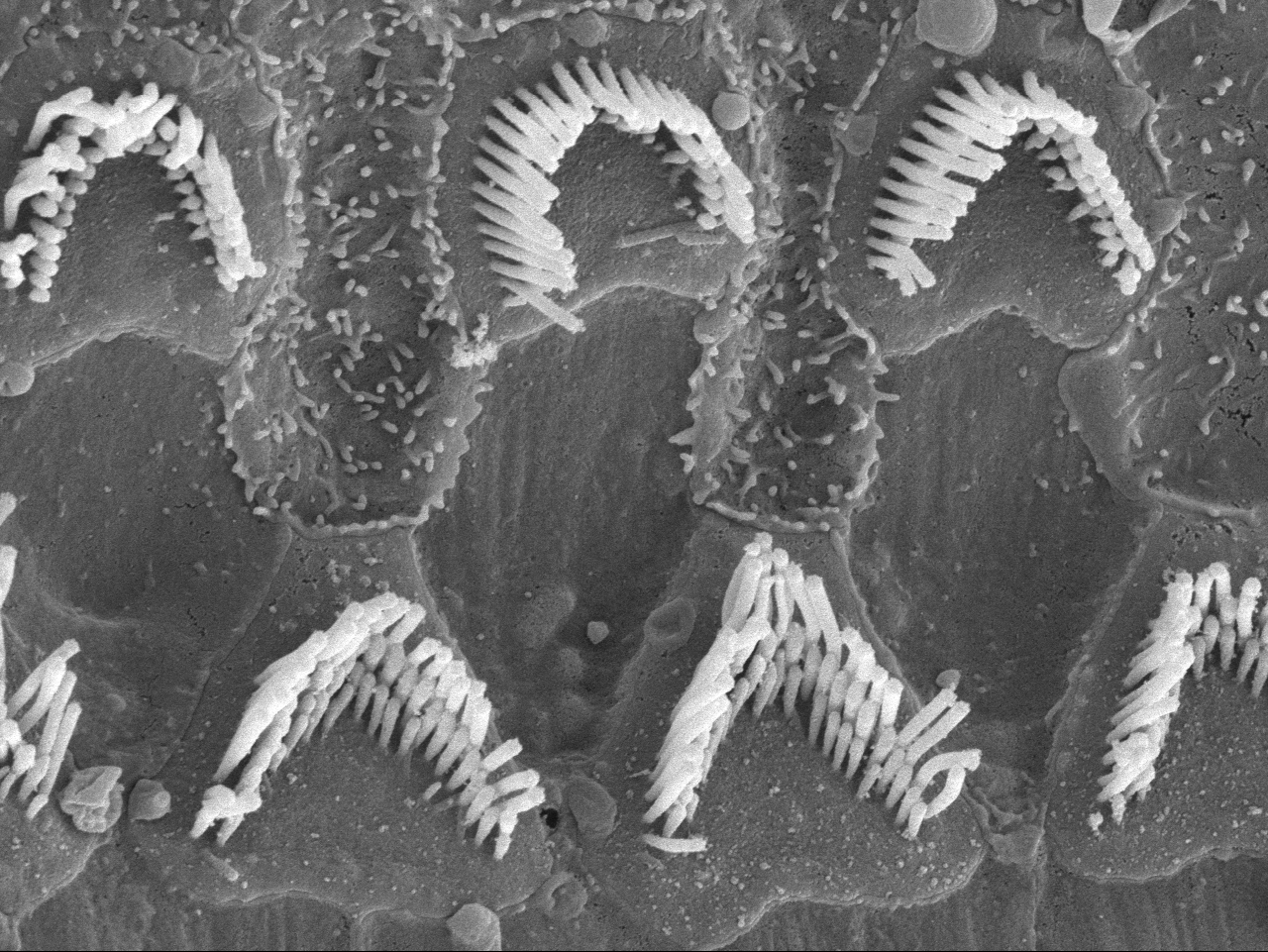

Supplement: Supplementary file 9 — Source data Fig. 1 [file 44321_2026_433_MOESM9_ESM.zip › Figure 1/1J/WT-APEX-high.tif]

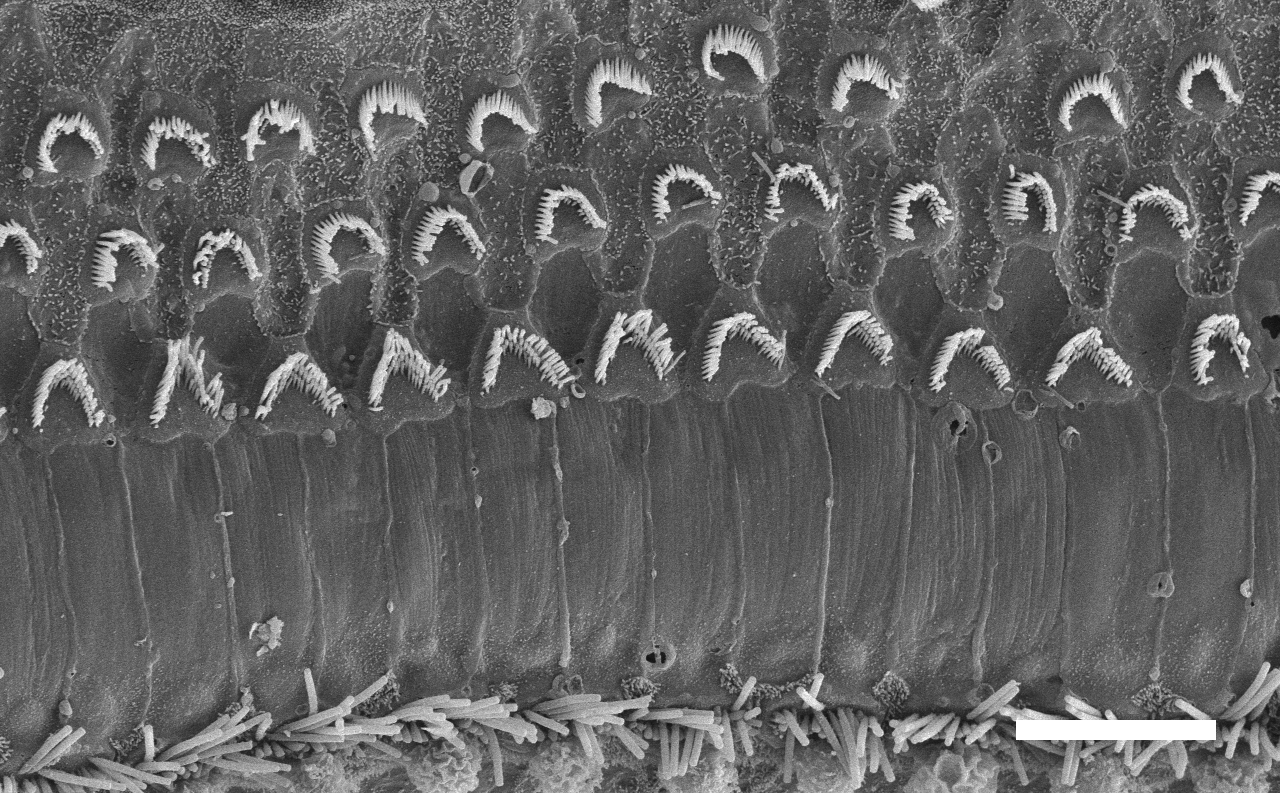

Supplement: Supplementary file 9 — Source data Fig. 1 [file 44321_2026_433_MOESM9_ESM.zip › Figure 1/1J/WT-APEX-low-bar.tif]

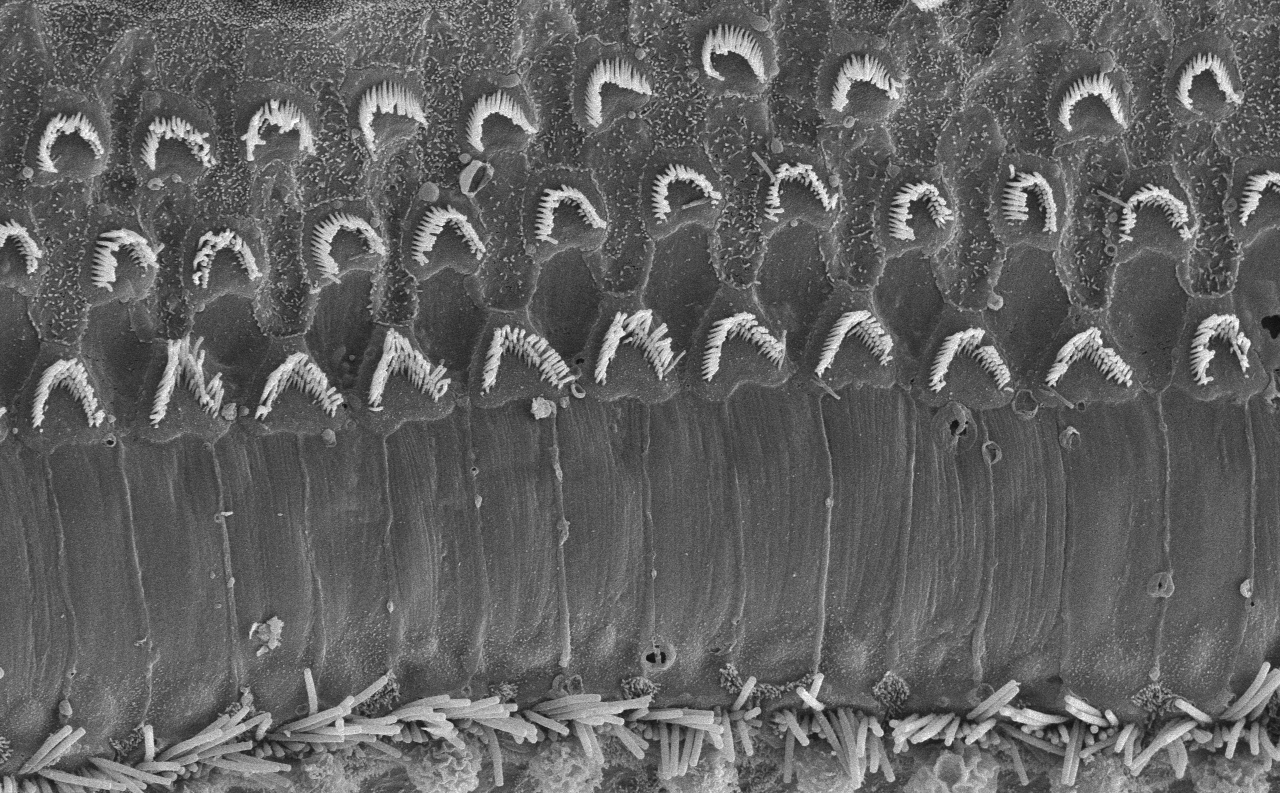

Supplement: Supplementary file 9 — Source data Fig. 1 [file 44321_2026_433_MOESM9_ESM.zip › Figure 1/1J/WT-APEX-low.tif]

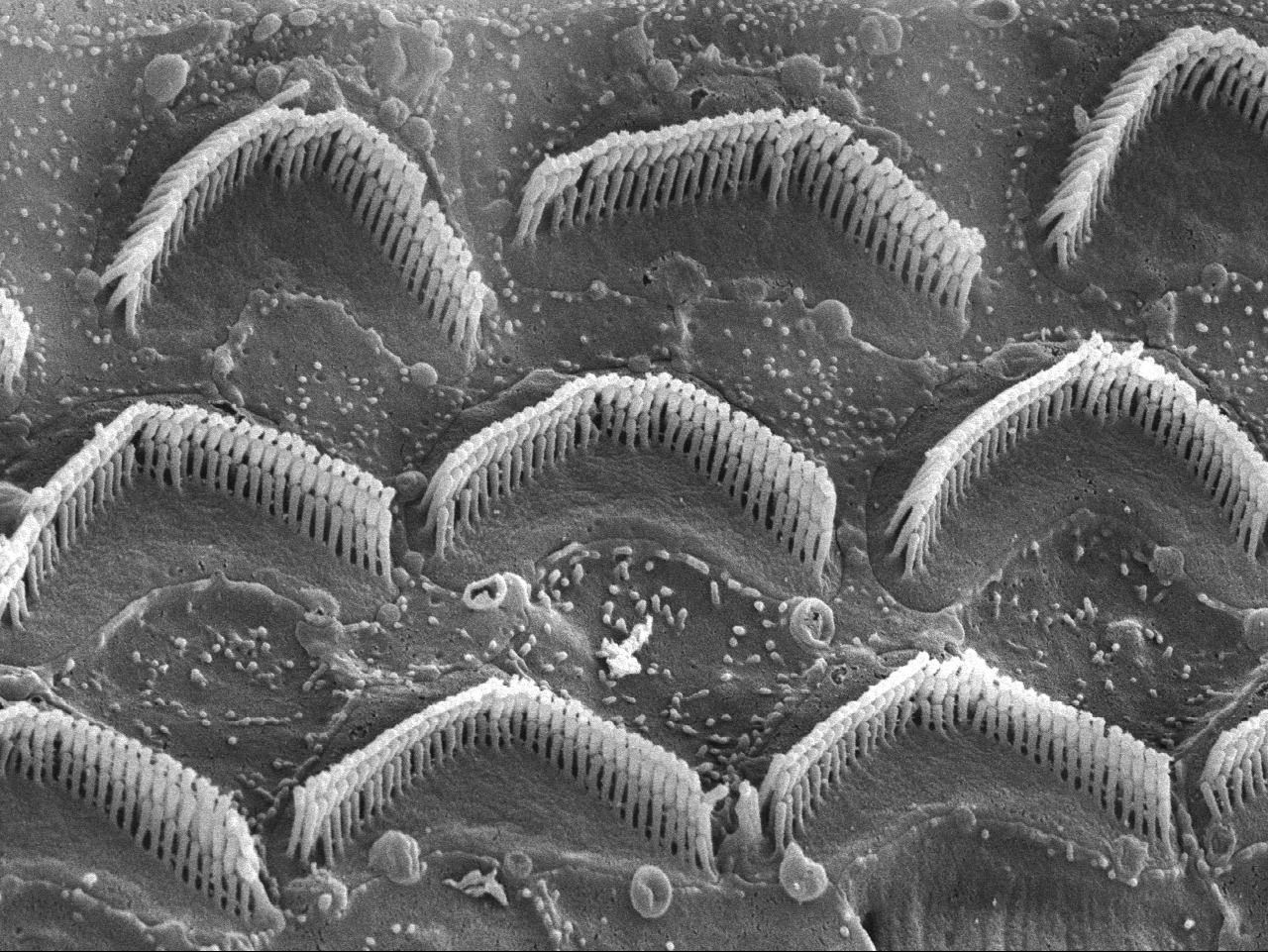

Supplement: Supplementary file 9 — Source data Fig. 1 [file 44321_2026_433_MOESM9_ESM.zip › Figure 1/1J/WT-BASE-high.tif]

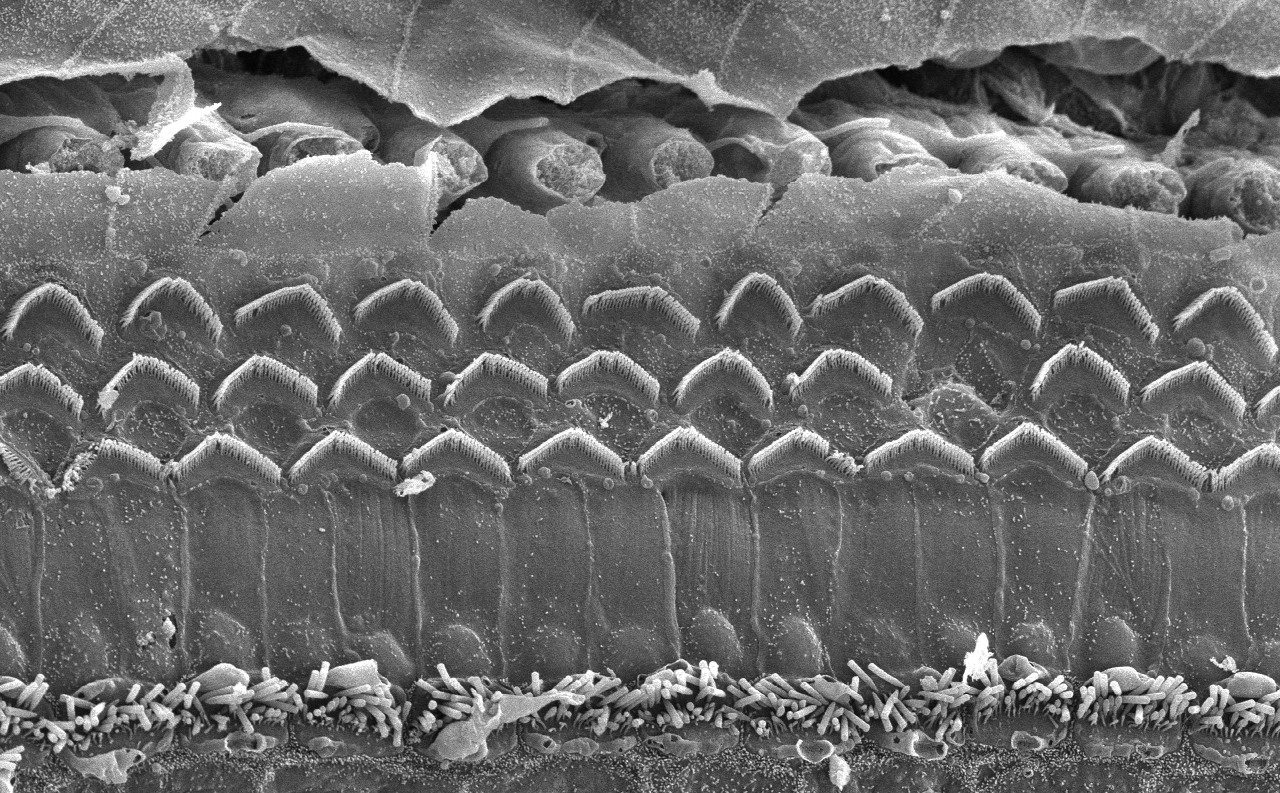

Supplement: Supplementary file 9 — Source data Fig. 1 [file 44321_2026_433_MOESM9_ESM.zip › Figure 1/1J/WT-BASE-low.tif]

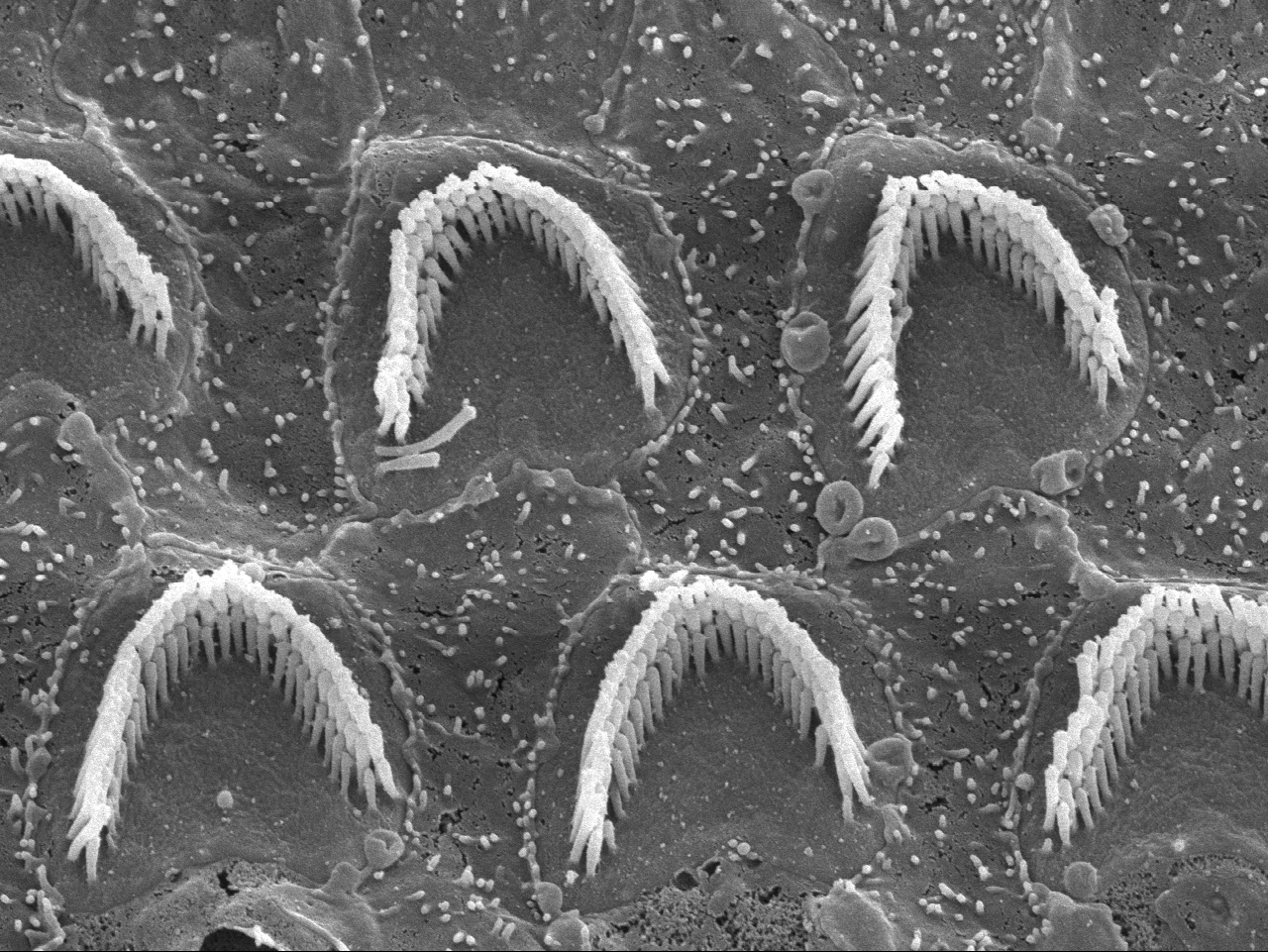

Supplement: Supplementary file 9 — Source data Fig. 1 [file 44321_2026_433_MOESM9_ESM.zip › Figure 1/1J/WT-MID-high.tif]

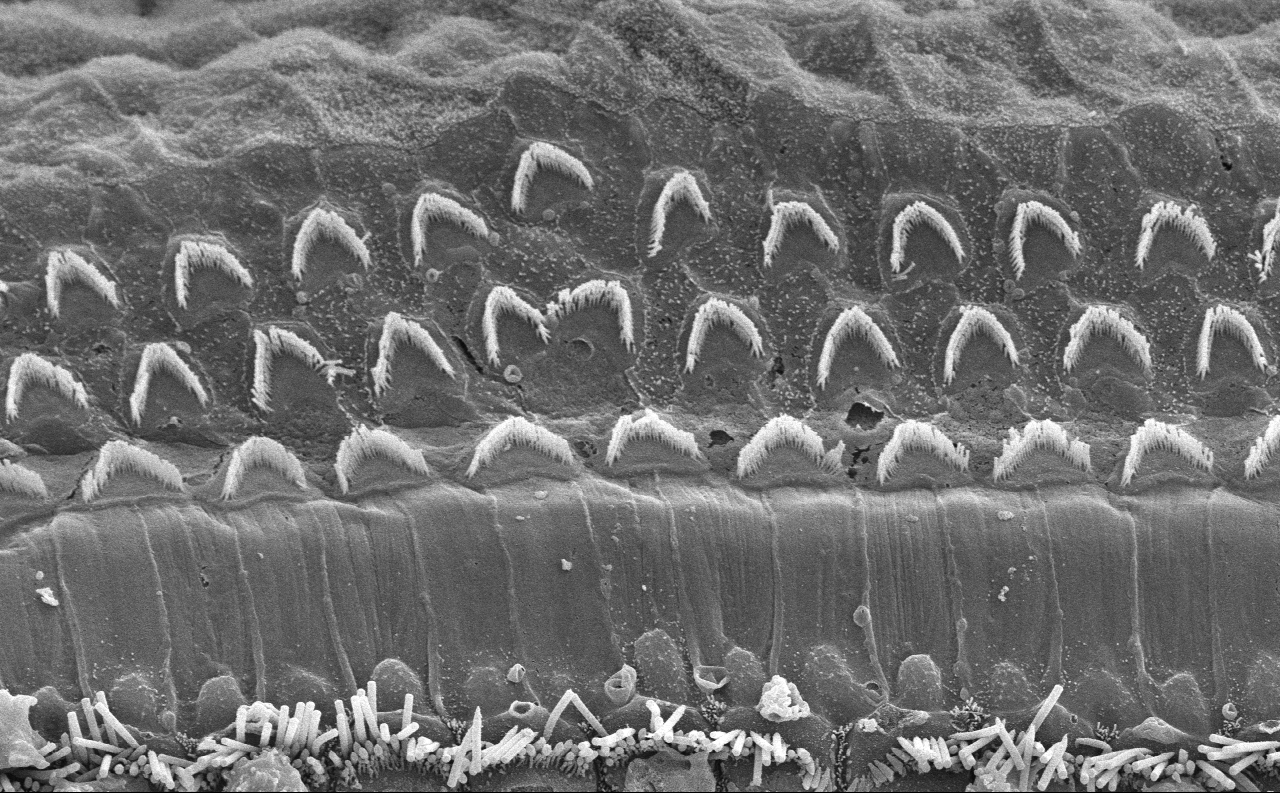

Supplement: Supplementary file 9 — Source data Fig. 1 [file 44321_2026_433_MOESM9_ESM.zip › Figure 1/1J/WT-MID-low.tif]

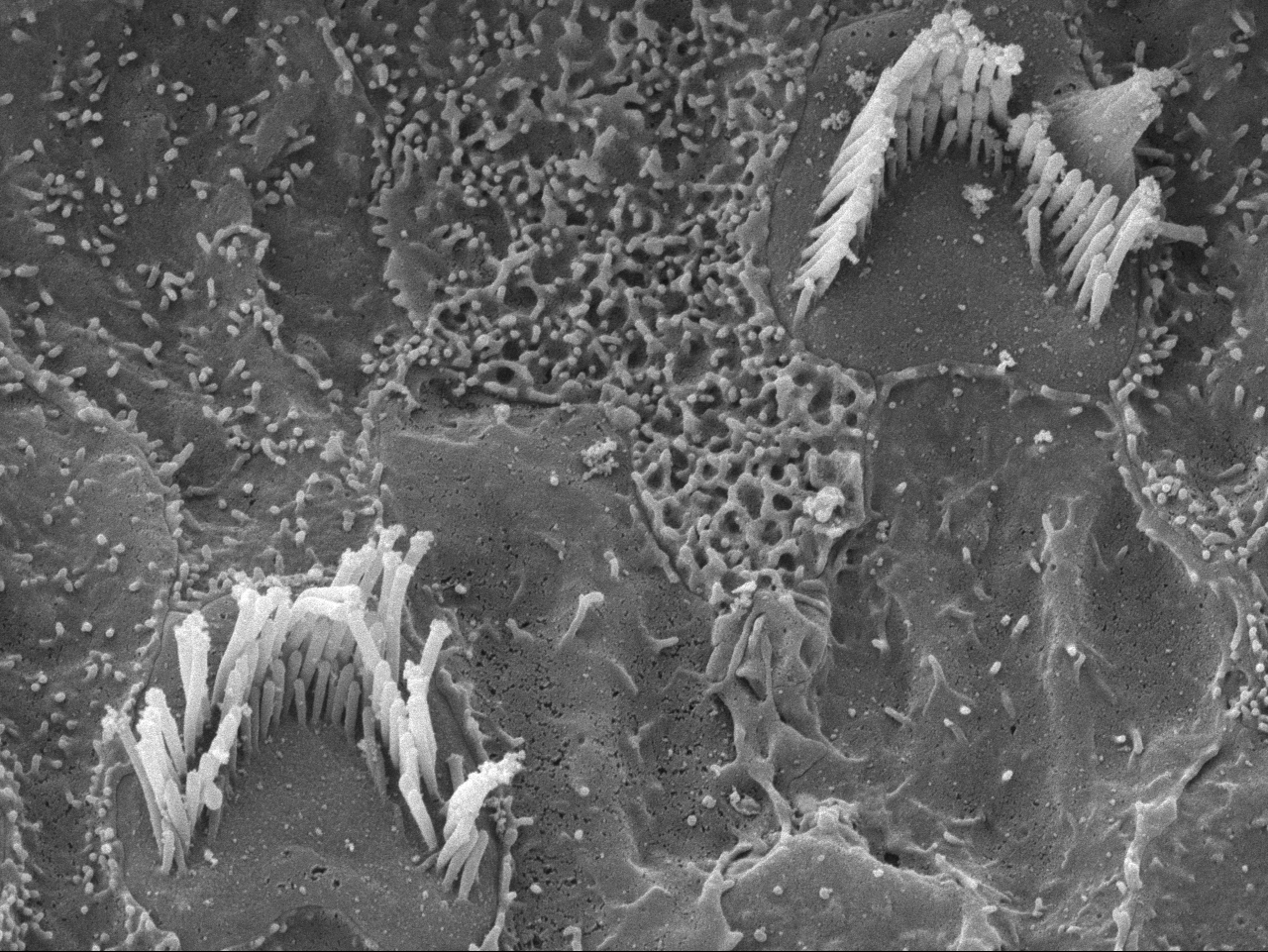

Supplement: Supplementary file 9 — Source data Fig. 1 [file 44321_2026_433_MOESM9_ESM.zip › Figure 1/1J/het-apex-high.tif]

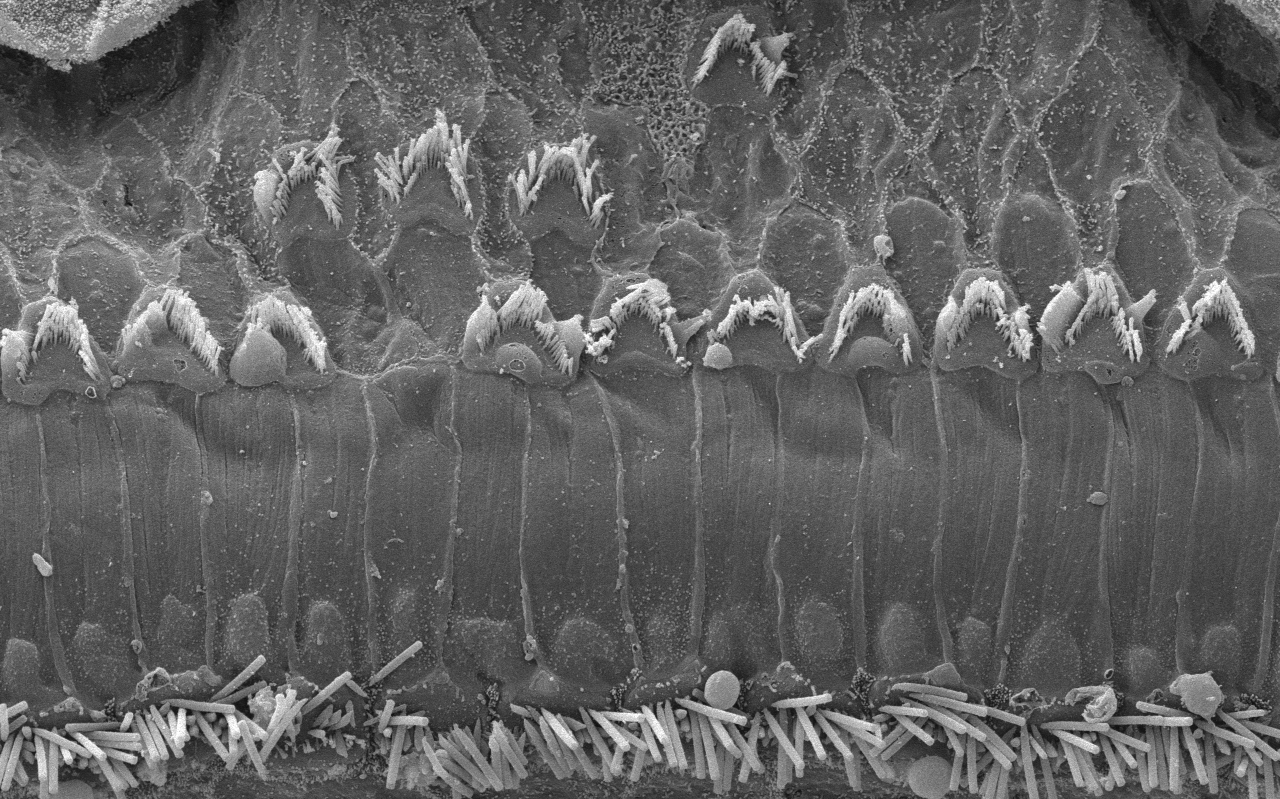

Supplement: Supplementary file 9 — Source data Fig. 1 [file 44321_2026_433_MOESM9_ESM.zip › Figure 1/1J/het-apex-low.tif]

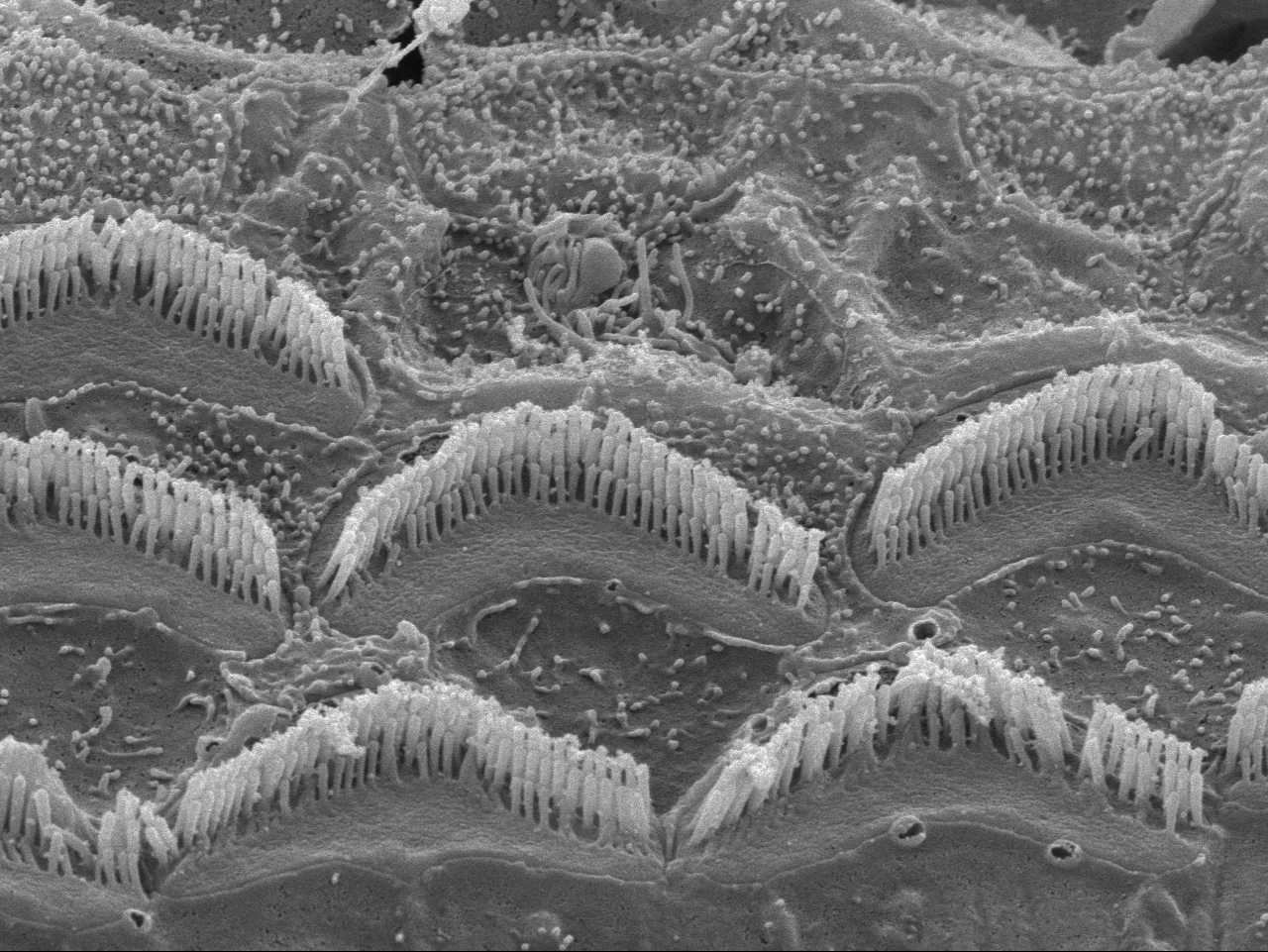

Supplement: Supplementary file 9 — Source data Fig. 1 [file 44321_2026_433_MOESM9_ESM.zip › Figure 1/1J/het-base-high.tif]

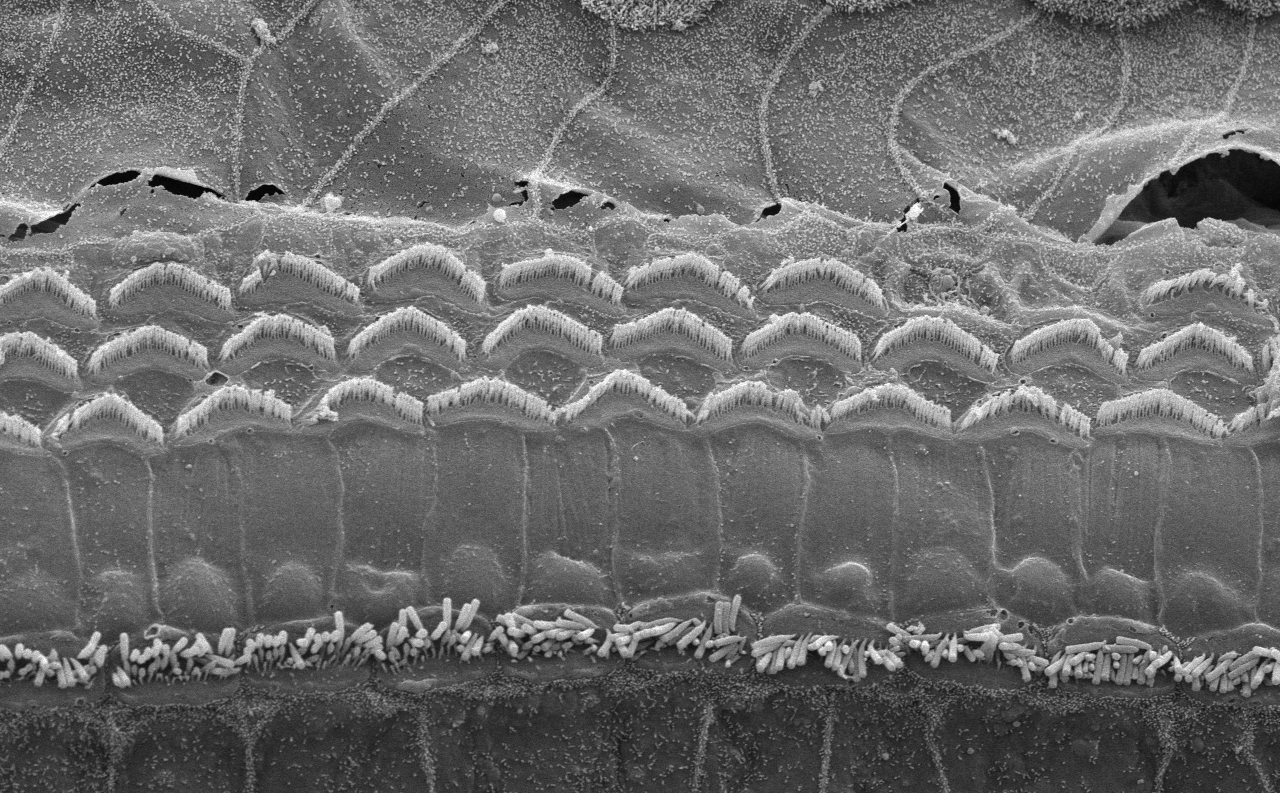

Supplement: Supplementary file 9 — Source data Fig. 1 [file 44321_2026_433_MOESM9_ESM.zip › Figure 1/1J/het-base-low.tif]

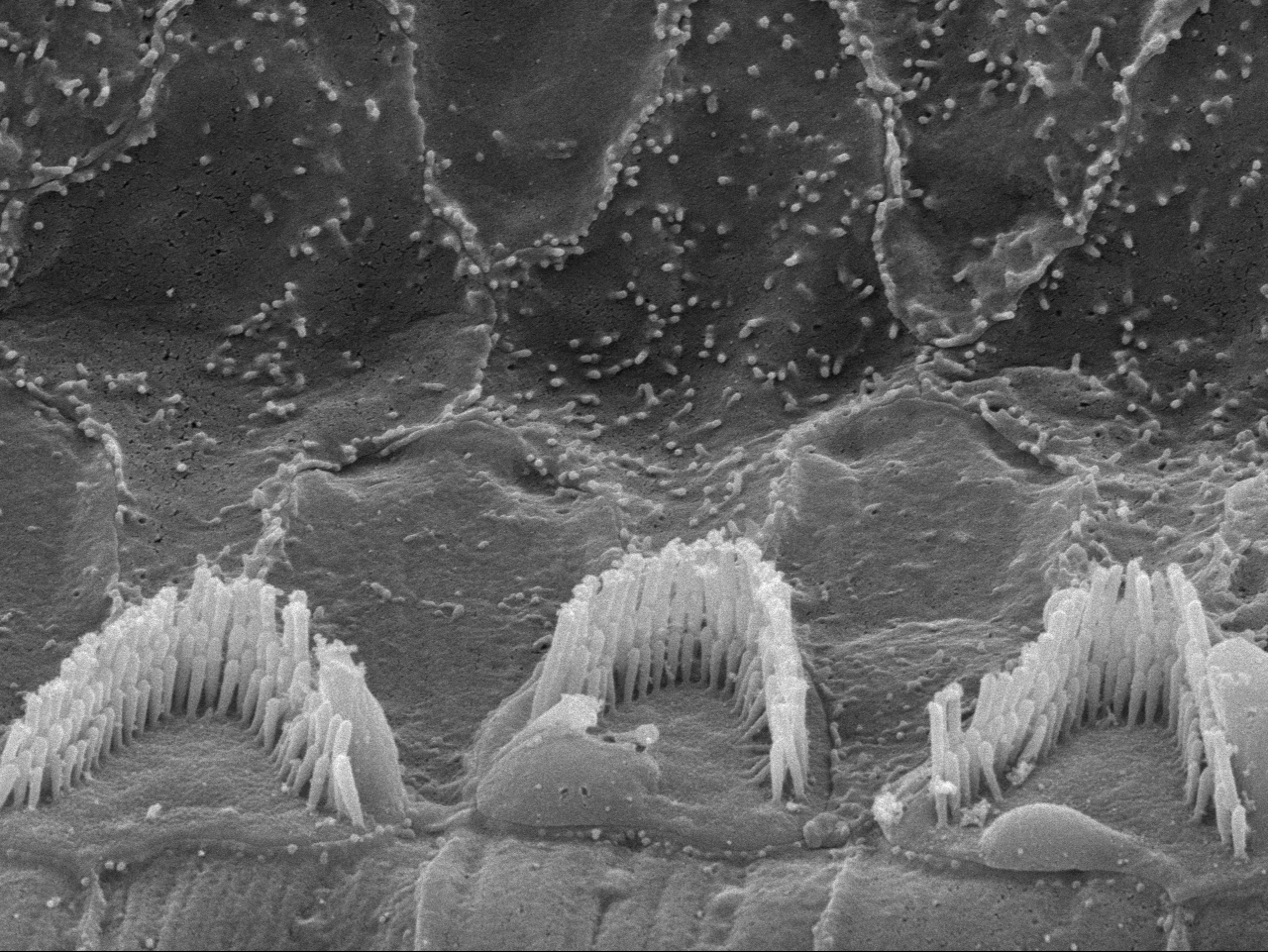

Supplement: Supplementary file 9 — Source data Fig. 1 [file 44321_2026_433_MOESM9_ESM.zip › Figure 1/1J/het-mid-high.tif]

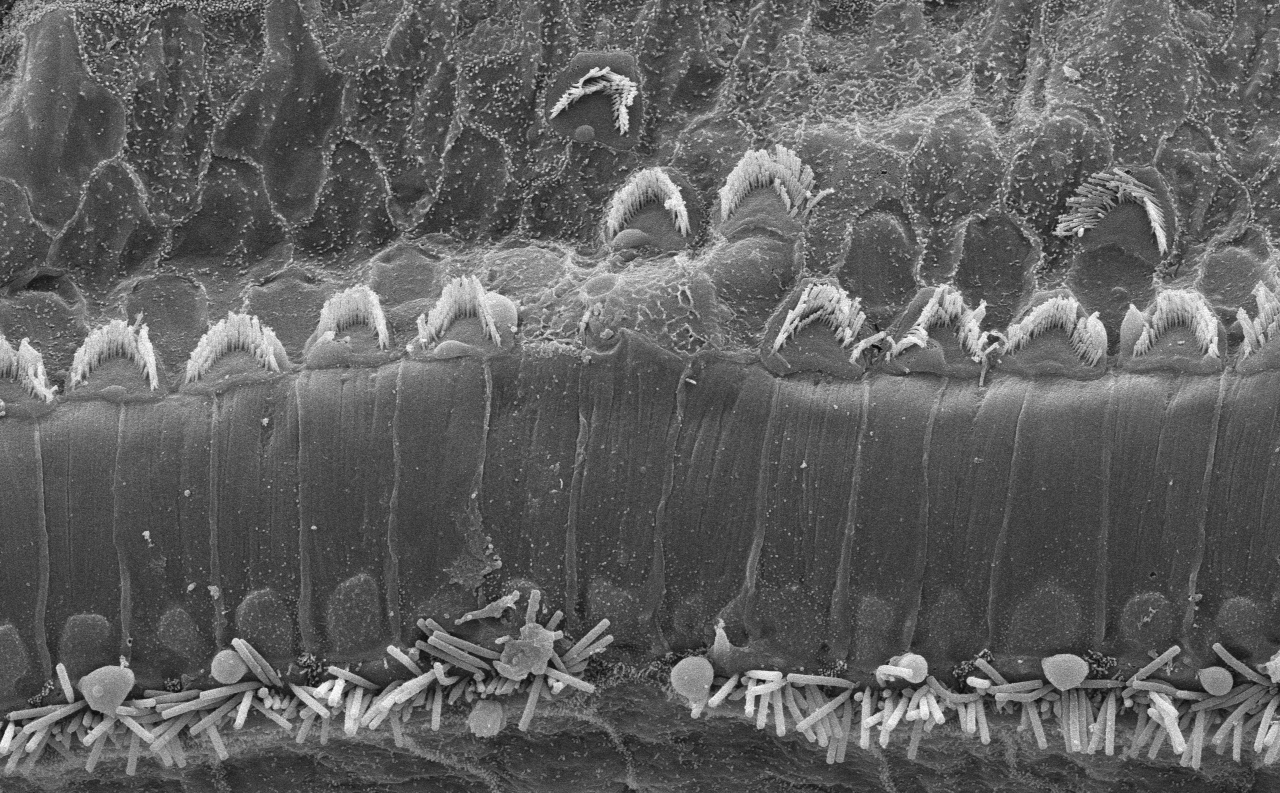

Supplement: Supplementary file 9 — Source data Fig. 1 [file 44321_2026_433_MOESM9_ESM.zip › Figure 1/1J/het-mid-low.tif]

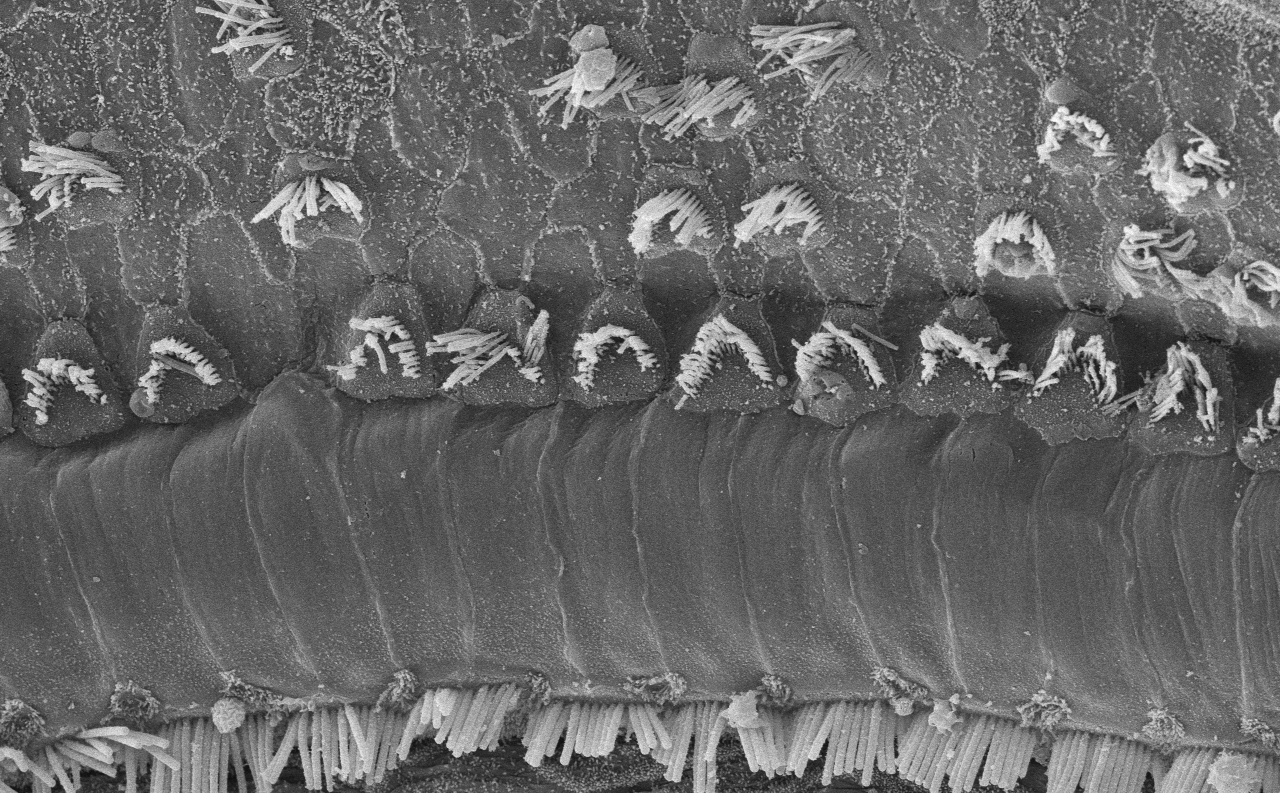

Supplement: Supplementary file 9 — Source data Fig. 1 [file 44321_2026_433_MOESM9_ESM.zip › Figure 1/1J/hom-aepx-low.tif]

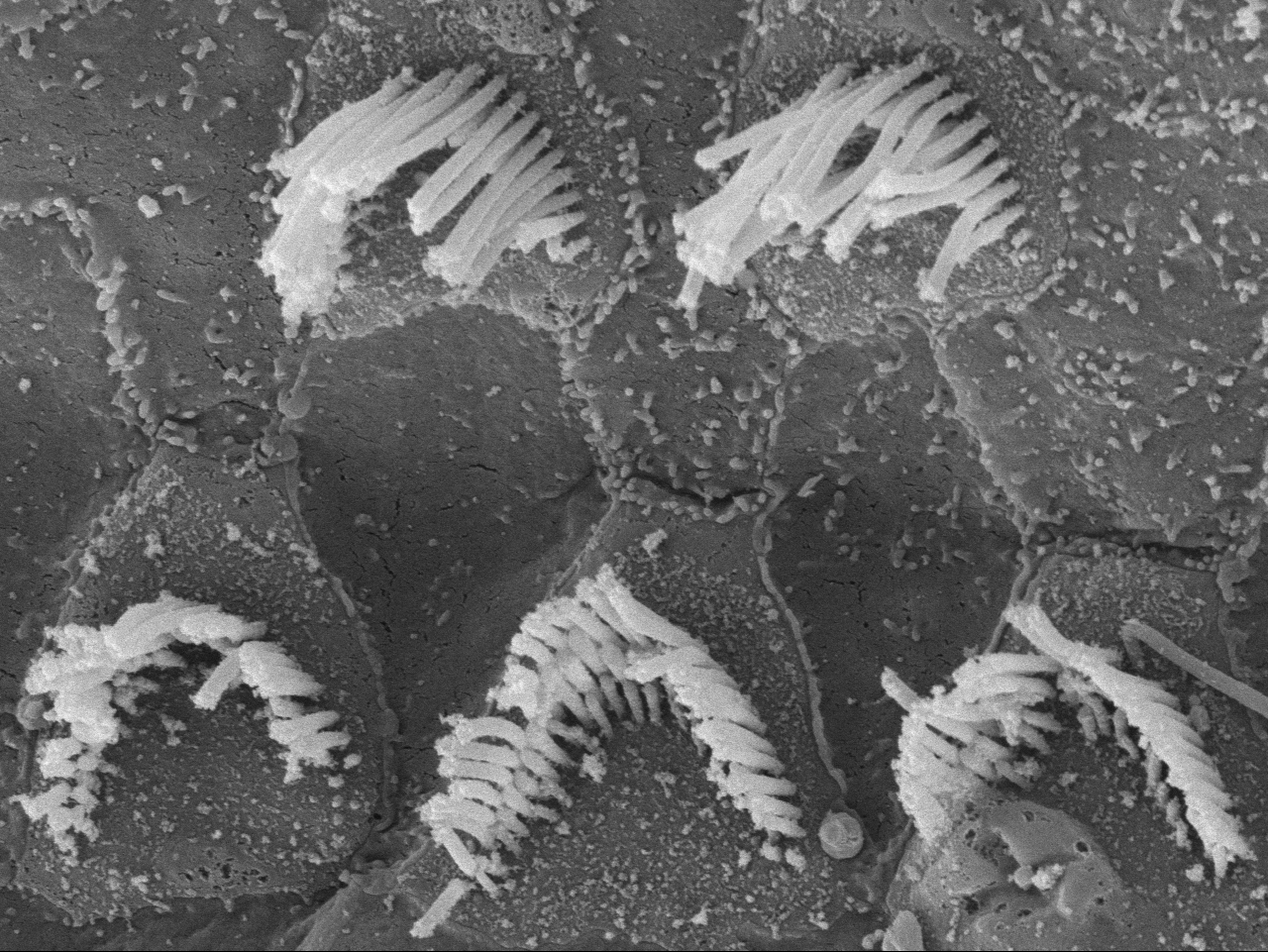

Supplement: Supplementary file 9 — Source data Fig. 1 [file 44321_2026_433_MOESM9_ESM.zip › Figure 1/1J/hom-apex-high.tif]

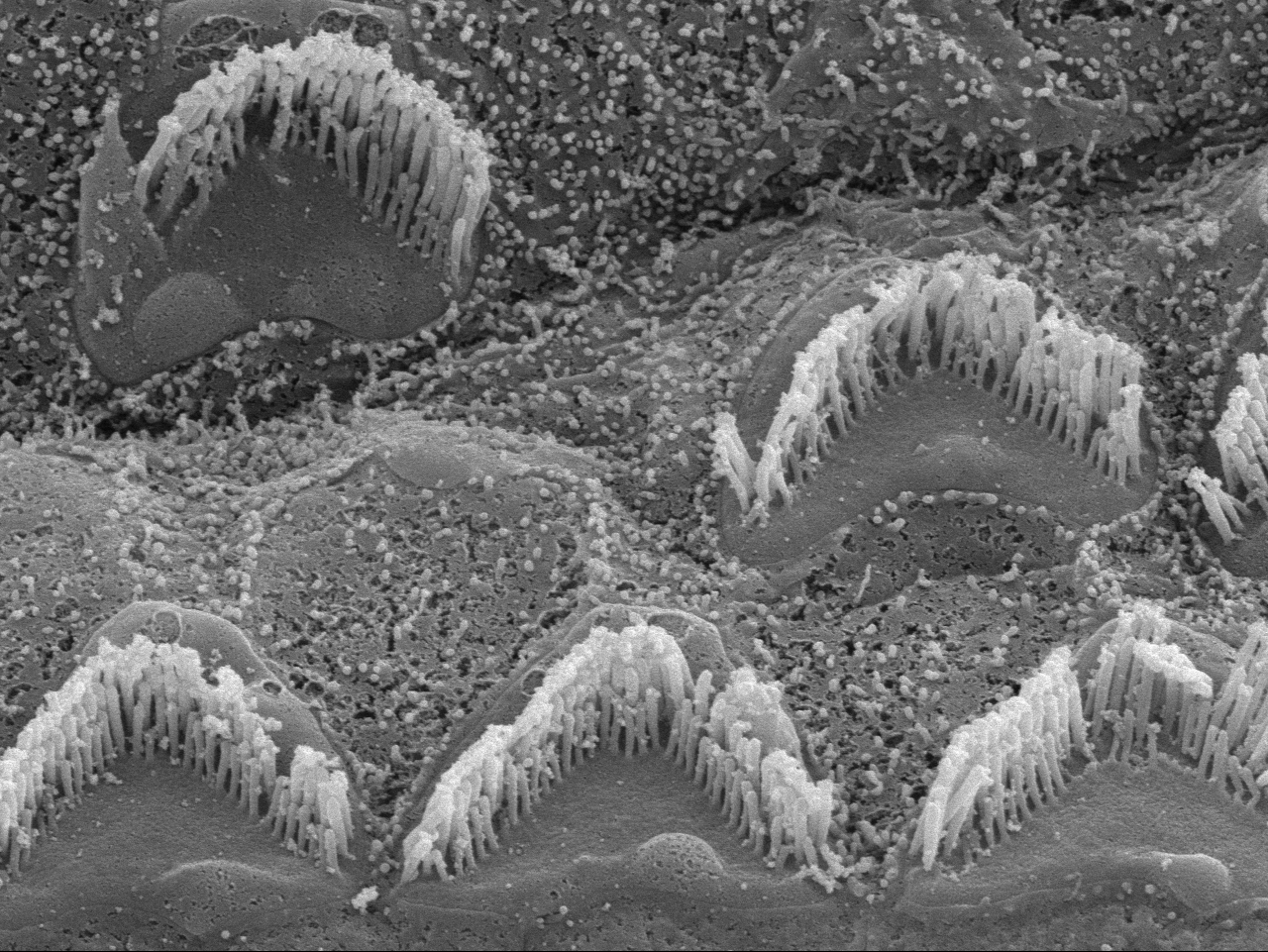

Supplement: Supplementary file 9 — Source data Fig. 1 [file 44321_2026_433_MOESM9_ESM.zip › Figure 1/1J/hom-base-high.tif]

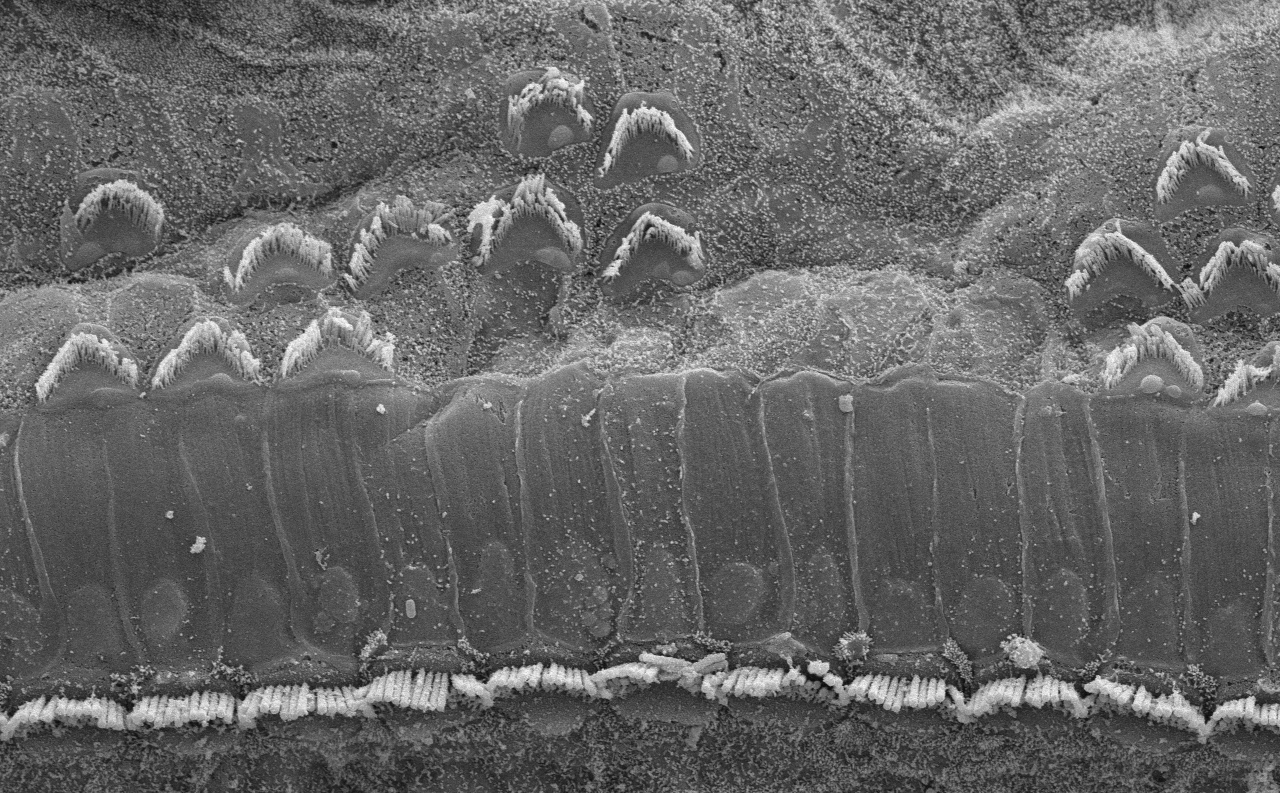

Supplement: Supplementary file 9 — Source data Fig. 1 [file 44321_2026_433_MOESM9_ESM.zip › Figure 1/1J/hom-base-low.tif]

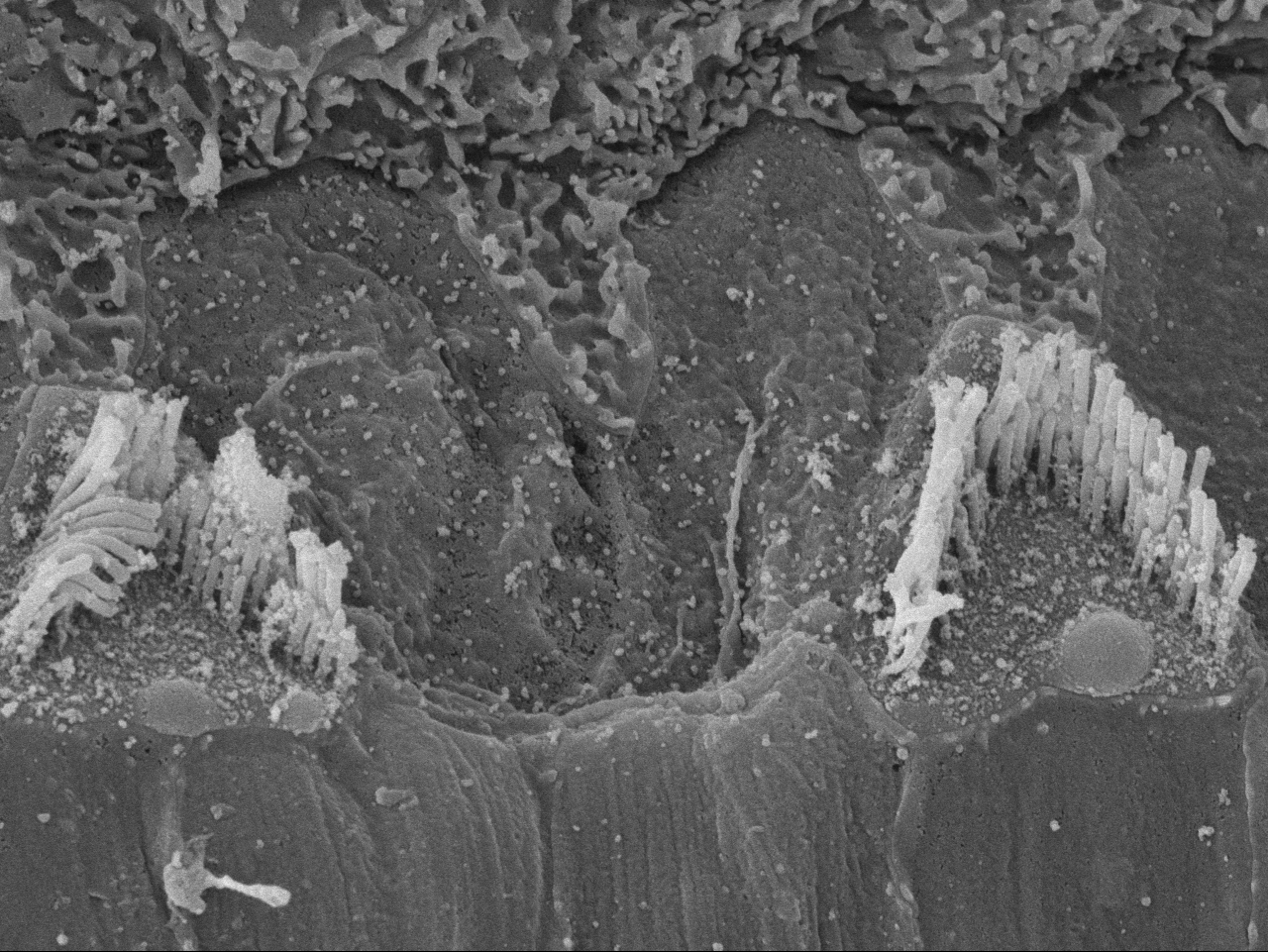

Supplement: Supplementary file 9 — Source data Fig. 1 [file 44321_2026_433_MOESM9_ESM.zip › Figure 1/1J/hom-mid-high.tif]

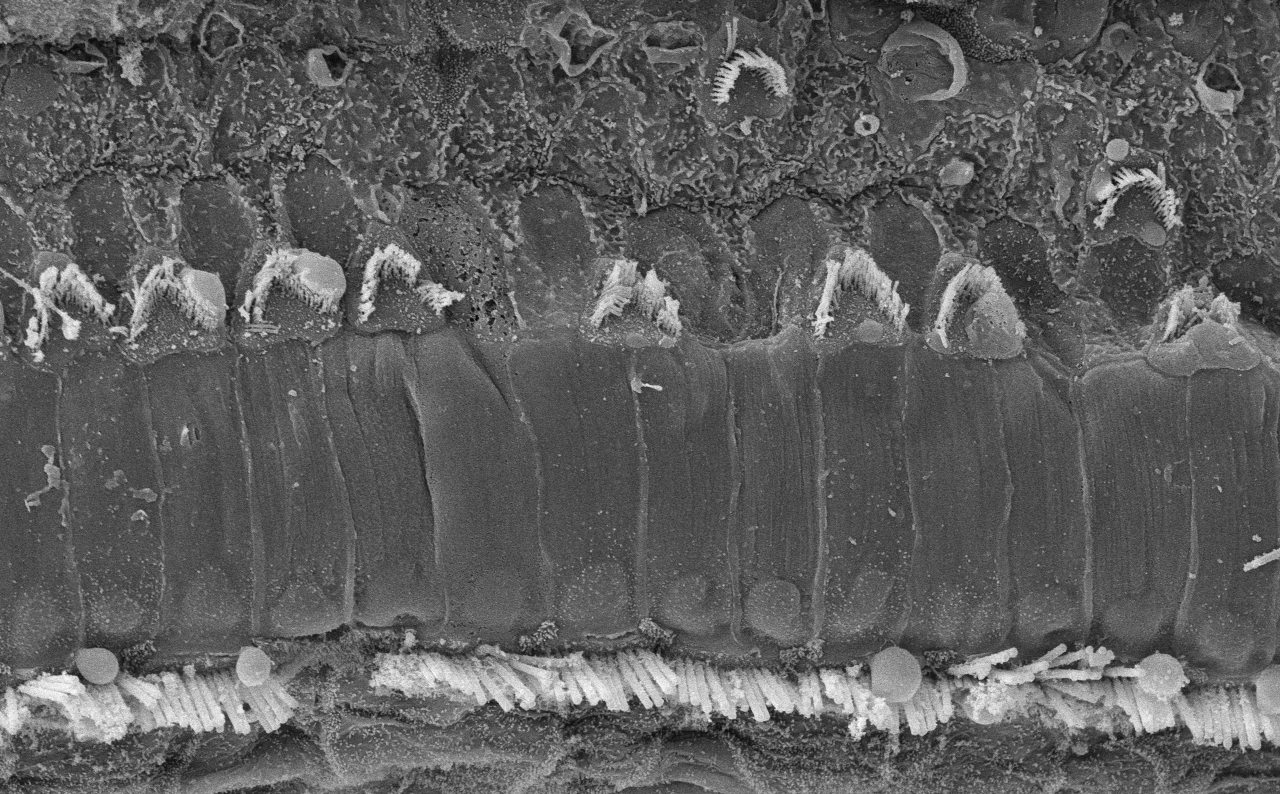

Supplement: Supplementary file 9 — Source data Fig. 1 [file 44321_2026_433_MOESM9_ESM.zip › Figure 1/1J/hom-mid-low.tif]

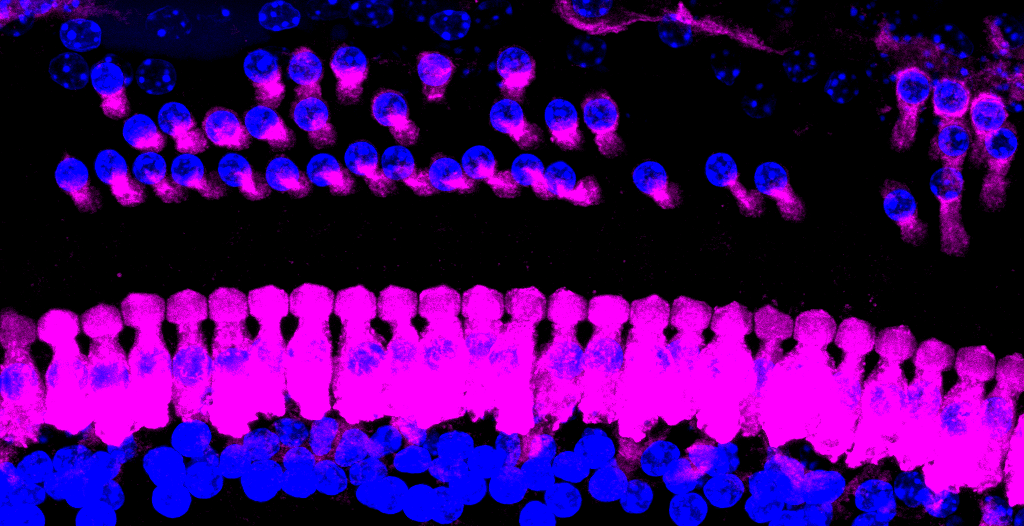

Supplement: Supplementary file 9 — Source data Fig. 1 [file 44321_2026_433_MOESM9_ESM.zip › Figure 1/1K/1m-het-mid-merge.tif]

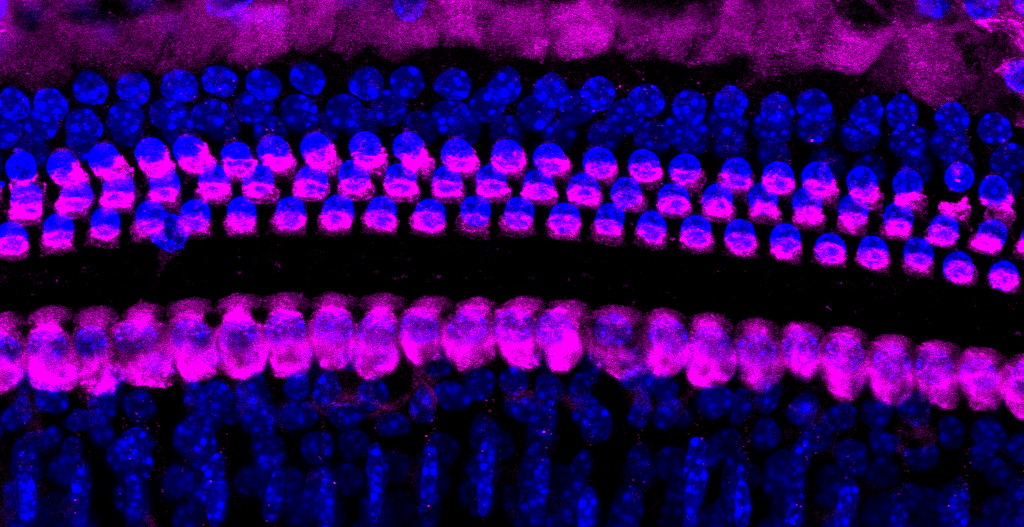

Supplement: Supplementary file 9 — Source data Fig. 1 [file 44321_2026_433_MOESM9_ESM.zip › Figure 1/1K/1m-wt-base-merge.tif]

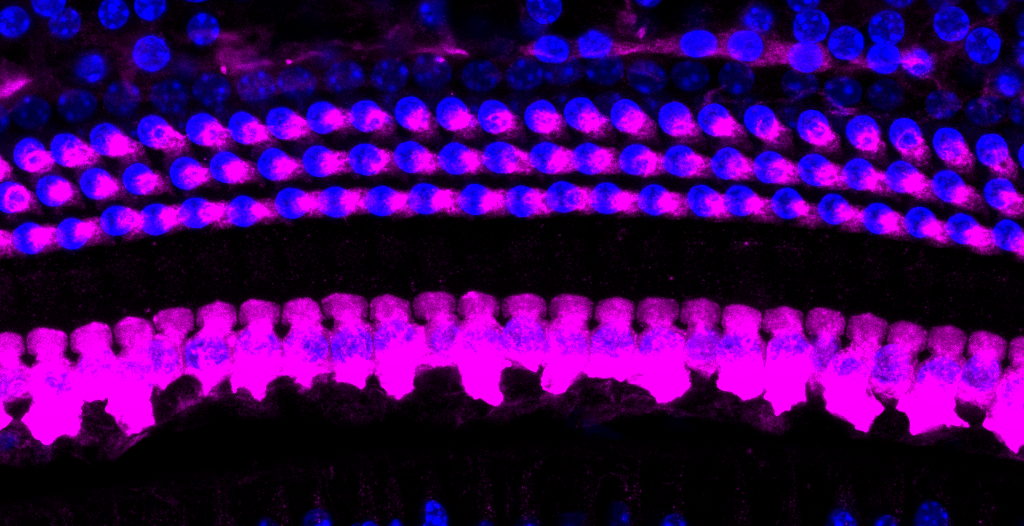

Supplement: Supplementary file 9 — Source data Fig. 1 [file 44321_2026_433_MOESM9_ESM.zip › Figure 1/1K/1m-wt-mid-merge.tif]

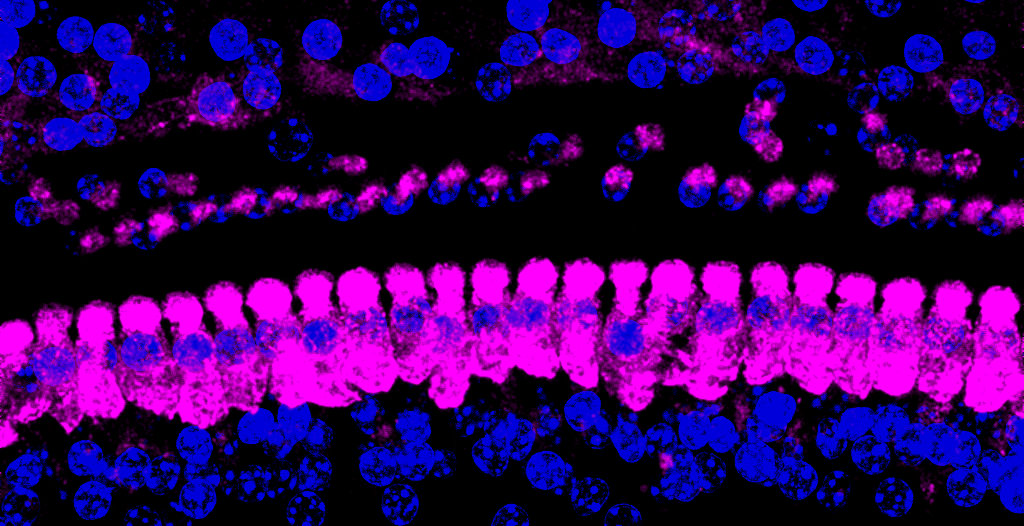

Supplement: Supplementary file 9 — Source data Fig. 1 [file 44321_2026_433_MOESM9_ESM.zip › Figure 1/1K/4w-het-apex-merge.tif]

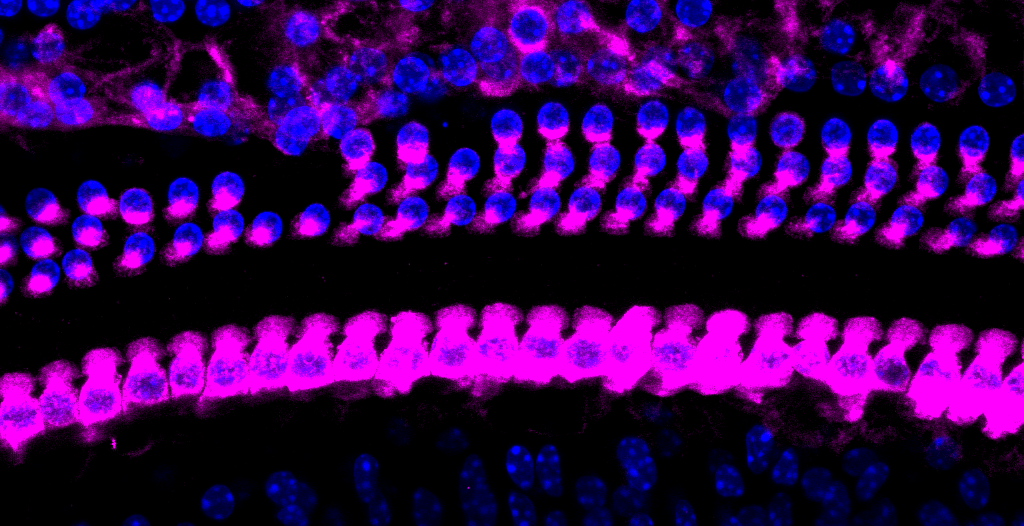

Supplement: Supplementary file 9 — Source data Fig. 1 [file 44321_2026_433_MOESM9_ESM.zip › Figure 1/1K/4w-het-base-merge.tif]

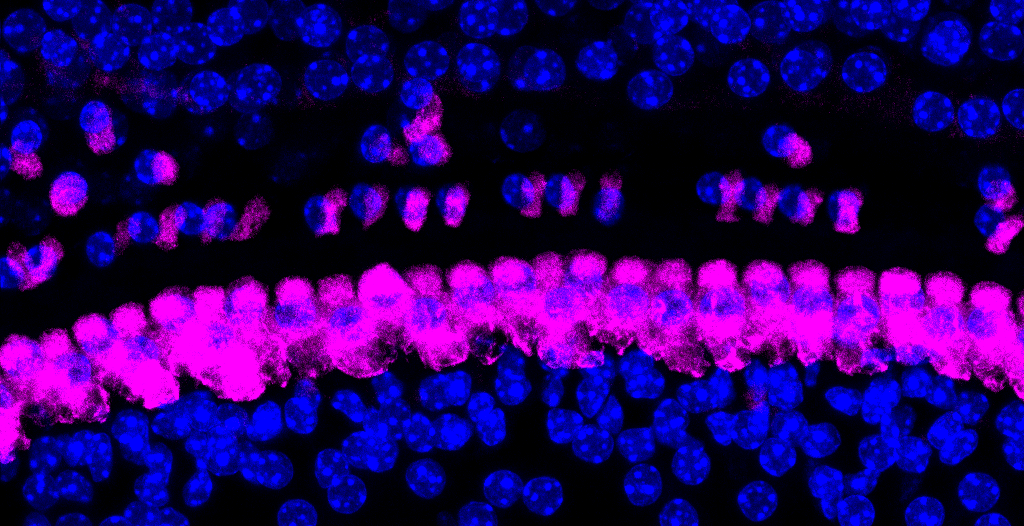

Supplement: Supplementary file 9 — Source data Fig. 1 [file 44321_2026_433_MOESM9_ESM.zip › Figure 1/1K/4w-hom-apex-merge.tif]

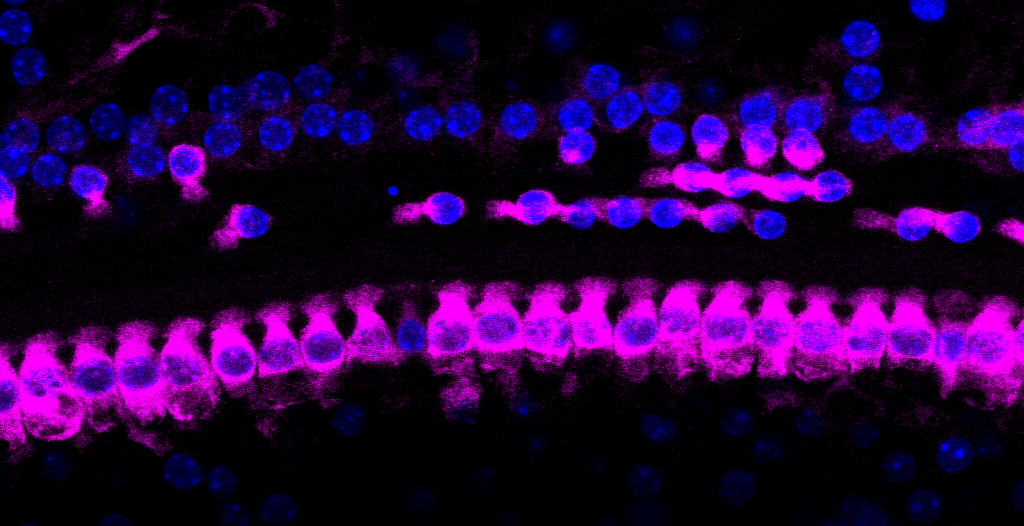

Supplement: Supplementary file 9 — Source data Fig. 1 [file 44321_2026_433_MOESM9_ESM.zip › Figure 1/1K/4w-hom-base-merge.tif]

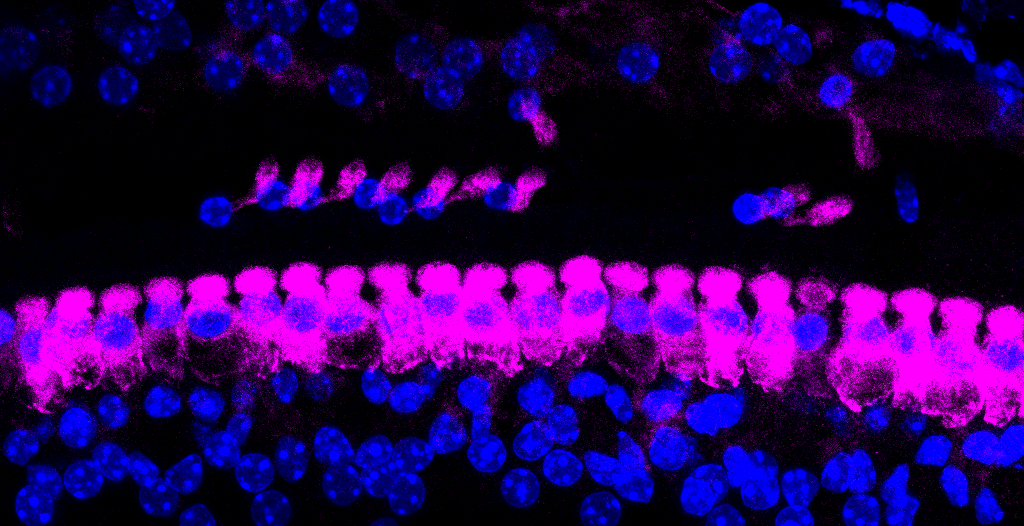

Supplement: Supplementary file 9 — Source data Fig. 1 [file 44321_2026_433_MOESM9_ESM.zip › Figure 1/1K/4w-hom-mid-merge.tif]

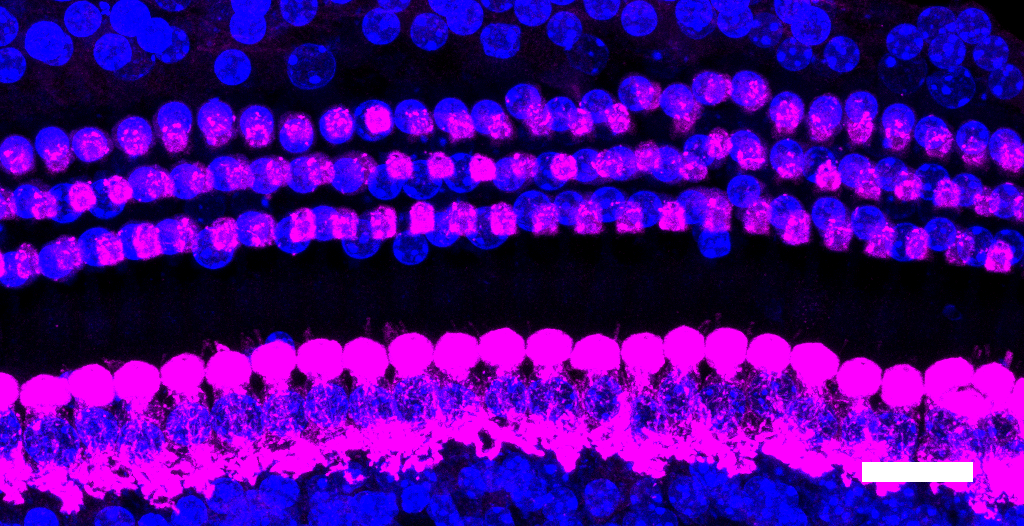

Supplement: Supplementary file 9 — Source data Fig. 1 [file 44321_2026_433_MOESM9_ESM.zip › Figure 1/1K/4w-wt-APEX-merge.tif]

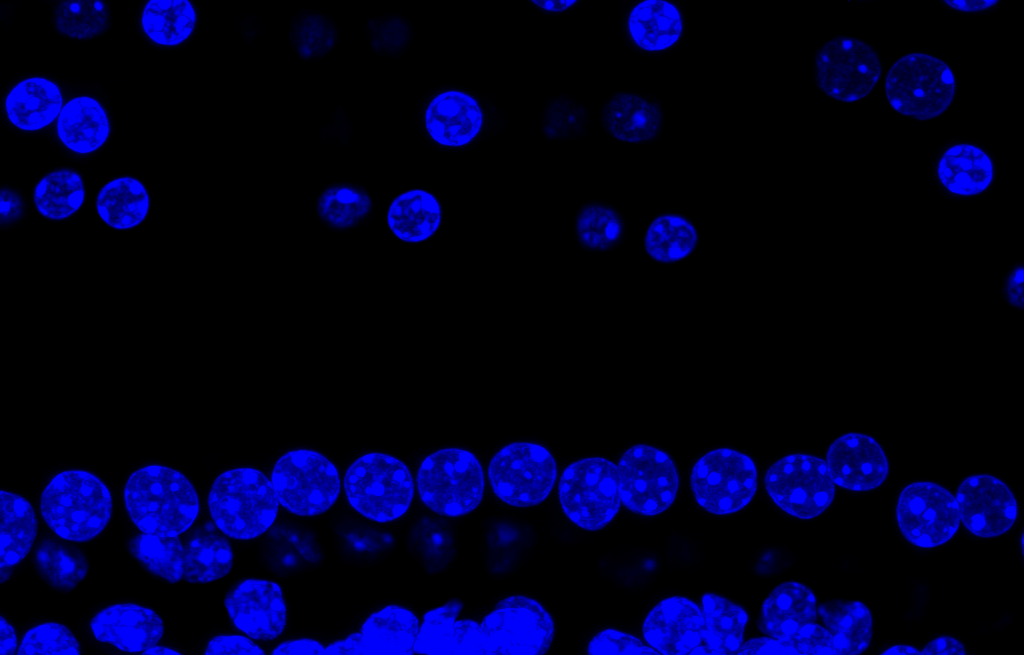

Supplement: Supplementary file 13 — Source data Fig. 5 [file 44321_2026_433_MOESM13_ESM.zip › Figure 5/5A/8w-ctrl-APEX-dapi.tif]

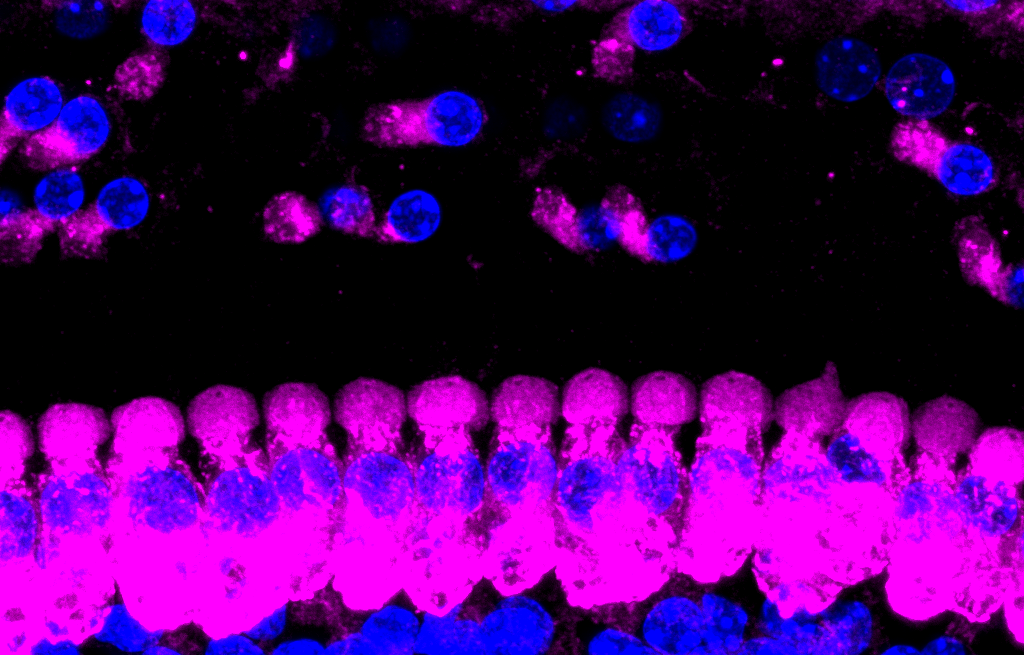

Supplement: Supplementary file 13 — Source data Fig. 5 [file 44321_2026_433_MOESM13_ESM.zip › Figure 5/5A/8w-ctrl-APEX-merge.tif]

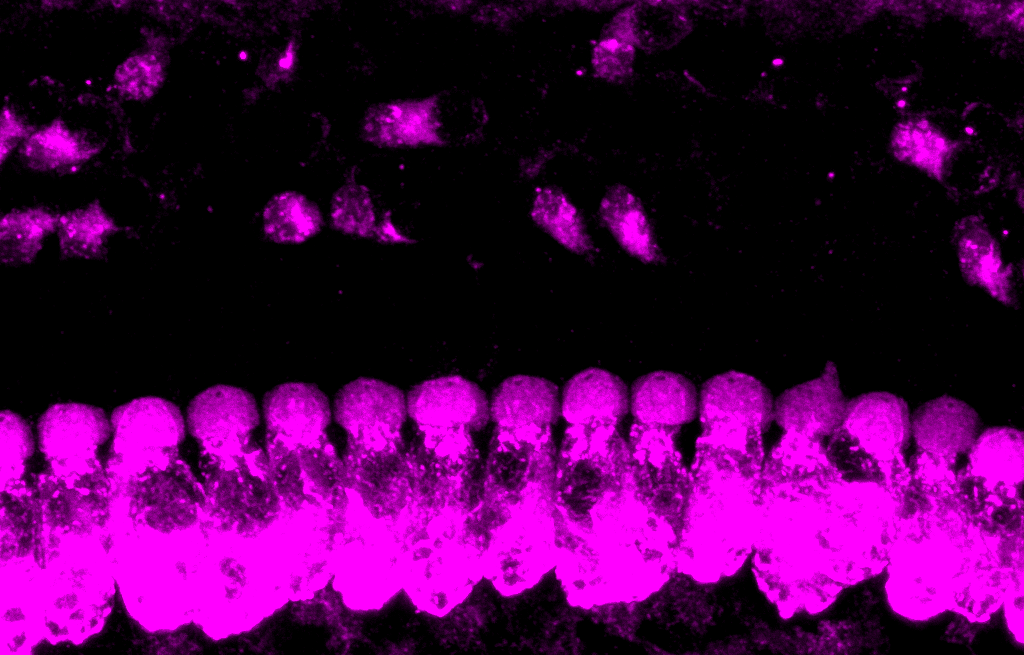

Supplement: Supplementary file 13 — Source data Fig. 5 [file 44321_2026_433_MOESM13_ESM.zip › Figure 5/5A/8w-ctrl-APEX-myo.tif]

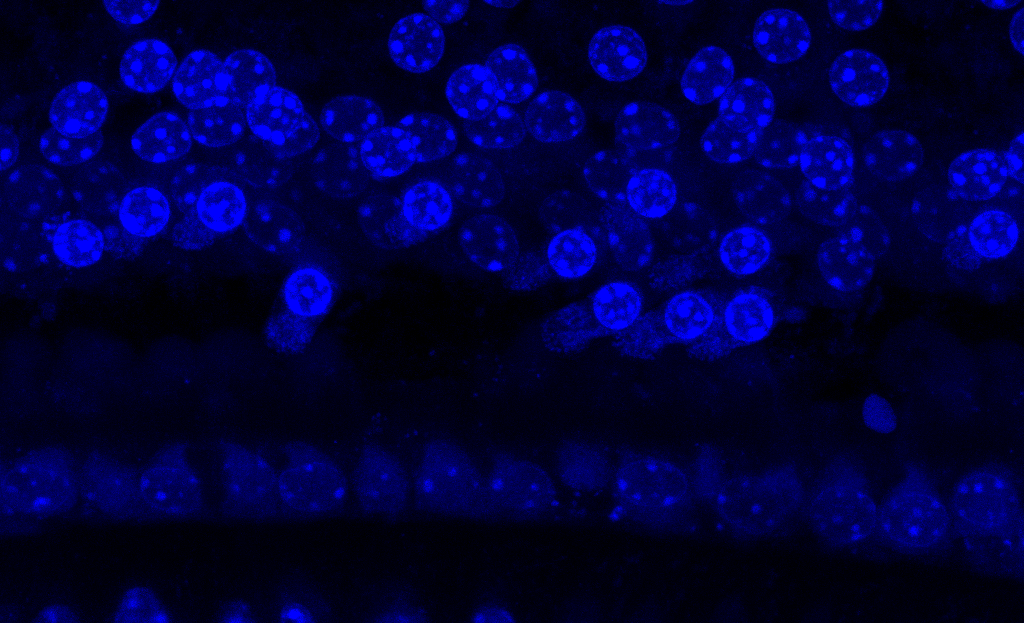

Supplement: Supplementary file 13 — Source data Fig. 5 [file 44321_2026_433_MOESM13_ESM.zip › Figure 5/5A/8w-ctrl-Base-DAPI.tif]

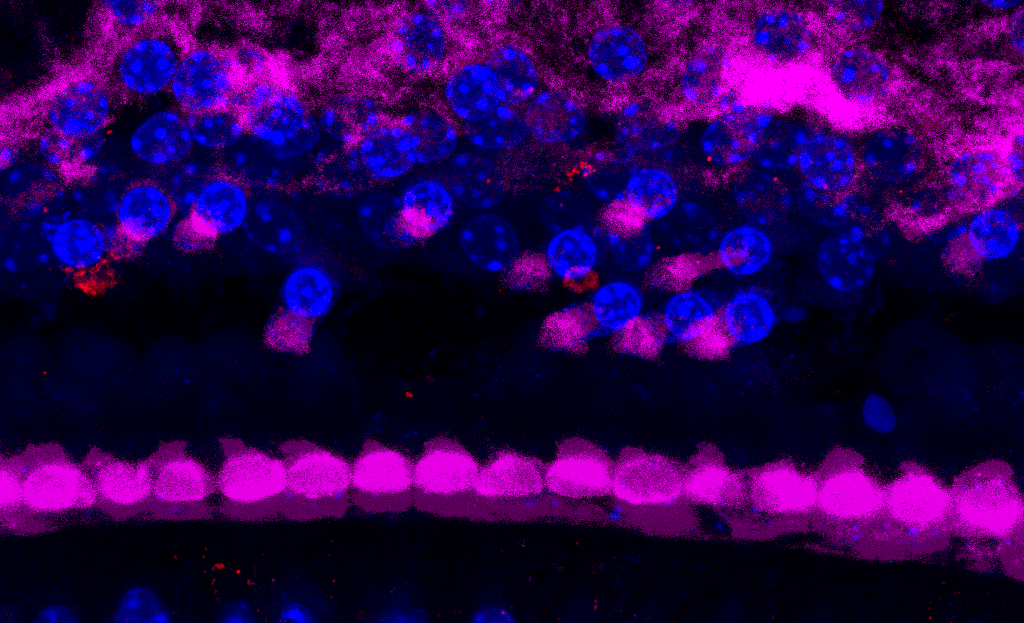

Supplement: Supplementary file 13 — Source data Fig. 5 [file 44321_2026_433_MOESM13_ESM.zip › Figure 5/5A/8w-ctrl-Base-MERGE.tif]

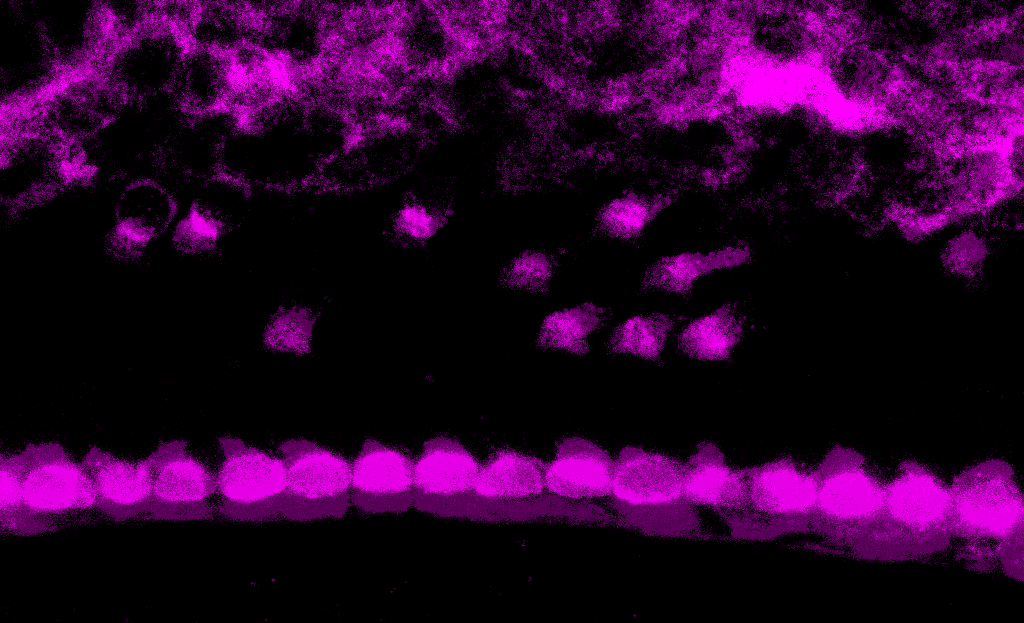

Supplement: Supplementary file 13 — Source data Fig. 5 [file 44321_2026_433_MOESM13_ESM.zip › Figure 5/5A/8w-ctrl-Base-MYO.tif]

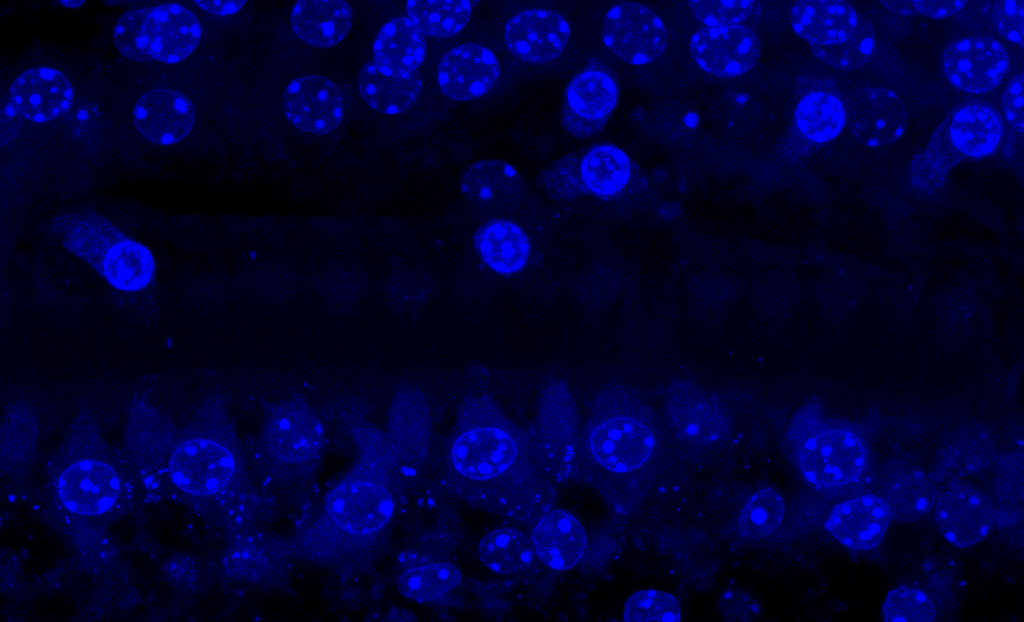

Supplement: Supplementary file 13 — Source data Fig. 5 [file 44321_2026_433_MOESM13_ESM.zip › Figure 5/5A/8w-ctrl-MIDDLE-DAPI.tif]

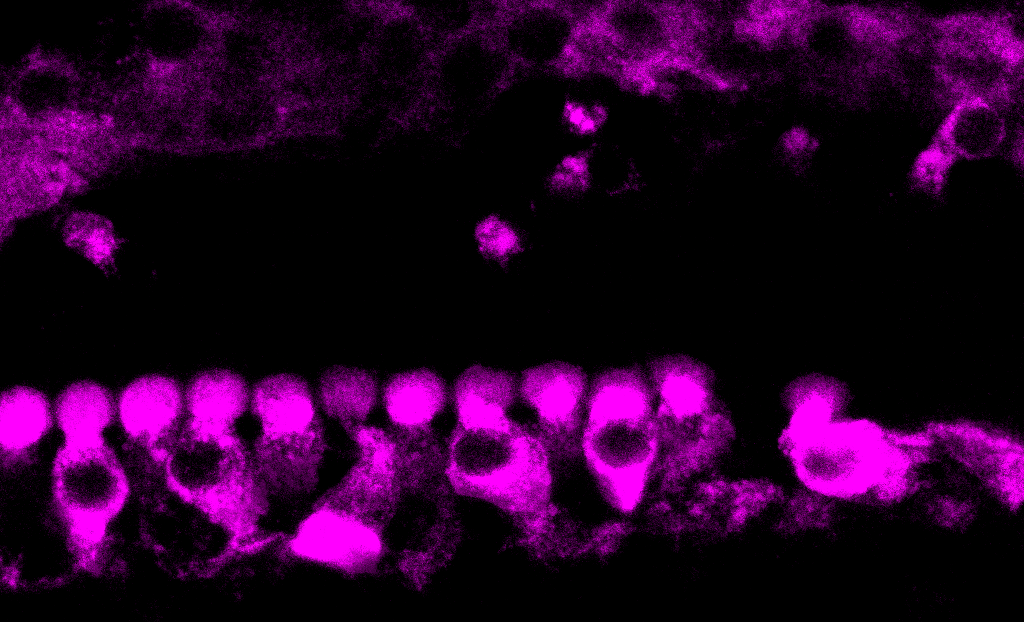

Supplement: Supplementary file 13 — Source data Fig. 5 [file 44321_2026_433_MOESM13_ESM.zip › Figure 5/5A/8w-ctrl-MIDDLE-MYO.tif]

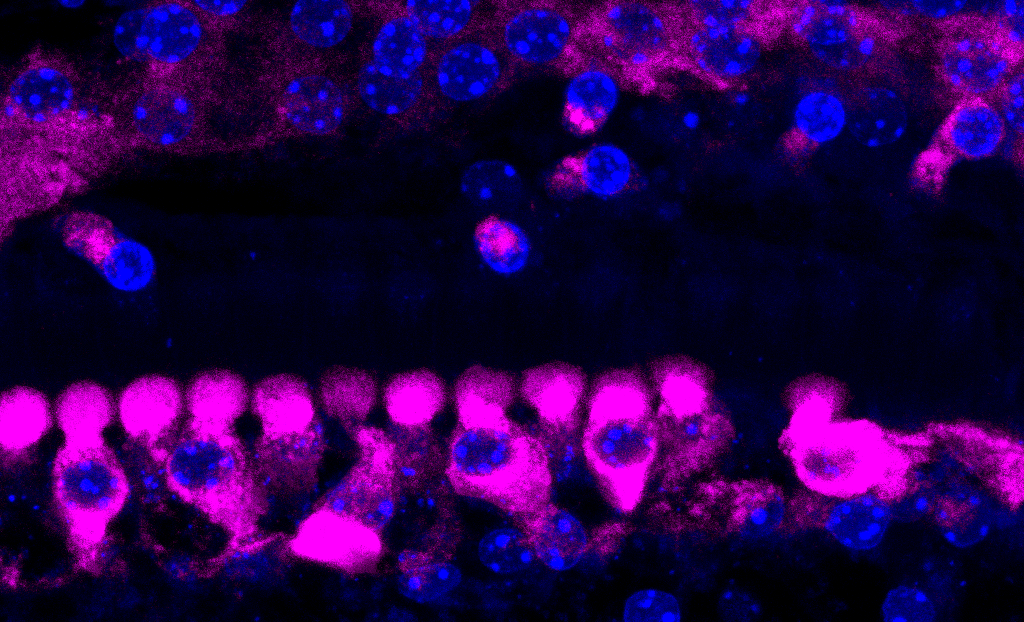

Supplement: Supplementary file 13 — Source data Fig. 5 [file 44321_2026_433_MOESM13_ESM.zip › Figure 5/5A/8w-ctrl-MIDDLE-merge.tif]

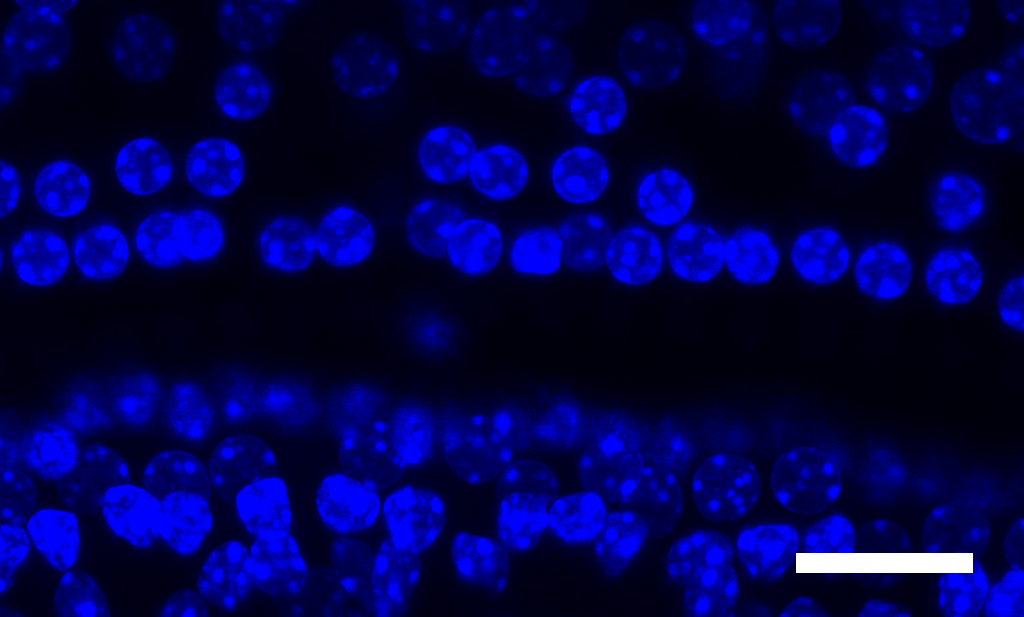

Supplement: Supplementary file 13 — Source data Fig. 5 [file 44321_2026_433_MOESM13_ESM.zip › Figure 5/5A/8w-high-apex-DAPI.tif]

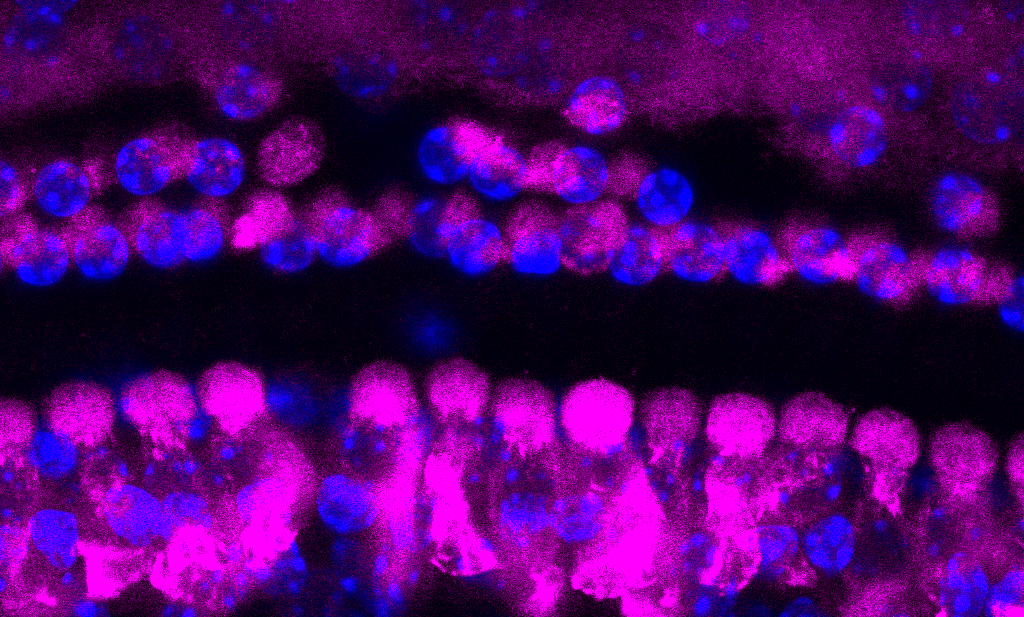

Supplement: Supplementary file 13 — Source data Fig. 5 [file 44321_2026_433_MOESM13_ESM.zip › Figure 5/5A/8w-high-apex-MERGE.tif]

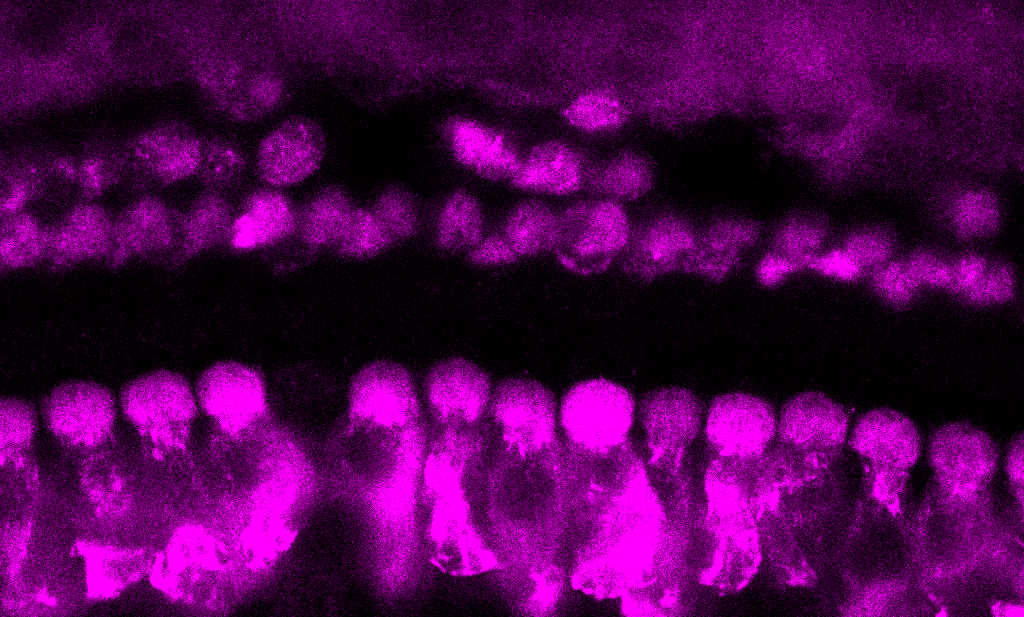

Supplement: Supplementary file 13 — Source data Fig. 5 [file 44321_2026_433_MOESM13_ESM.zip › Figure 5/5A/8w-high-apex-MYO.tif]

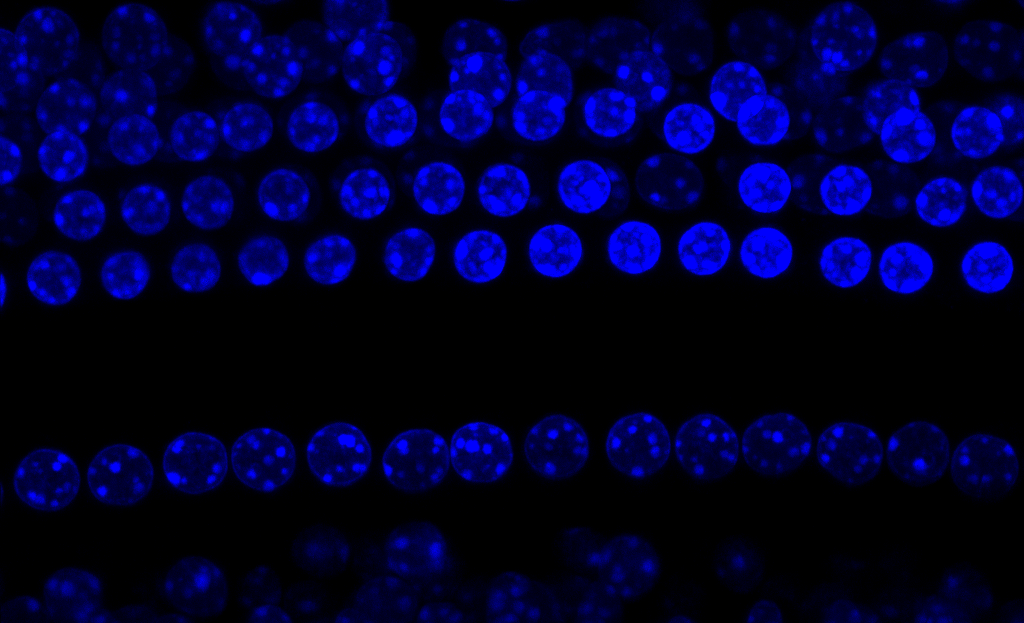

Supplement: Supplementary file 13 — Source data Fig. 5 [file 44321_2026_433_MOESM13_ESM.zip › Figure 5/5A/8w-high-base-DAPI.tif]

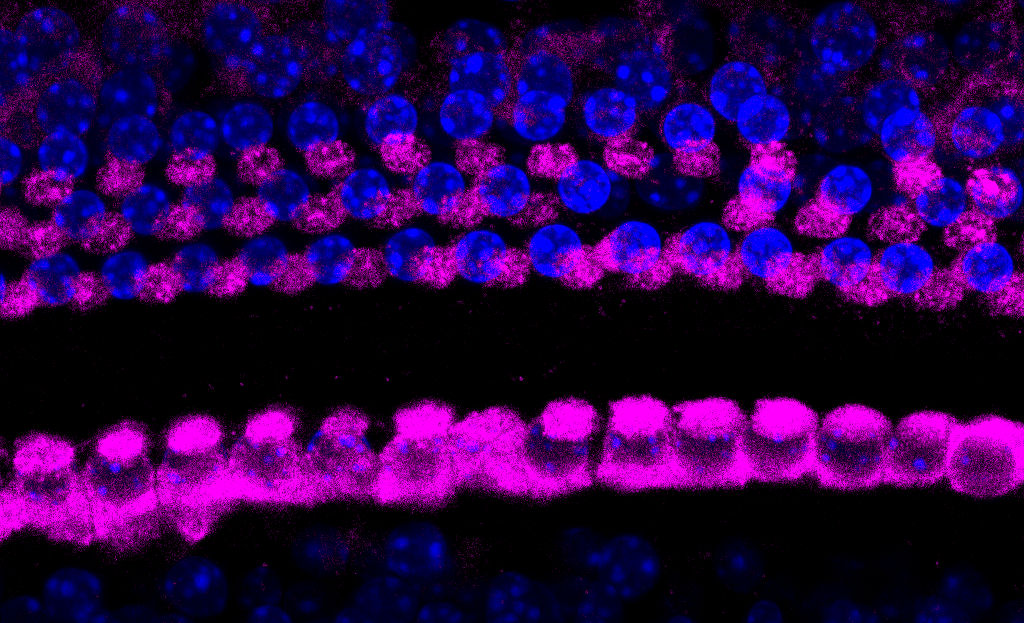

Supplement: Supplementary file 13 — Source data Fig. 5 [file 44321_2026_433_MOESM13_ESM.zip › Figure 5/5A/8w-high-base-MERGE.tif]

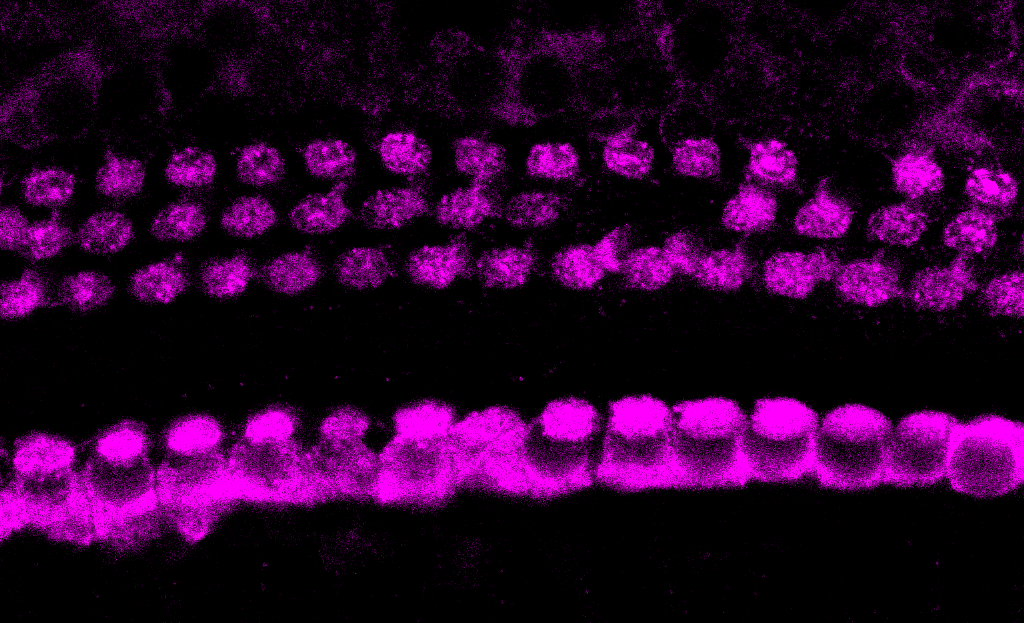

Supplement: Supplementary file 13 — Source data Fig. 5 [file 44321_2026_433_MOESM13_ESM.zip › Figure 5/5A/8w-high-base-MYO.tif]

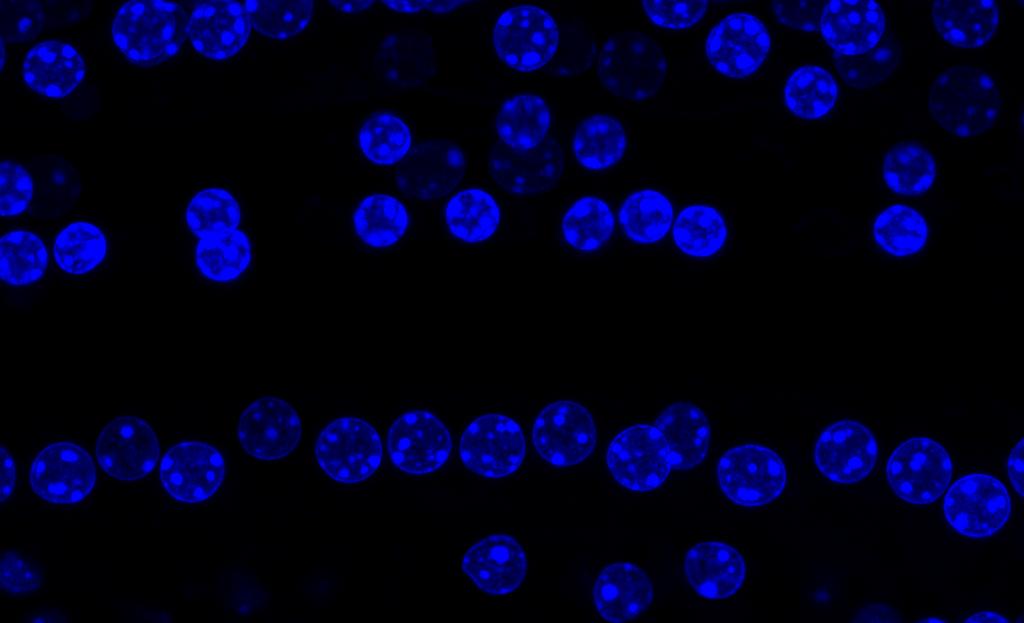

Supplement: Supplementary file 13 — Source data Fig. 5 [file 44321_2026_433_MOESM13_ESM.zip › Figure 5/5A/8w-high-mid-DAPI.tif]

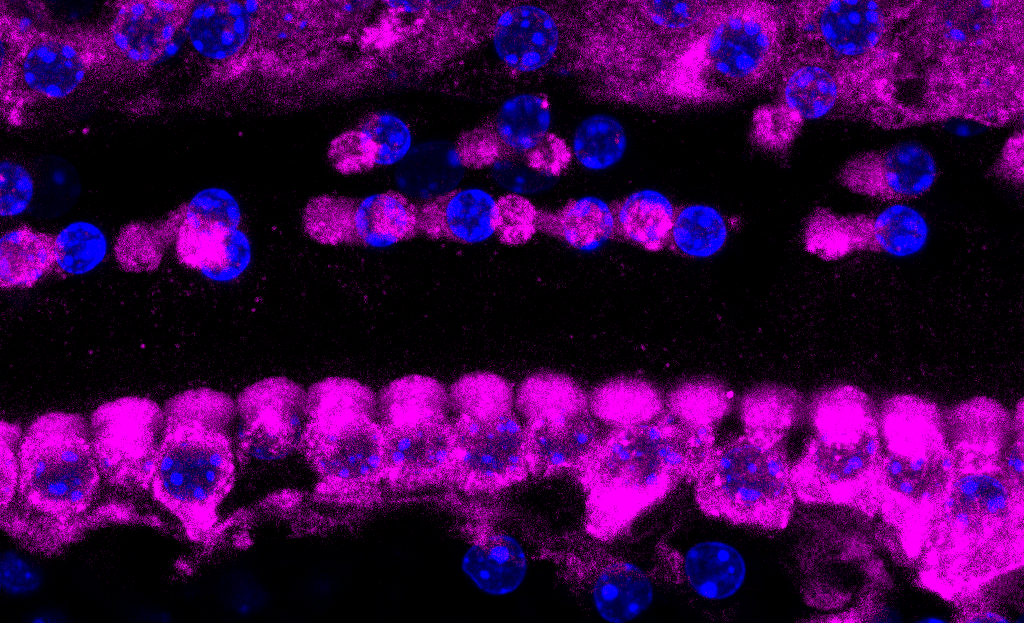

Supplement: Supplementary file 13 — Source data Fig. 5 [file 44321_2026_433_MOESM13_ESM.zip › Figure 5/5A/8w-high-mid-MERGE.tif]

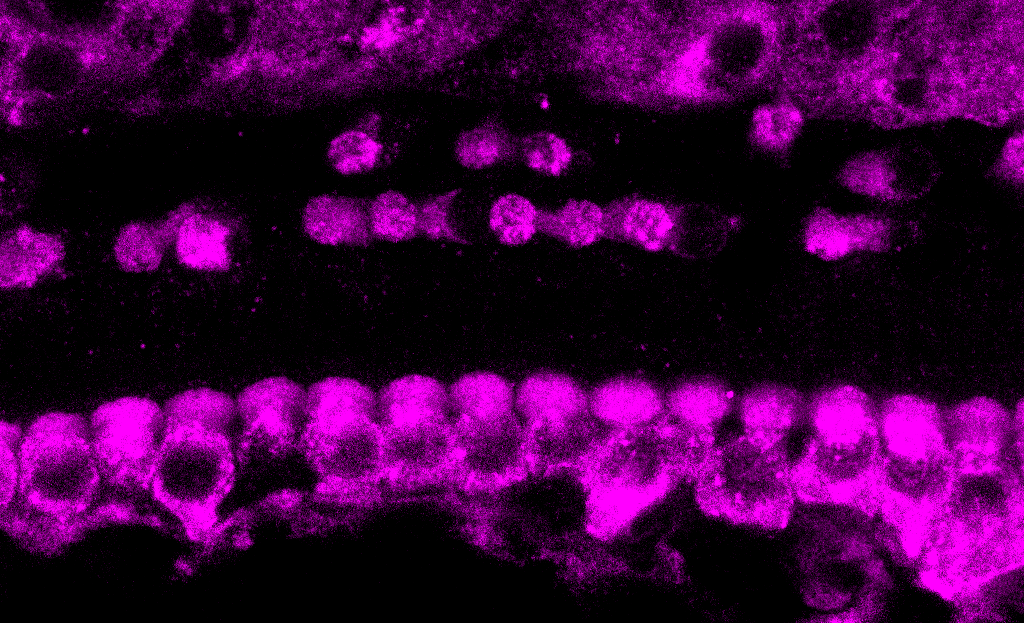

Supplement: Supplementary file 13 — Source data Fig. 5 [file 44321_2026_433_MOESM13_ESM.zip › Figure 5/5A/8w-high-mid-MYO.tif]

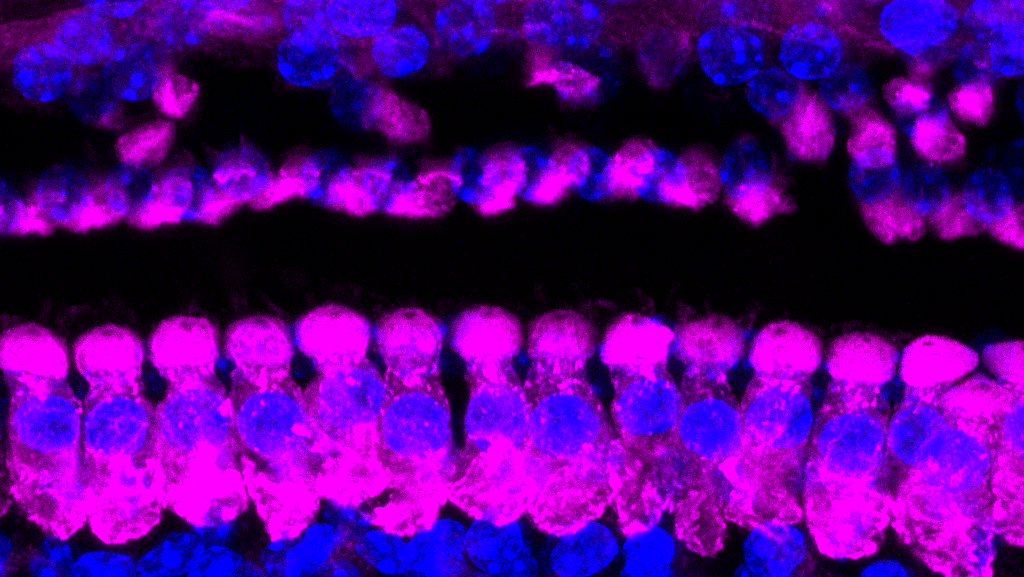

Supplement: Supplementary file 13 — Source data Fig. 5 [file 44321_2026_433_MOESM13_ESM.zip › Figure 5/5A/8w-low-apex- MERGE.tif]

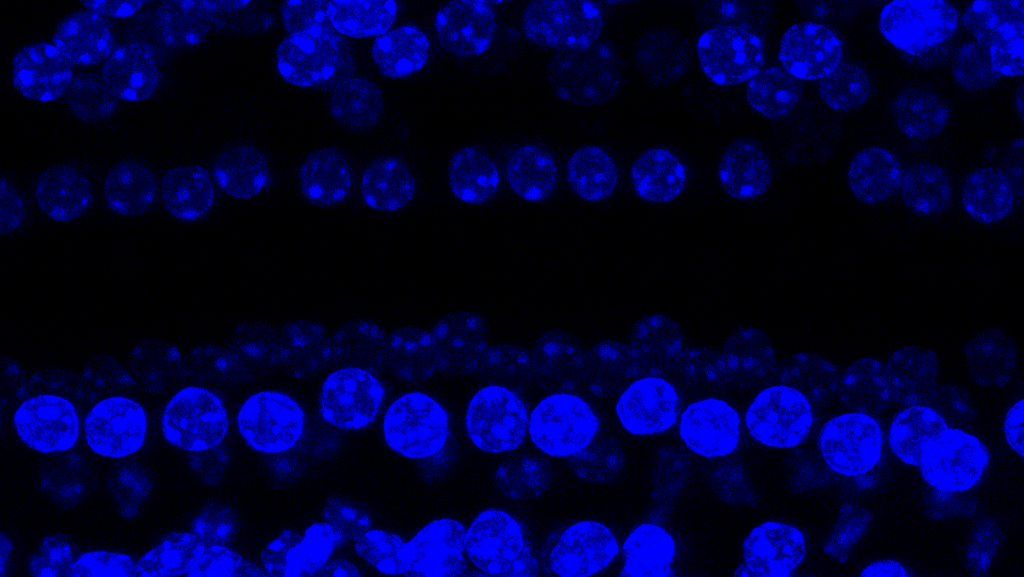

Supplement: Supplementary file 13 — Source data Fig. 5 [file 44321_2026_433_MOESM13_ESM.zip › Figure 5/5A/8w-low-apex-DAPI.tif]

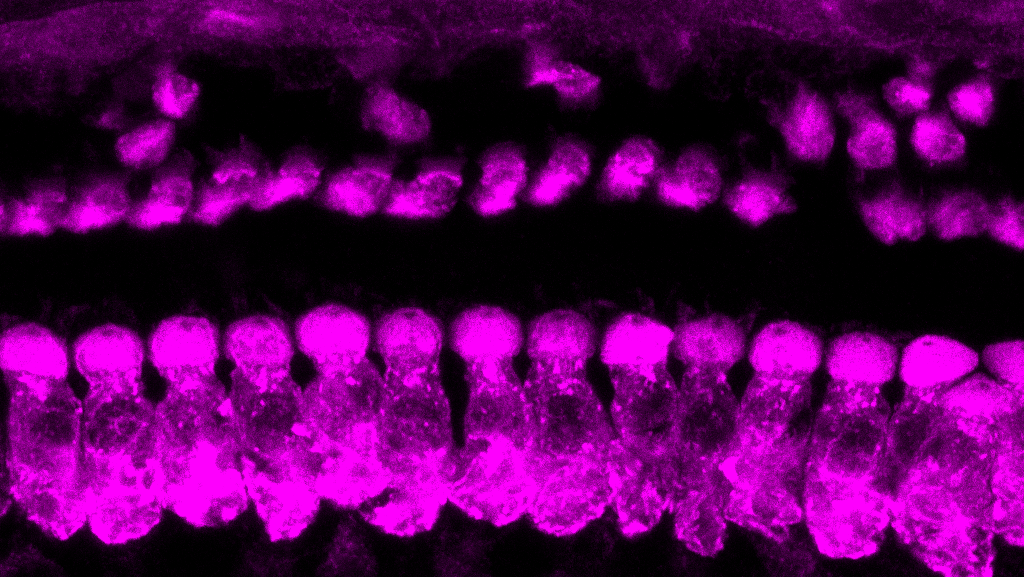

Supplement: Supplementary file 13 — Source data Fig. 5 [file 44321_2026_433_MOESM13_ESM.zip › Figure 5/5A/8w-low-apex-MYO.tif]

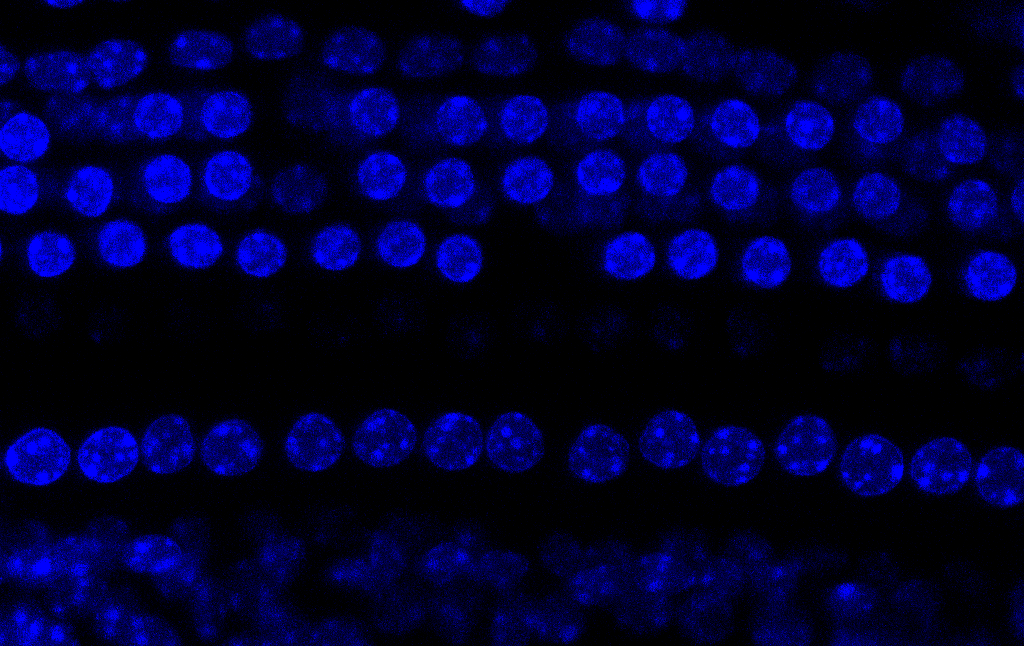

Supplement: Supplementary file 13 — Source data Fig. 5 [file 44321_2026_433_MOESM13_ESM.zip › Figure 5/5A/8w-low-base-DAPI.tif]

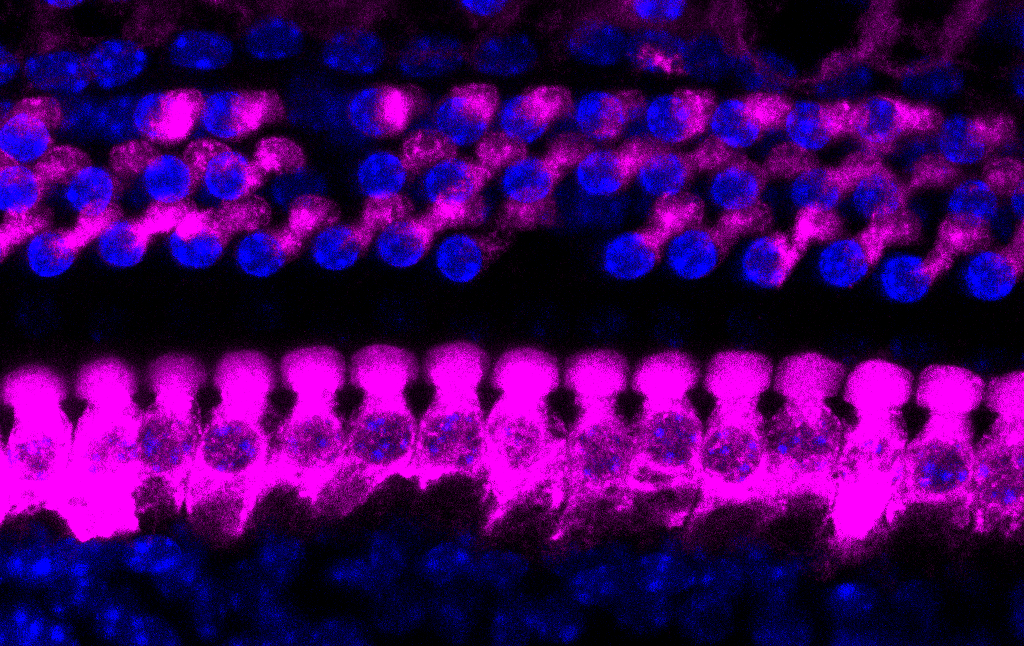

Supplement: Supplementary file 13 — Source data Fig. 5 [file 44321_2026_433_MOESM13_ESM.zip › Figure 5/5A/8w-low-base-MERGE.tif]

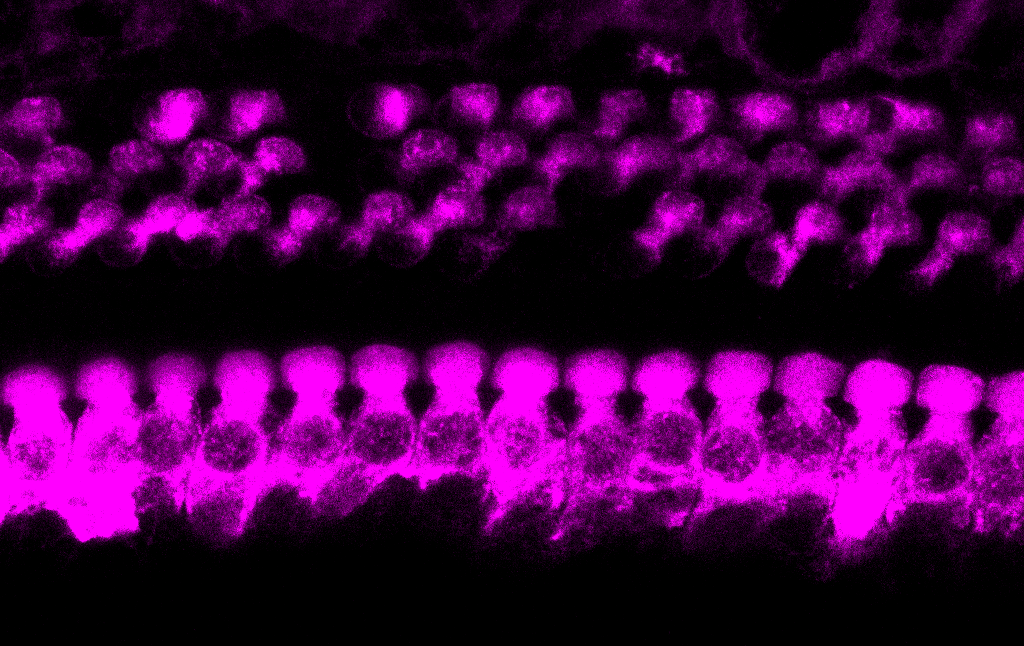

Supplement: Supplementary file 13 — Source data Fig. 5 [file 44321_2026_433_MOESM13_ESM.zip › Figure 5/5A/8w-low-base-MYO.tif]

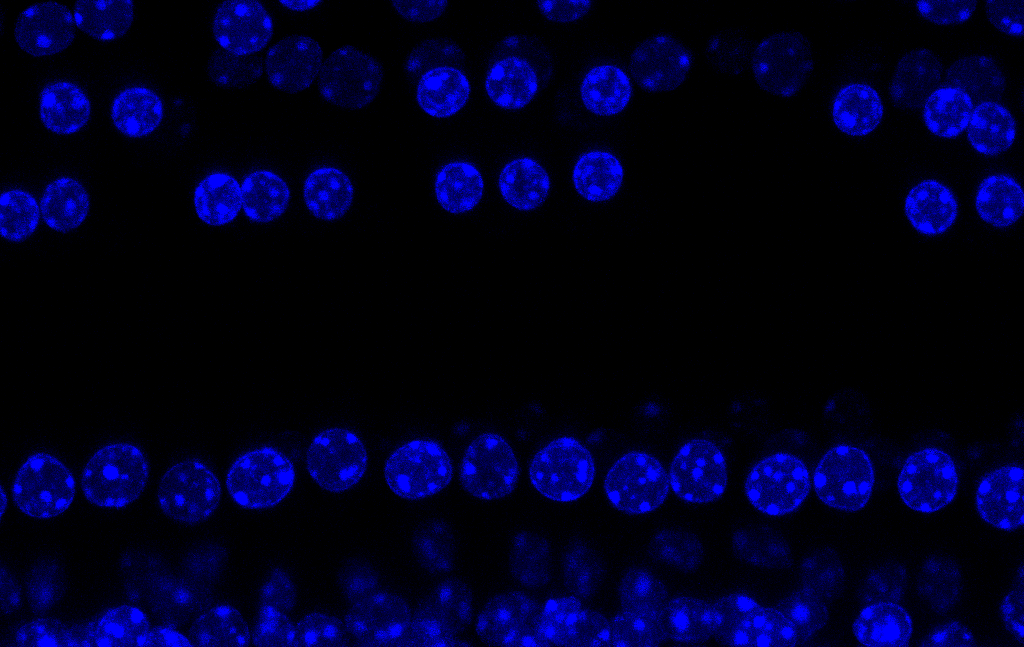

Supplement: Supplementary file 13 — Source data Fig. 5 [file 44321_2026_433_MOESM13_ESM.zip › Figure 5/5A/8w-low-mid-DAPI.tif]

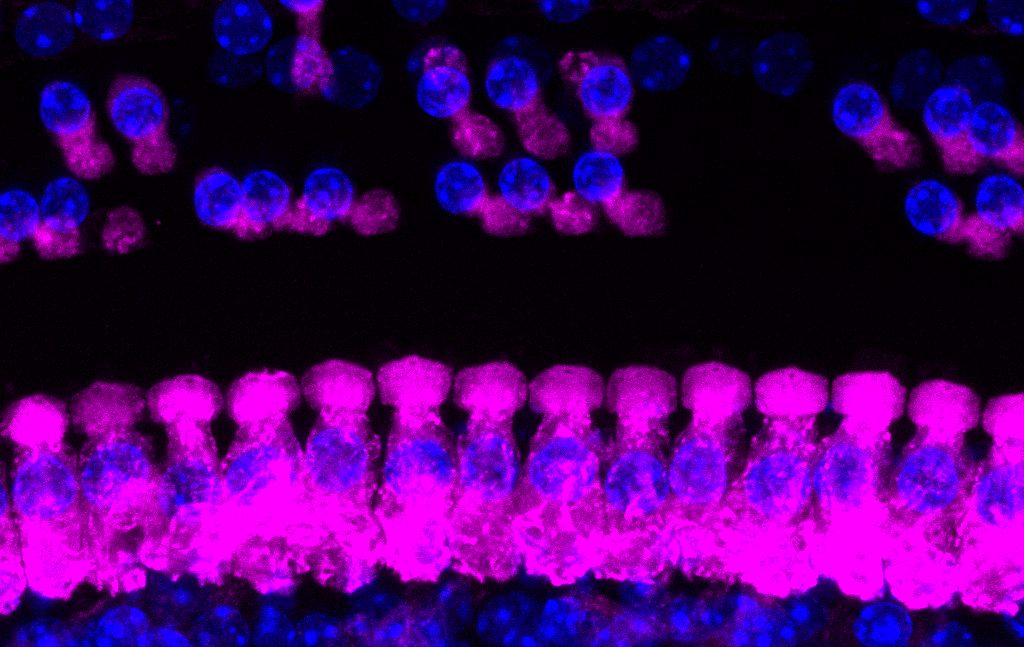

Supplement: Supplementary file 13 — Source data Fig. 5 [file 44321_2026_433_MOESM13_ESM.zip › Figure 5/5A/8w-low-mid-MERGE.tif]

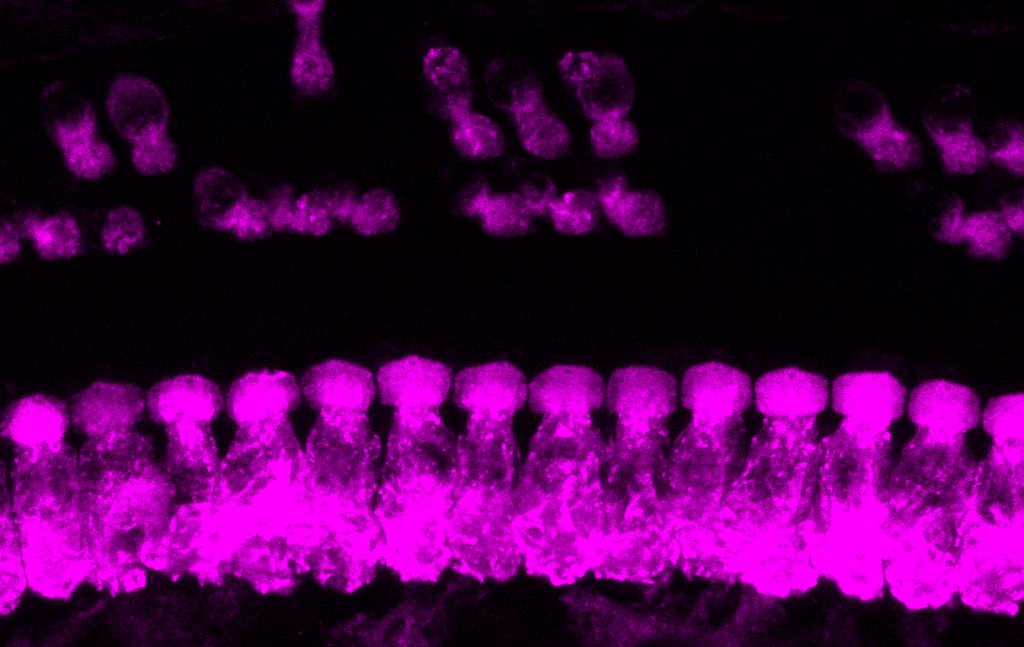

Supplement: Supplementary file 13 — Source data Fig. 5 [file 44321_2026_433_MOESM13_ESM.zip › Figure 5/5A/8w-low-mid-MYO.tif]

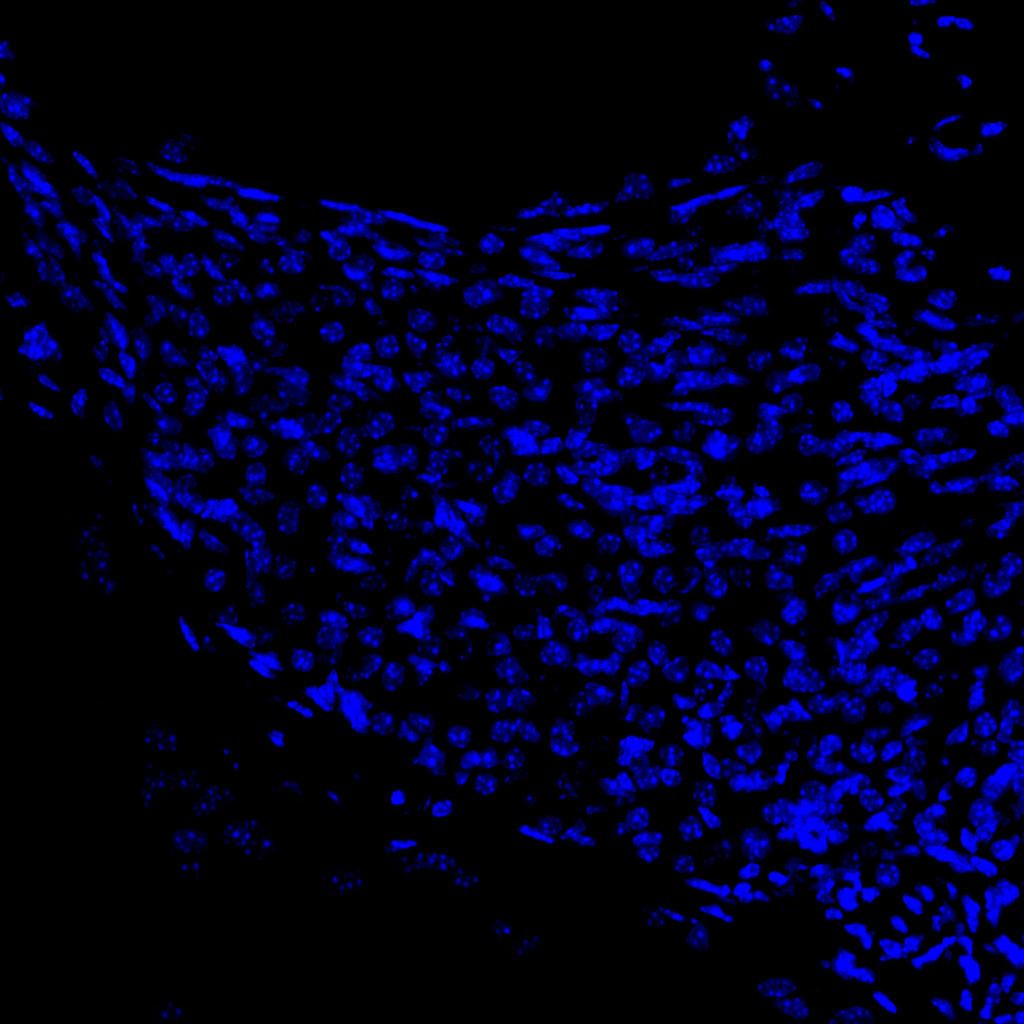

Supplement: Supplementary file 13 — Source data Fig. 5 [file 44321_2026_433_MOESM13_ESM.zip › Figure 5/5D/8w-ctrl-APEX-DAPI.tif]

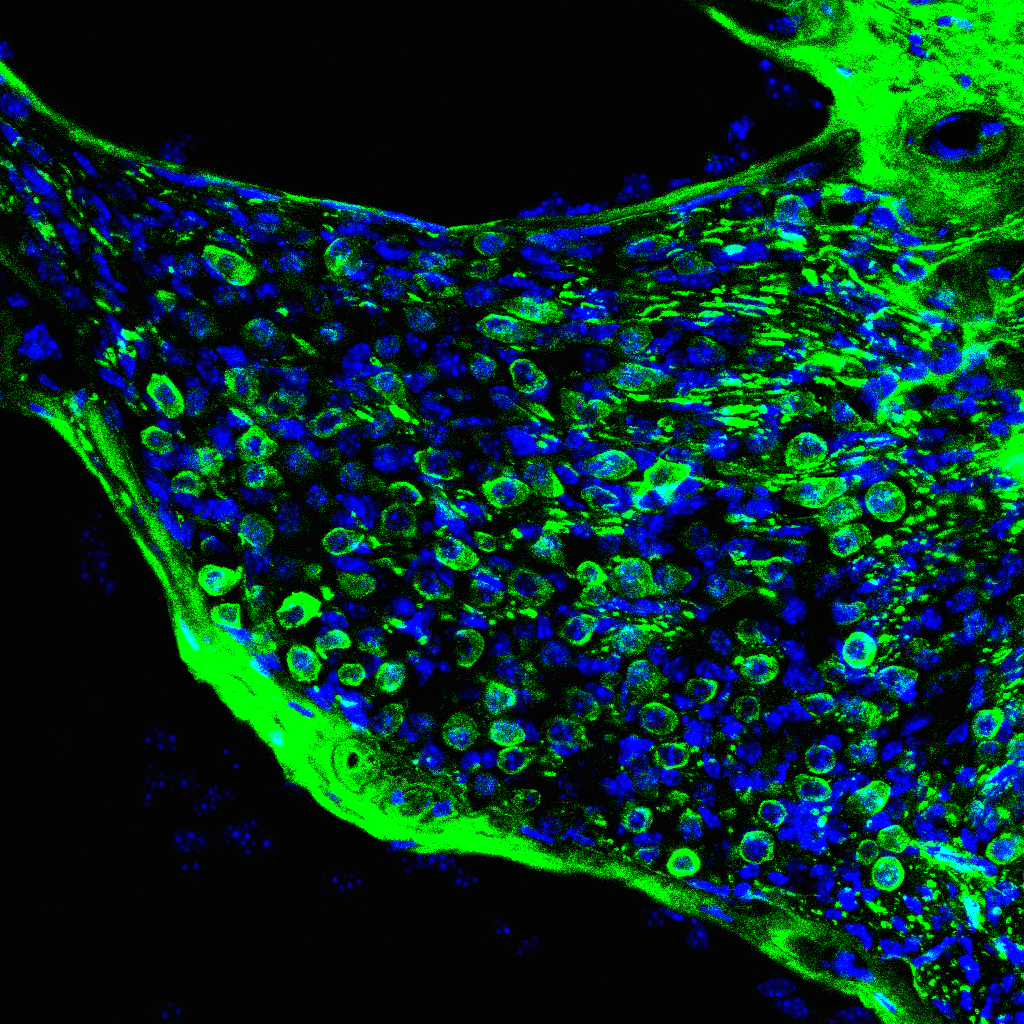

Supplement: Supplementary file 13 — Source data Fig. 5 [file 44321_2026_433_MOESM13_ESM.zip › Figure 5/5D/8w-ctrl-APEX-MERGE.tif]

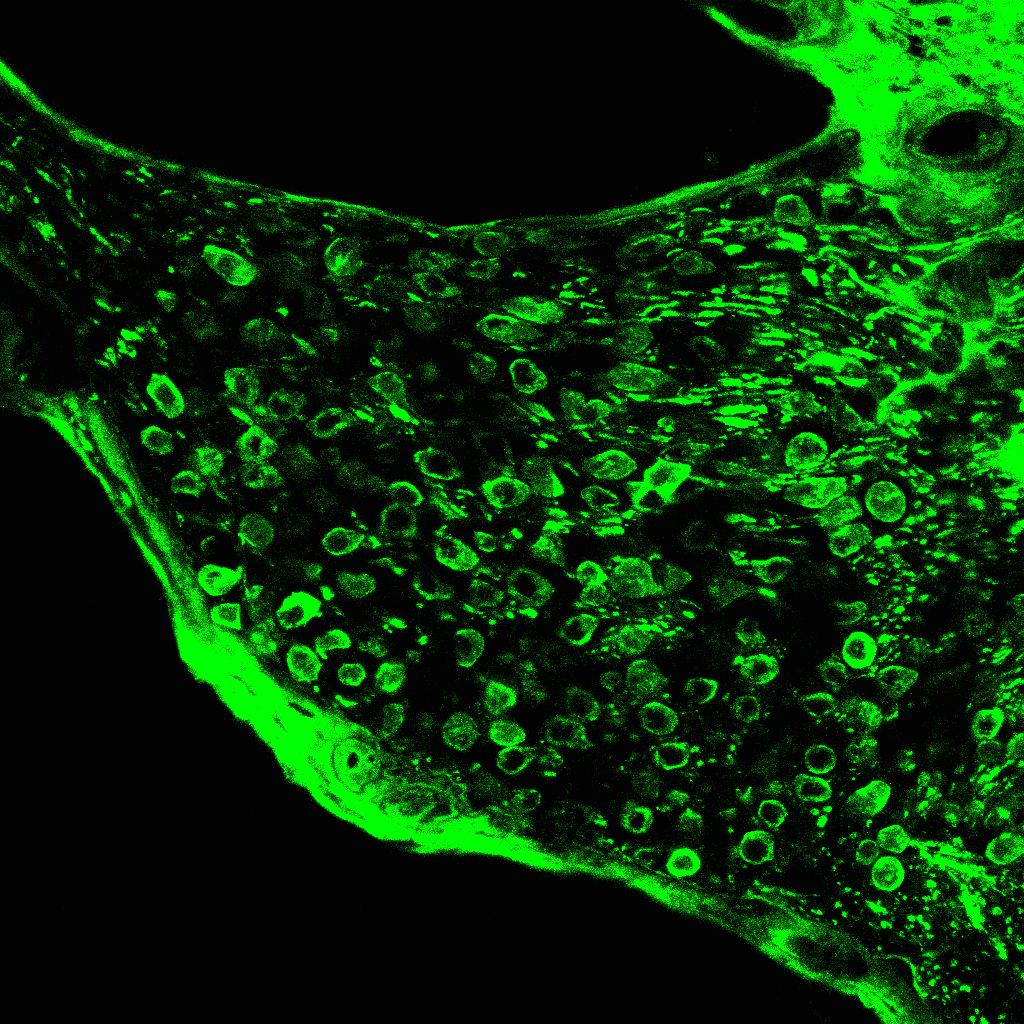

Supplement: Supplementary file 13 — Source data Fig. 5 [file 44321_2026_433_MOESM13_ESM.zip › Figure 5/5D/8w-ctrl-APEX-TUJ1.tif]

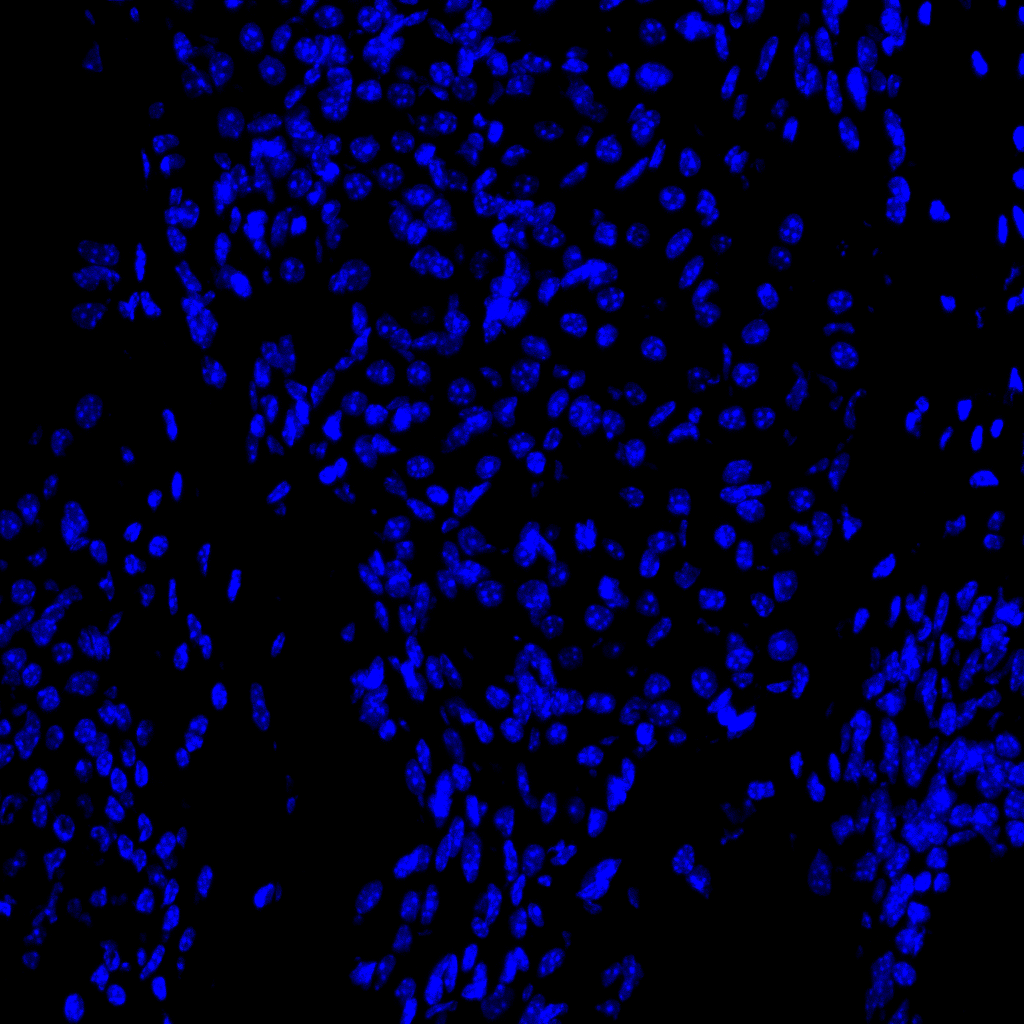

Supplement: Supplementary file 13 — Source data Fig. 5 [file 44321_2026_433_MOESM13_ESM.zip › Figure 5/5D/8w-ctrl-BASE-DAPI.tif]

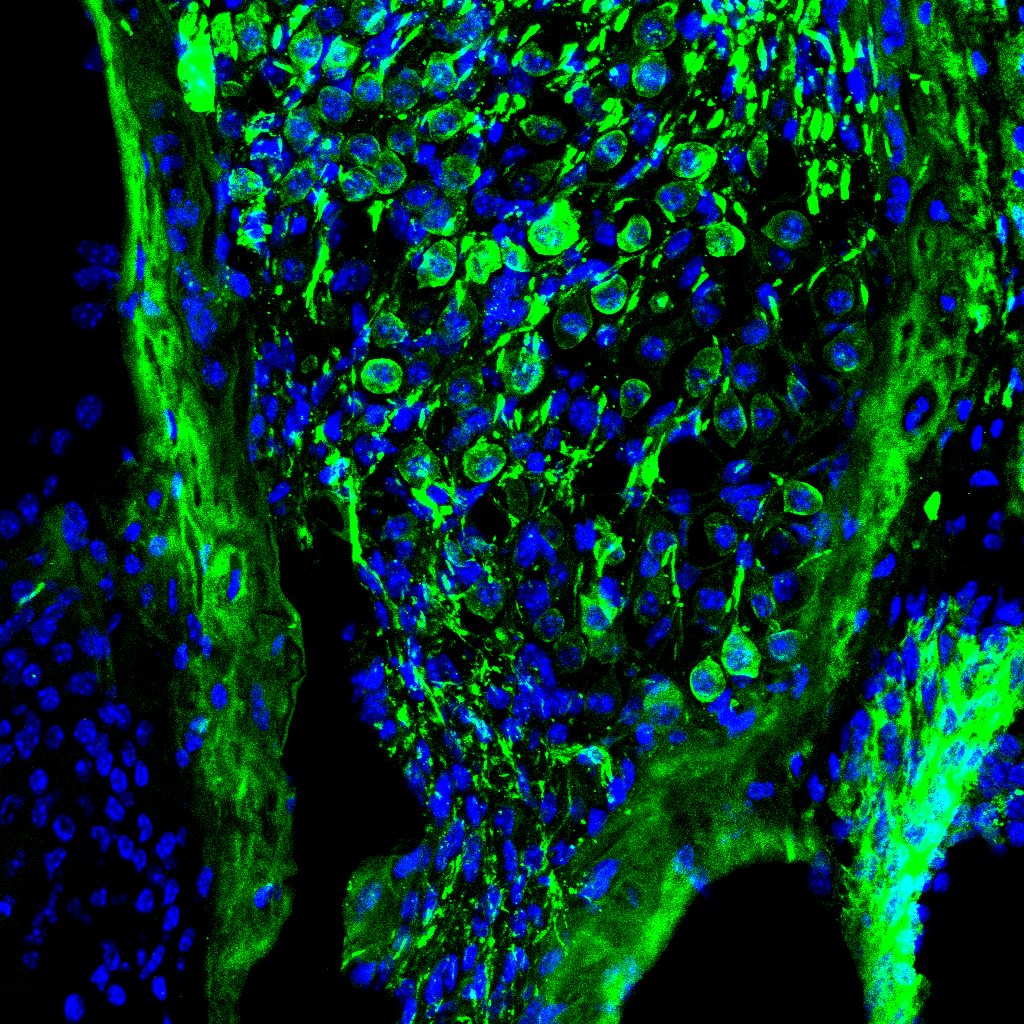

Supplement: Supplementary file 13 — Source data Fig. 5 [file 44321_2026_433_MOESM13_ESM.zip › Figure 5/5D/8w-ctrl-BASE-MERGE.tif]

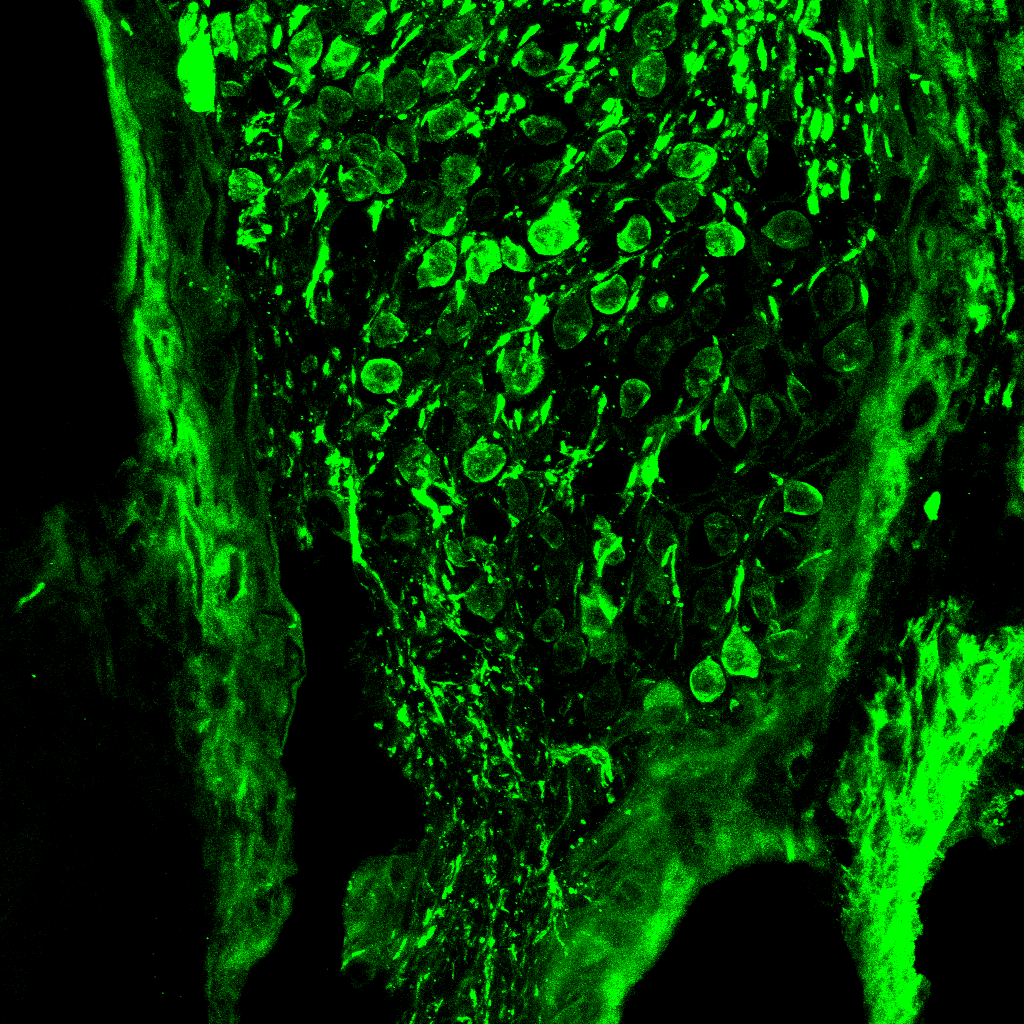

Supplement: Supplementary file 13 — Source data Fig. 5 [file 44321_2026_433_MOESM13_ESM.zip › Figure 5/5D/8w-ctrl-BASE-TUJ1.tif]

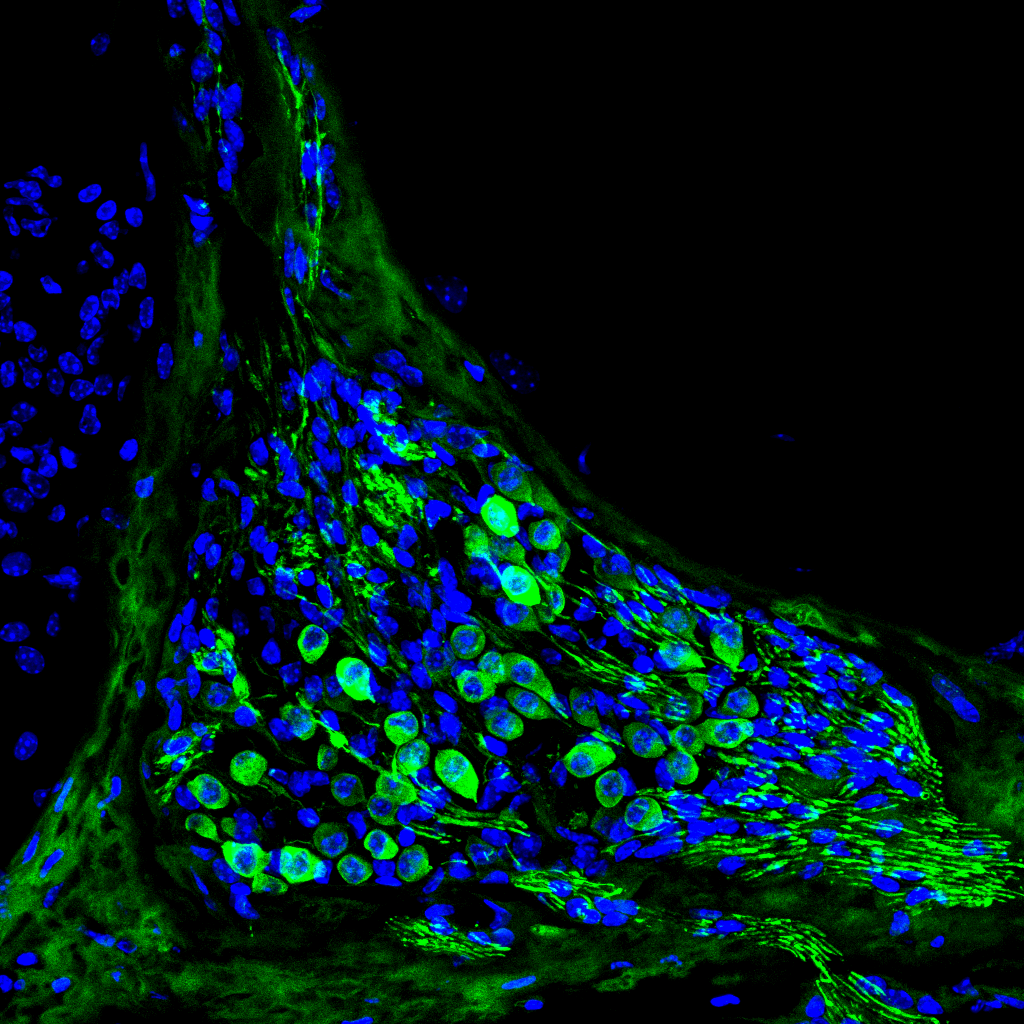

Supplement: Supplementary file 13 — Source data Fig. 5 [file 44321_2026_433_MOESM13_ESM.zip › Figure 5/5D/8w-ctrl-mic-merge.tif]

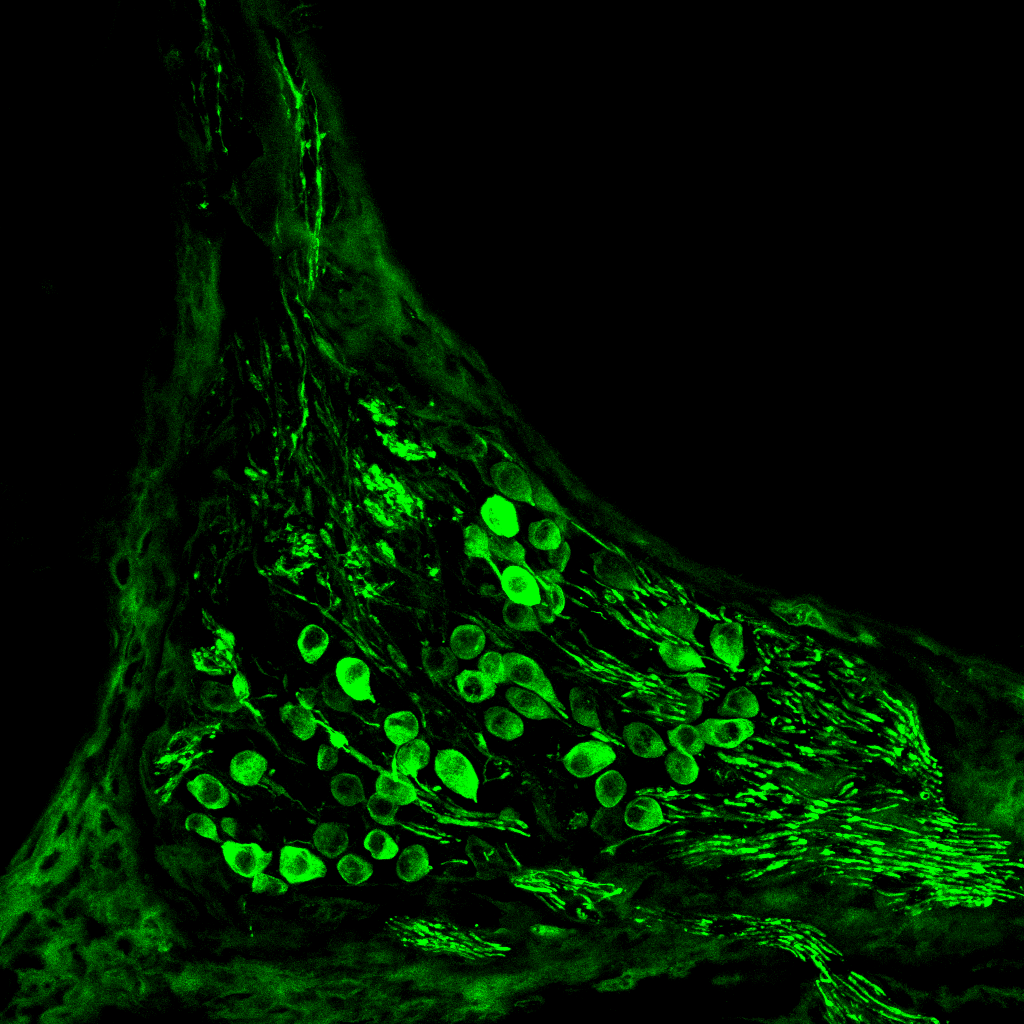

Supplement: Supplementary file 13 — Source data Fig. 5 [file 44321_2026_433_MOESM13_ESM.zip › Figure 5/5D/8w-ctrl-mic-tuj1.tif]

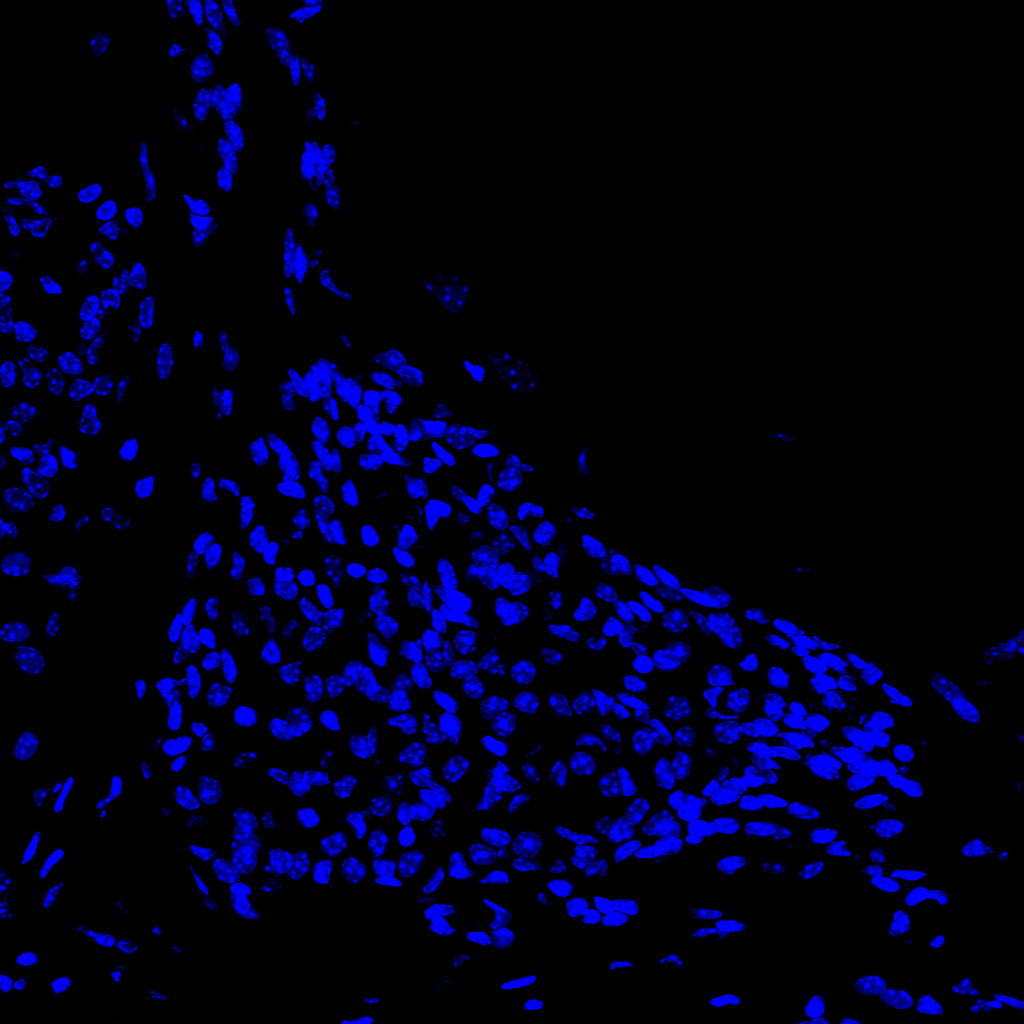

Supplement: Supplementary file 13 — Source data Fig. 5 [file 44321_2026_433_MOESM13_ESM.zip › Figure 5/5D/8w-ctrl-mid-dapi.tif]

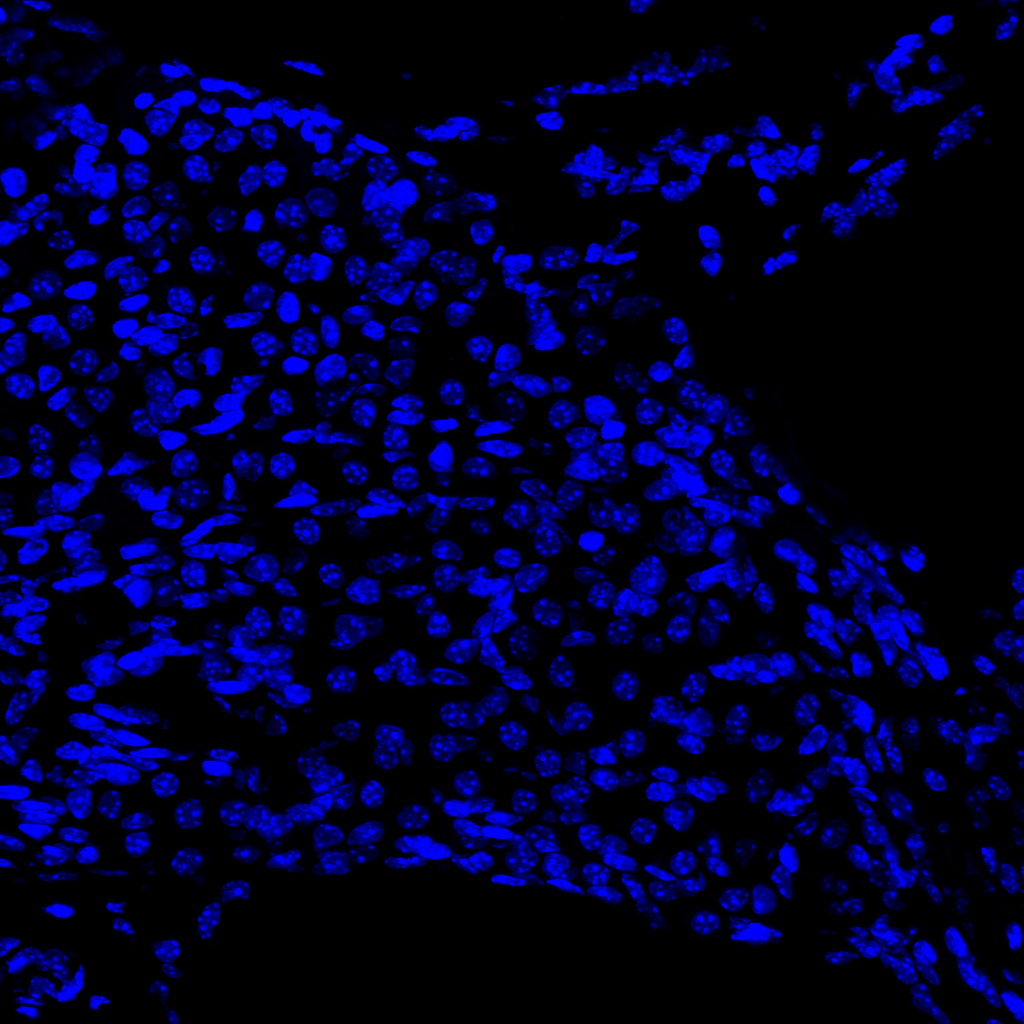

Supplement: Supplementary file 13 — Source data Fig. 5 [file 44321_2026_433_MOESM13_ESM.zip › Figure 5/5D/8w-high-APEX-dapi.tif]

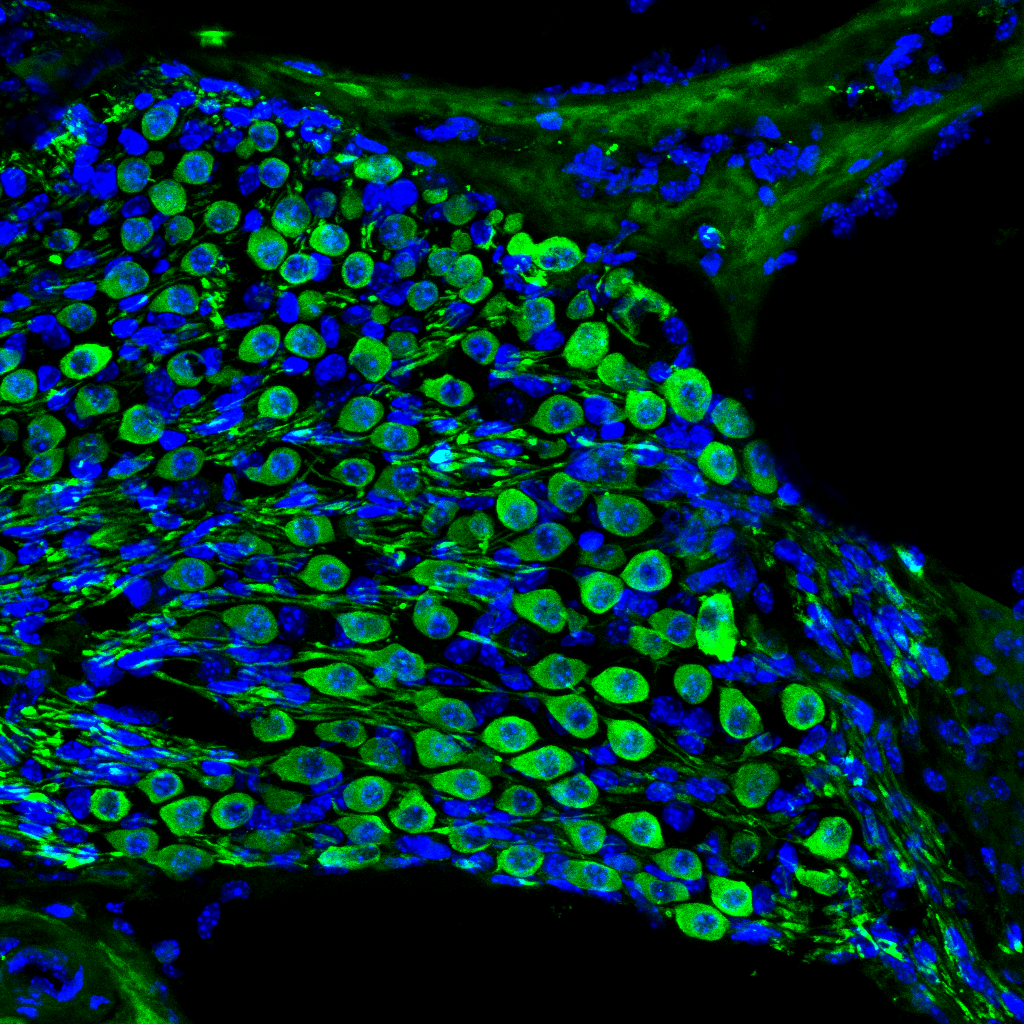

Supplement: Supplementary file 13 — Source data Fig. 5 [file 44321_2026_433_MOESM13_ESM.zip › Figure 5/5D/8w-high-APEX-merge.tif]

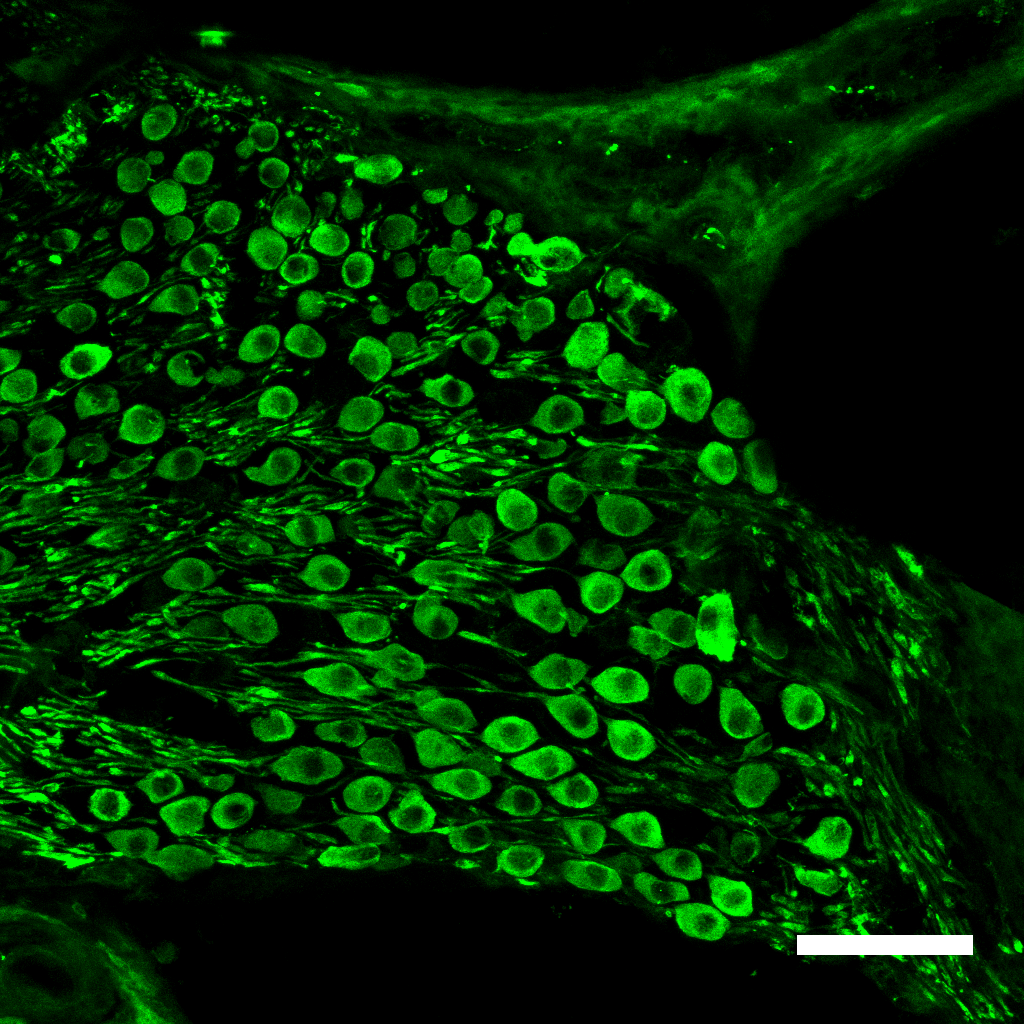

Supplement: Supplementary file 13 — Source data Fig. 5 [file 44321_2026_433_MOESM13_ESM.zip › Figure 5/5D/8w-high-APEX-tuj1.tif]

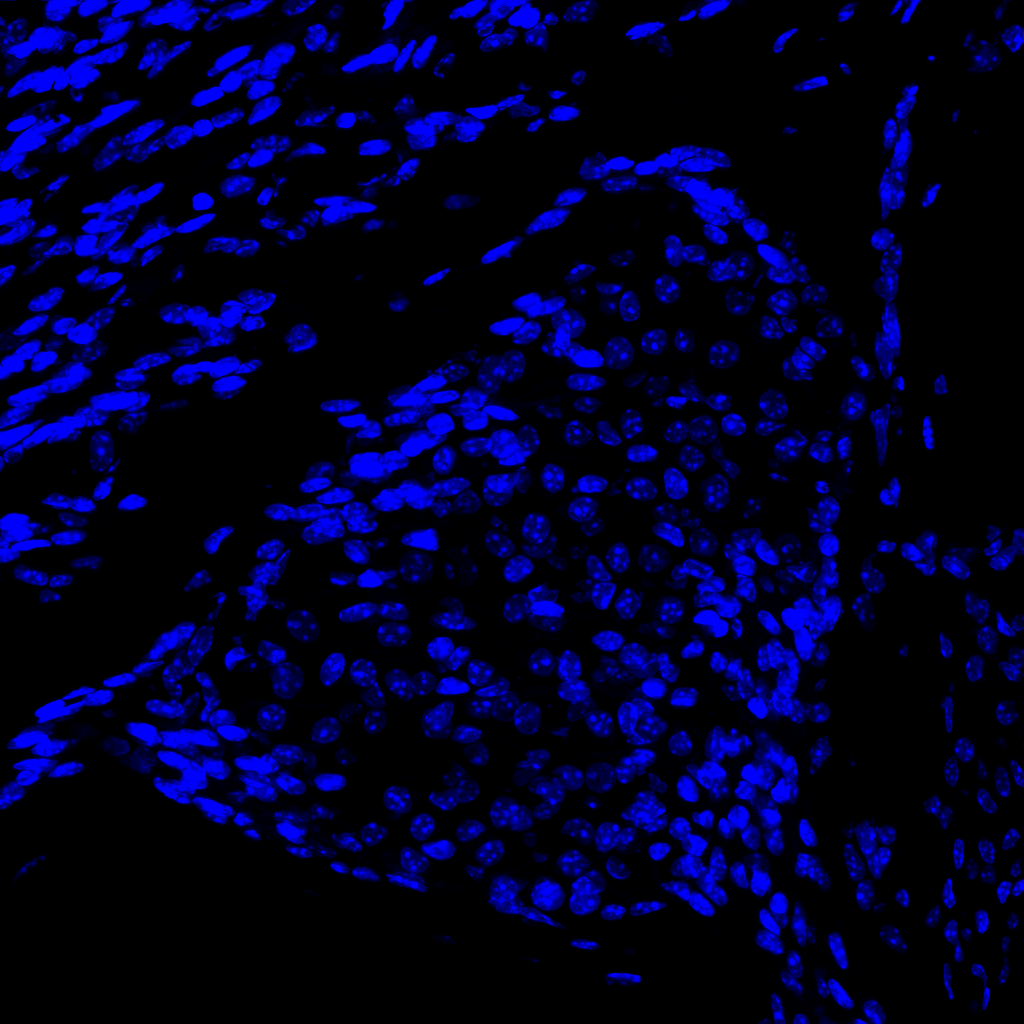

Supplement: Supplementary file 13 — Source data Fig. 5 [file 44321_2026_433_MOESM13_ESM.zip › Figure 5/5D/8w-high-MIDDLE-dapi.tif]

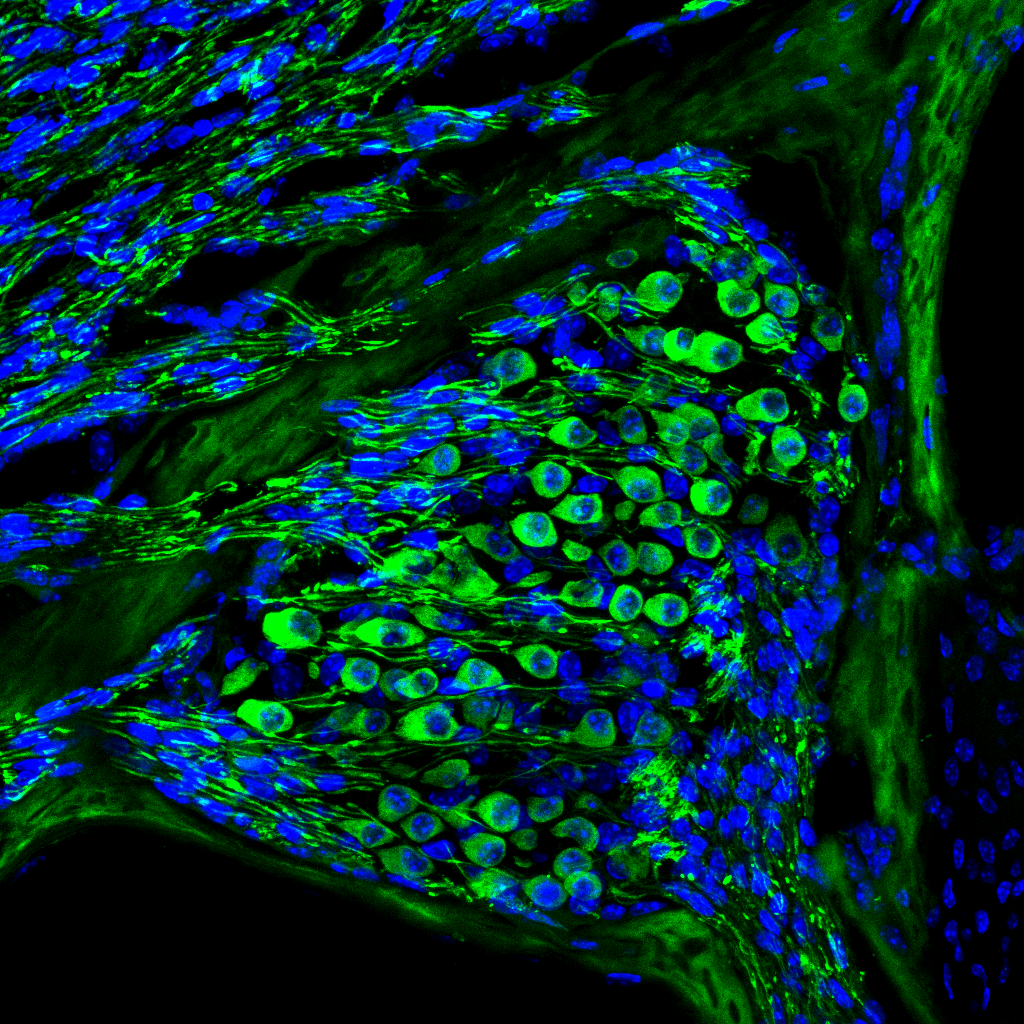

Supplement: Supplementary file 13 — Source data Fig. 5 [file 44321_2026_433_MOESM13_ESM.zip › Figure 5/5D/8w-high-MIDDLE-merge.tif]

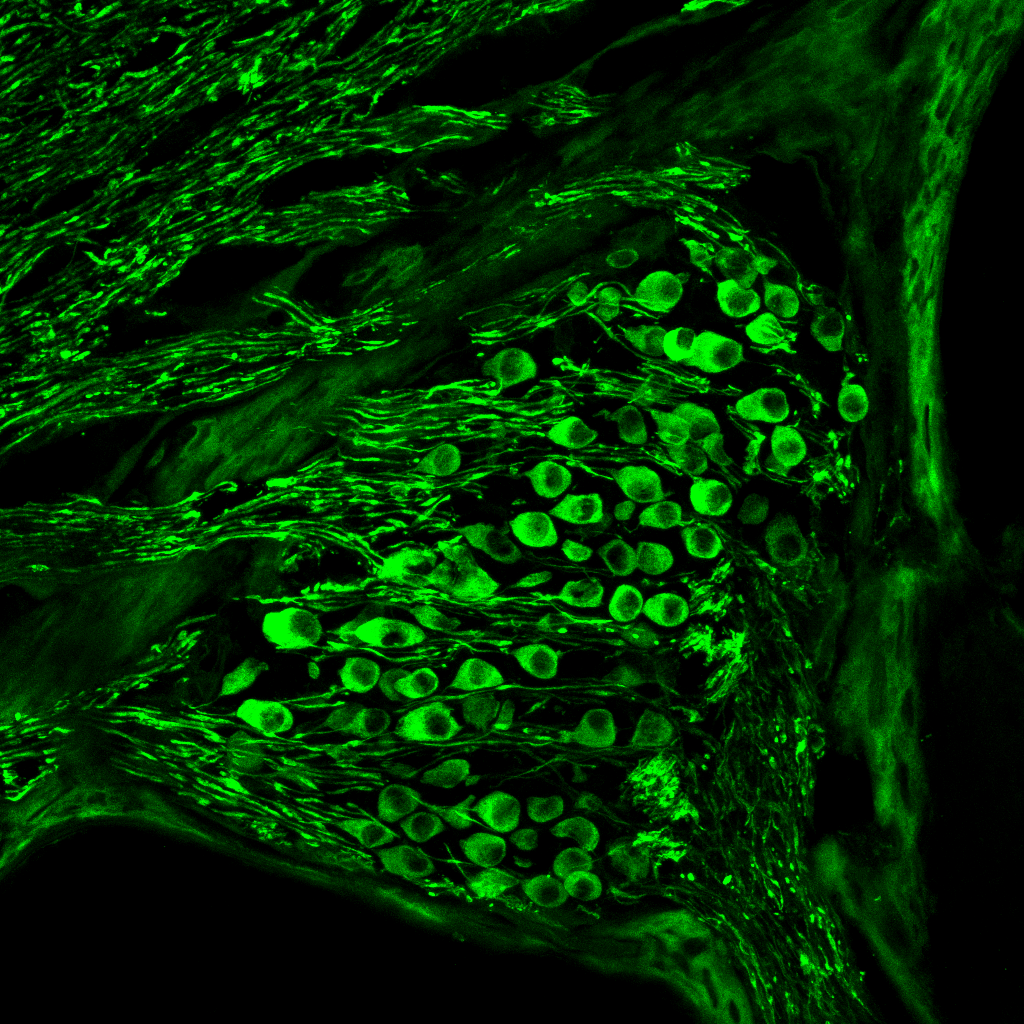

Supplement: Supplementary file 13 — Source data Fig. 5 [file 44321_2026_433_MOESM13_ESM.zip › Figure 5/5D/8w-high-MIDDLE-tuj1.tif]

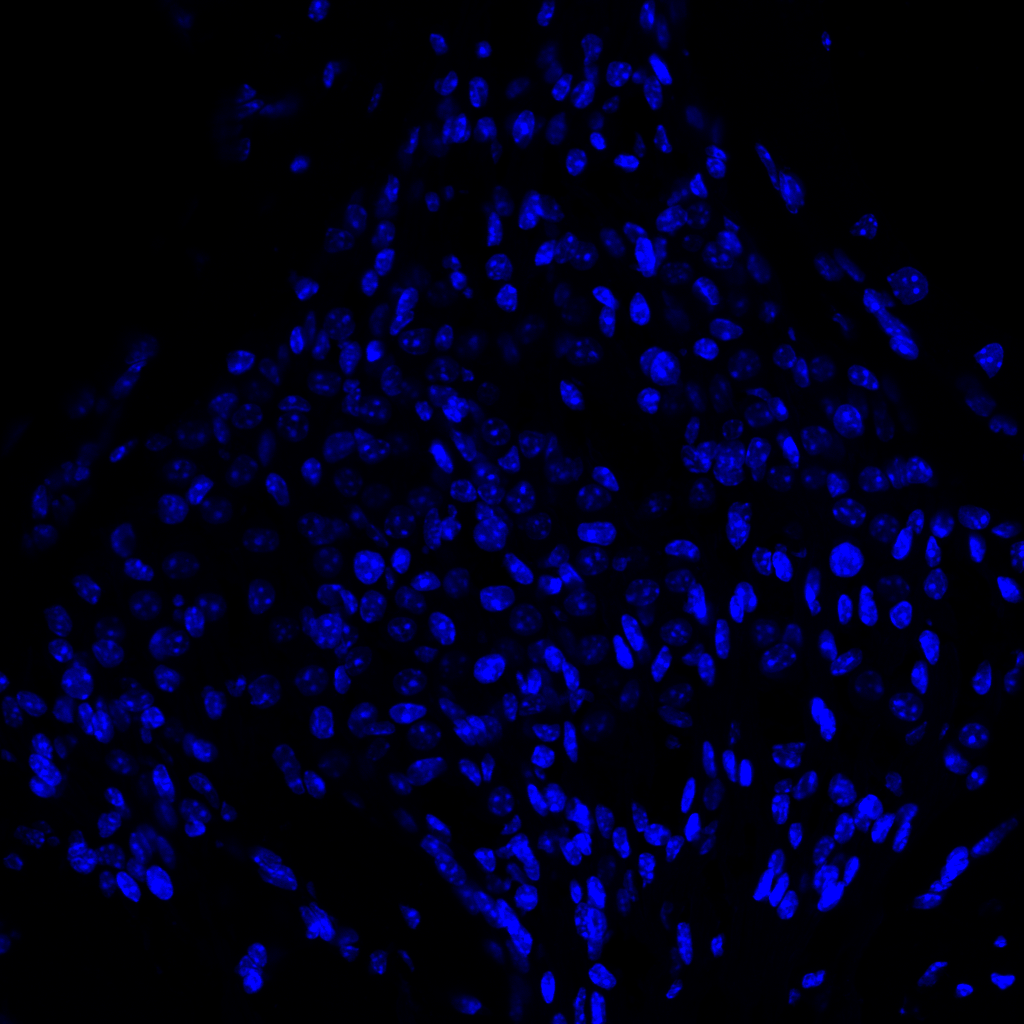

Supplement: Supplementary file 13 — Source data Fig. 5 [file 44321_2026_433_MOESM13_ESM.zip › Figure 5/5D/8w-high-base-dapi.tif]

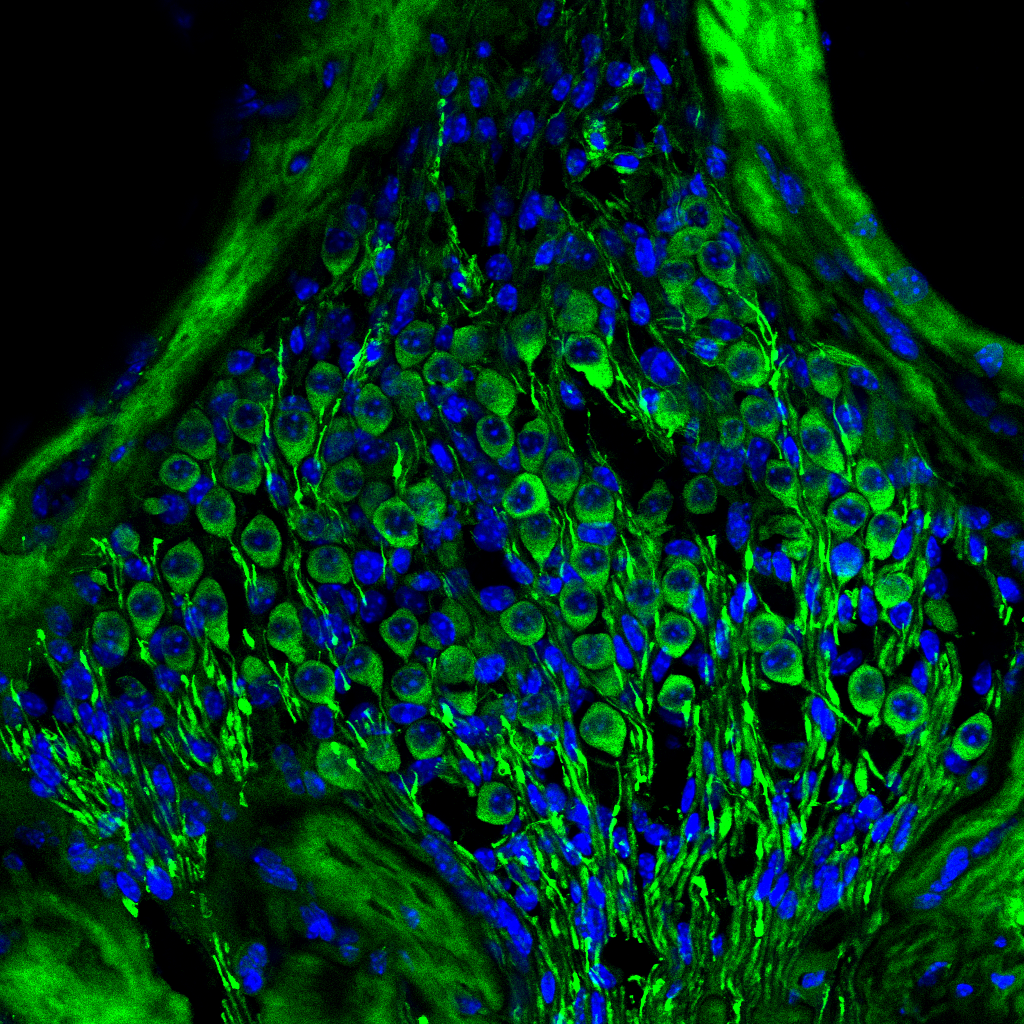

Supplement: Supplementary file 13 — Source data Fig. 5 [file 44321_2026_433_MOESM13_ESM.zip › Figure 5/5D/8w-high-base-merge.tif]

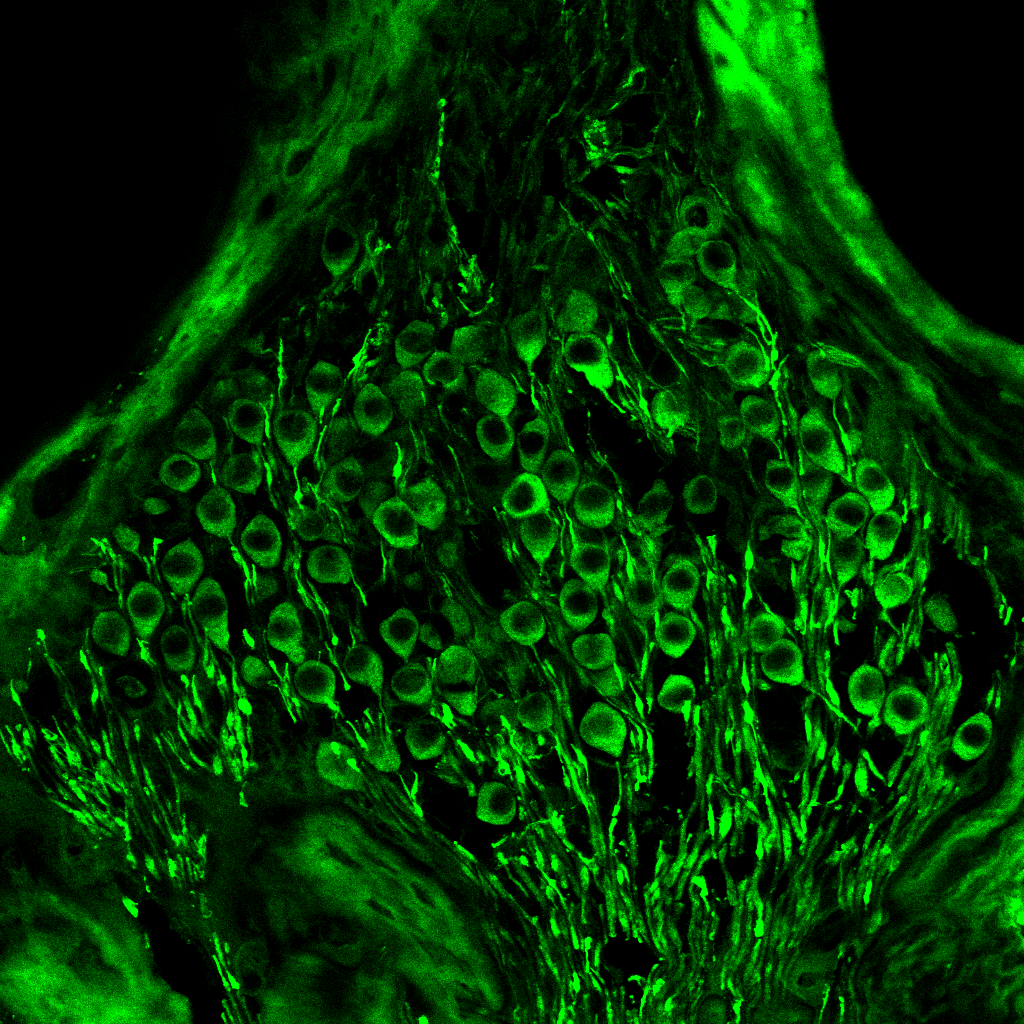

Supplement: Supplementary file 13 — Source data Fig. 5 [file 44321_2026_433_MOESM13_ESM.zip › Figure 5/5D/8w-high-base-tuj1.tif]

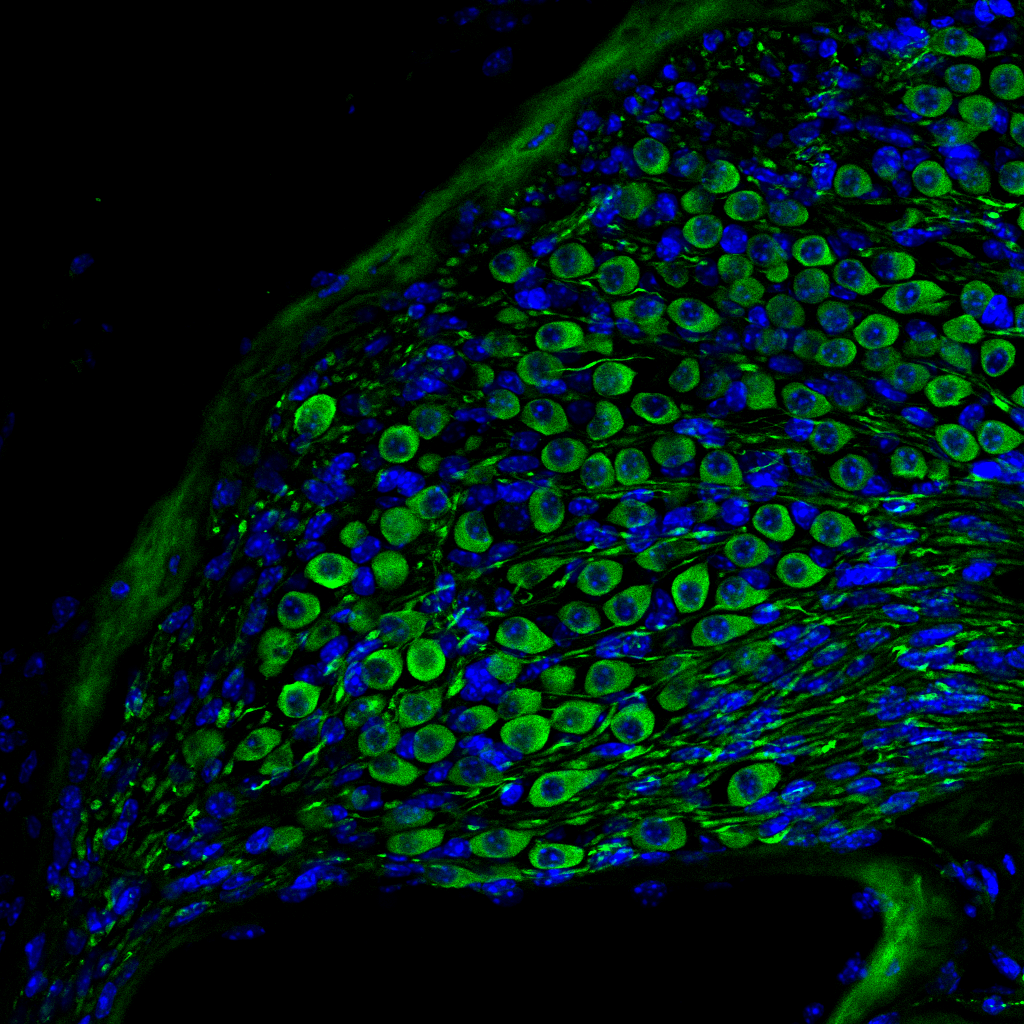

Supplement: Supplementary file 13 — Source data Fig. 5 [file 44321_2026_433_MOESM13_ESM.zip › Figure 5/5D/8w-low-aepx-merge.tif]

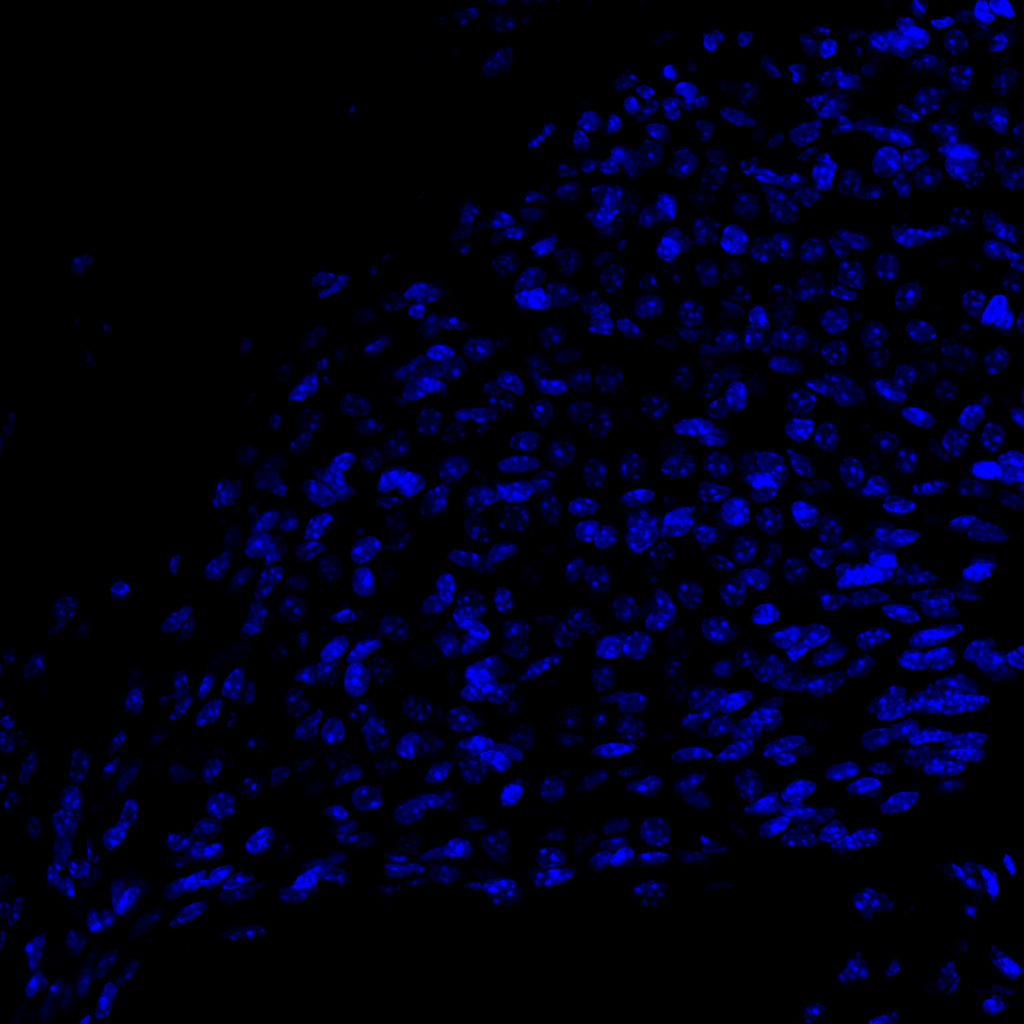

Supplement: Supplementary file 13 — Source data Fig. 5 [file 44321_2026_433_MOESM13_ESM.zip › Figure 5/5D/8w-low-apex-DAPI.tif]

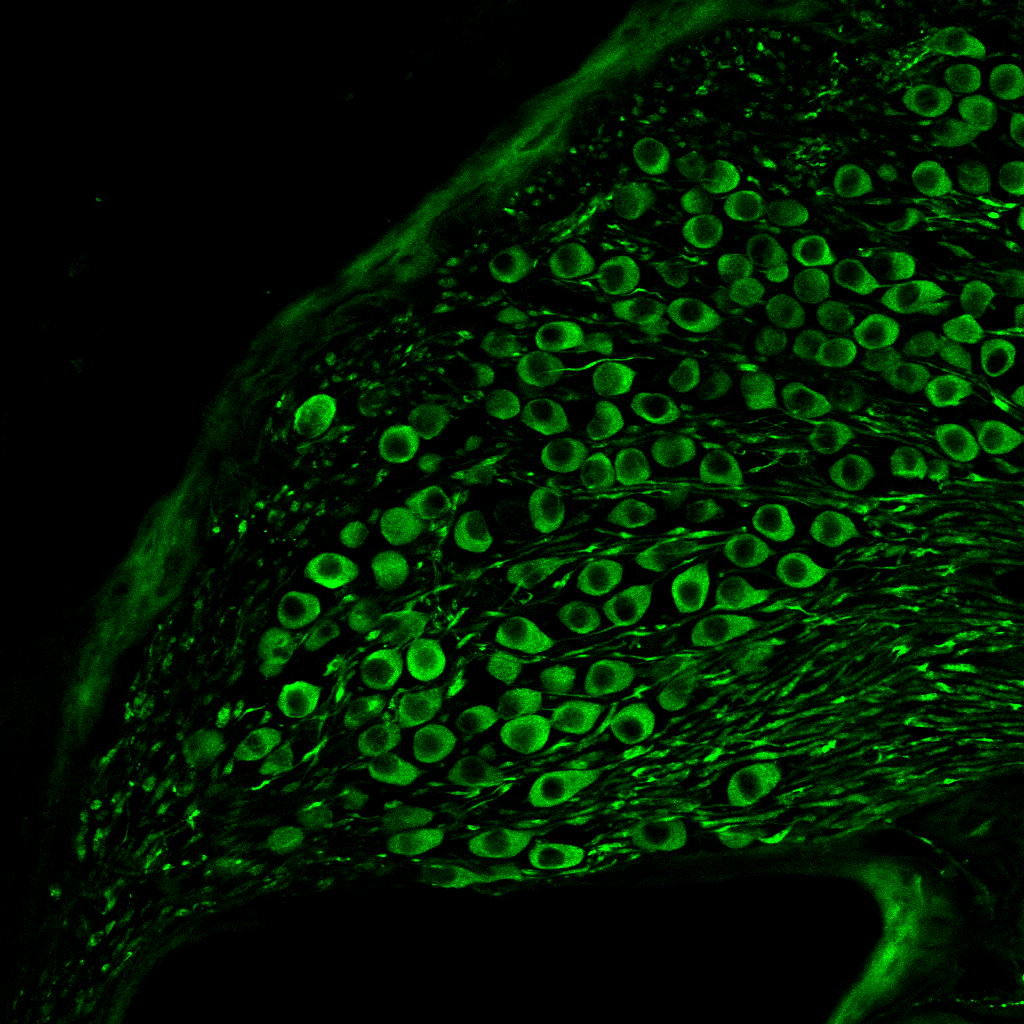

Supplement: Supplementary file 13 — Source data Fig. 5 [file 44321_2026_433_MOESM13_ESM.zip › Figure 5/5D/8w-low-apex-TUJ1.tif]

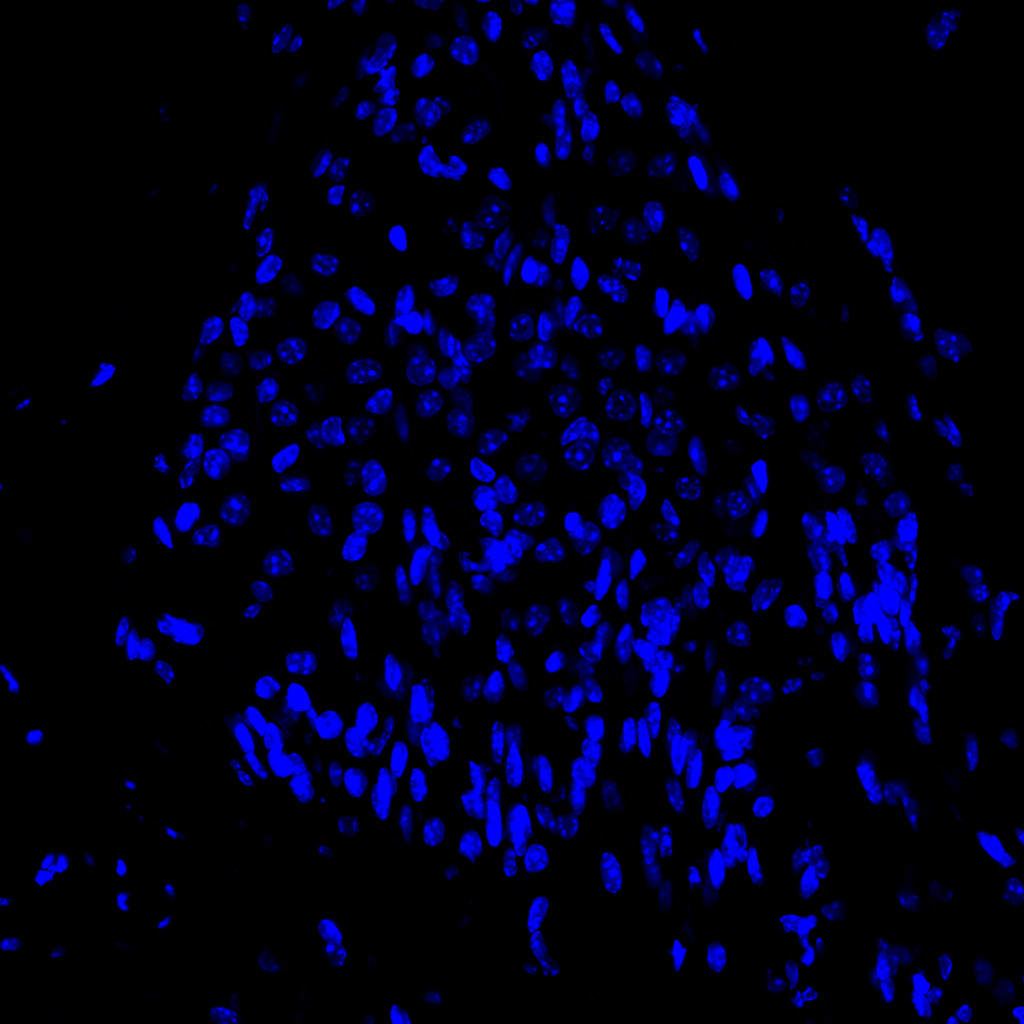

Supplement: Supplementary file 13 — Source data Fig. 5 [file 44321_2026_433_MOESM13_ESM.zip › Figure 5/5D/8w-low-base-DAPI.tif]

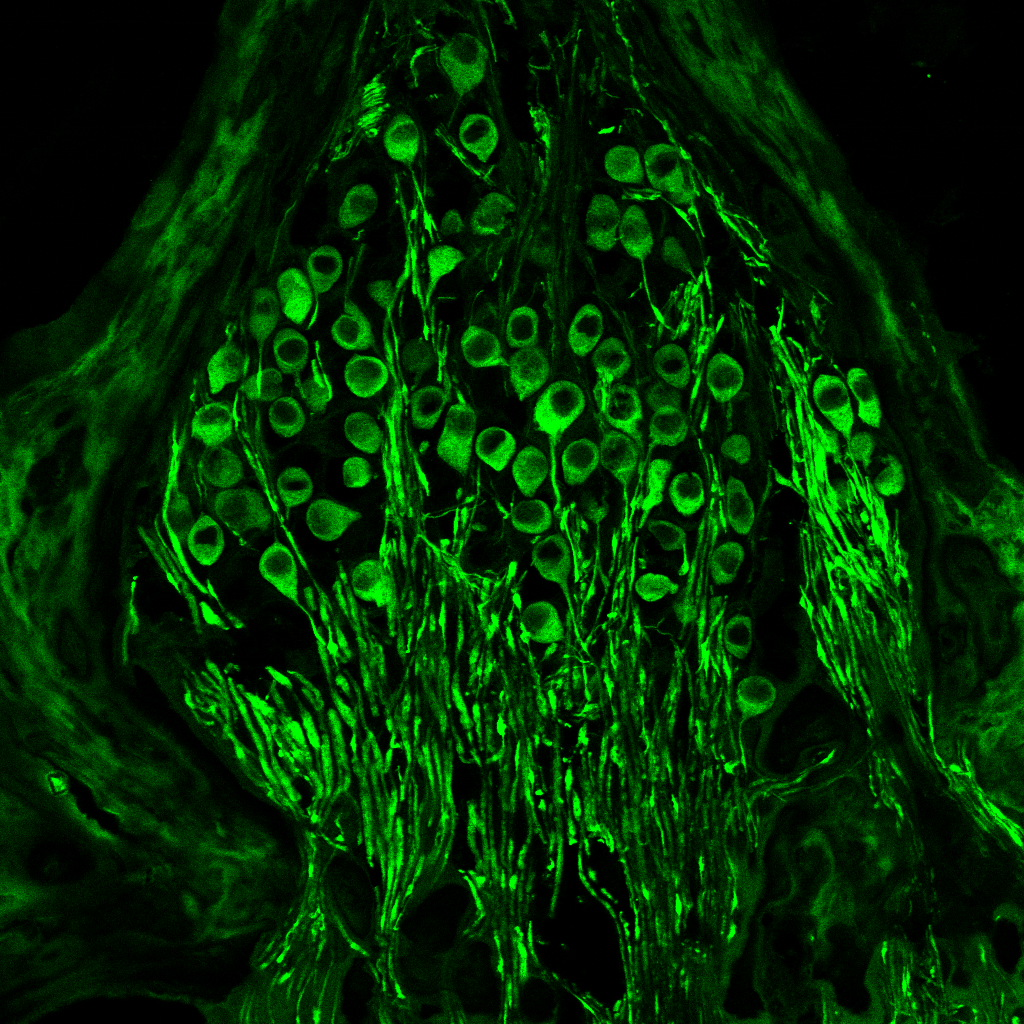

Supplement: Supplementary file 13 — Source data Fig. 5 [file 44321_2026_433_MOESM13_ESM.zip › Figure 5/5D/8w-low-base-TUJ1.tif]

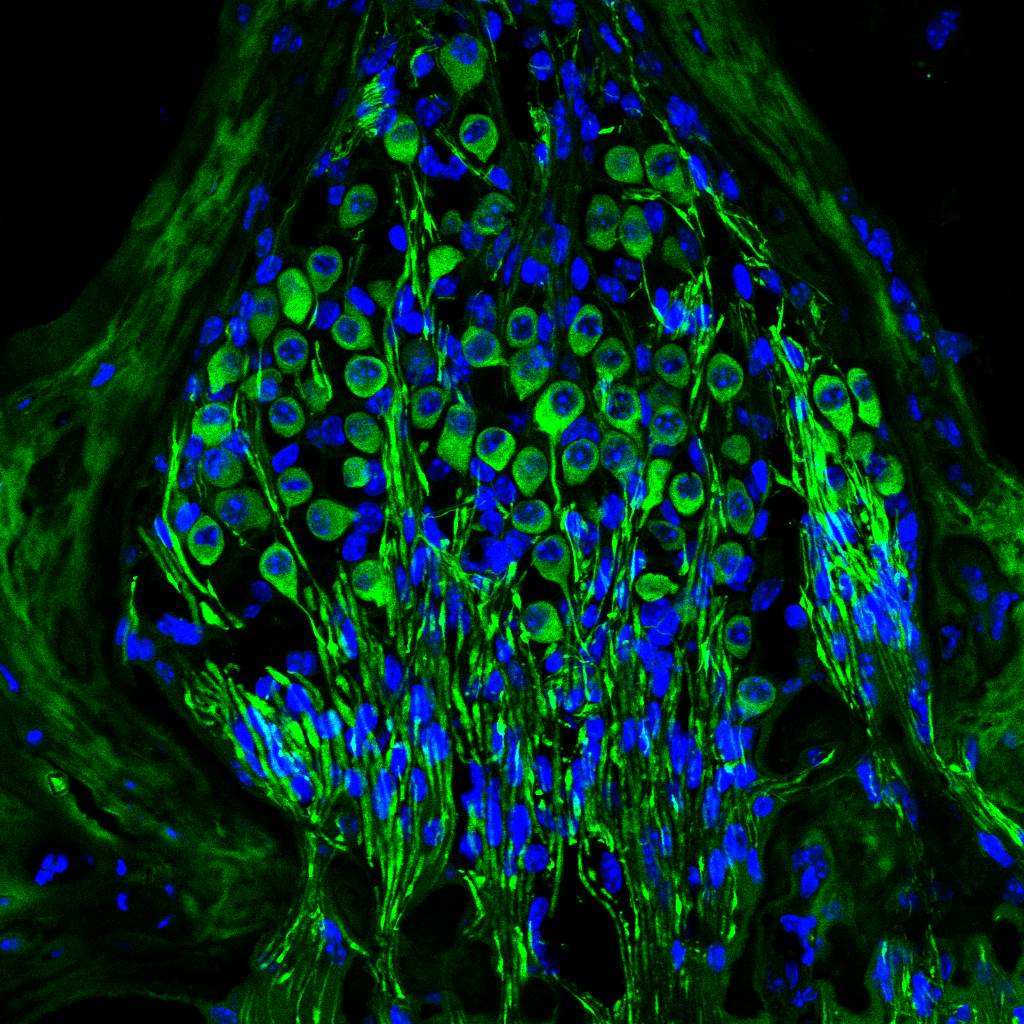

Supplement: Supplementary file 13 — Source data Fig. 5 [file 44321_2026_433_MOESM13_ESM.zip › Figure 5/5D/8w-low-base-merge.tif]

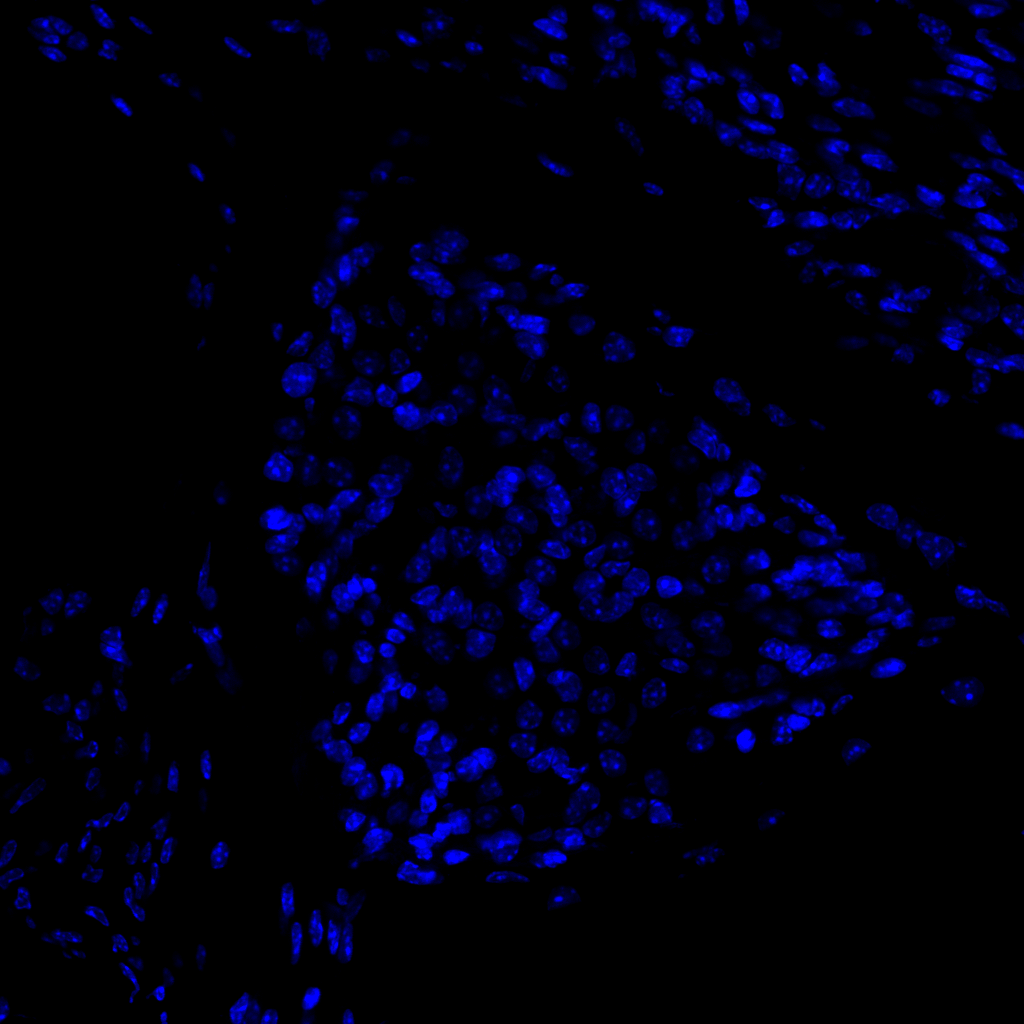

Supplement: Supplementary file 13 — Source data Fig. 5 [file 44321_2026_433_MOESM13_ESM.zip › Figure 5/5D/8w-low-mid-DAPI.tif]

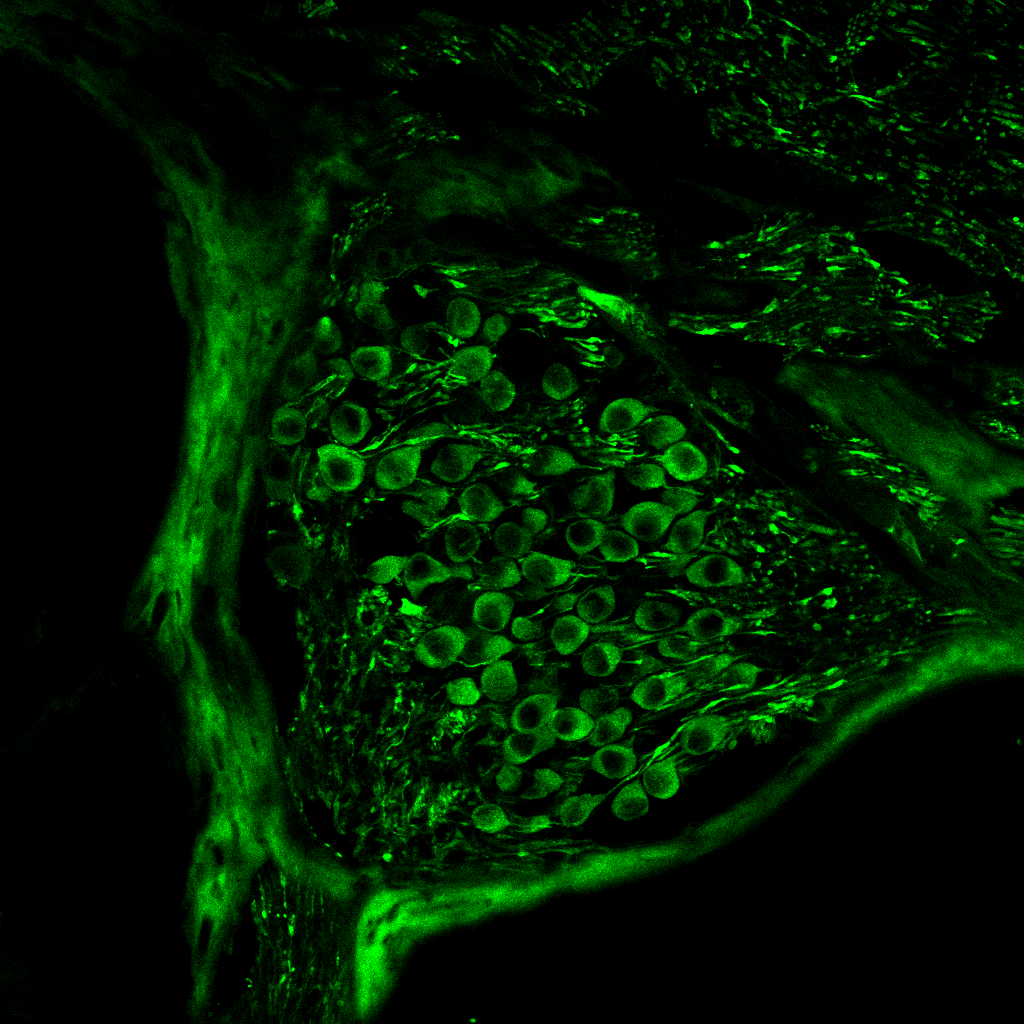

Supplement: Supplementary file 13 — Source data Fig. 5 [file 44321_2026_433_MOESM13_ESM.zip › Figure 5/5D/8w-low-mid-TUJ1.tif]

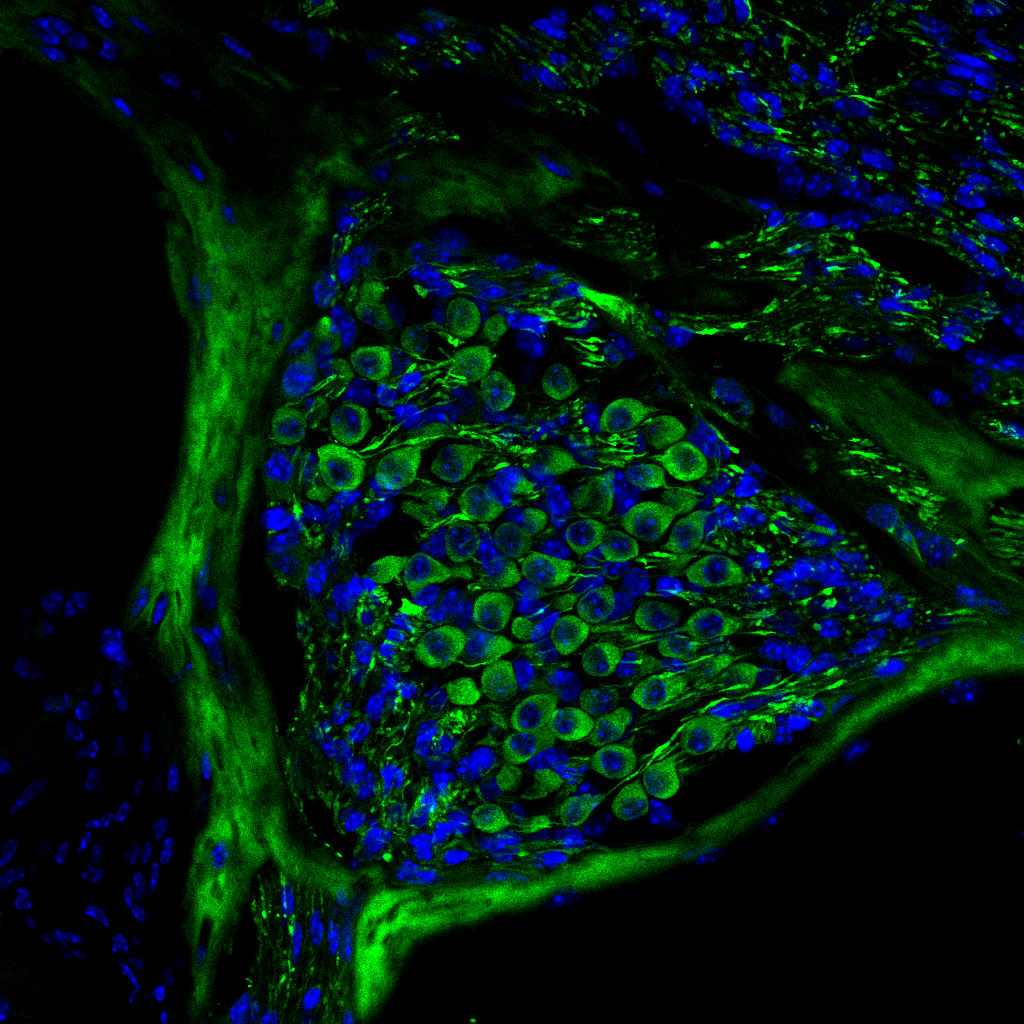

Supplement: Supplementary file 13 — Source data Fig. 5 [file 44321_2026_433_MOESM13_ESM.zip › Figure 5/5D/8w-low-mid-merge.tif]

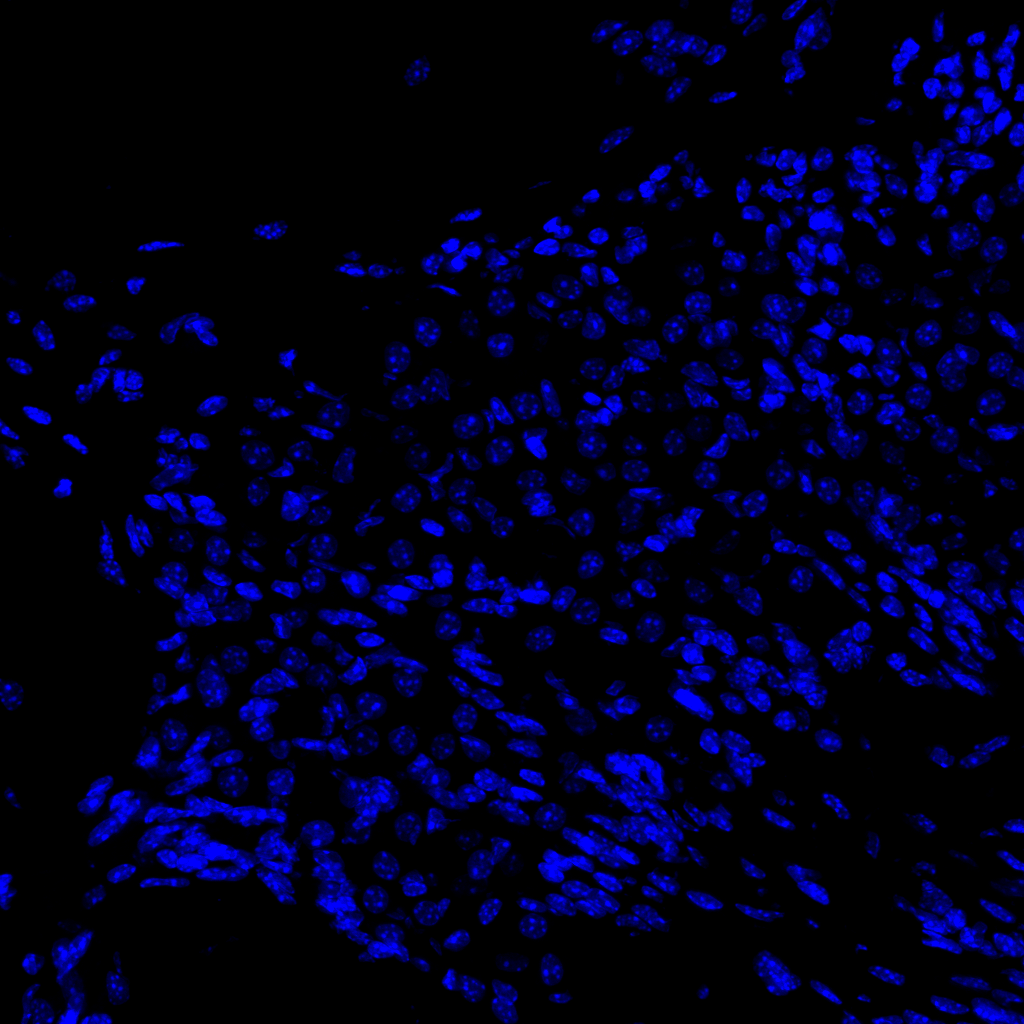

Supplement: Supplementary file 13 — Source data Fig. 5 [file 44321_2026_433_MOESM13_ESM.zip › Figure 5/5D/8w-wt-APEX-dapi.tif]

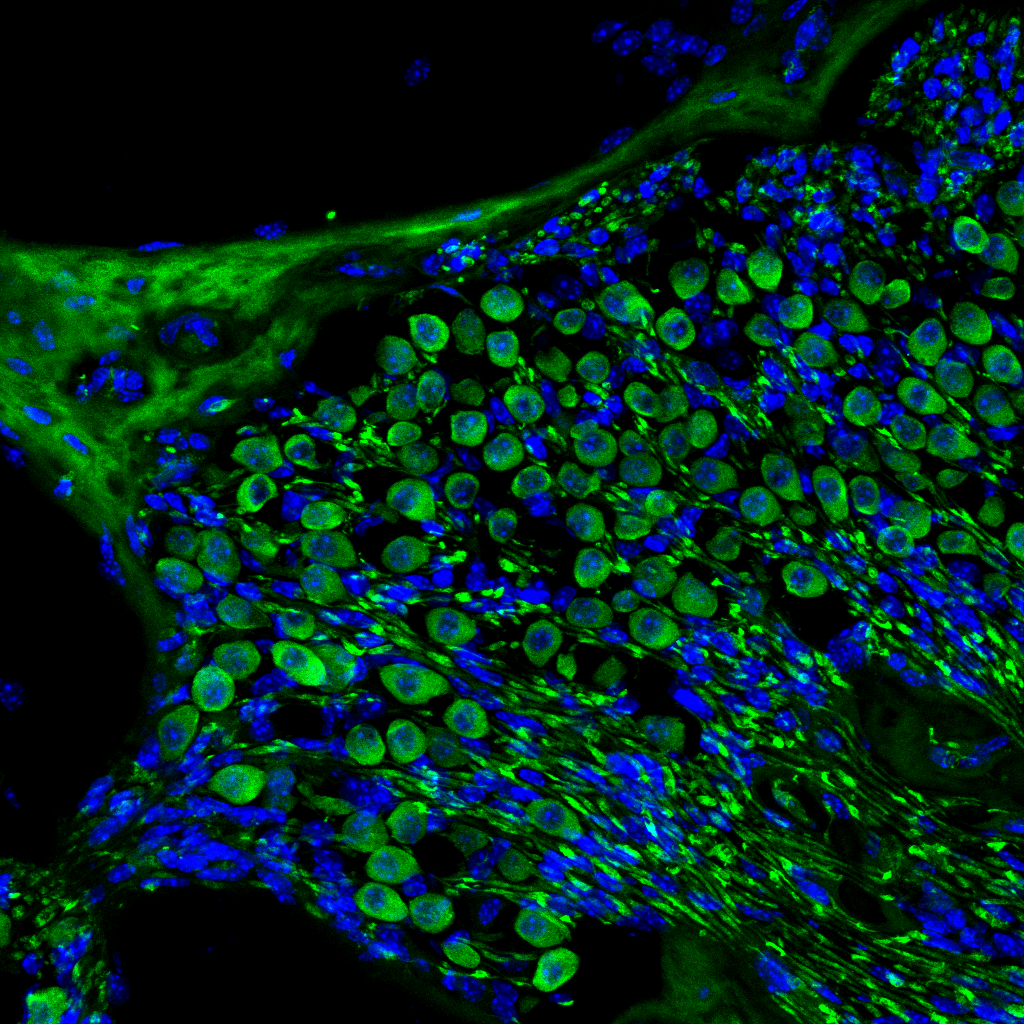

Supplement: Supplementary file 13 — Source data Fig. 5 [file 44321_2026_433_MOESM13_ESM.zip › Figure 5/5D/8w-wt-APEX-merge.tif]

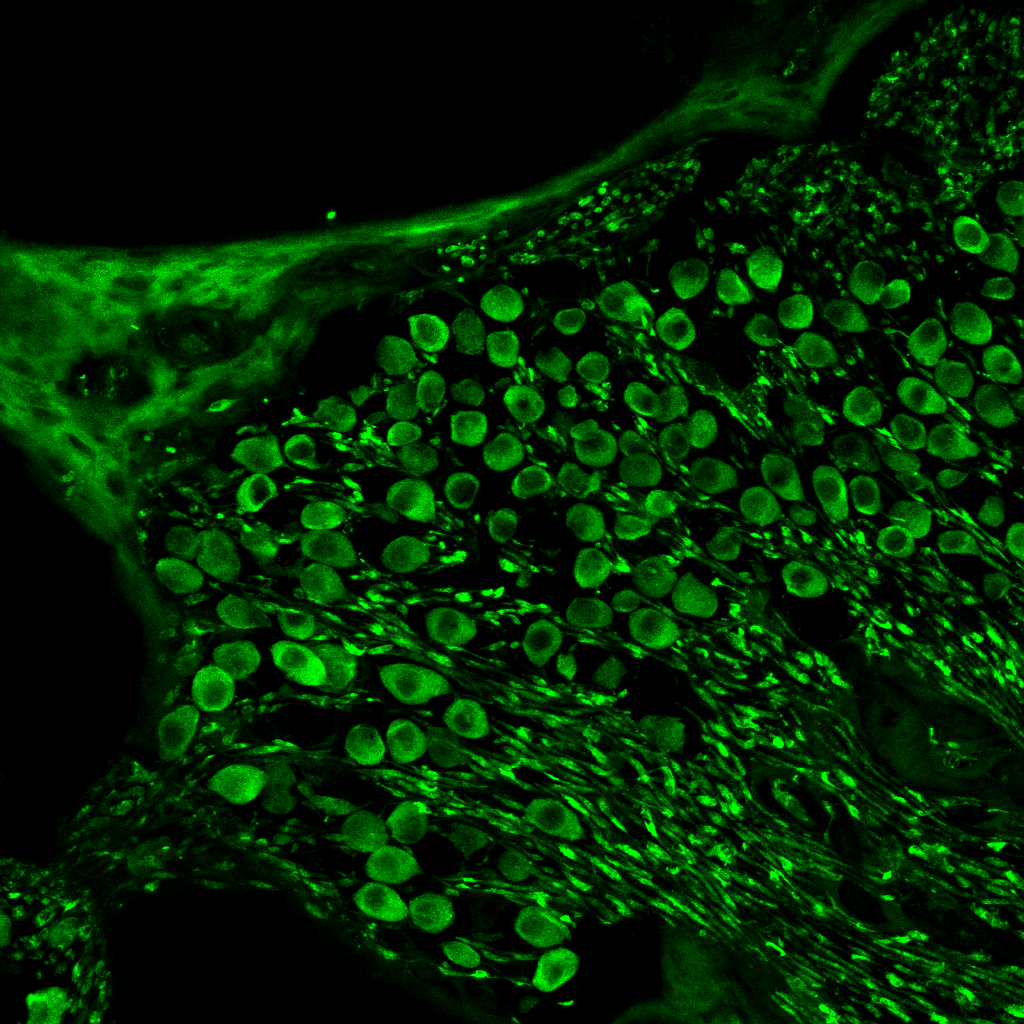

Supplement: Supplementary file 13 — Source data Fig. 5 [file 44321_2026_433_MOESM13_ESM.zip › Figure 5/5D/8w-wt-APEX-tuj1.tif]

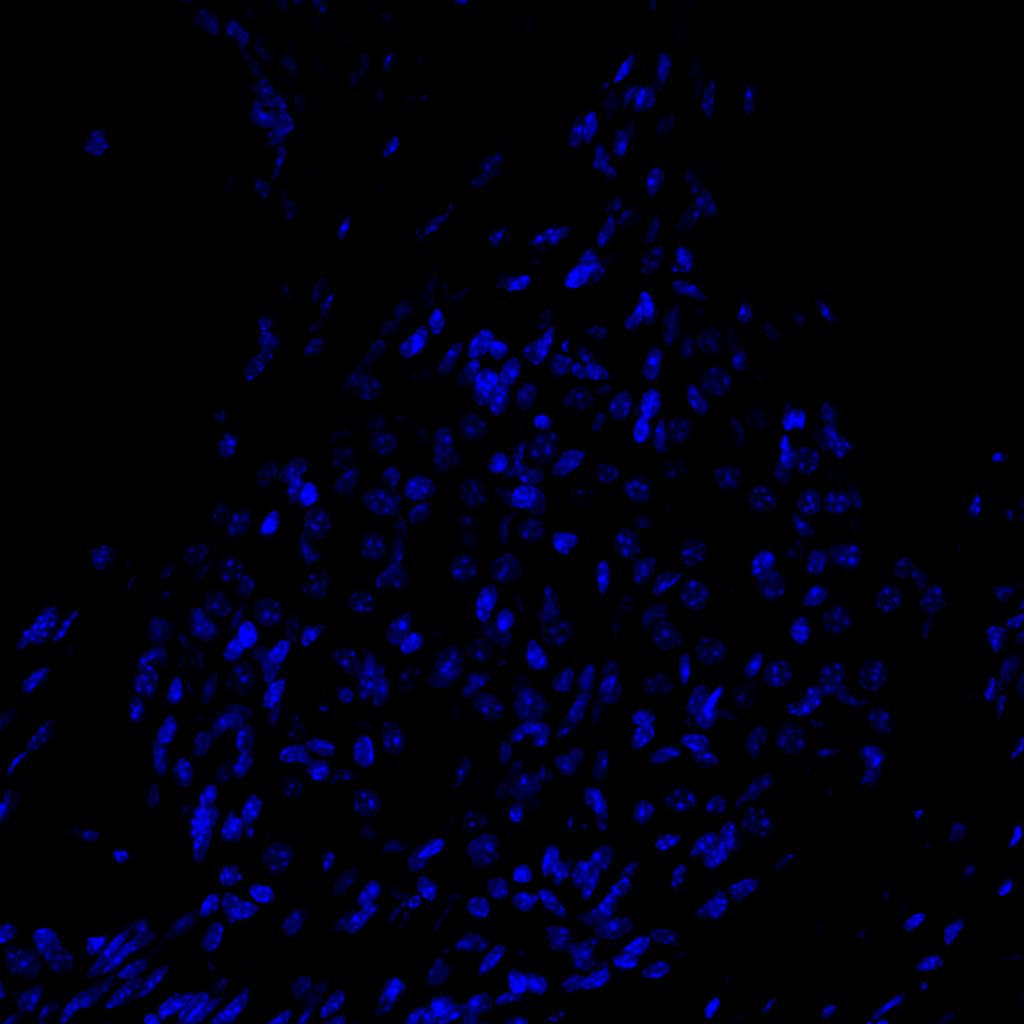

Supplement: Supplementary file 13 — Source data Fig. 5 [file 44321_2026_433_MOESM13_ESM.zip › Figure 5/5D/8w-wt-BASE-dapi.tif]

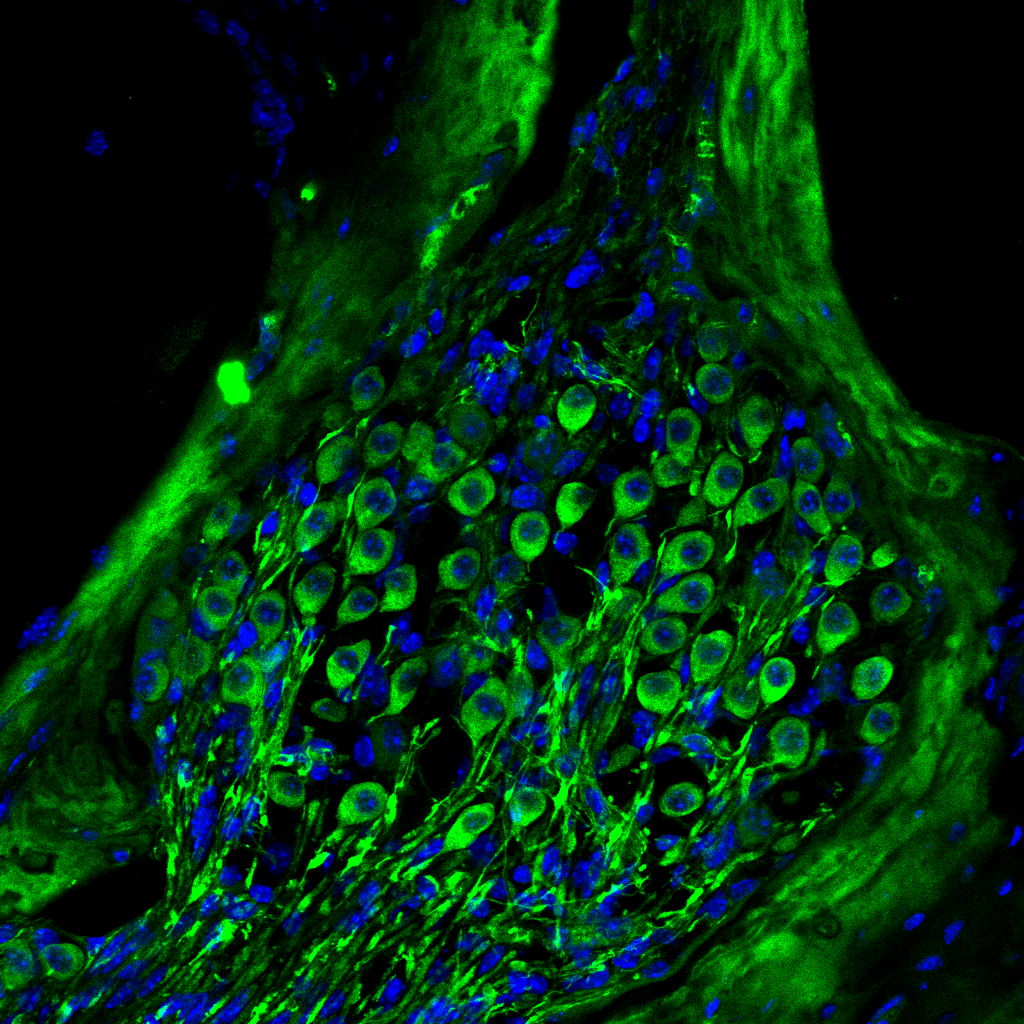

Supplement: Supplementary file 13 — Source data Fig. 5 [file 44321_2026_433_MOESM13_ESM.zip › Figure 5/5D/8w-wt-BASE-merge.tif]

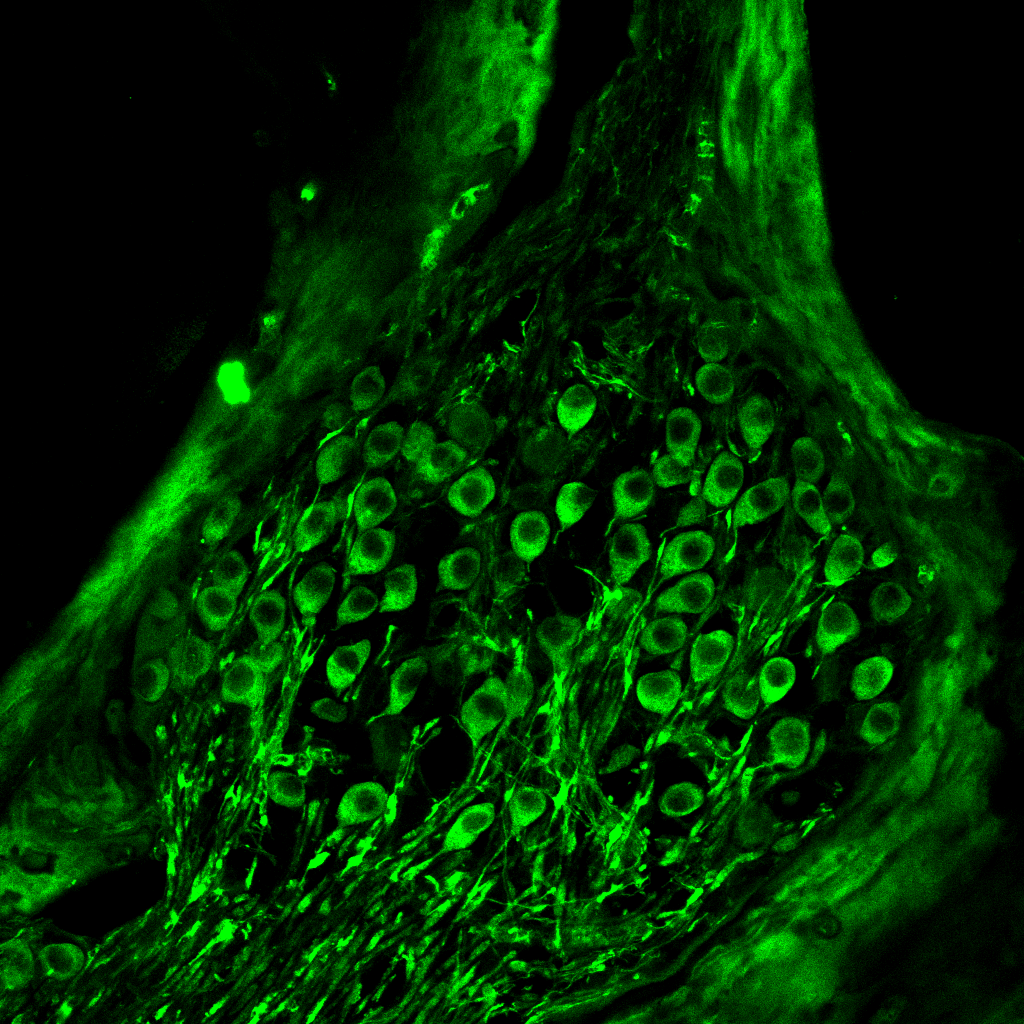

Supplement: Supplementary file 13 — Source data Fig. 5 [file 44321_2026_433_MOESM13_ESM.zip › Figure 5/5D/8w-wt-BASE-tuj1.tif]

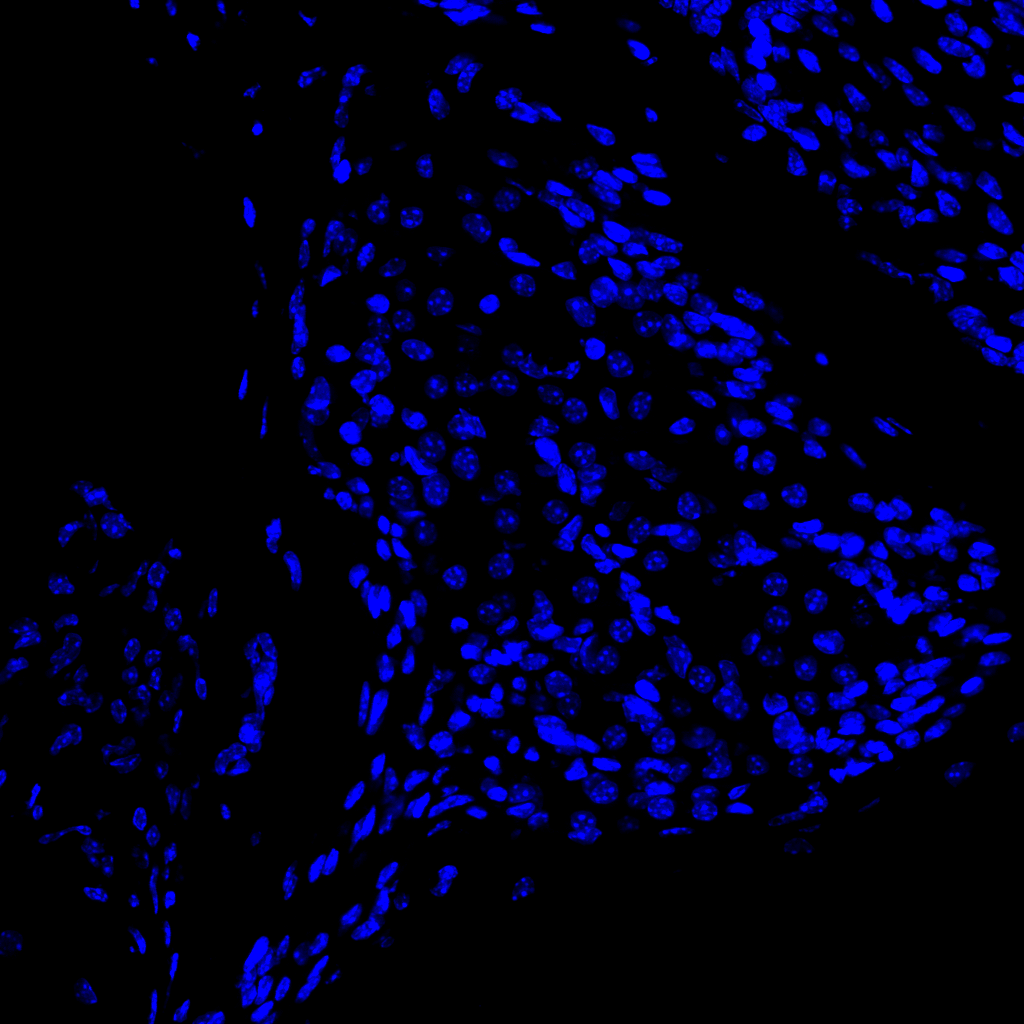

Supplement: Supplementary file 13 — Source data Fig. 5 [file 44321_2026_433_MOESM13_ESM.zip › Figure 5/5D/8w-wt-mid-dapi.tif]

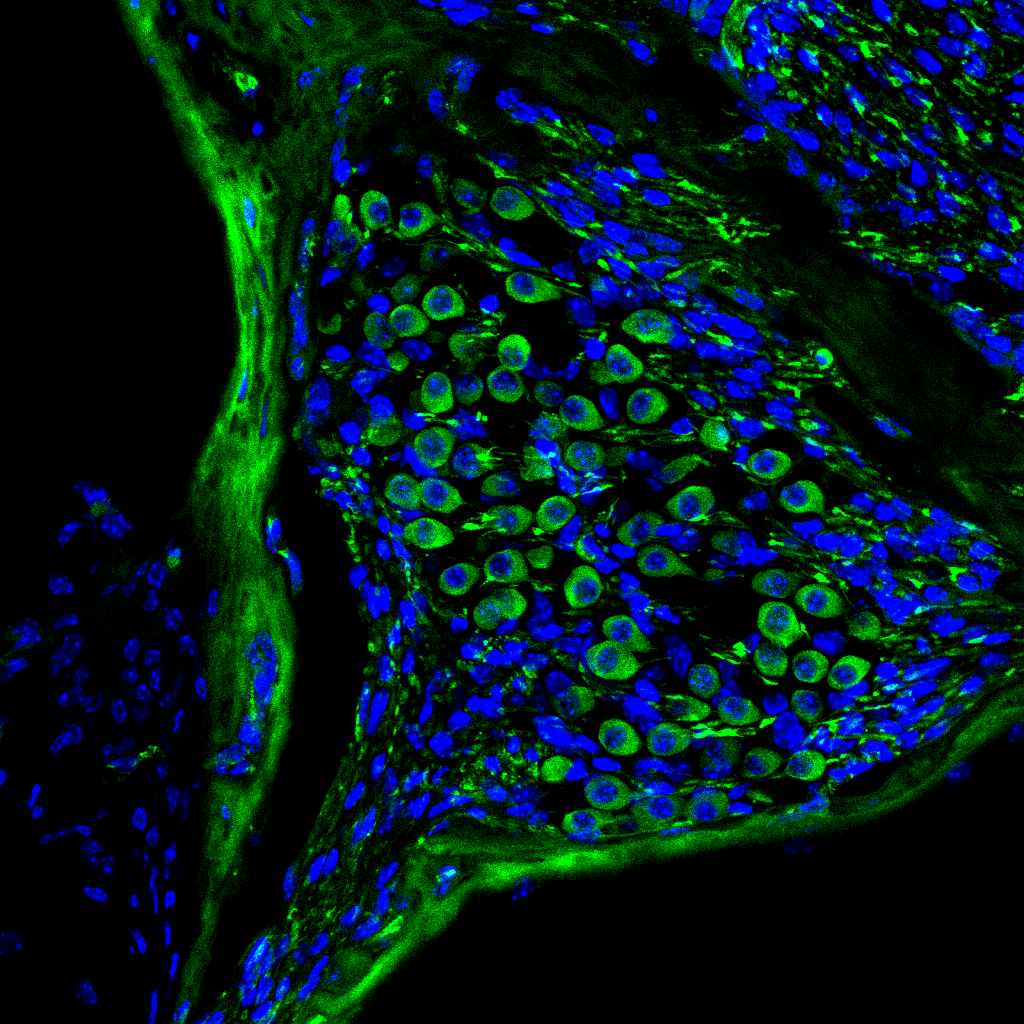

Supplement: Supplementary file 13 — Source data Fig. 5 [file 44321_2026_433_MOESM13_ESM.zip › Figure 5/5D/8w-wt-mid-merge.tif]

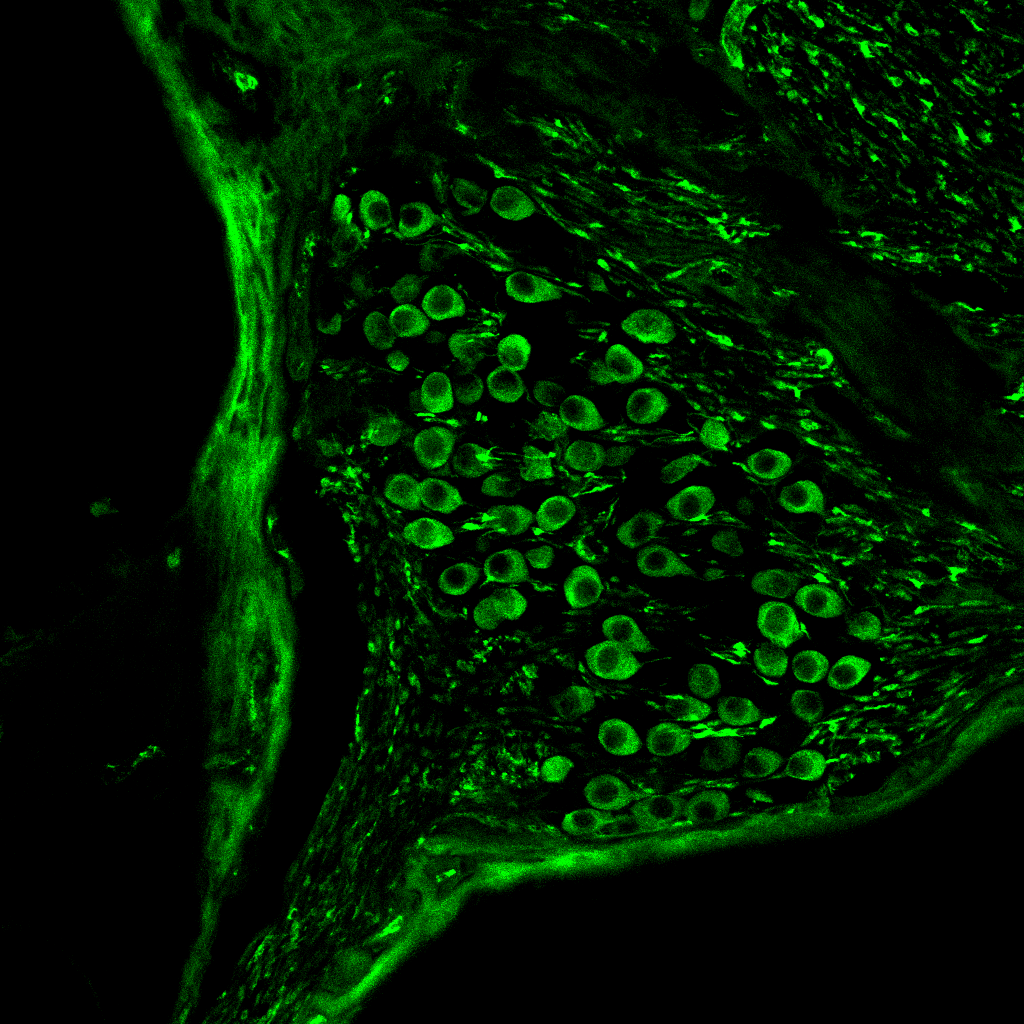

Supplement: Supplementary file 13 — Source data Fig. 5 [file 44321_2026_433_MOESM13_ESM.zip › Figure 5/5D/8w-wt-mid-tuj1.tif]

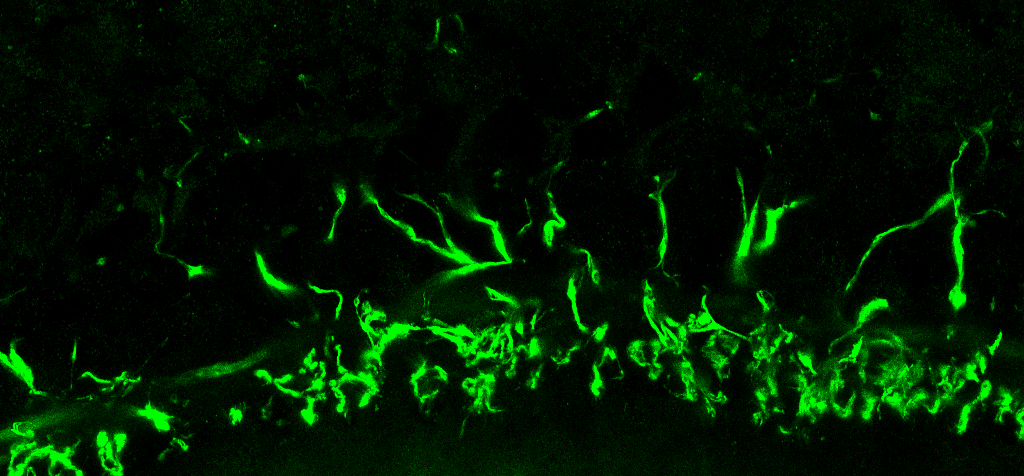

Supplement: Supplementary file 13 — Source data Fig. 5 [file 44321_2026_433_MOESM13_ESM.zip › Figure 5/5F/8w-ctrl-aepx-NF200.tif]

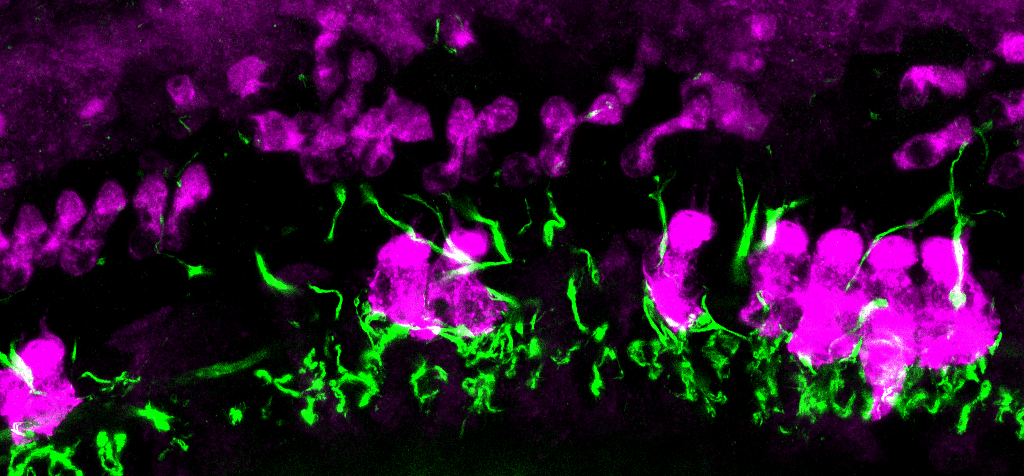

Supplement: Supplementary file 13 — Source data Fig. 5 [file 44321_2026_433_MOESM13_ESM.zip › Figure 5/5F/8w-ctrl-apex-merge.tif]

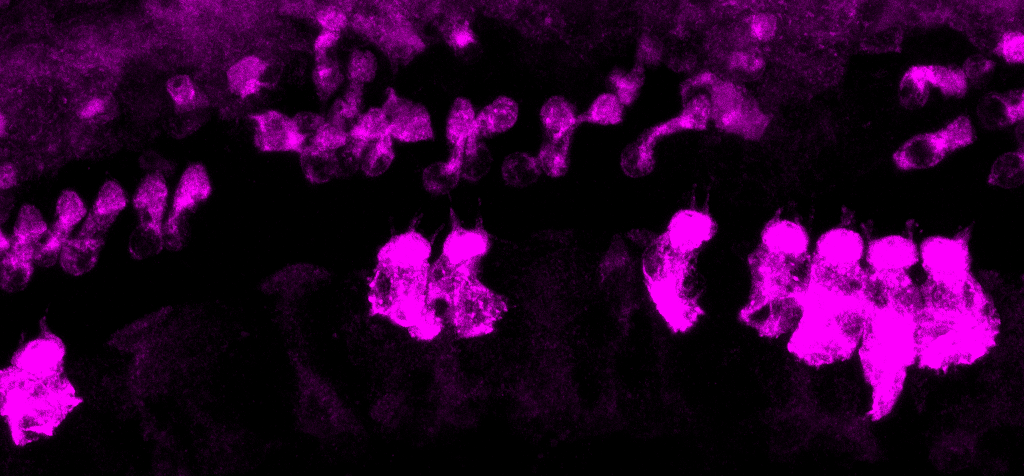

Supplement: Supplementary file 13 — Source data Fig. 5 [file 44321_2026_433_MOESM13_ESM.zip › Figure 5/5F/8w-ctrl-apex-myo.tif]

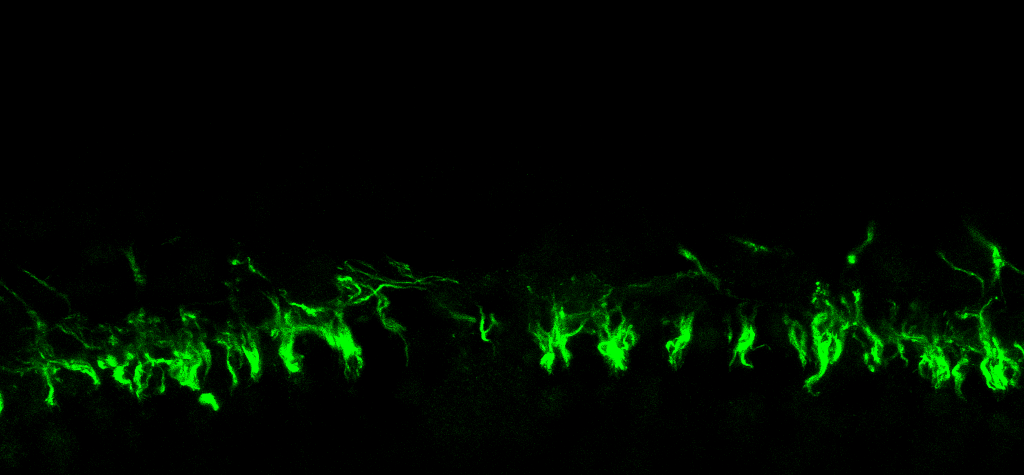

Supplement: Supplementary file 13 — Source data Fig. 5 [file 44321_2026_433_MOESM13_ESM.zip › Figure 5/5F/8w-ctrl-base-NF200.tif]

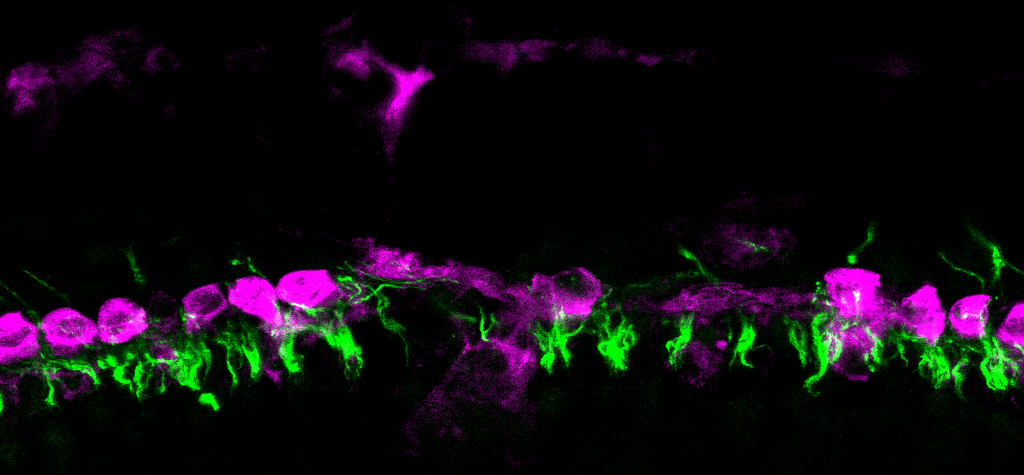

Supplement: Supplementary file 13 — Source data Fig. 5 [file 44321_2026_433_MOESM13_ESM.zip › Figure 5/5F/8w-ctrl-base-merge.tif]

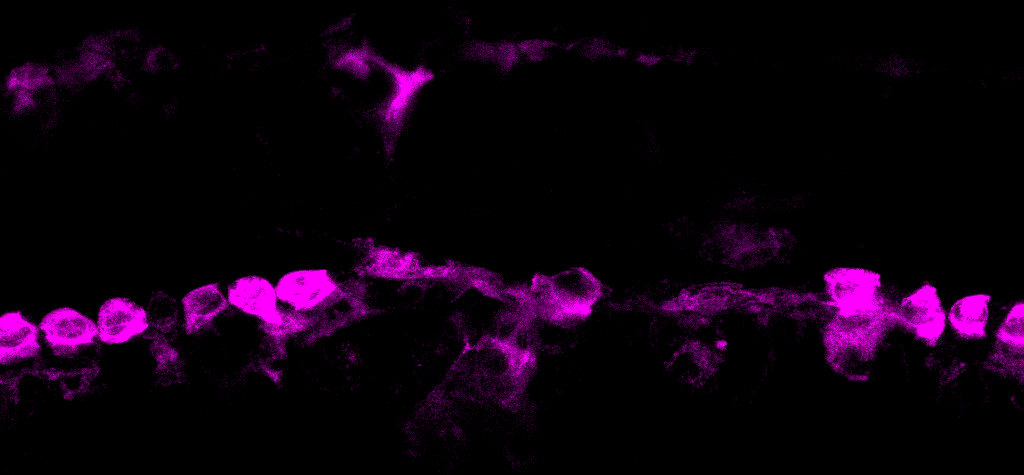

Supplement: Supplementary file 13 — Source data Fig. 5 [file 44321_2026_433_MOESM13_ESM.zip › Figure 5/5F/8w-ctrl-base-myo.tif]

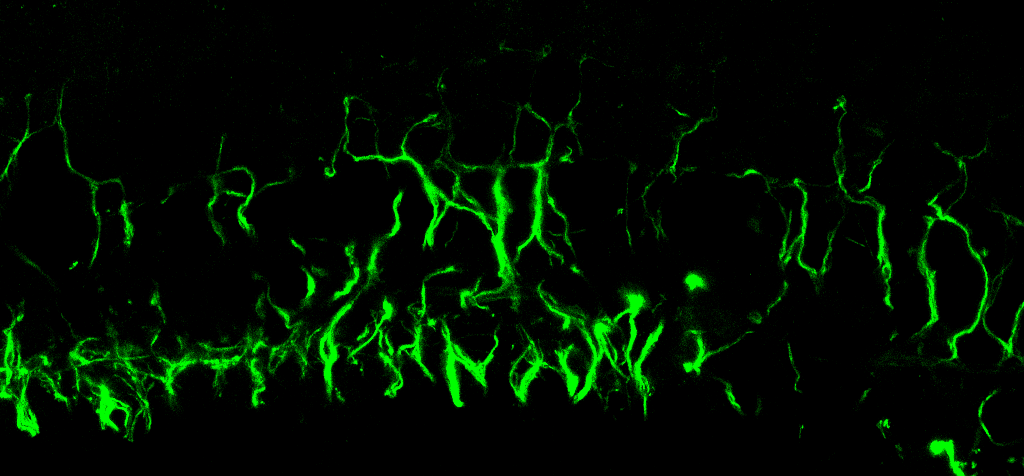

Supplement: Supplementary file 13 — Source data Fig. 5 [file 44321_2026_433_MOESM13_ESM.zip › Figure 5/5F/8w-ctrl-mid-NF200.tif]

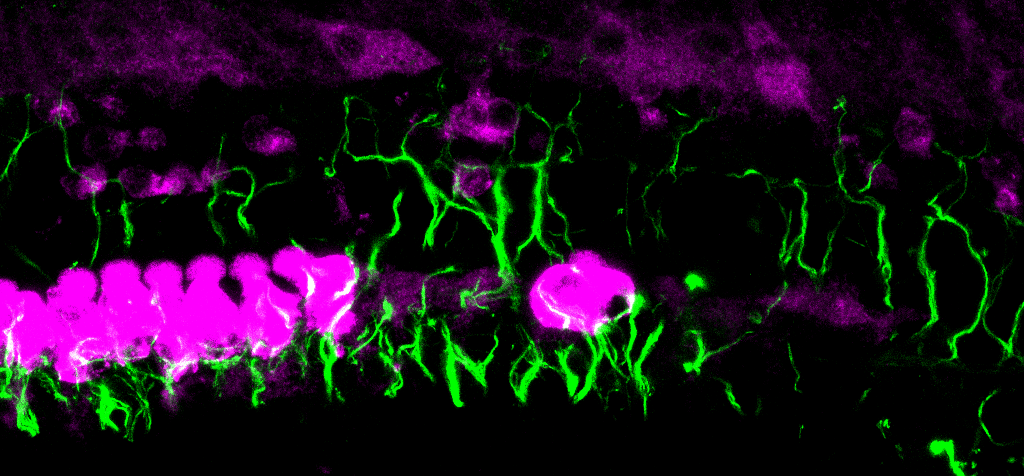

Supplement: Supplementary file 13 — Source data Fig. 5 [file 44321_2026_433_MOESM13_ESM.zip › Figure 5/5F/8w-ctrl-mid-merge.tif]
