# Supplementary figures and images for: Long-term restoration of auditory function in a DFNA2 mouse model by adenine base editing (part 2 of 2)
Source: EMBO Mol Med. 2026 May 20;18(6):2293–321. doi: 10.1038/s44321-026-00433-5 (PMC13270111; doi:10.1038/s44321-026-00433-5)

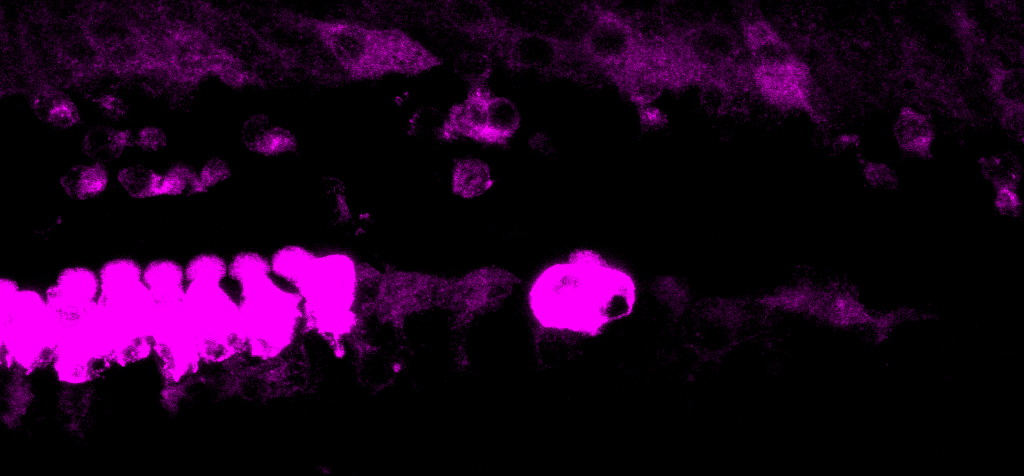

Supplement: Supplementary file 13 — Source data Fig. 5 [file 44321_2026_433_MOESM13_ESM.zip › Figure 5/5F/8w-ctrl-mid-myo.tif]

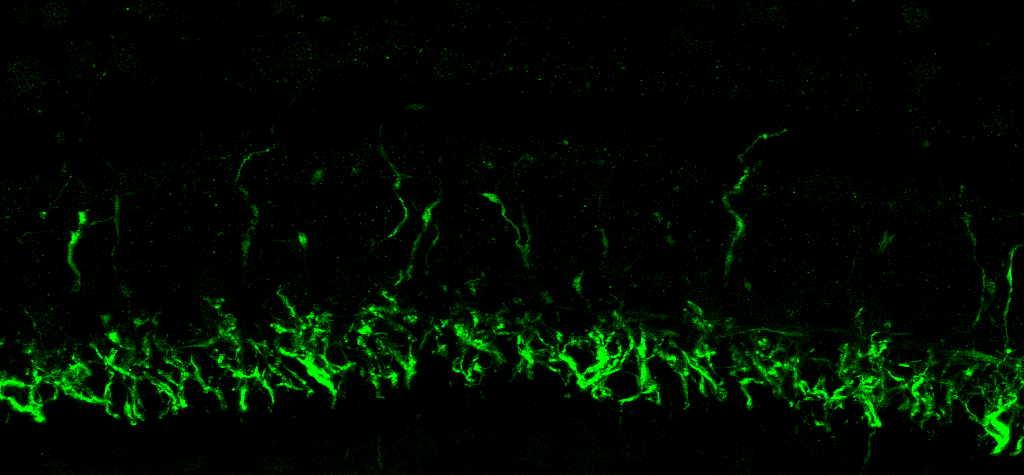

Supplement: Supplementary file 13 — Source data Fig. 5 [file 44321_2026_433_MOESM13_ESM.zip › Figure 5/5F/8w-high-apex-NF200.tif]

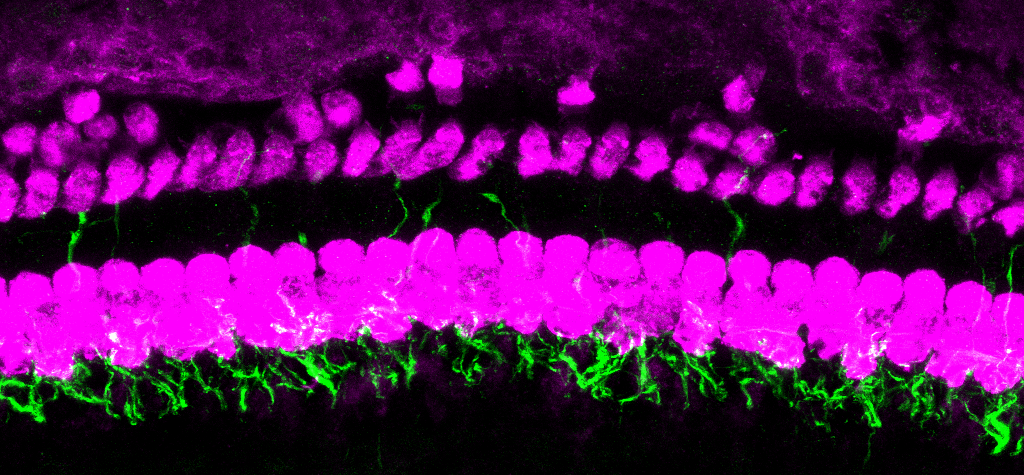

Supplement: Supplementary file 13 — Source data Fig. 5 [file 44321_2026_433_MOESM13_ESM.zip › Figure 5/5F/8w-high-apex-merge.tif]

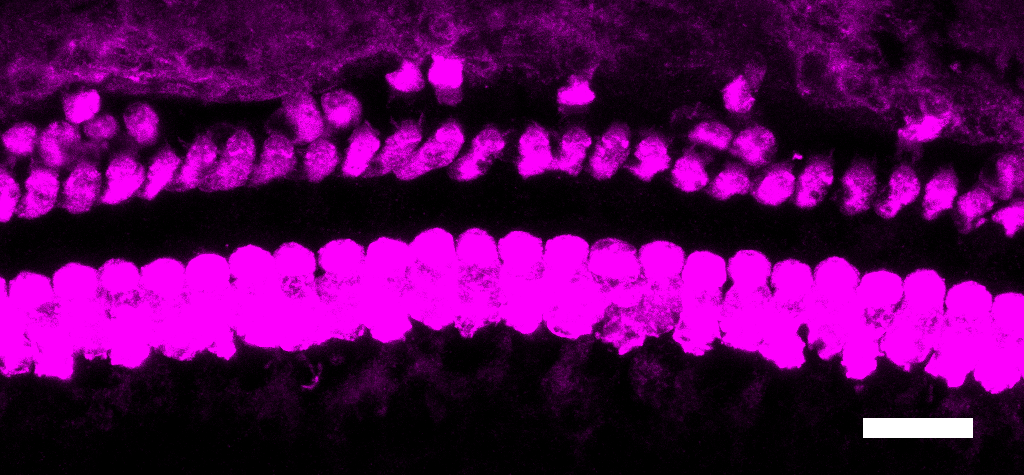

Supplement: Supplementary file 13 — Source data Fig. 5 [file 44321_2026_433_MOESM13_ESM.zip › Figure 5/5F/8w-high-apex-myo.tif]

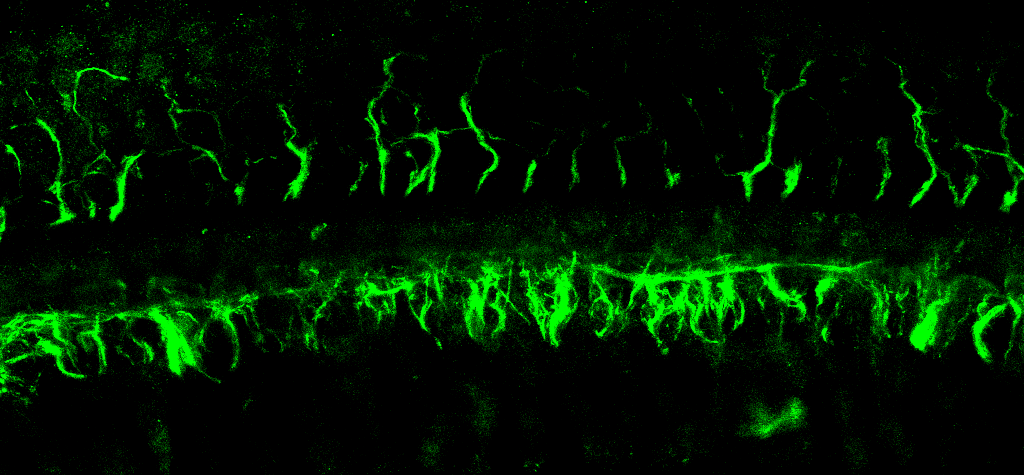

Supplement: Supplementary file 13 — Source data Fig. 5 [file 44321_2026_433_MOESM13_ESM.zip › Figure 5/5F/8w-high-base-NF200.tif]

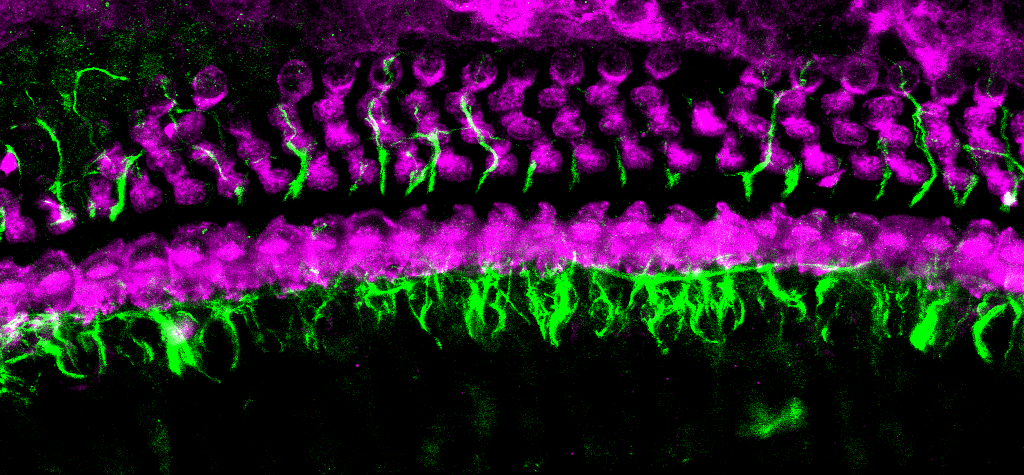

Supplement: Supplementary file 13 — Source data Fig. 5 [file 44321_2026_433_MOESM13_ESM.zip › Figure 5/5F/8w-high-base-merge.tif]

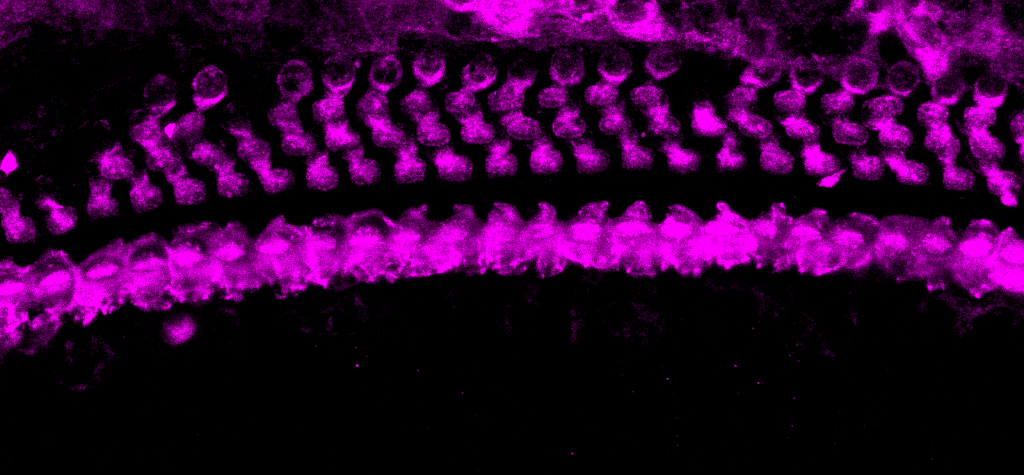

Supplement: Supplementary file 13 — Source data Fig. 5 [file 44321_2026_433_MOESM13_ESM.zip › Figure 5/5F/8w-high-base-myo.tif]

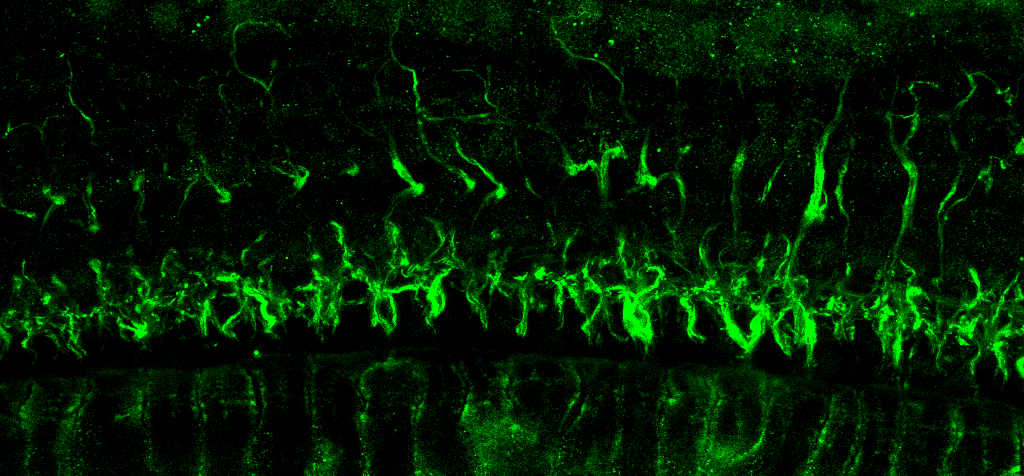

Supplement: Supplementary file 13 — Source data Fig. 5 [file 44321_2026_433_MOESM13_ESM.zip › Figure 5/5F/8w-high-mid-NF200.tif]

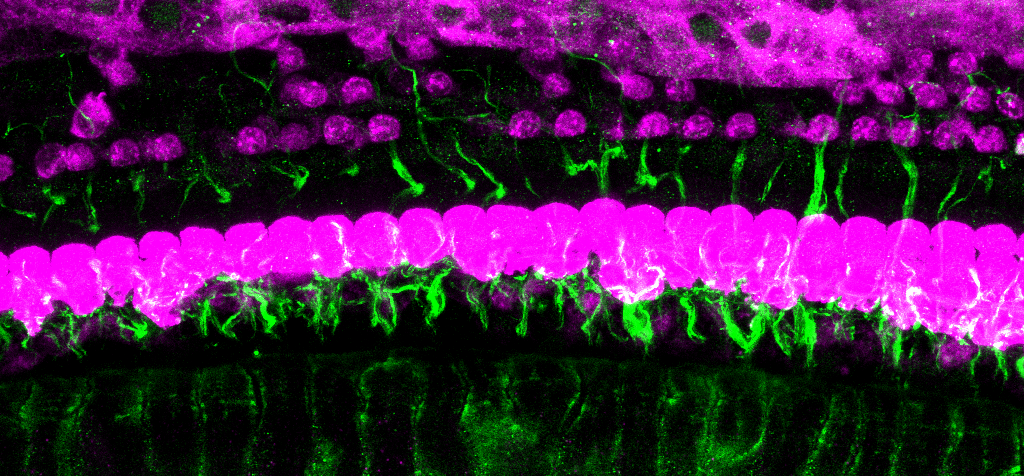

Supplement: Supplementary file 13 — Source data Fig. 5 [file 44321_2026_433_MOESM13_ESM.zip › Figure 5/5F/8w-high-mid-merge.tif]

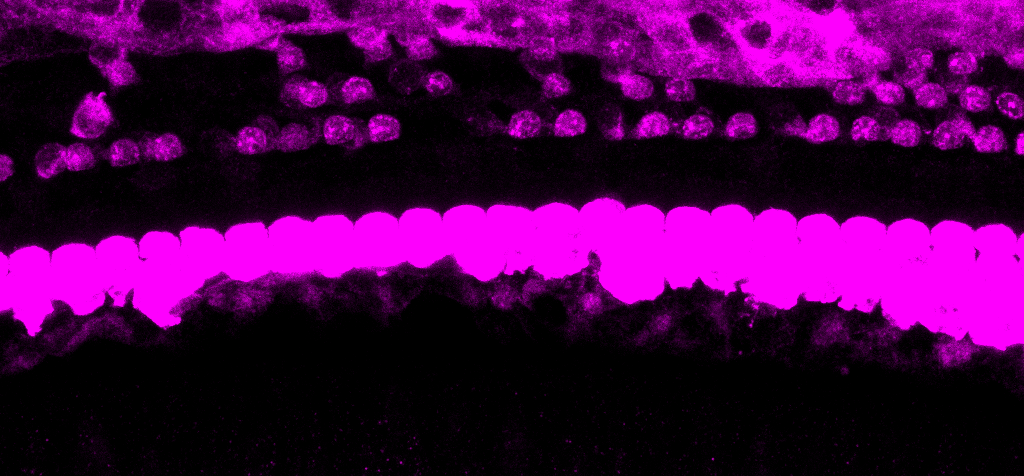

Supplement: Supplementary file 13 — Source data Fig. 5 [file 44321_2026_433_MOESM13_ESM.zip › Figure 5/5F/8w-high-mid-myo.tif]

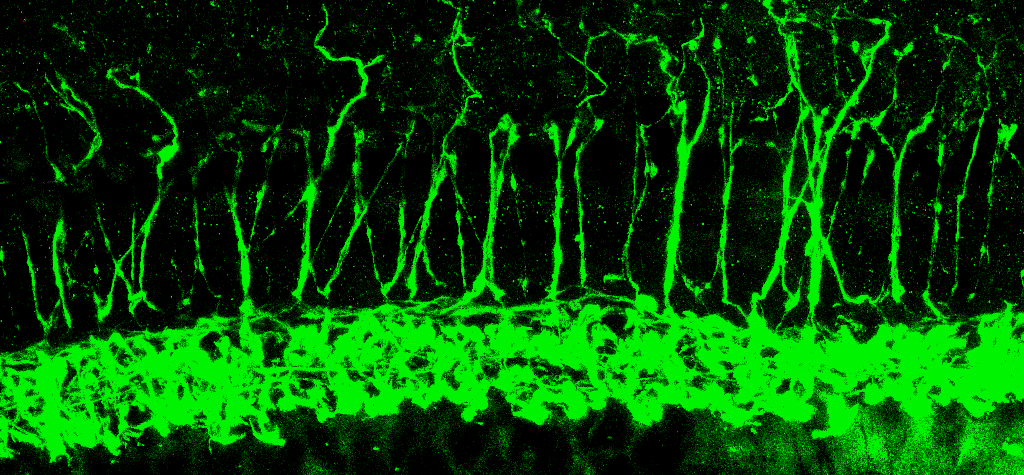

Supplement: Supplementary file 13 — Source data Fig. 5 [file 44321_2026_433_MOESM13_ESM.zip › Figure 5/5F/8w-low-apex-NF200.tif]

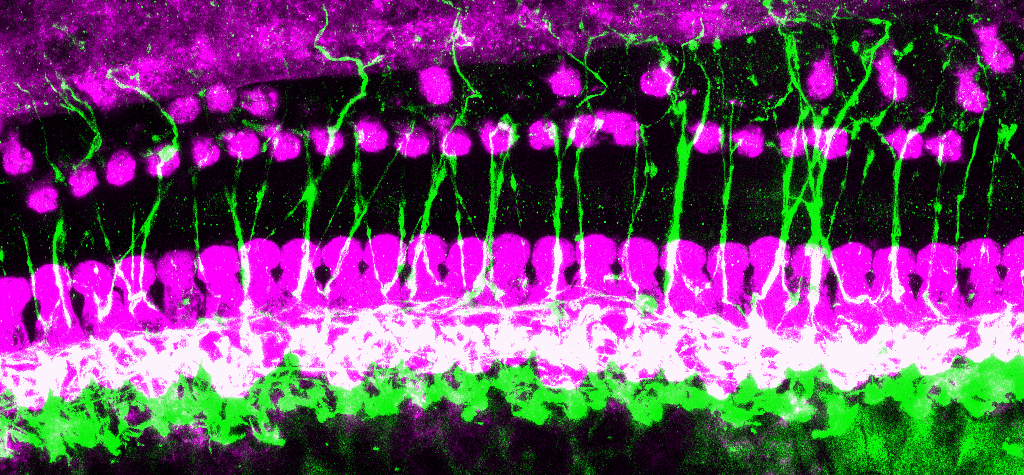

Supplement: Supplementary file 13 — Source data Fig. 5 [file 44321_2026_433_MOESM13_ESM.zip › Figure 5/5F/8w-low-apex-merge.tif]

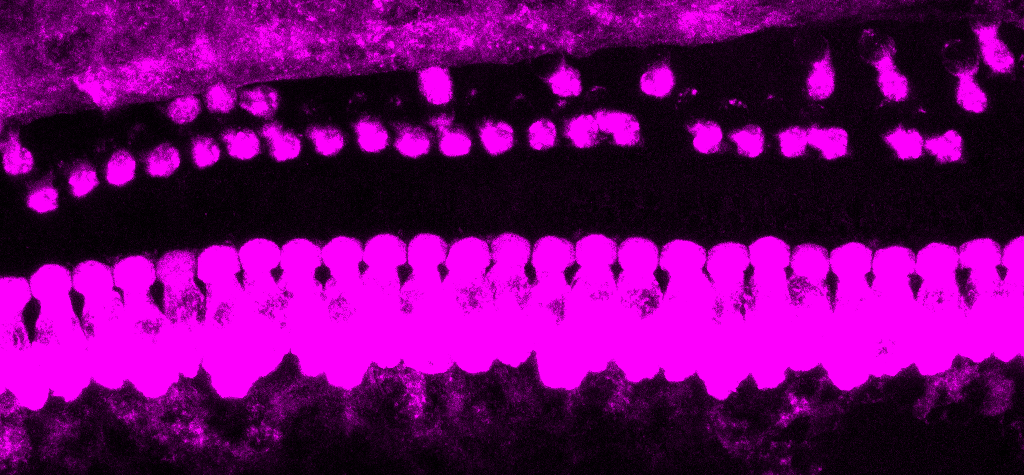

Supplement: Supplementary file 13 — Source data Fig. 5 [file 44321_2026_433_MOESM13_ESM.zip › Figure 5/5F/8w-low-apwx-myo.tif]

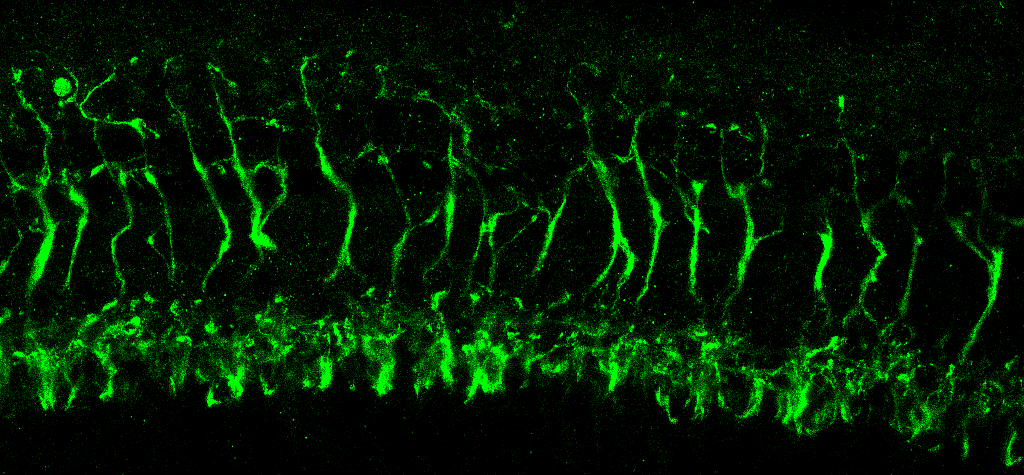

Supplement: Supplementary file 13 — Source data Fig. 5 [file 44321_2026_433_MOESM13_ESM.zip › Figure 5/5F/8w-low-base-NF200.tif]

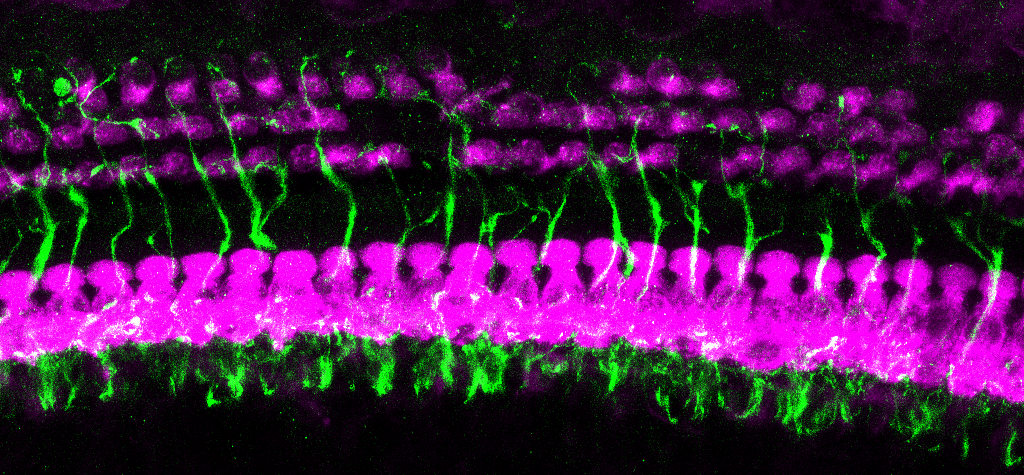

Supplement: Supplementary file 13 — Source data Fig. 5 [file 44321_2026_433_MOESM13_ESM.zip › Figure 5/5F/8w-low-base-merge.tif]

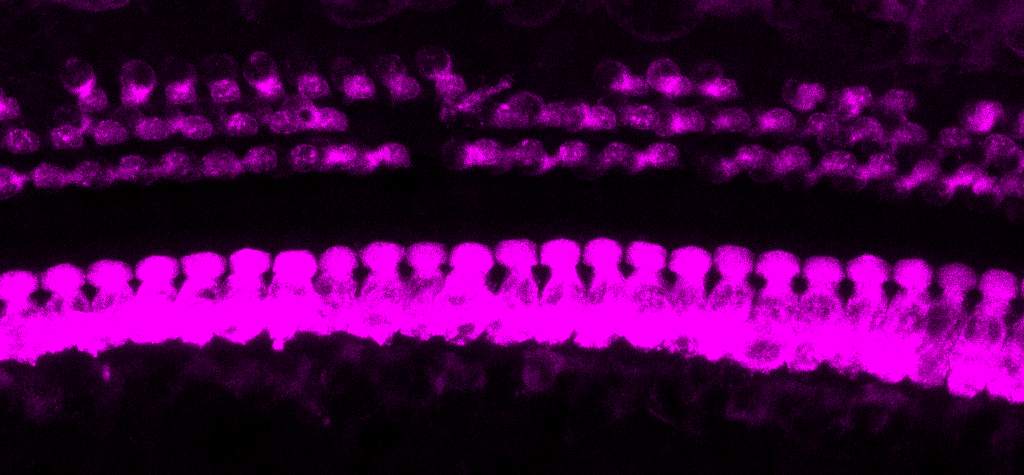

Supplement: Supplementary file 13 — Source data Fig. 5 [file 44321_2026_433_MOESM13_ESM.zip › Figure 5/5F/8w-low-base-myo.tif]

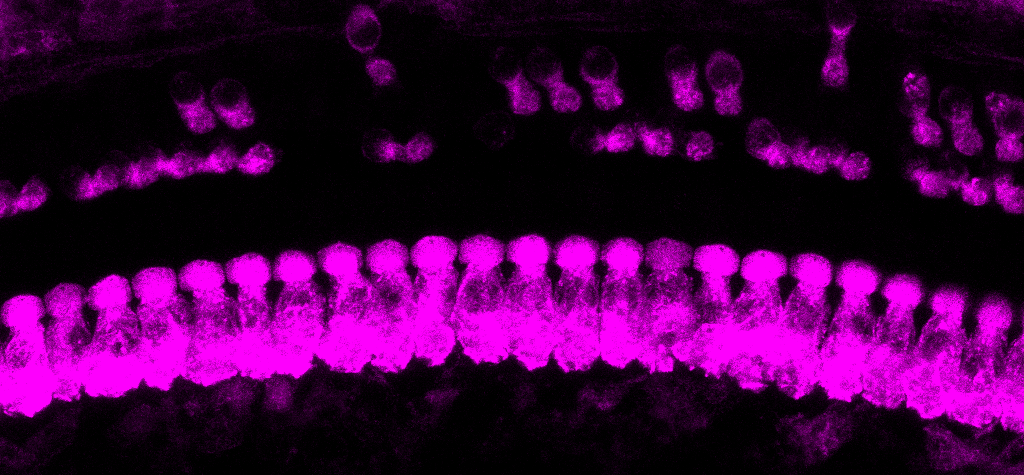

Supplement: Supplementary file 13 — Source data Fig. 5 [file 44321_2026_433_MOESM13_ESM.zip › Figure 5/5F/8w-low-mid-MYO.tif]

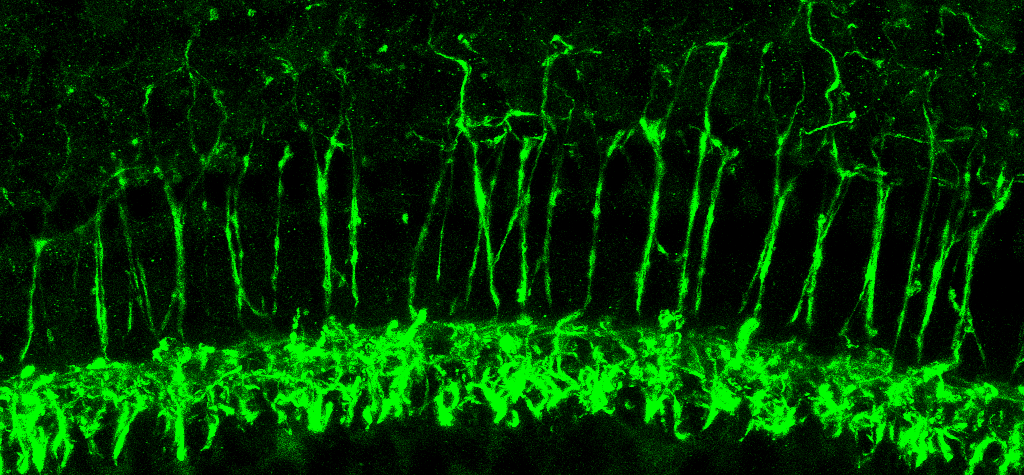

Supplement: Supplementary file 13 — Source data Fig. 5 [file 44321_2026_433_MOESM13_ESM.zip › Figure 5/5F/8w-low-mid-NF200.tif]

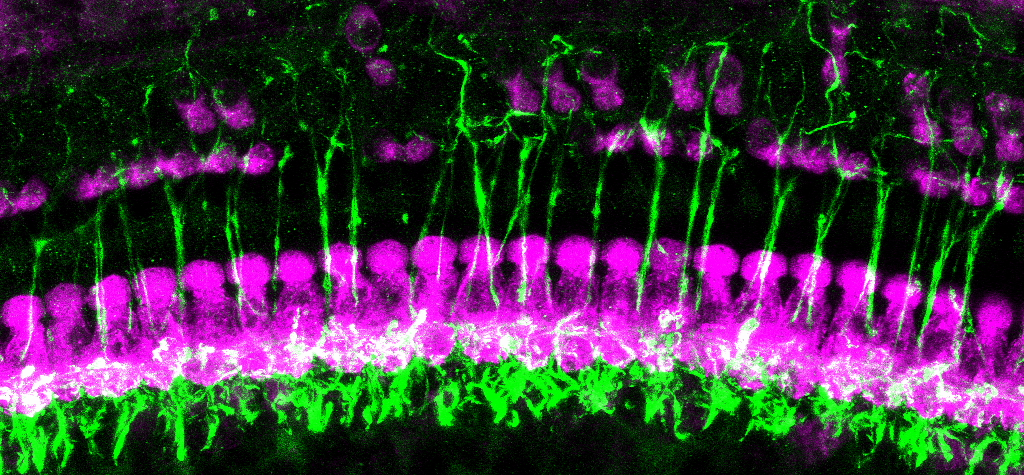

Supplement: Supplementary file 13 — Source data Fig. 5 [file 44321_2026_433_MOESM13_ESM.zip › Figure 5/5F/8w-low-mid-merge.tif]

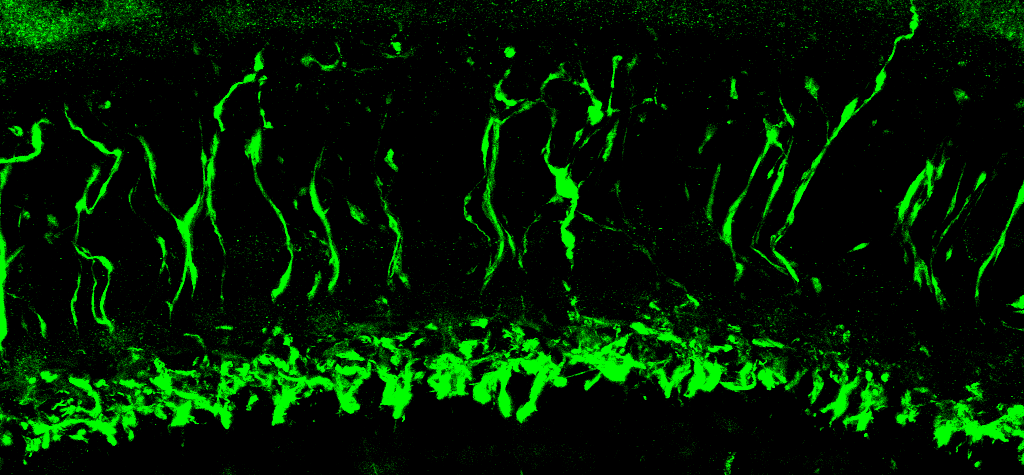

Supplement: Supplementary file 13 — Source data Fig. 5 [file 44321_2026_433_MOESM13_ESM.zip › Figure 5/5F/8w-wt-apex-NF200.tif]

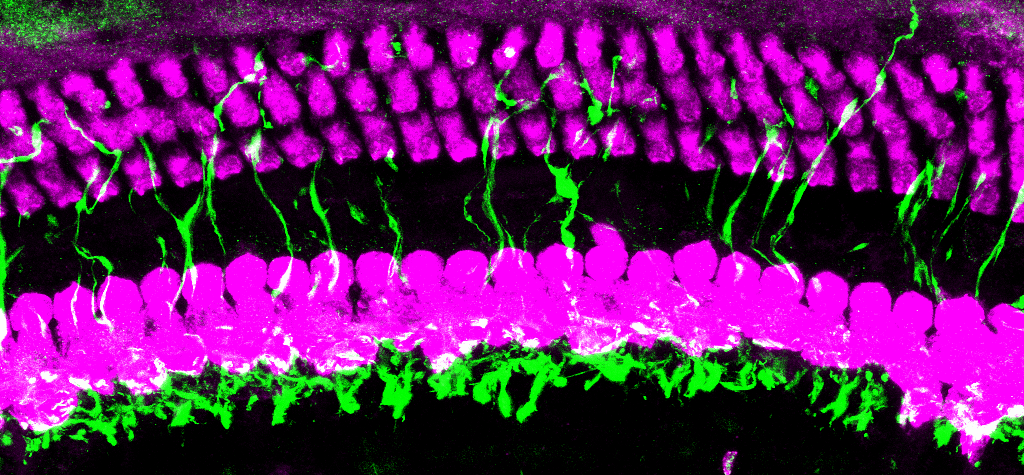

Supplement: Supplementary file 13 — Source data Fig. 5 [file 44321_2026_433_MOESM13_ESM.zip › Figure 5/5F/8w-wt-apex-merge.tif]

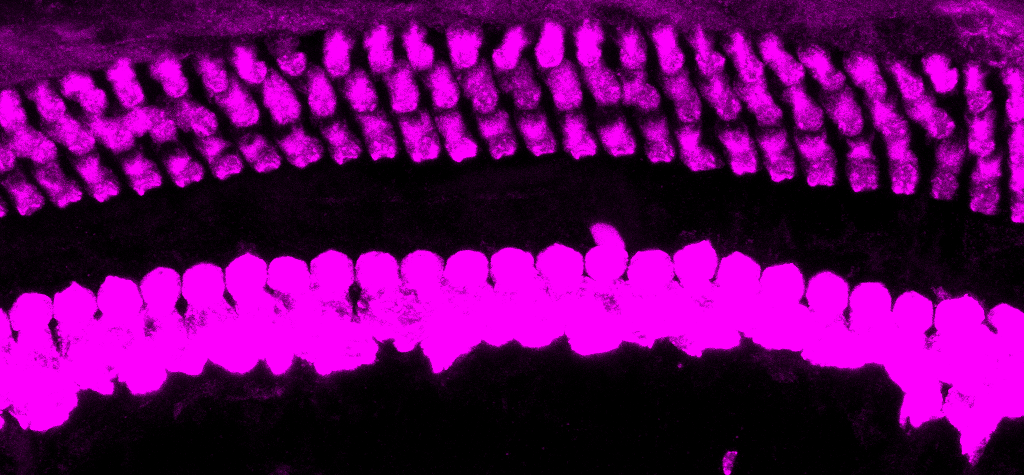

Supplement: Supplementary file 13 — Source data Fig. 5 [file 44321_2026_433_MOESM13_ESM.zip › Figure 5/5F/8w-wt-apex-myo.tif]

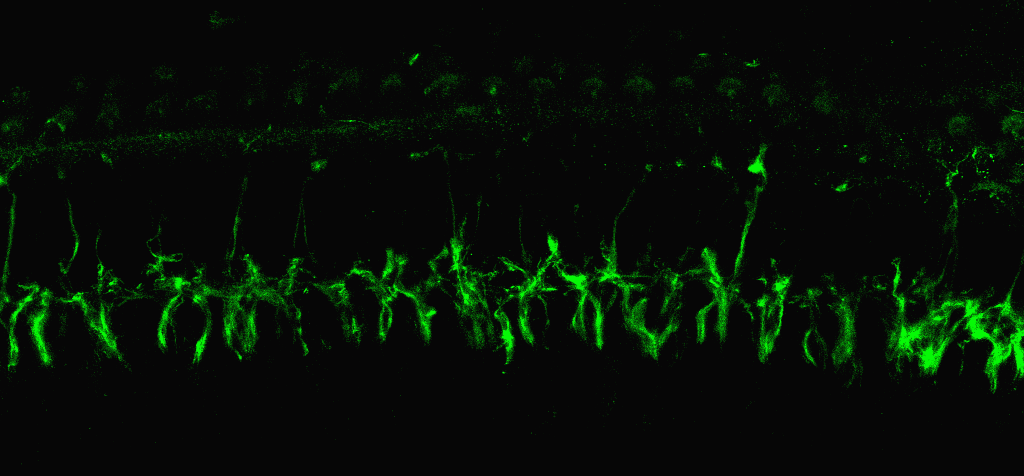

Supplement: Supplementary file 13 — Source data Fig. 5 [file 44321_2026_433_MOESM13_ESM.zip › Figure 5/5F/8w-wt-base-NF200.tif]

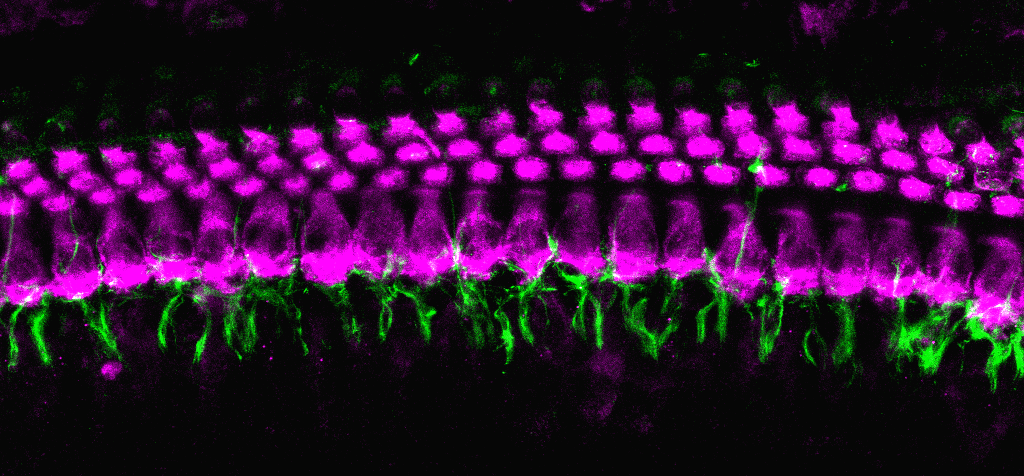

Supplement: Supplementary file 13 — Source data Fig. 5 [file 44321_2026_433_MOESM13_ESM.zip › Figure 5/5F/8w-wt-base-merge.tif]

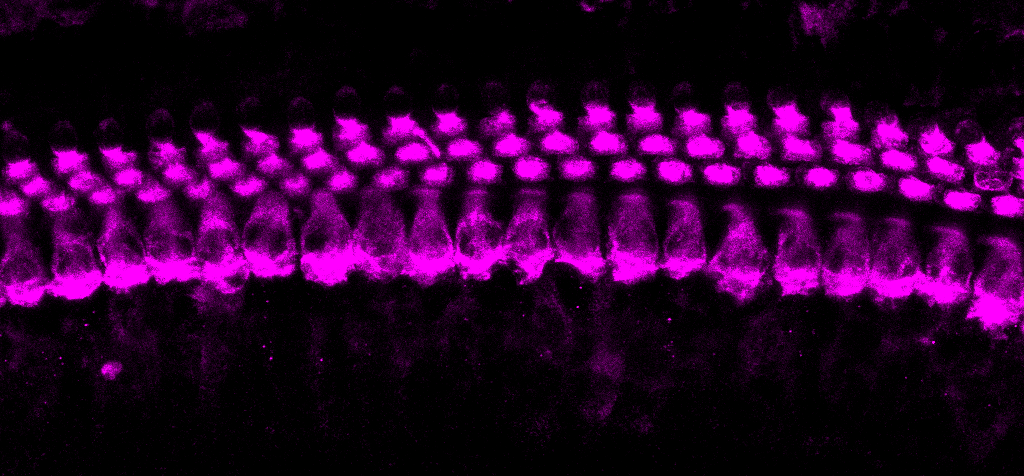

Supplement: Supplementary file 13 — Source data Fig. 5 [file 44321_2026_433_MOESM13_ESM.zip › Figure 5/5F/8w-wt-base-myo.tif]

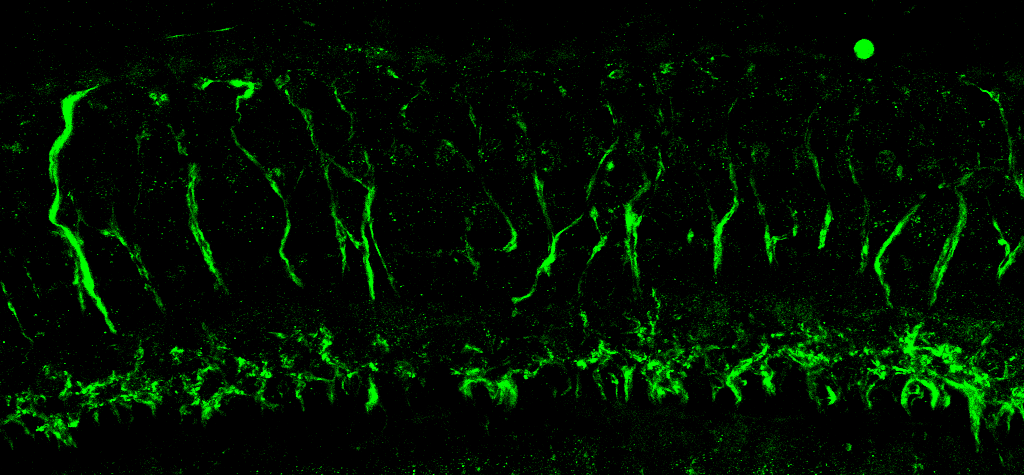

Supplement: Supplementary file 13 — Source data Fig. 5 [file 44321_2026_433_MOESM13_ESM.zip › Figure 5/5F/8w-wt-mid-NF200.tif]

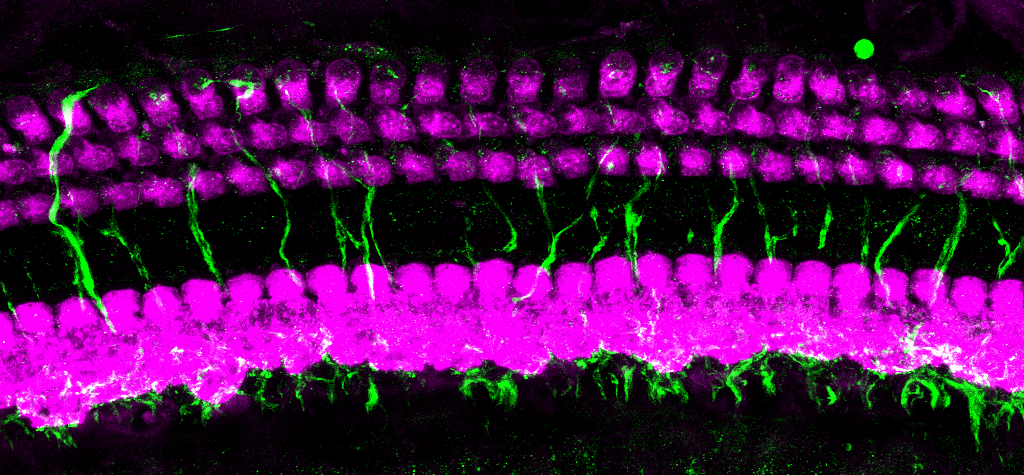

Supplement: Supplementary file 13 — Source data Fig. 5 [file 44321_2026_433_MOESM13_ESM.zip › Figure 5/5F/8w-wt-mid-merge.tif]

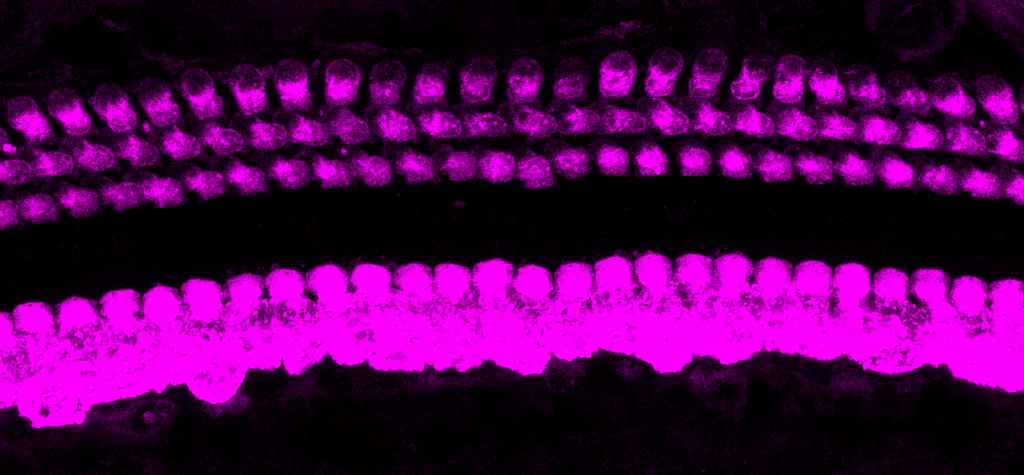

Supplement: Supplementary file 13 — Source data Fig. 5 [file 44321_2026_433_MOESM13_ESM.zip › Figure 5/5F/8w-wt-mid-myo.tif]

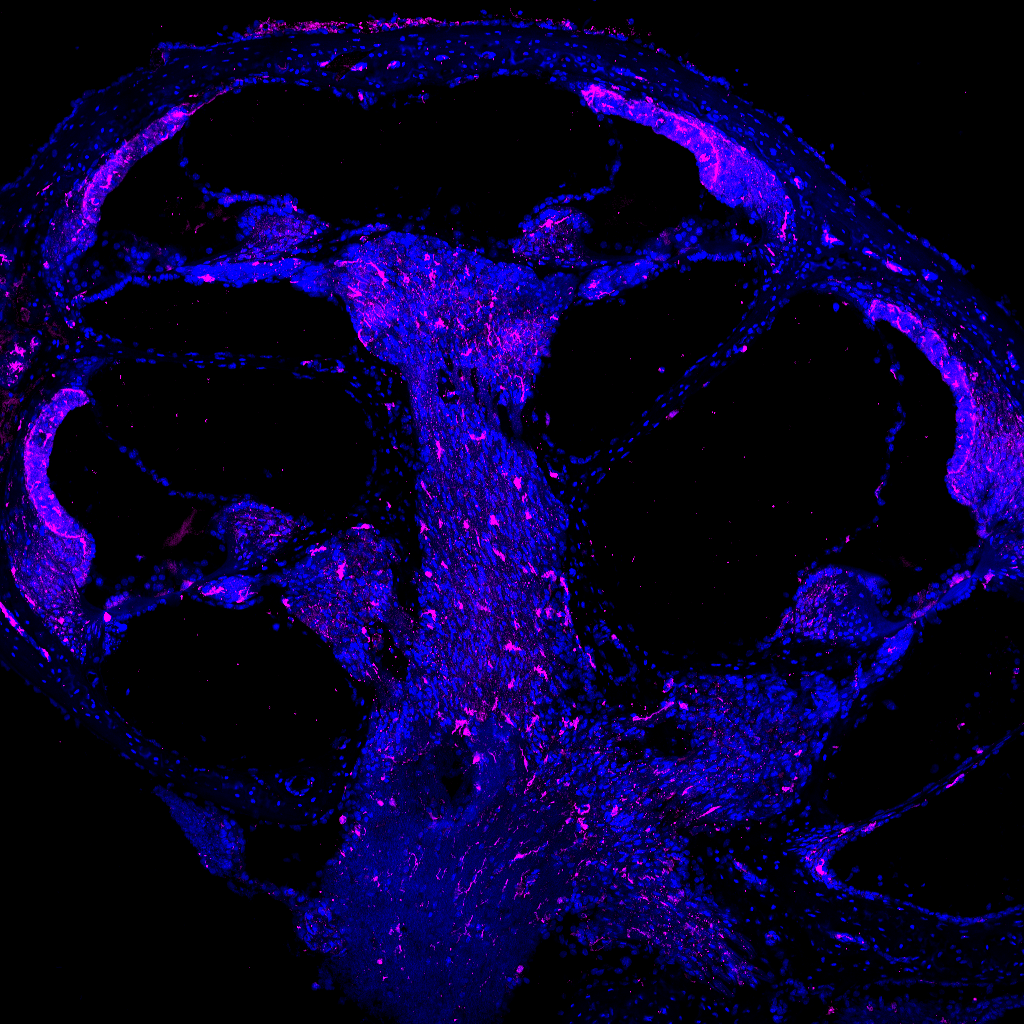

Supplement: Supplementary file 15 — Source data Fig. 7 [file 44321_2026_433_MOESM15_ESM.zip › Figure 7/7D/3w-ctrl.tif]

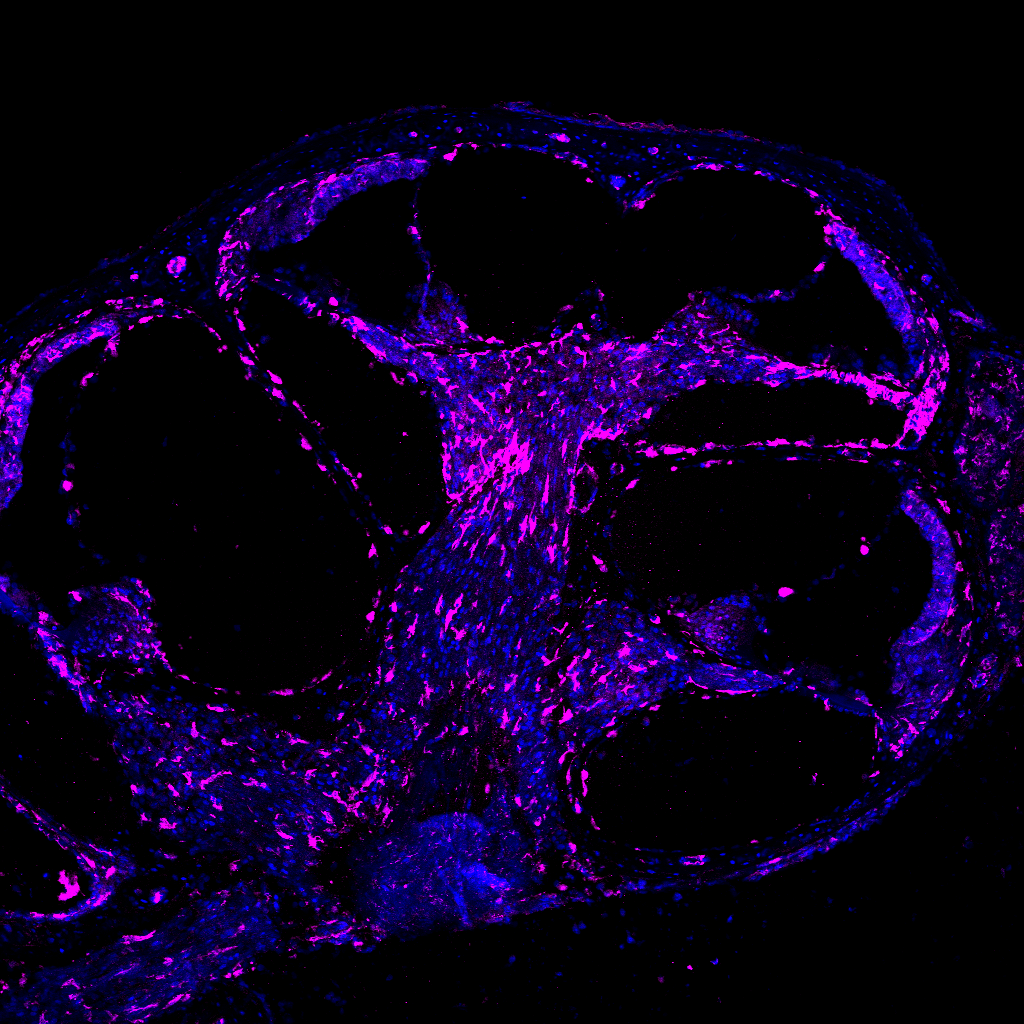

Supplement: Supplementary file 15 — Source data Fig. 7 [file 44321_2026_433_MOESM15_ESM.zip › Figure 7/7D/3w-high.tif]

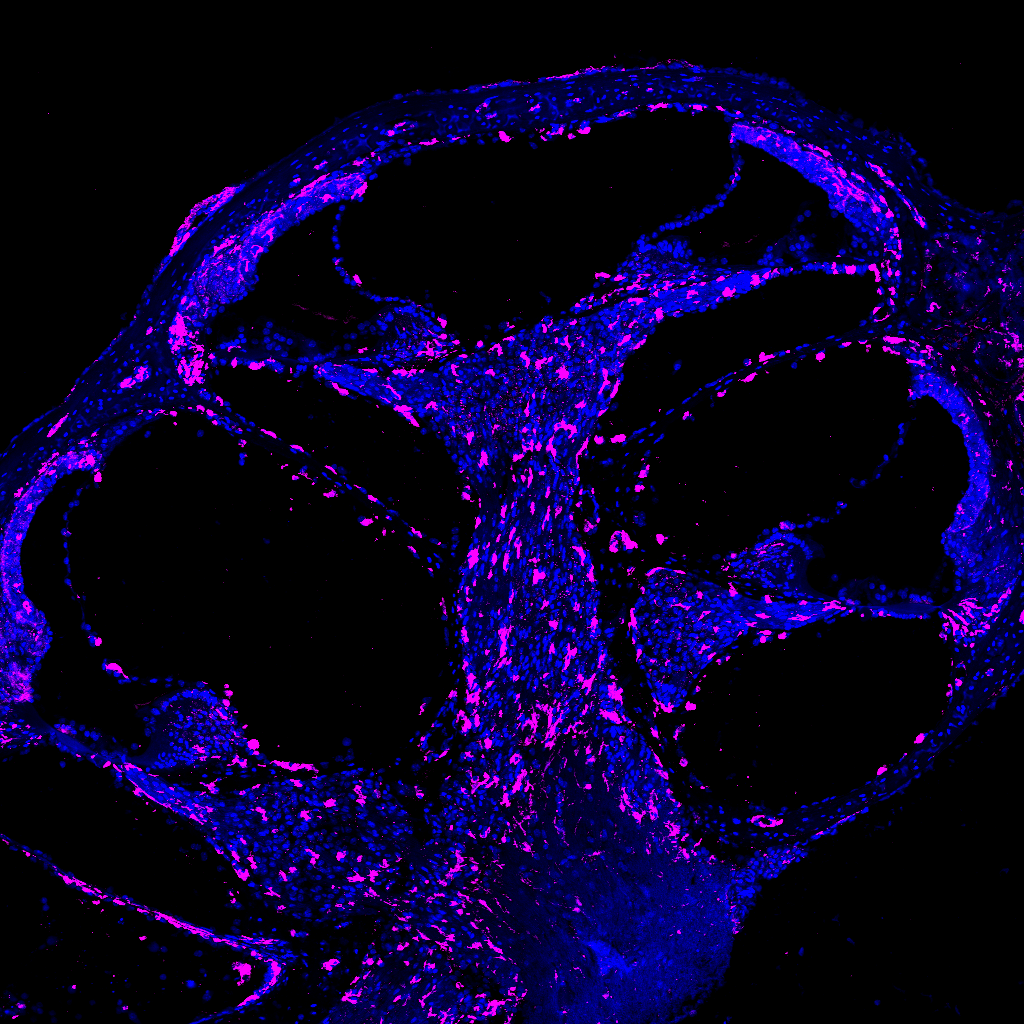

Supplement: Supplementary file 15 — Source data Fig. 7 [file 44321_2026_433_MOESM15_ESM.zip › Figure 7/7D/3w-low.tif]

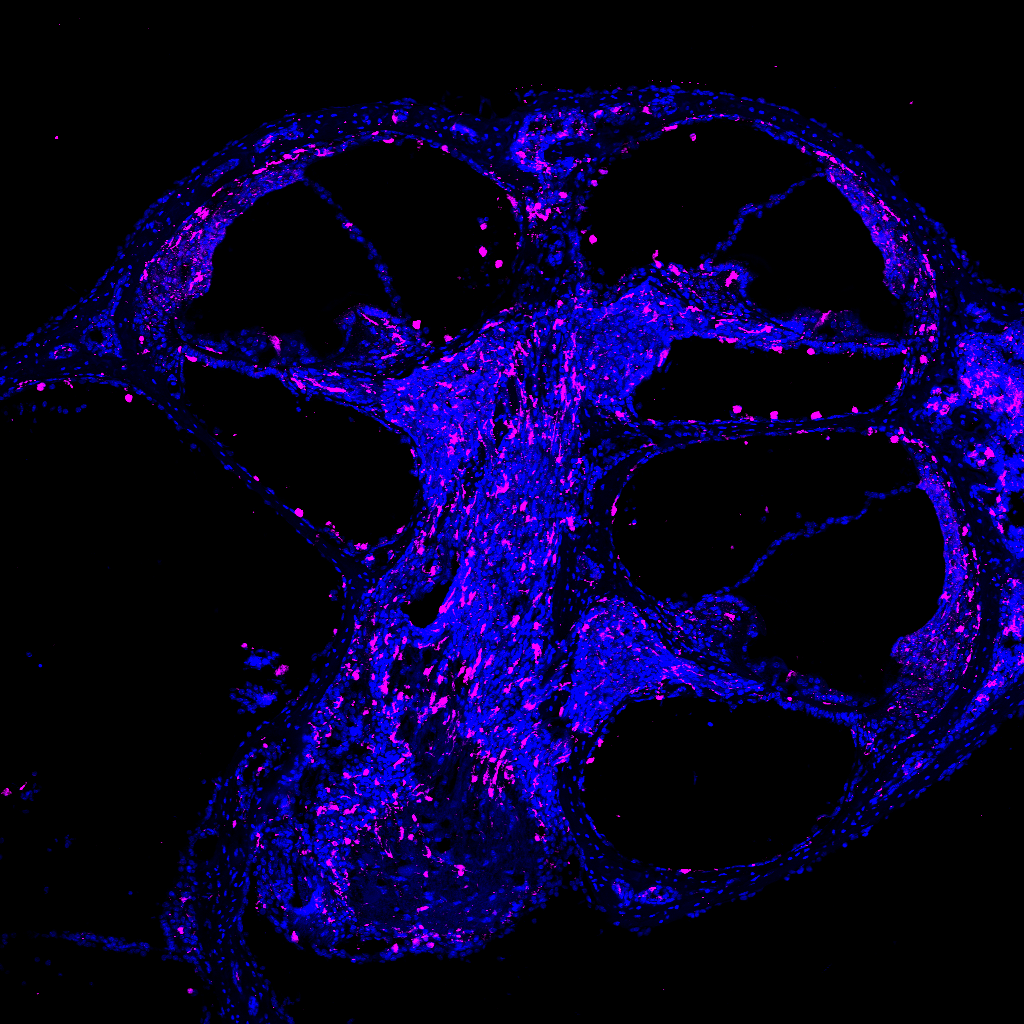

Supplement: Supplementary file 15 — Source data Fig. 7 [file 44321_2026_433_MOESM15_ESM.zip › Figure 7/7D/P10-ctrl.tif]

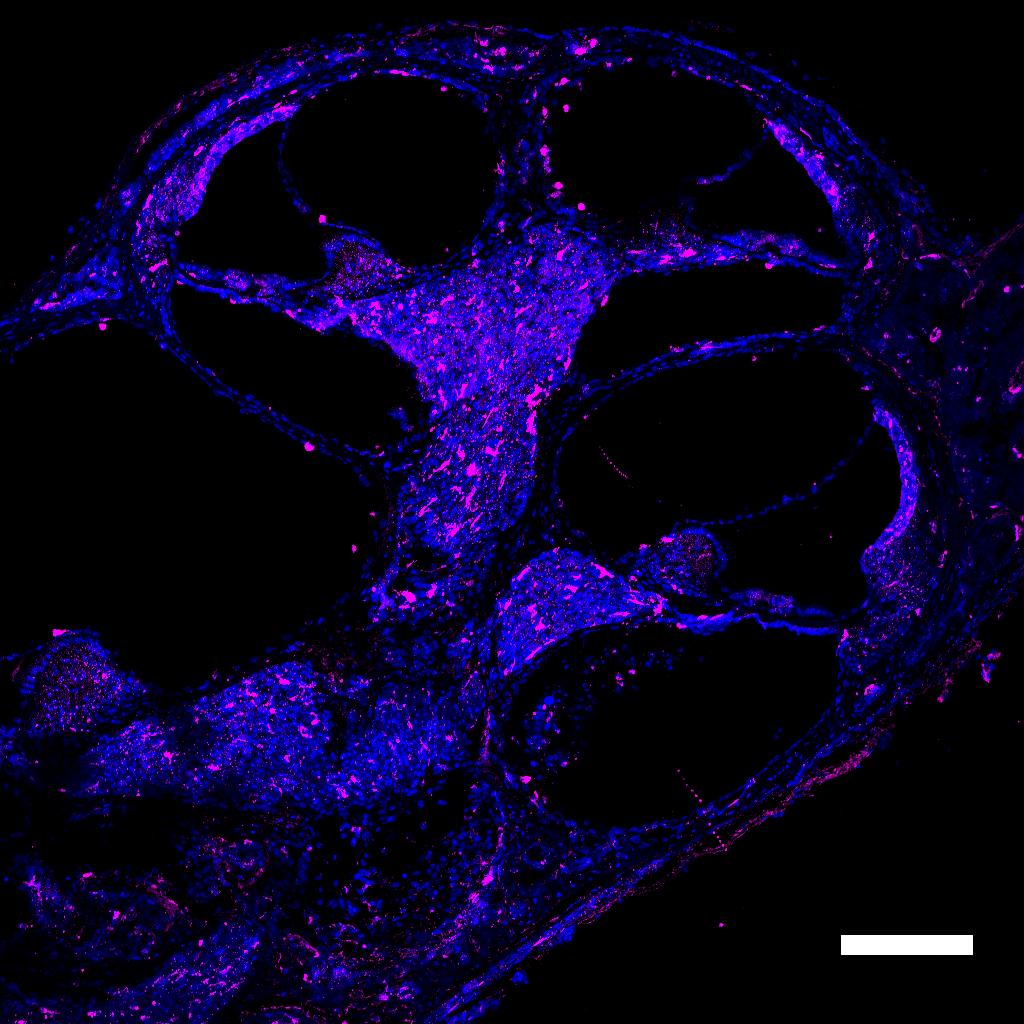

Supplement: Supplementary file 15 — Source data Fig. 7 [file 44321_2026_433_MOESM15_ESM.zip › Figure 7/7D/P10-high.tif]

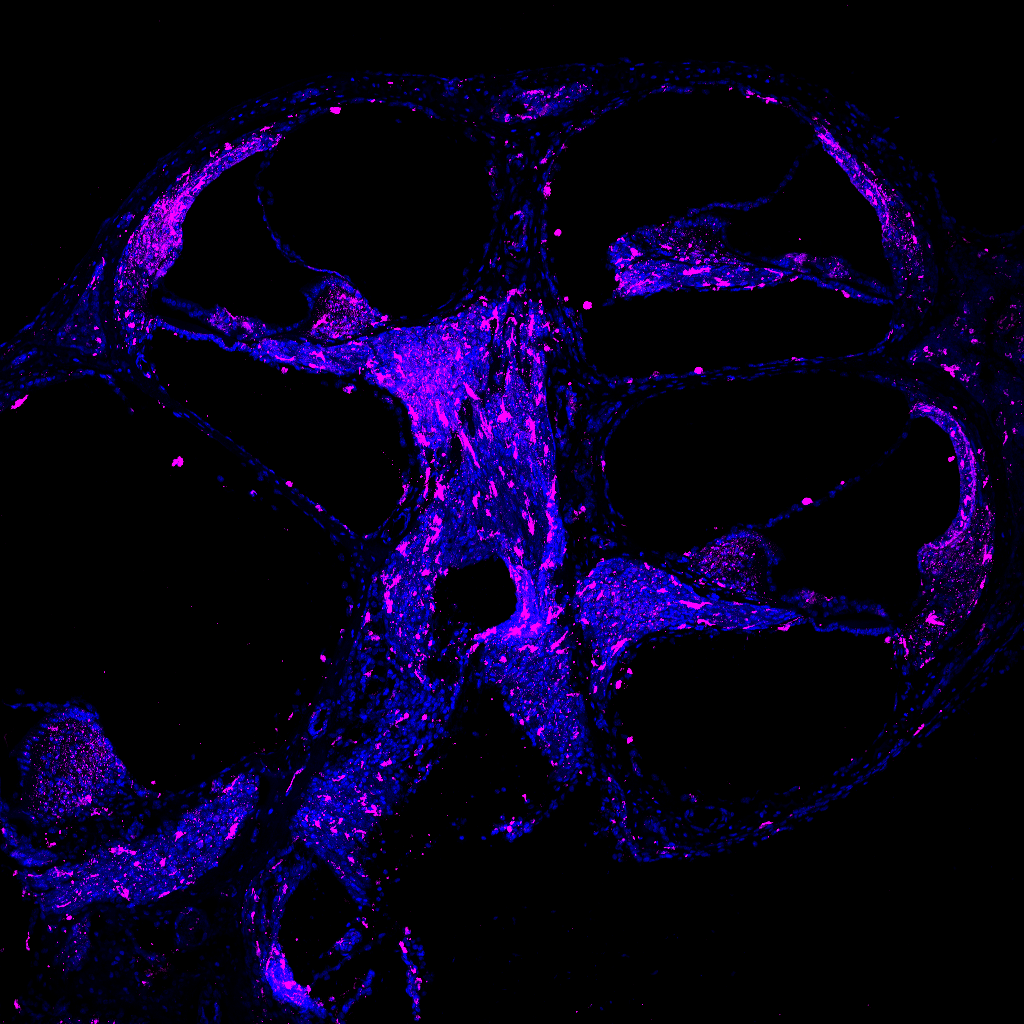

Supplement: Supplementary file 15 — Source data Fig. 7 [file 44321_2026_433_MOESM15_ESM.zip › Figure 7/7D/P10-low.tif]
