# Supplementary material for: Long-term restoration of auditory function in a DFNA2 mouse model by adenine base editing
Source: EMBO Mol Med. 2026 May 20;18(6):2293–321. doi: 10.1038/s44321-026-00433-5 (PMC13270111; doi:10.1038/s44321-026-00433-5)
Supplement: Supplementary file 16 — Expanded View Figures [file 44321_2026_433_MOESM16_ESM.pdf]

Expanded View Figures

Figure EV1. Generation of the *Kcnq4* c.964 G > A (p.G322S) knock-in mouse model and in vitro correction of the human *KCNQ4* c.961 G > A (p.G321S) variant.

(**A**) Schematic illustrating the *Kcnq4* c.964 G > A (p.G322S) substitution, recapitulating the pathogenic c.961 G > A (p.G321S) variant identified in DFNA2 patients. Red bases indicate disease-associated mutations; green bases represent synonymous substitutions; gray bases represent PAM sequence (NGG). The light red dashed box marks the optimal editing window of ABE8e. The sgRNA targeting the murine locus and the corresponding sgRNA for the human KCNQ4 allele are shown. (**B**) CRISPR/Cas9-mediated knock-in strategy for generating the *Kcnq4* c.964 G > A (p.G322S) model, incorporating a donor template with a synonymous p.R339= mutation to prevent Cas9 re-cutting. (**C**) Representative Sanger sequencing chromatograms confirming genotypes of $Kcnq4^{+/+}$, $Kcnq4^{+/G322S}$, and $Kcnq4^{G322S/G322S}$ mice. (**D**) HTS-based analysis of editing efficiency in a HEK293T cell line with stable integration of *KCNQ4* p.G321S following transfection with ABE8e and human-specific sgRNA ($n = 3$). Data were presented as mean ± SEM.

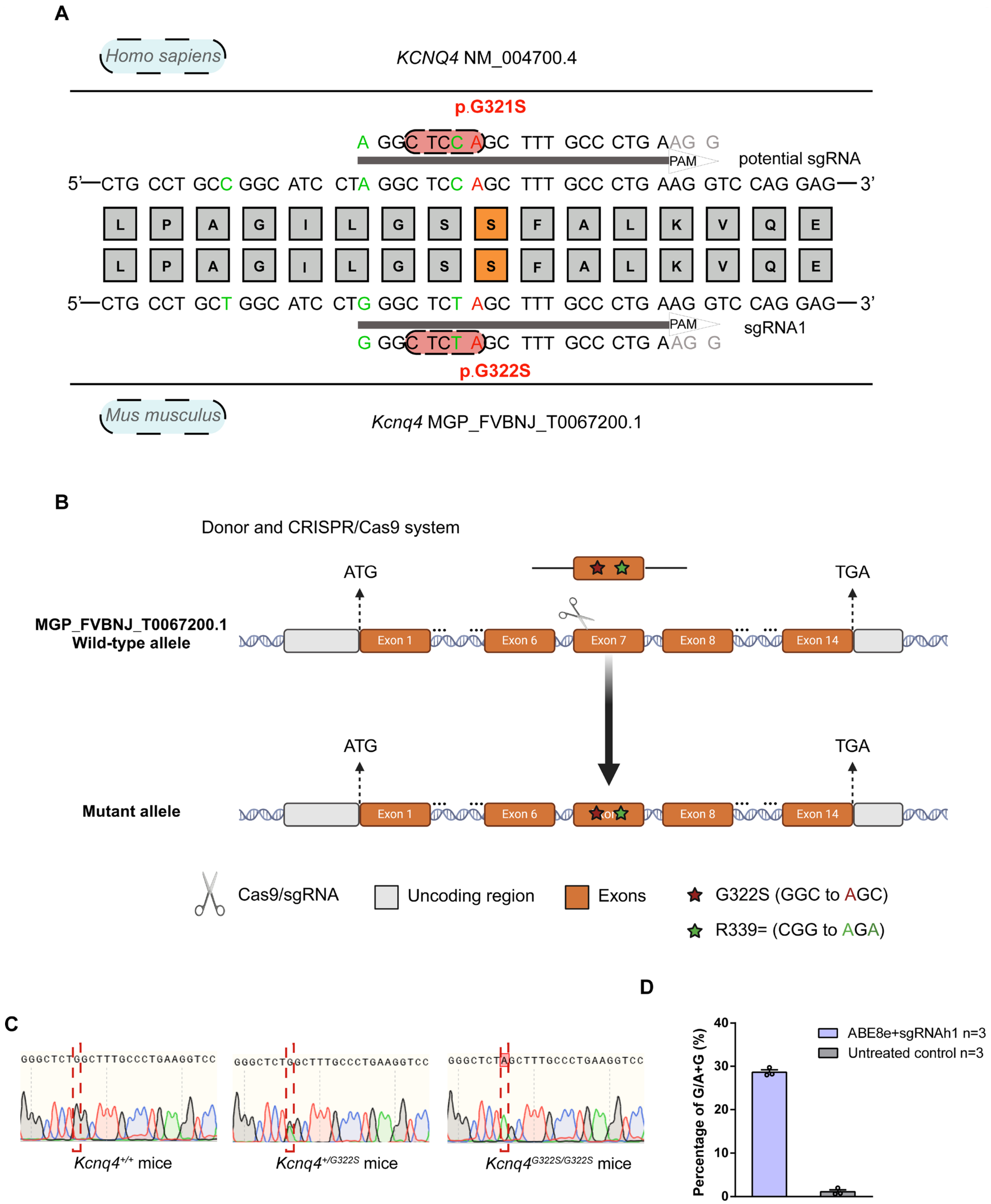
A
Homo sapiens
KCNQ4 NM_004700.4
p.G321S
A GGC TCC AGC TTT GCC CTG AAG G
PAM
potential sgRNA
5'—CTG CCT GCC GGC ATC CTA GGC TCC AGC TTT GCC CTG AAG GTC CAG GAG—3'
L P A G I L G S S F A L K V Q E
L P A G I L G S S F A L K V Q E
5'—CTG CCT GCT GGC ATC CTG GGC TCT AGC TTT GCC CTG AAG GTC CAG GAG—3'
PAM
sgRNA1
G GGC TCT AGC TTT GCC CTG AAG G
p.G322S
Mus musculus
Kcnq4 MGP_FVBNJ_T0067200.1
B
Donor and CRISPR/Cas9 system
ATG
TGA
MGP_FVBNJ_T0067200.1
Wild-type allele
Exon 1
Exon 6
Exon 7
Exon 8
Exon 14
Mutant allele
Cas9/sgRNA
Uncoding region
Exons
G322S (GGC to AGC)
R339= (CGG to AGA)
C
GGGCTCTGGCTTTGCCCTGAAGGTCC
GGGCTCTGGCTTTGCCCTGAAGGTCC
GGGCTCTAGCTTTGCCCTGAAGGTCC
Kcnq4+/+ mice
Kcnq4+/G322S mice
Kcnq4G322S/G322S mice
D
Percentage of G/A+G (%)
40
30
20
10
0
ABE8e+sgRNAh1 n=3
Untreated control n=3

 © The Author(s)

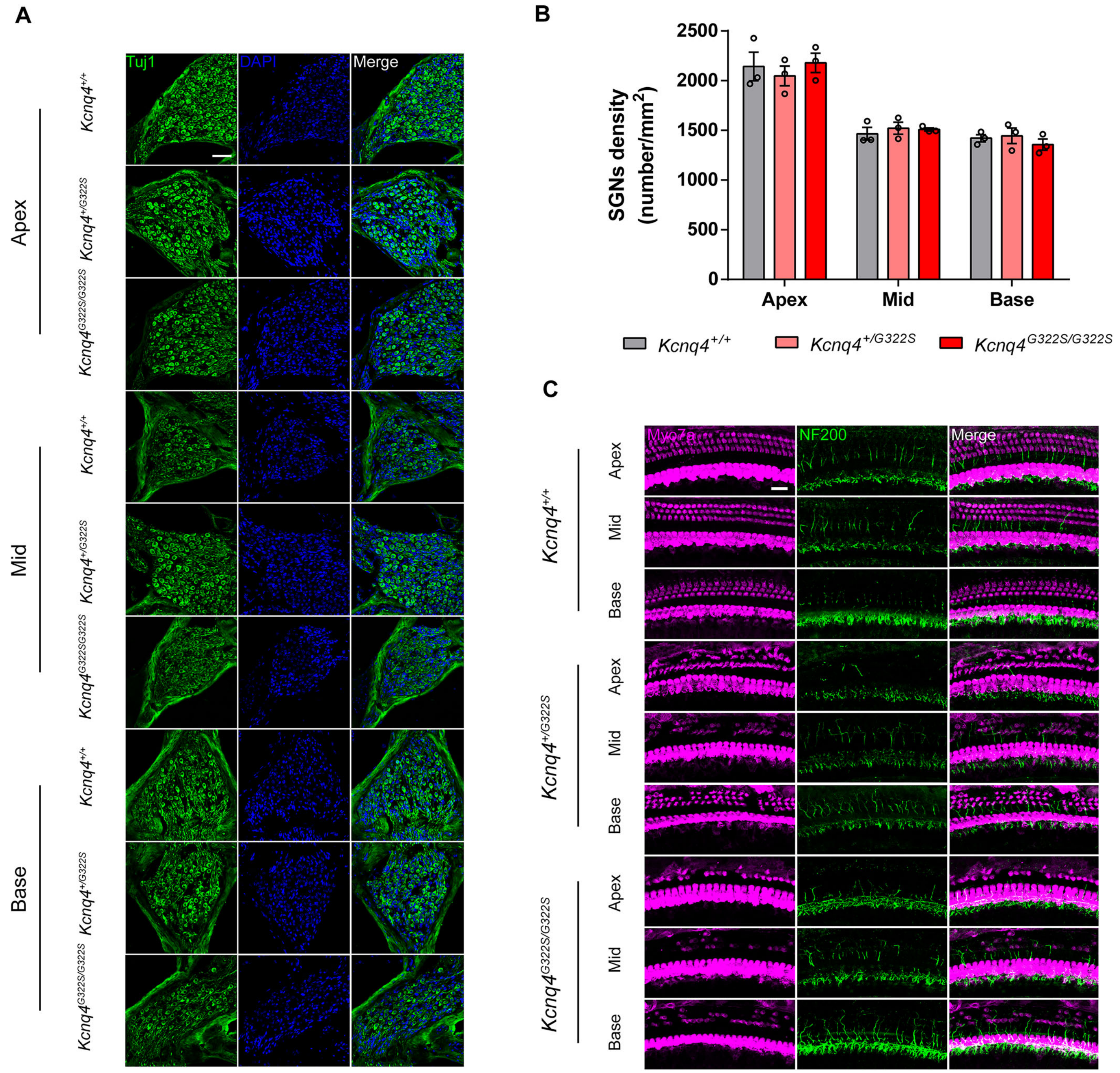

Figure EV2. Morphological analysis of SGNs and afferent/efferent nerve fibers by immunofluorescence staining in the *Kcnq4* p.G322S mouse model at 4 weeks of age.

(**A**) Representative immunofluorescence images of SGNs in the apical, middle, and basal cochlear turns from *Kcnq4*$^{+/+}$, *Kcnq4*$^{+/G322S}$, and *Kcnq4*$^{G322S/G322S}$ mice. Scale bar, 50 μm. (**B**) Quantification of SGN density in (**A**) from each genotype ($n = 3$). (**C**) Representative immunofluorescence images of afferent/efferent nerve fibers in the apical, middle, and basal cochlear turns from *Kcnq4*$^{+/+}$, *Kcnq4*$^{+/G322S}$, and *Kcnq4*$^{G322S/G322S}$ mice ($n = 3$). Scale bar, 20 μm. Data were presented as mean ± SEM.

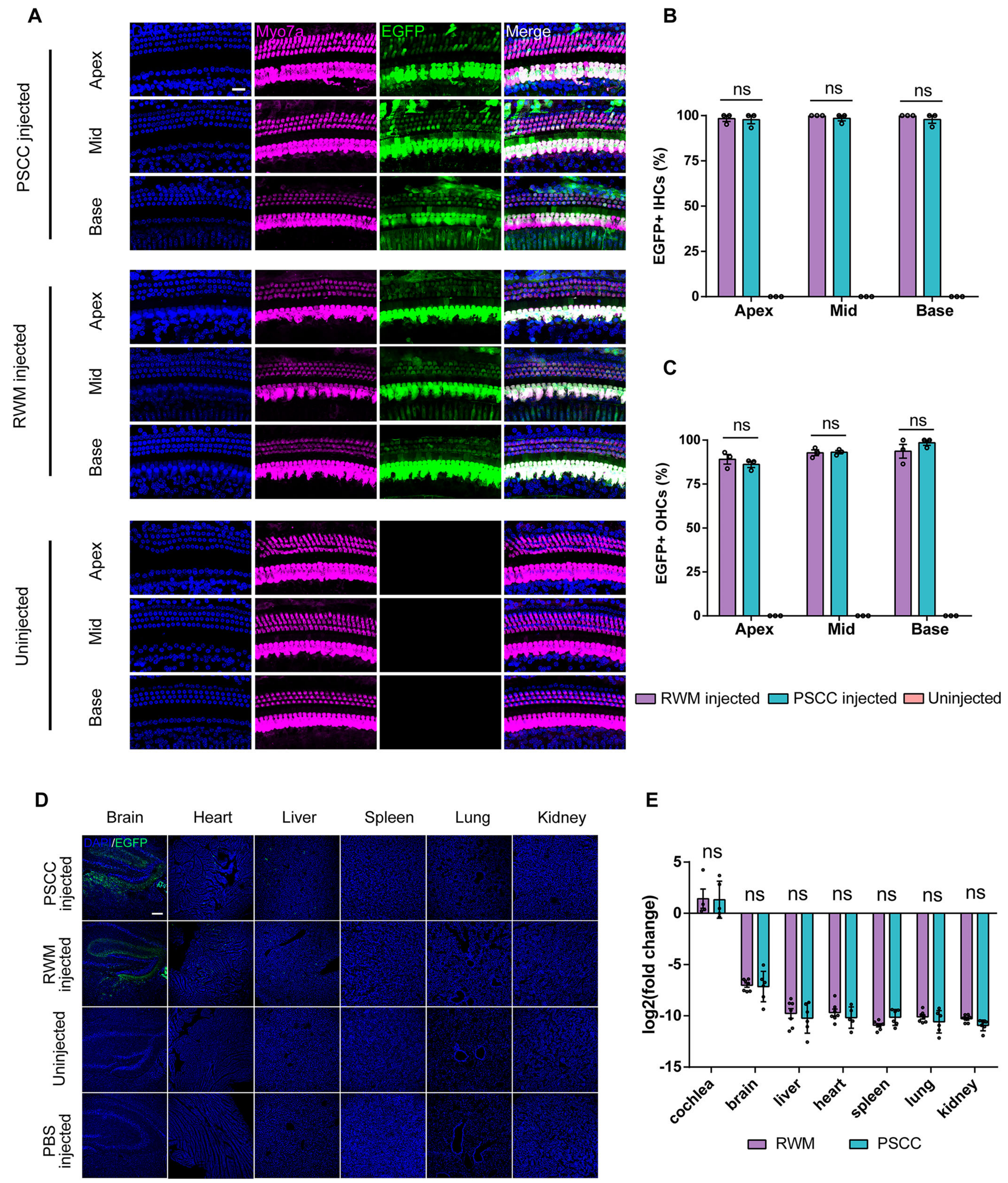
A
DAPI
Myo7a
EGFP
Merge
PSCC injected
RWM injected
Uninjected
Apex
Mid
Base
B
EGFP+ IHCs (%)
ns
C
EGFP+ OHCs (%)
RWM injected
PSCC injected
Uninjected
D
Brain
Heart
Liver
Spleen
Lung
Kidney
DAPI/EGFP
PSCC injected
RWM injected
Uninjected
PBS injected
E
log2(fold change)
cochlea
brain
liver
heart
spleen
lung
kidney
RWM
PSCC

 © The Author(s)

Figure EV3. Assessment of cochlear and major organ transduction following AAV-ie delivery via PSCC or RWM injection.

(**A**) Representative immunofluorescence images of the whole-mount from *Kcnq4*$^{+/+}$ mice three weeks after AAV-ie-CMV-EGFP administration via PSCC or RWM (1×10^{10} gc/mouse). (**B**, **C**) Transduction efficiency was quantified as the percentage of EGFP^{+} IHCs (**B**) and OHCs (**C**) across the apical, middle, and basal cochlear turns ($n = 3$). (**D**) Representative immunofluorescence images of major organs from *Kcnq4*$^{+/+}$ mice three weeks after AAV-ie-CMV-EGFP administration via PSCC or RWM injection (1×10^{10} gc/mouse), along with PBS-injected and uninjected controls. (**E**) RT-qPCR quantification of EGFP expression in major organs (**D**), normalized to cochlear levels (PSCC, cochlea, heart $n = 5$, others $n = 6$; RWM, cochlea $n = 4$, others $n = 7$). Data were presented as mean ± SEM. Statistical analysis was performed using two-way ANOVA with Bonferroni's post hoc test. ns not significant.

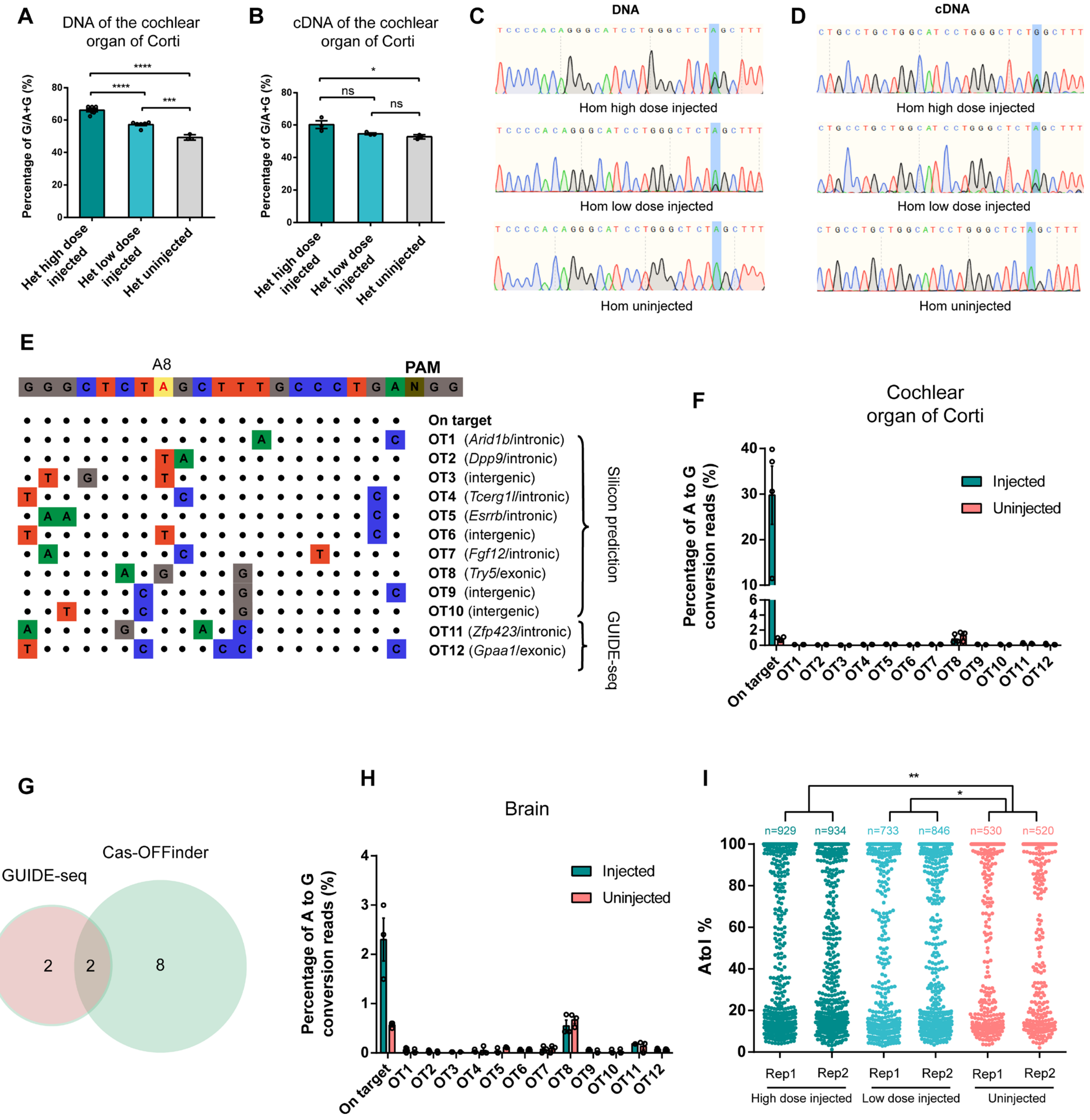

Figure EV4. Quantitative assessment of on-target editing efficiency and off-target effects.

(**A**) Post-editing G/(A + G) ratio in genomic DNA from the cochlear organ of Corti of *Kcnq4*$^{+/G322S}$ mice, assessed by HTS three weeks post-AAV_ABE8e_N + C_322 administration (high dose: 3.64 × 10^{10} gc/mouse, *n* = 6; low dose: 1.3 × 10^{10} gc/mouse, *n* = 6; uninjected *n* = 3). (**B**) Post-editing G/(A + G) ratio in cDNA from the cochlear organ of Corti of *Kcnq4*$^{+/G322S}$ mice, assessed by HTS three weeks post-AAV_ABE8e_N + C_322 administration (*n* = 3). (**C, D**) Representative Sanger sequencing chromatograms showing A-to-G conversion at the target site in genomic DNA (**C**) and cDNA (**D**) from the cochlear organ of Corti of *Kcnq4*$^{G322S/G322S}$ mice three weeks after AAV_ABE8e_N + C_322 treatment. (**E**) Predicted top ten off-target sites (≤3 mismatches, NGG PAM) identified by Cas-OFFinder and two sites detected by GUIDE-seq. (**F**) Off-target A-to-G editing efficiencies assessed by HTS in the cochlear organ of Corti of AAV_ABE8e_N + C_322 treated (3.64 × 10^{10} gc/mouse) *Kcnq4*$^{G322S/G322S}$ mice, 3 weeks post-injection (*n* = 3). (**G**) Venn diagram of the top ten Cas-OFFinder–predicted off-target sites and four GUIDE-seq–detected sites. (**H**) Off-target A-to-G editing efficiencies assessed by HTS in the brain of AAV_ABE8e_N + C_322-treated (3.64 × 10^{10} gc/mouse) *Kcnq4*$^{G322S/G322S}$ mice, 3 weeks post-injection (*n* = 3). (**I**) Transcriptome-wide A-to-I RNA editing in the cochlea evaluated by RNA-seq in *Kcnq4*$^{G322S/G322S}$ mice treated with AAV_ABE8e_N + C_322 (high-dose: 3.64 × 10^{10} gc/mouse; low-dose: 1.3 × 10^{10} gc/mouse; *n* = 2). Data were presented as mean ± SEM. Statistical analysis was performed using one-way or two-way ANOVA with Bonferroni's post hoc test. *$p < 0.05$; **$p < 0.01$; ***$p < 0.001$; ****$p < 0.0001$; ns not significant (Exact *P* values are provided in Appendix Table S1).

 © The Author(s)

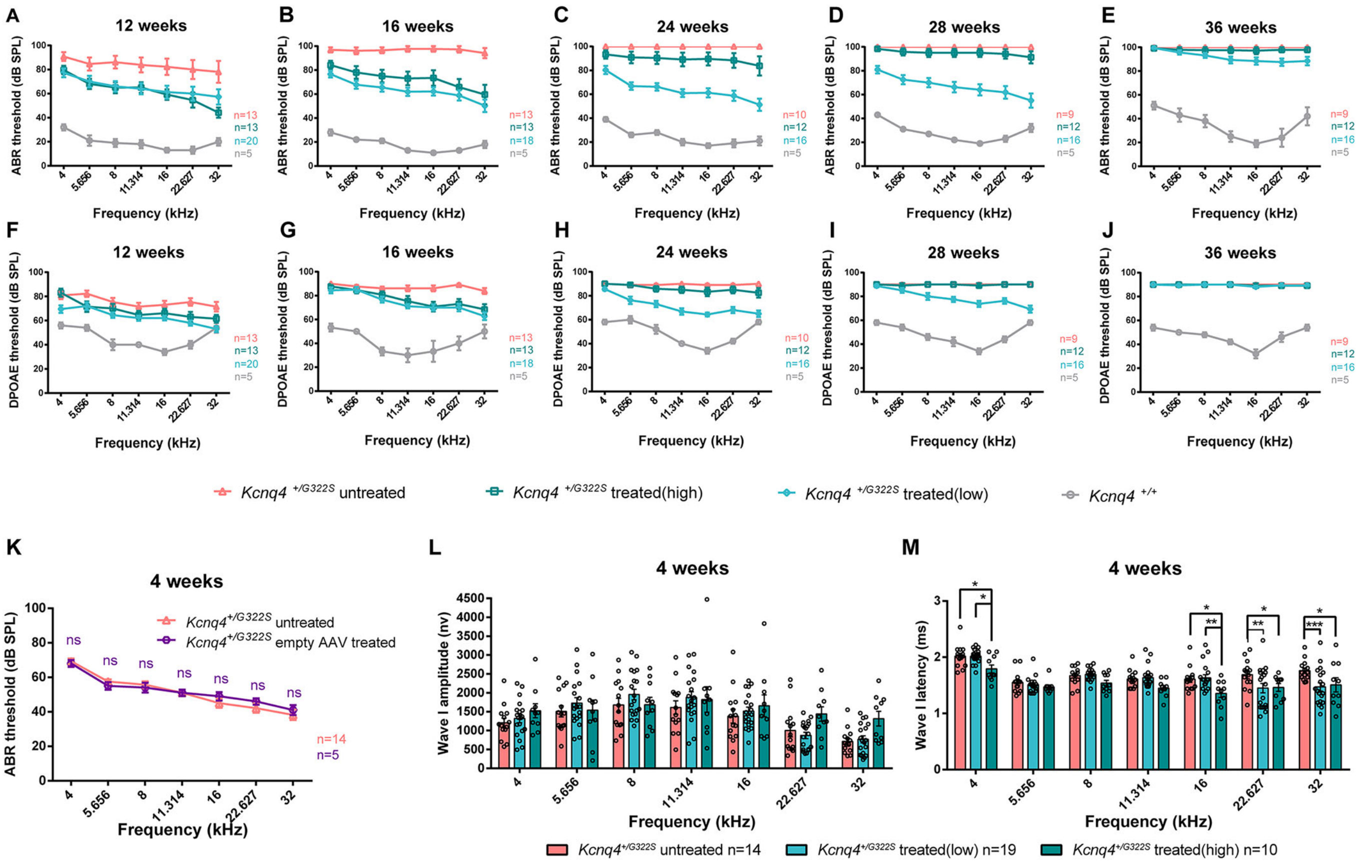

Figure EV5. Extended auditory evaluations following AAV_ABE8e_N + C_322 treatment.

(**A–J**) ABR and DPOAE measurements were performed at 12, 16, 24, 28, and 36 weeks post-treatment of AAV_ABE8e_N + C_322 in high-dose (3.64×10^{10} gc/mouse), low-dose (1.3×10^{10} gc/mouse), or in untreated *Kcnq4*$^{+/G322S}$ mice, as well as *Kcnq4*$^{+/+}$ controls. ABR thresholds are shown in (**A–E**); DPOAE thresholds in (**F–J**). Sample sizes (*n*) are indicated alongside each graph. (**K**) ABR thresholds in *Kcnq4*$^{+/G322S}$ mice treated with AAV_ABE8e_N + AAV_ABE8e_C_empty (3.64×10^{10} gc/mouse, $n = 5$) compared to untreated *Kcnq4*$^{+/G322S}$ controls ($n = 14$) at 4 weeks post-treatment. (**L**, **M**) Quantification of ABR wave I amplitude (L) and latency (**M**) at 90 dB SPL in high-dose ($n = 10$), low-dose ($n = 19$), and untreated ($n = 14$) *Kcnq4*$^{+/G322S}$ mice at 4 weeks post-injection of AAV_ABE8e_N + C_322. Data were presented as mean ± SEM. Statistical analysis was performed using two-way ANOVA with Bonferroni's post hoc test. $*p < 0.05$; $**p < 0.01$; $***p < 0.001$; ns not significant (Exact *P* values are provided in Appendix Table S1).

© The Author(s)
