# Supplementary material for: Long-term restoration of auditory function in a DFNA2 mouse model by adenine base editing
Source: EMBO Mol Med. 2026 May 20;18(6):2293–321. doi: 10.1038/s44321-026-00433-5 (PMC13270111; doi:10.1038/s44321-026-00433-5)
Supplement: Supplementary file 8 — Peer Review File [file 44321_2026_433_MOESM8_ESM.pdf]

EMBO Molecular Medicine

Long-term restoration of auditory function in a DFNA2 mouse model by adenine base editing

Yuxuan Kong, Yingjie Zhang, Erjie Xie, Xiao Li, Zhuoxi Peng, Jingyuan Zhang, Yu Zhao, and Huijun Yuan

Corresponding authors: Yingjie Zhang (yingjiezhang@scu.edu.cn) , Yu Zhao (yuzhao@wchscu.edu.cn), Huijun Yuan (yuanhj301@wchscu.cn)

Review Timeline:

Submission Date:	13th Sep 25
Editorial Decision:	16th Oct 25
Revision Received:	5th Mar 26
Editorial Decision:	30th Mar 26
Revision Received:	8th Apr 26
Accepted:	13th Apr 26

Editor: Zeljko Durdevic

Transaction Report:

(Note: With the exception of the correction of typographical or spelling errors that could be a source of ambiguity, letters and reports are not edited. Depending on transfer agreements, referee reports obtained elsewhere may or may not be included in this compilation. Referee reports are anonymous unless the Referee chooses to sign their reports.)

16th Oct 2025

Dear Dr. Zhang,

Thank you for the submission of your manuscript to EMBO Molecular Medicine. We have now received feedback from the three reviewers who agreed to evaluate your manuscript. All three referees recognize interest of the study but also raise important concerns that should be addressed in a major revision. If you would like to discuss further the points raised by the referees, I am available to do so via email or video. Let me know if you are interested in this option.

Further consideration of a revision that addresses reviewers' concerns in full will entail a second round of review. EMBO Molecular Medicine encourages a single round of revision only and therefore, acceptance or rejection of the manuscript will depend on the completeness of your responses included in the next, final version of the manuscript. For this reason, and to save you from any frustrations in the end, I would strongly advise against returning an incomplete revision.

We would welcome the submission of a revised version within three months for further consideration. Please let us know if you require longer to complete the revision.

I look forward to receiving your revised manuscript.

Yours sincerely,

Zeljko Durdevic

Zeljko Durdevic
Senior Editor
EMBO Molecular Medicine

*****

When submitting your revised manuscript, please carefully review the instructions that follow below. We perform an initial quality control of all revised manuscripts before re-review; failure to include requested items will delay the evaluation of your revision.

We require:

1) A .docx formatted version of the manuscript text (including legends for main figures, EV figures and tables). Please make sure that the changes are highlighted to be clearly visible.

2) Individual production quality figure files as .eps, .tif, .jpg (one file per figure). For guidance, download the 'Figure Guide PDF': (https://www.embopress.org/page/journal/17574684/authorguide#figureformat).

3) A .docx formatted letter INCLUDING the reviewers' reports and your detailed point-by-point responses to their comments. As part of the EMBO Press transparent editorial process, the point-by-point response is part of the Review Process File (RPF), which will be published alongside your paper.

4) A complete author checklist, which you can download from our author guidelines (https://www.embopress.org/page/journal/17574684/authorguide#submissionofrevisions). Please insert information in the checklist that is also reflected in the manuscript. The completed author checklist will also be part of the RPF.

5) Please note that all corresponding authors are required to supply an ORCID ID for their name upon submission of a revised manuscript.

6) It is mandatory to include a 'Data Availability' section after the Materials and Methods. Before submitting your revision, primary datasets produced in this study need to be deposited in an appropriate public database, and the accession numbers and

database listed under 'Data Availability'. Please remember to provide a reviewer password if the datasets are not yet public (see https://www.embopress.org/page/journal/17574684/authorguide#dataavailability).

In case you have no data that requires deposition in a public database, please state so in this section. Note that the Data Availability Section is restricted to new primary data that are part of this study.

7) For data quantification: please specify the name of the statistical test used to generate error bars and P values, the number (n) of independent experiments (specify technical or biological replicates) underlying each data point and the test used to calculate p-values in each figure legend. The figure legends should contain a basic description of n, P and the test applied. Graphs must include a description of the bars and the error bars (s.d., s.e.m.). See also 'Figure Legend' guidelines: https://www.embopress.org/page/journal/17574684/authorguide#figureformat

8) At EMBO Press we ask authors to provide source data for the main manuscript figures. You will receive a separate email with instructions for providing source data with your revised manuscript, including how to upload and organize the files.

9) Our journal encourages inclusion of *data citations in the reference list* to directly cite datasets that were re-used and obtained from public databases. Data citations in the article text are distinct from normal bibliographical citations and should directly link to the database records from which the data can be accessed. In the main text, data citations are formatted as follows: "Data ref: Smith et al, 2001" or "Data ref: NCBI Sequence Read Archive PRJNA342805, 2017". In the Reference list, data citations must be labeled with "[DATASET]". A data reference must provide the database name, accession number/identifiers and a resolvable link to the landing page from which the data can be accessed at the end of the reference. Further instructions are available at .

10) We replaced Supplementary Information with Expanded View (EV) Figures and Tables that are collapsible/expandable online. A maximum of 5 EV Figures can be typeset. EV Figures should be cited as 'Figure EV1, Figure EV2" etc... in the text and their respective legends should be included in the main text after the legends of regular figures.

- For the figures that you do NOT wish to display as Expanded View figures, they should be bundled together with their legends in a single PDF file called *Appendix*, which should start with a short Table of Content. Appendix figures should be referred to in the main text as: "Appendix Figure S1, Appendix Figure S2" etc.

- Additional Tables/Datasets should be labeled and referred to as Table EV1, Dataset EV1, etc. Legends have to be provided in a separate tab in case of .xls files. Alternatively, the legend can be supplied as a separate text file (README) and zipped together with the Table/Dataset file.

See detailed instructions here:

.

11) The paper explained: EMBO Molecular Medicine articles are accompanied by a summary of the articles to emphasize the major findings in the paper and their medical implications for the non-specialist reader. Please provide a draft summary of your article highlighting

- the medical issue you are addressing,

- the results obtained and

- their clinical impact.

This may be edited to ensure that readers understand the significance and context of the research. Please refer to any of our published articles for an example.

12) Author contributions: You will be asked to provide CRediT (Contributor Role Taxonomy) terms in the submission system. These replace a narrative author contribution section in the manuscript.

13) A Conflict of Interest statement should be provided in the main text.

14) Every published paper now includes a 'Synopsis' to further enhance discoverability. Synopses are displayed on the journal webpage and are freely accessible to all readers. They include a short stand first (maximum of 300 characters, including space) as well as 2-5 one-sentences bullet points that summarizes the paper. Please write the bullet points to summarize the key NEW findings. They should be designed to be complementary to the abstract - i.e. not repeat the same text. We encourage inclusion of key acronyms and quantitative information (maximum of 30 words / bullet point). Please use the passive voice. Please attach these in a separate file or send them by email, we will incorporate them accordingly.

Please also suggest a striking image or visual abstract to illustrate your article as a PNG file 550 px wide x 300-600 px high.

15) Include a Reagents and Tools Table as part of the Methods section, which can be downloaded from our author guidelines (https://www.embopress.org/page/journal/17574684/authorguide#structuredmethods)

EMBO Molecular Medicine has a "scooping protection" policy, whereby similar findings that are published by others during review or revision are not a criterion for rejection. Should you decide to submit a revised version, I do ask that you get in touch after three months if you have not completed it, to update us on the status.

***** Reviewer's comments *****

Referee #1 (Comments on Novelty/Model System for Author):

In this study, the authors created a mutant mouse model for DFNA2 (Kcnq4+/G322S), which developed early-onset hearing loss by 4 weeks of age. Significant OHC loss was found. The authors then tested several base editors in vitro to assess their ability to target the c.964G>A mutation this mutant mouse strain and found that the ABE8e base editor has the best ability to target this mutation. They used the dual-AAV approach to deliver the ABE8e adenine base editor to the inner ears of the Kcnq4+/G322S mutant mice at P2-P3 in vivo. Two viral titers were tested (3.64e10 vs 1.3e10 gc/mouse). They found that while the mutant mice that were treated with high-dose gene therapy showed greater hearing recovery initially at 4 weeks, the hearing deteriorated more quickly compared to those mutant mice that were treated with low-dose gene therapy. Gene therapy treatment also led to the improvement of HC and SGN survival. In addition, the OHCs from treated mutant mice also showed a significant increase in IKn density and restoration of the transient inward K+ current during hyperpolarization. I think this is a nice study which utilized base editor successfully to partially improve the auditory function in a new mouse model of DFNA2.

Referee #1 (Remarks for Author):

My comments are listed below:
1. The authors quantified the OHC loss in the heterozygous and homozygous mutant-s (lines 132-135). The OHC loss for the basal turn in the heterozygous animals was not included. Was there any OHC loss in the heterozygous mice at the basal turn of the cochlea?
2. The authors quantified the cDNA correction efficiency of the ABE8e gene therapy in the Kcnq4+/G322S mutant mice after in vivo delivery (lines 190-192). What is cDNA correction efficiency? How was this quantified?
3. One interesting finding from this study is the fact that the mutant mice treated with low-dose gene therapy showed better long-term hearing recovery than those animals treated with high-dose gen therapy. The authors postulated that this may be due to the increased inflammatory response generated by the high-dose gene therapy. Some supporting data are shown in Figure 7. However, when wildtype mice were injected with high-dose ABE8e gene therapy, hearing loss was not detected (Figure 3D). Was the increase in inflammation also observed in the WT mice? It would be useful to include images from WT mice in Figure 7 as an additional control.
4. The data shown in Figures 4 and 5 are from mutant mice that received high-dose gene therapy. Did the mutant mice that receive low-dose gene therapy also show similar improvement in HC and SGN survival and restoration of potassium current? This is especially important since the mutant mice in the low-dose group had better hearing recovery long term.
5. In Figure 5D, how was the SGN survival quantified? The areas of Rosenthal's canal shown in the confocal images are consistently smaller in the untreated mice than the other two groups. I would suggest the authors count SGNs in a specified area which will make the counts more consistent and non-biased.
6. In Figure 5F, the authors showed that the treated mutant mice had increased afferent/efferent nerve fibers compared to non-treated mutants. Yet, the images seem to suggest that at the apex and middle turns of the cochlea, the non-treated mutant seem to have greater number of the afferent/efferent nerve fibers compared to the treated mutants.
7. In figure 6, how did the authors know which OHCs in the treated mutant mice were transduced with AAVs and underwent successful base editing? The sample sizes provided in the figure legend, do they represent the number of mice used for each experiment or the number of OHCs recorded?

Referee #2 (Comments on Novelty/Model System for Author):

appropriate mouse model, straight forward for first line of preclinical proof of concept

Referee #2 (Remarks for Author):

EMBO Molecular Medicine - Referee Report

Title: Base editing rescue of dominant Kcnq4 p.G322S hearing loss in mice (Kong et al.)

Summary & significance

Kong et al. report dual-AAV delivery of split ABE8e to correct a dominant Kcnq4 p.G322S mutation that models human DFNA2 (KCNQ4 p.G321S). In the organ of Corti they achieve approximately 22-30% genomic correction (peaks around 40%) and approximately 25-44% cDNA correction within three weeks, alongside sustained functional rescue: best ABR threshold gains reach about 49 dB at 32 kHz, with low-dose effects persisting to 32 weeks. Morphology and physiology corroborate rescue (hair cells, spiral ganglion neurons, afferent/efferent fibers; partial KCNQ4 current restoration in outer hair cells). The work substantially advances mutation-correction strategies for dominant hearing loss and is likely of high interest to the field.

Major strengths

• Robust in vivo editing for the inner ear with clear benchmarking against prior rates, and careful quantification at DNA and cDNA levels.
• functional data (4-32 kHz ABR/DPOAE)
• Dose-response insight (early high-dose benefit but faster decline; superior durability at low dose).
• Anatomical/physiological correlates (hair cells/spiral ganglion neuron survival; nerve fiber density; outer hair cell KCNQ4 current).
• Initial safety/off-target assessment, including predicted DNA off-targets (HTS, GUIDE-seq) and transcriptome-wide RNA A-to-I analysis.

Overall recommendation

Major revision. The study is exciting and timely, with convincing efficacy. Addressing the points above-especially a deeper, data-driven analysis of why rescue is limited (co-transduction/reconstitution, editing mosaicism, disease kinetics), strengthening off-target/biodistribution safety, and reporting/availability-will substantially enhance the manuscript's rigor and translational value.

Major points

1) Scope and mechanism of the incomplete/limited rescue

The functional gains are impressive but not complete or uniform over time and frequency. The Discussion touches on delay vs. durability and hair cell degeneration, but the manuscript would benefit from a deeper, quantitative analysis of potential limiting steps:

• Vector biology & delivery
- Despite excellent reporter transduction (inner hair cell > 98%; outer hair cell 86-99% by turn), editing efficiencies at the target locus are lower; please discuss bottlenecks beyond cell entry (e.g., AAV uncoating, nuclear entry, expression timing).
- Clarify co-transduction and intein reconstitution rates in vivo for N + C halves (split ABE). In vitro, splitting reduced activity by about 21%. Can you estimate cochlear N ∩ C co-delivery/recombination from dual-fluor or barcoded reporters, or reference literature rates, to help explain the gap between transduction and editing? Also please compare to other dual-AAV approaches
• Editing pharmacodynamics
- ideally provide a timeline model for onset of editor expression vs. progression of hair cell loss, especially in basal vs apical turns. This could explain the delayed yet durable low-dose rescue and why early cell loss may be irreversible before editing acts.
- If available, include longitudinal editing levels beyond 3 weeks to relate molecular correction to the ABR/DPOAE trajectories (weeks 4-32).
• Allelic context & mosaicism
- In heterozygotes the DNA/cDNA correction is modest (e.g., roughly 8-17% DNA, 2-8% cDNA above baseline). Please discuss how much cell-level correction is required to overcome dominant-negative effects and whether mosaic correction in critical nodes (e.g., basal outer hair cells) suffices for large ABR effects. Moreover, how could rescuing some hair cell might or might not slow the degenerative process
• OHC functional rescue: Kharkovets et al., 2006 report on a depolarized resting potential of outer hair cells and argue that function of OHCs is hampered because of this before OHC loss due degeneration because of chronic depolarization. I think the manuscript would benefit from reporting some current-clamp measurements of OHC resting potential in WT, Mut and Mut+rescue
• Tonotopic analysis
- Because DFNA2 begins basally, a brief turn-resolved correlation (editing vs. transduction vs. hair cell survival vs. thresholds) would strengthen the mechanistic link between tonotopic vulnerability and rescue magnitude.

2) Dose-dependent decline and neuro-immune responses

The high-dose cohort shows earlier improvement but accelerated decline from around 16 weeks, whereas low-dose sustains gains. The cytokine/microglia/macrophage data suggest greater local immune activation at higher dose. Please:

• Expand on causality (editing toxicity vs. AAV load vs. editor overexpression). Could hit-and-run expression (self-limiting vectors, protein/RNP delivery) mitigate late decline?
• Include any hair-cell counts and synaptic metrics at late endpoints for high- vs low-dose to link deterioration to pathology.
• Discuss aspects relevant for future clinical translation: dose selection and immune-modulatory strategies for the clinic.

3) Off-target and biodistribution-request deeper analysis

The DNA off-target panel based on predicted sites was clean in cochlea and brain, but RNA-seq revealed dose-dependent A-to-I RNA editing. A few additions would substantially strengthen the safety case:

• Report genome-wide DNA off-targets beyond the predicted/GUIDE-seq panel if feasible (e.g., CHANGE-seq, DISCOVER-seq,

CIRCLE-seq), or explicitly justify the current panel's coverage.
• Provide annotation of RNA edits (exonic vs. UTR/intronic; predicted consequence; overlap with ADAR hotspots) and an estimate of effect sizes at edited loci.
• Because AAV-ie reporter showed expression in hippocampus and mild heart/liver, please clarify whether ABE components (not only EGFP) show extra-cochlear expression and whether off-target assays were performed in those tissues at the editor target sequence.
• State whether germline tissues were examined (or why not) given neonatal injections.
4) Statistics, design, and reporting
• You note blinding for ABR/DPOAE thresholding; please explicitly report randomization, litter/ear as unit of analysis, sex as a biological variable, and how you handled capping (ABR > 90 dB set to 100 dB) in statistical tests.
• For longitudinal analyses, specify mixed-effects models or repeated-measures ANOVA choices and multiple-comparison control.
5) Data availability
The current "available upon reasonable request" policy is below community expectations for a methods-heavy gene-editing study. Please deposit raw HTS/CRISPResso2 outputs, GUIDE-seq data, and RNA-seq FASTQs in public repositories with accession numbers included at acceptance (technical hold until accession numbers or active links are provided).
Minor points and clarity edits
• Clarify whether bystander edits were fully excluded in vivo at the on-target window (you note none in vitro). Provide in vivo amplicon spectra.
• Include a simple schematic of editing window and PAM for the murine and human target to contextualize sgRNA choice and the two synonymous differences in the human guide.
• Briefly compare editor classes (ABE8e vs newer compact/base-deaminase variants) for payload/precision trade-offs relevant to possible clinical translation.
• In Methods, add exact ABR/DPOAE parameter values in a supplementary table for reproducibility (stimulus timing, artifact rejection, and threshold criteria already described).
Required citations to add:
• Kubisch et al., Cell, 1999 - cloning of KCNQ4 and mapping to DFNA2 (original description).
• Coucke et al., Human Molecular Genetics, 1999 - pathogenic KCNQ4 mutations in DFNA2 families.
• Al-Moyed et al., EMBO Molecular Medicine, 2018 - first proof-of-concept dual-AAV rescue of otoferlin in inner hair cells of Otof knockout mice.
• Vona et al., MedComm, 2025 - recent overview of preclinical and clinical inner-ear gene therapy studies.

Referee #3 (Comments on Novelty/Model System for Author):

Preclinical mouse models for human deafness are essential before considering moving to non-human primates and/or human clinical trials.

Referee #3 (Remarks for Author):

The work demonstrated base editing on a mouse model for human KCNQ4. A dual-AAV delivery approach with the ABE8e adenine base editor led to a correction efficiency of 22.2-29.8%. Given the promise of CRISPR base editing for gene therapy for multiple diseases, it is critical that this work be done on relevant mouse models for human deafness. A major challenge is lasting auditory restoration, to achieve a long duration for therapy in humans. The authors chose to attempt such therapy for an autosomal dominant form of deafness, KCNQ4, responsible for approximately 9.5% of this form of hearing loss. The results led to improved auditory threshold for at least 32 weeks, with a delay in degeneration of hair cells, spiral ganglion neurons, and afferent and efferent nerve fibers.

After screening their deafness cohort, a number of KCNQ4 variants were identified. A homozygous mouse model for one of these, c.964G>A (p.G322S), was created using CRISPR/Cas9-mediated knock-in technology, demonstrating hearing loss. A thorough study was done to evaluate the restoration of hearing in the mutants following high or low-dose AAV_ABE8e_N+C_322 or an empty AAV, including immunofluorescence for cochlear morphology, whole-cell voltage–clamp recordings, and a safety evaluation of inflammation and macrophage recruitment. Most compelling, with only an ~25% correction, the authors showed a dramatic ABR recovery (~50 dB), which was very efficient. The histology, immunofluorescence and electrophysiology all match well with this recovery.

Timing is critical and while Kcnq4 expression in OHCs begins at P8, there was a successful window of intervention seen from P9-14.

The in vitro screening of the ABE systems and sgRNAs was straightforward. For the in vivo correction, the viral dose was reasonable and matches an appropriate range (up to 5x10^10 vg/gc per mouse at p0).

In the GFP and controls, the authors showed that dual AAV injection does not impair hearing in wild-type mice, and that AAV-ie-CMV-EGFP injection resulted in GFP expression in the tissue. Have control groups been done, either the GFP vector alone or PBS/saline, to show no effect to controls and not only in untreated mice? If done, it would be relevant to show in supplementary data. This is, however, not essential moving forward.

In the off-target analysis, the authors saw a dose-dependent off-target RNA, though not DNA, editing. This finding has clinical implications and needs to be evaluated if this technique moves to the clinic with regards to safety. It is exactly for this reason that this preclinical model is essential.

The high-dose group exhibited accelerated auditory threshold deterioration. Do the authors predict that the decline is due to immunologic (AAV tropism, macrophage infiltration), off-targets, or bystander edits?
Injections were on neonates and in the future, a juvenile or adult cohort of mice would add to the clinical relevance.
Finally, this is the most successful report of Kcnq4 base editing and correction of hearing in a mouse model for this human form of autosomal dominant.

Essential to change:
In Figure 3A, use the word SACRIFICE, and not KILL

Point-by-Point Response

Response to the editor

When submitting your revised manuscript, please carefully review the instructions that follow below. We perform an initial quality control of all revised manuscripts before re-review; failure to include requested items will delay the evaluation of your revision.

Responese: We sincerely thank the editor for the careful evaluation of our manuscript. We have revised the manuscript and incorporated additional experiments and discussions in accordance with the comments and suggestions provided by the editor and the reviewers. The specific revisions are detailed as follows.

We require:

1) A .docx formatted version of the manuscript text (including legends for main figures, EV figures and tables). Please make sure that the changes are highlighted to be clearly visible.

Responese: Done. We have revised the manuscript text, including the legends for the main figures, EV figures, and tables, and have highlighted all modifications in yellow for clarity.

2) Individual production quality figure files as .eps, .tif, .jpg (one file per figure). For guidance, download the 'Figure Guide PDF': (https://www.embopress.org/page/journal/17574684/authorguide#figureformat).

Responese: Done. In the revised version, we have uploaded high-resolution figures in TIFF format.

3) A .docx formatted letter INCLUDING the reviewers' reports and your detailed point-by-point responses to their comments. As part of the EMBO Press transparent editorial process, the point-by-point response is part of the Review Process File (RPF), which will be published alongside your paper.

Responese: Done. The point-by-point response to the reviewers is provided in the following sections of this document.

4) A complete author checklist, which you can download from our author guidelines (https://www.embopress.org/page/journal/17574684/authorguide#submissionofrevisions). Please insert information in the checklist that is also reflected in the manuscript. The completed author checklist will also be part of the RPF.

Responese: Done. The author checklist has been completed and submitted along with this revision.

5) Please note that all corresponding authors are required to supply an ORCID ID for their name upon submission of a revised manuscript.

Responese: Done. All corresponding authors have provided their ORCID ID.

6) It is mandatory to include a 'Data Availability' section after the Materials and Methods. Before submitting your revision, primary datasets produced in this study need to be deposited in an appropriate public database, and the accession numbers and database listed under 'Data Availability'. Please remember to provide a reviewer password if the datasets are not yet public (see https://www.embopress.org/page/journal/17574684/authorguide#dataavailability).

In case you have no data that requires deposition in a public database, please state so in this section. Note that the Data Availability Section is restricted to new primary data that are part of this study.

Responese: Done. The relevant content has been updated in the Data Availability section.

7) For data quantification: please specify the name of the statistical test used to generate error bars and P values, the number (n) of independent experiments (specify technical or biological replicates) underlying each data point and the test used to calculate p-values in each figure legend. The figure legends should contain a basic description of n, P and the test applied. Graphs must include a description of the bars and the error bars (s.d., s.e.m.). See also 'Figure Legend' guidelines: https://www.embopress.org/page/journal/17574684/authorguide#figureformat

Responese: Done. The statistical information is provided in the figure legends.

8) At EMBO Press we ask authors to provide source data for the main manuscript figures. You will receive a separate email with instructions for providing source data with your revised manuscript, including how to upload and organize the files.

Responese: Done. The source data for the main figures have been submitted along with this revision.

9) Our journal encourages inclusion of *data citations in the reference list* to directly cite datasets that were re-used and obtained from public databases. Data citations in the article text are distinct from normal bibliographical citations and should directly link to the database records from which the data can be accessed. In the main text, data citations are formatted as follows: "Data ref: Smith et al, 2001" or "Data ref: NCBI Sequence Read

Archive PRJNA342805, 2017". In the Reference list, data citations must be labeled with "[DATASET]". A data reference must provide the database name, accession number/identifiers and a resolvable link to the landing page from which the data can be accessed at the end of the reference. Further instructions are available at <https://www.embopress.org/page/journal/17574684/authorguide#referencesformat>.

Responese: Thank you for the editor's suggestion. We did not use any publicly available datasets in this study.

10) We replaced Supplementary Information with Expanded View (EV) Figures and Tables that are collapsible/expandable online. A maximum of 5 EV Figures can be typeset. EV Figures should be cited as 'Figure EV1, Figure EV2" etc... in the text and their respective legends should be included in the main text after the legends of regular figures.
- For the figures that you do NOT wish to display as Expanded View figures, they should be bundled together with their legends in a single PDF file called *Appendix*, which should start with a short Table of Content. Appendix figures should be referred to in the main text as: "Appendix Figure S1, Appendix Figure S2" etc.
- Additional Tables/Datasets should be labeled and referred to as Table EV1, Dataset EV1, etc. Legends have to be provided in a separate tab in case of .xls files. Alternatively, the legend can be supplied as a separate text file (README) and zipped together with the Table/Dataset file.
See detailed instructions here:
<https://www.embopress.org/page/journal/17574684/authorguide#expandedview>.

Response: Done. Figures have been submitted with this revision, and the corresponding citations in the main text have been updated accordingly.

11) The paper explained: EMBO Molecular Medicine articles are accompanied by a summary of the articles to emphasize the major findings in the paper and their medical implications for the non-specialist reader. Please provide a draft summary of your article highlighting
- the medical issue you are addressing,
- the results obtained and
- their clinical impact.

Response：Done. We have prepared the following content in accordance with the editor's request.
Medical issue: Gene therapies have emerged as a transformative approach for treating monogenic disorders. Hereditary hearing loss, the most prevalent genetic sensory disorder, currently has no approved pharmacological treatments, making it an ideal target for gene-based interventions. Pathogenic variants in *KCNQ4* underlie roughly 9.5% of autosomal dominant nonsyndromic hearing loss (DFNA2). Although previous attempts have aimed to disrupt mutant *Kcnq4* alleles, long-lasting restoration of auditory function has remained elusive. Here, we explored a mutation-correction strategy to determine

whether precise gene editing could achieve more robust and sustained hearing recovery in a DFNA2 mouse model.

Result: Using dual AAV vectors to deliver the ABE8e base editor, we achieved highly efficient correction of *Kcnq4* mutations in mice. This intervention led to substantial and sustained restoration of auditory function for at least 32 weeks, accompanied by preservation of hair cell, spiral ganglion neuron, and nerve fiber structure, as well as enhanced electrophysiological function of outer hair cells. Importantly, we observed a dose-dependent safety profile: mice receiving higher doses exhibited accelerated hearing decline at later stages compared with lower-dose groups. These findings underscore the critical importance of dose optimization for achieving durable efficacy while maintaining safety in inner ear gene editing.

Clinical Impact: These results provide a compelling proof-of-concept for the use of base editing to treat DFNA2 hereditary hearing loss. They also highlight the crucial role of dose selection in gene therapy, offering key guidance to ensure long-term safety and efficacy, and laying the groundwork for future clinical translation.

12) Author contributions: You will be asked to provide CRediT (Contributor Role Taxonomy) terms in the submission system. These replace a narrative author contribution section in the manuscript.

Response: Done. In accordance with the requirements, we have revised the description of the Author Contributions.

13) A Conflict of Interest statement should be provided in the main text.

Response: Done.

14) Every published paper now includes a 'Synopsis' to further enhance discoverability. Synopses are displayed on the journal webpage and are freely accessible to all readers. They include a short stand first (maximum of 300 characters, including space) as well as 2-5 one-sentences bullet points that summarizes the paper. Please write the bullet points to summarize the key NEW findings. They should be designed to be complementary to the abstract - i.e. not repeat the same text. We encourage inclusion of key acronyms and quantitative information (maximum of 30 words / bullet point). Please use the passive voice. Please attach these in a separate file or send them by email, we will incorporate them accordingly.

Response: Thank you for the editor's reminder. We have prepared the following 'Synopsis' to further enhance discoverability.

Auditory function was successfully restored in a Kcnq4 p.G322S mouse model of DFNA2 following the delivery of the adenine base editor ABE8e via dual-AAV vectors.

1. A genomic correction efficiency of approximately 21%–29% was achieved in homozygous mice using the ABE8e base editor.

2. Auditory function in heterozygous mice was restored and maintained for a sustained period of at least 32 weeks post-treatment.

3. Structural preservation of hair cells, spiral ganglion neurons, and nerve fibers was observed and accompanied by improved electrophysiological function of OHC.

4. A dose-dependent efficacy profile was identified, in which accelerated decline was noted at high doses while durable efficacy was obtained with low-dose treatment.

Please also suggest a striking image or visual abstract to illustrate your article as a PNG file 550 px wide x 300-600 px high.

Response: Done. We have provided a visual abstract to illustrate our article.

15) Include a Reagents and Tools Table as part of the Methods section, which can be downloaded from our author guidelines (https://www.embopress.org/page/journal/17574684/authorguide#structuredmethods)

Response: Done. The Reagents and Tools Table has been inserted into the Methods section.

EMBO Molecular Medicine has a "scooping protection" policy, whereby similar findings that are published by others during review or revision are not a criterion for rejection. Should you decide to submit a revised version, I do ask that you get in touch after three months if you have not completed it, to update us on the status.

Response: We would like to thank the editor once again for reviewing our manuscript. We hope that the revised version meets the expectations of both the editor and the reviewers.

Response to the reviewers

Note: In this response, the reviewers' comments and questions are presented in **black**, while responses are in **blue.** Significant modifications and key points have been **highlighted in yellow** for ease of reference.

Referee #1 (Comments on Novelty/Model System for Author):

In this study, the authors created a mutant mouse model for DFNA2 (Kcnq4+/G322S), which developed early-onset hearing loss by 4 weeks of age. Significant OHC loss was

found. The authors then tested several base editors in vitro to assess their ability to target the c.964G>A mutation this mutant mouse strain and found that the ABE8e base editor has the best ability to target this mutation. They used the dual-AAV approach to deliver the ABE8e adenine base editor to the inner ears of the Kcnq4+/G322S mutant mice at P2-P3 in vivo. Two viral titers were tested (3.64e10 vs 1.3e10 gc/mouse). They found that while the mutant mice that were treated with high-dose gene therapy showed greater hearing recovery initially at 4 weeks, the hearing deteriorated more quickly compared to those mutant mice that were treated with low-dose gene therapy. Gene therapy treatment also led to the improvement of HC and SGN survival. In addition, the OHCs from treated mutant mice also showed a significant increase in IKn density and restoration of the transient inward K+ current during hyperpolarization. I think this is a nice study which utilized base editor successfully to partially improve the auditory function in a new mouse model of DFNA2.

Response：We thank the reviewer’ comments.

Referee #1 (Remarks for Author):

My comments are listed below:

1. The authors quantified the OHC loss in the heterozygous and homozygous mutant-s (lines 132-135). The OHC loss for the basal turn in the heterozygous animals was not included. Was there any OHC loss in the heterozygous mice at the basal turn of the cochlea?

Response：We thank the reviewer for the insightful inquiry and apologize for any ambiguity in our initial description that may have caused confusion. At the 4-week time point, the number of OHCs in the basal turn of heterozygous mice did not show a statistically significant reduction compared with wild-type controls ($P = 0.5250$; revised Fig. 1K, M). This morphological finding is consistent with our ABR data at the same stage, which indicate that high-frequency hearing is less affected than mid- to low-frequency hearing (revised Fig. 1B). These results suggest that the p.G322S mutation may initially impair OHC function without causing overt OHC loss in the basal turn at this early stage.

With increasing age, however, we observed a significant reduction in OHC numbers across the apical, middle, and basal turns in heterozygous mice at 8 weeks of age, and IHC loss was also detectable (revised Fig. 5A–C). Together, these findings support a time-dependent and progressive hair cell degeneration in Kcnq4+/G322S mutant mice, consistent with the progressive nature of DFNA2-associated hearing loss, and suggest that the absence of significant basal-turn OHC loss at 4 weeks reflects an early stage of pathology rather than preservation of hair cell integrity.

The corresponding revisions have also been incorporated into the lines 137-138 of the manuscript.

2. The authors quantified the cDNA correction efficiency of the ABE8e gene therapy in the Kcnq4+/G322S mutant mice after in vivo delivery (lines 190-192). What is cDNA

correction efficiency? How was this quantified?

Response: We thank the reviewer for this pertinent question. Since Kcnq4 mRNA is expressed exclusively in cells that naturally transcribe the gene, we believe that assessing editing efficiency at the transcript level (via cDNA) offers a more precise measure of base editing outcomes within the relevant target cells. To quantify this, we determined the absolute cDNA correction efficiency by subtracting the background G percentage in the control group from the G ratio in the treated group [i.e., (G/(A+G))%Treated - (G/(A+G))%Control]. This absolute increase in the G signal was utilized as the final metric for A-to-G correction efficiency at the transcriptional level. Additionally, in our initial quantification of homozygous editing efficiency, the negligible baseline noise in control groups allowed for the direct reporting of G/(A+G) % (Xue *et al*, 2023). However, to maintain methodological uniformity throughout the study and rigorously mitigate potential sequencing artifacts, we have now standardized the calculation of homozygous efficiency by adopting the normalization criteria established for heterozygous editing. This refinement further strengthens the scientific rigor and analytical robustness of our reporting without altering any study conclusions. A detailed clarification has been incorporated into the Methods section (Lines 790–793) to ensure transparency and avoid ambiguity.

3. One interesting finding from this study is the fact that the mutant mice treated with low-dose gene therapy showed better long-term hearing recovery than those animals treated with high-dose gen therapy. The authors postulated that this may be due to the increased inflammatory response generated by the high-dose gene therapy. Some supporting data are shown in Figure 7. However, when wildtype mice were injected with high-dose ABE8e gene therapy, hearing loss was not detected (Figure 3D). Was the increase in inflammation also observed in the WT mice? It would be useful to include images from WT mice in Figure 7 as an additional control.

Response: We thank the reviewer for the insightful question and apologize for the lack of clarity in our initial description. In fact, WT mice were utilized for the evaluation of inflammatory responses to minimize potential observational bias resulting from pre-existing structural abnormalities in the mutant model.Additionally, as there is currently no clear evidence suggesting that KCNQ4 mutations modulate the immune landscape of the inner ear, the utilization of WT mice remains a scientifically sound and appropriate approach. For better clarity, the following modifications have been implemented in revised Figure 7D.

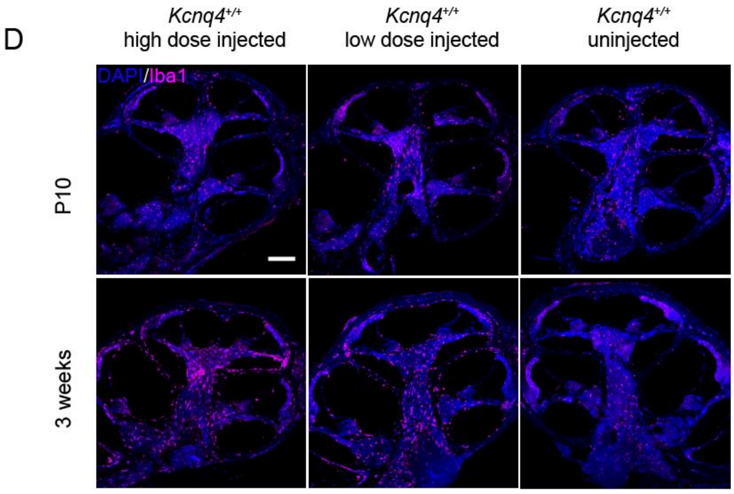

Figure 7. (D) Representative immunofluorescence images of cochlear macrophages at P10 and 3 weeks post-injection in *Kcnq4*$^{+/+}$ mice receiving low or high doses of AAV_ABE8e_N+C_322 .

Safety assessments following high-dose injections were performed at 4 weeks post-injection (revised Fig. 3D). At this early time point, no significant adverse effects on hearing were observed in Kcnq4+/+ mice. In the Kcnq4+/G332S model, high-dose treatment even conferred superior therapeutic efficacy compared to the low-dose group within the first 8 weeks (revised Fig4. A, B). However, longitudinal monitoring revealed a progressive decline in auditory function in the high-dose cohort, with hearing outcomes becoming worse than those of the low-dose group by 16 weeks (revised Fig. EV5B). These findings indicate that comprehensive safety and toxicity evaluations for inner ear gene therapy should include extended follow-up periods to detect potential late-onset adverse effects.

4. The data shown in Figures 4 and 5 are from mutant mice that received high-dose gene therapy. Did the mutant mice that receive low-dose gene therapy also show similar improvement in HC and SGN survival and restoration of potassium current? This is especially important since the mutant mice in the low-dose group had better hearing recovery long term.

Response : We thank the reviewer for these constructive suggestions. In response, we re-administered a low dose of AAV and allowed an 8-week follow-up period. We subsequently performed additional morphological analyses and electrophysiological recordings in heterozygous mutant mice after low-dose treatment. The results are summarized below. As shown in the revised Figure 5A–C, low-dose treated heterozygous mice displayed substantial improvements in OHC survival at 8 weeks of age. Compared with untreated controls, OHC counts increased by 0.67-, 1.25-, and 4.3-fold in the apical, middle, and basal turns, respectively, and did not differ significantly from those in the high-dose group. Although IHC survival in the low-dose group did not reach statistical significance compared with untreated group, a clear upward trend was evident. Our

observations demonstrate that, at the 8-week time point, the protective effects on hair cells were largely comparable between the low- and high-dose groups. This finding is consistent with the reduced difference observed between the two dosing regimens in terms of hearing preservation at this stage. Collectively, these results indicate that the superior long-term hearing protection observed in the low-dose group is unlikely to be attributable to differences in early post-treatment hair cell survival. The above results are described in detail in the revised manuscript (lines 300–313).

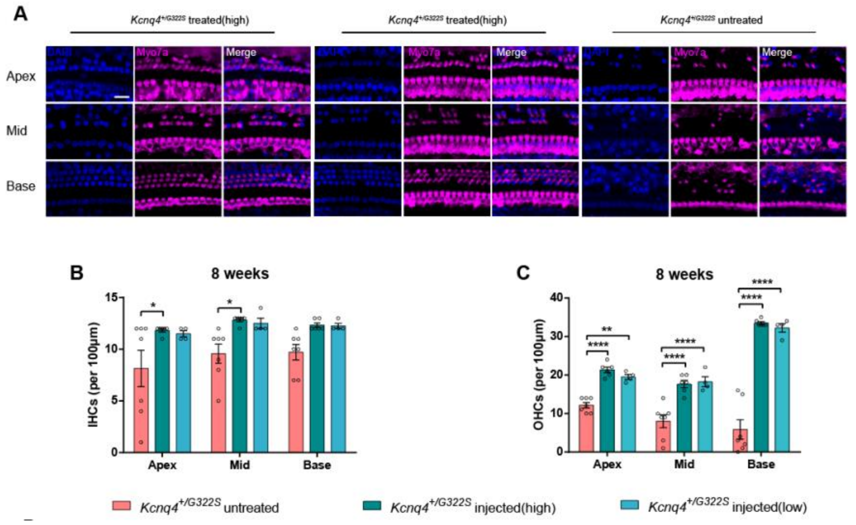

Figure 5. (A) Representative immunofluorescence images of HCs in the apical, middle, and basal turns of the cochleae from treated (3.64 × 10^10 gc/mouse and 1.3 × 10^10 gc/mouse) and untreated *Kcnq4*$^{+/G322S}$ mice. Scale bar, 20 μm. (B and C) Quantification of IHCs (B) and OHCs (C) per 100 μm in treated (high dose n=6, low dose n=4) and untreated (n = 7) *Kcnq4*$^{+/G322S}$ mice. Data are presented as mean ± SEM. Statistical analysis was performed using two-way ANOVA with Bonferroni's post hoc test. *$p < 0.05$; **$p < 0.01$; ***$p < 0.001$; ****$p < 0.0001$; ns: not significant.

We also re-administered the treatment and assessed the effects of low-dose therapy on spiral ganglion neuron (SGN) survival. To ensure methodological rigor, identical anatomical regions were selected across all groups for SGN quantification. Furthermore, to eliminate potential bias arising from differences in sampled area size, we replaced raw SGN counts with SGN density measurements (expressed as SGNs/mm²). This strategy improves data accuracy and enhances comparability across groups. Consistent with our previous observations in the high-dose group, as shown in the revised Figure 5D and E, at 8 weeks of age, SGN density in heterozygous mutant mice was significantly reduced by 35%, 21%, and 23% in the apical, middle, and basal turns, respectively, compared with WT littermates. Notably, SGN density in both the high- and low-dose treatment groups did not differ significantly from that of the WT group, confirming that both treatment regimens effectively preserve SGNs at this time point. The above results are described in detail in the revised manuscript (lines 319–320).

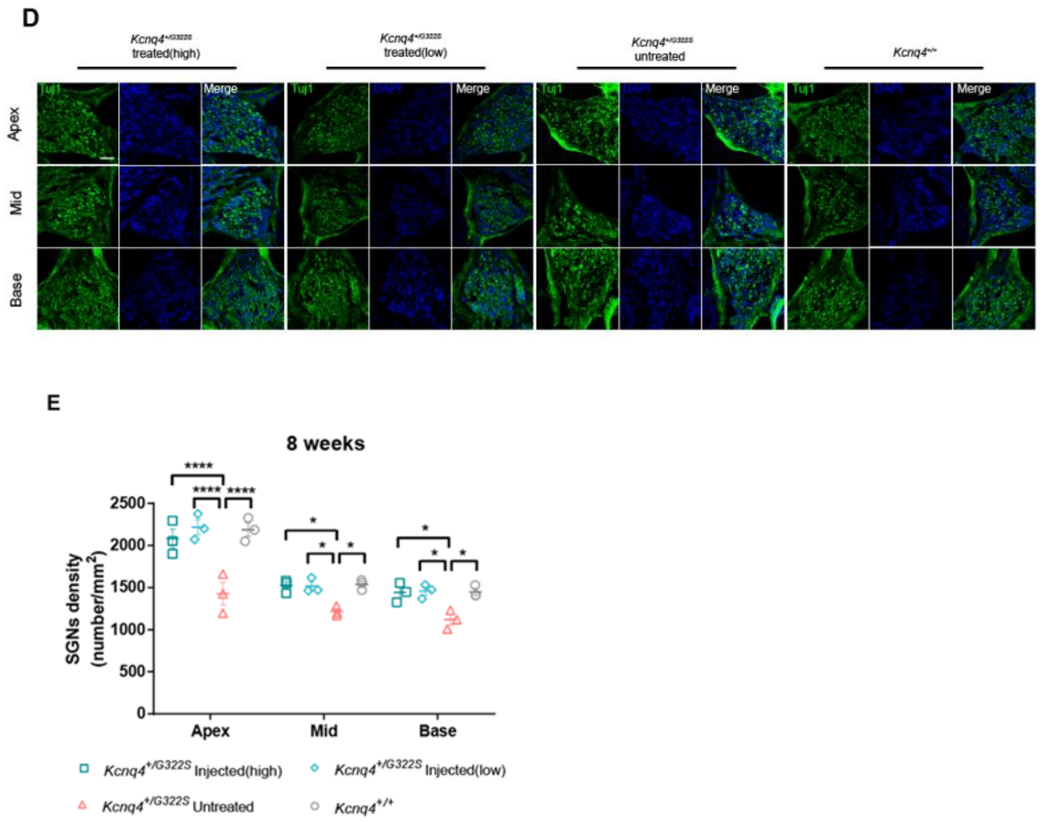

Figure 5. (D) Representative immunofluorescence images of SGNs in the apical, middle, and basal turns of the cochleae sections from *Kcnq4*$^{+/+}$, treated (3.64 × 10^10 gc/mouse and 1.3 × 10^10 gc/mouse), and untreated *Kcnq4*$^{+/G322S}$ mice. Scale bar, 50 µm. (E) Quantification of SGN density in cochlear sections in (D) from *Kcnq4*$^{+/+}$ (n = 3), treated (high dose n = 3, low dose n=3), and untreated (n = 3) *Kcnq4*$^{+/G322S}$ mice. Data are presented as mean ± SEM. Statistical analysis was performed using two-way ANOVA with Bonferroni's post hoc test. *$p < 0.05$; **$p < 0.01$; ***$p < 0.001$; ****$p < 0.0001$; ns: not significant.

During potassium current recordings in OHCs from heterozygous mutant mice following low-dose treatment (P9–P14), the overall current amplitude in the low-dose group was lower than that observed in the high-dose group. Nevertheless, a distinct IK,n current was clearly detectable in the low-dose group during hyperpolarizing steps (revised Figure 6B). Quantification of the peak current at −140 mV (revised Figure 6D) revealed a significant increase of approximately 87% in the low-dose group compared with untreated controls, indicating partial restoration of potassium currents following low-dose treatment. The above results are described in detail in the revised manuscript (lines 348–354).

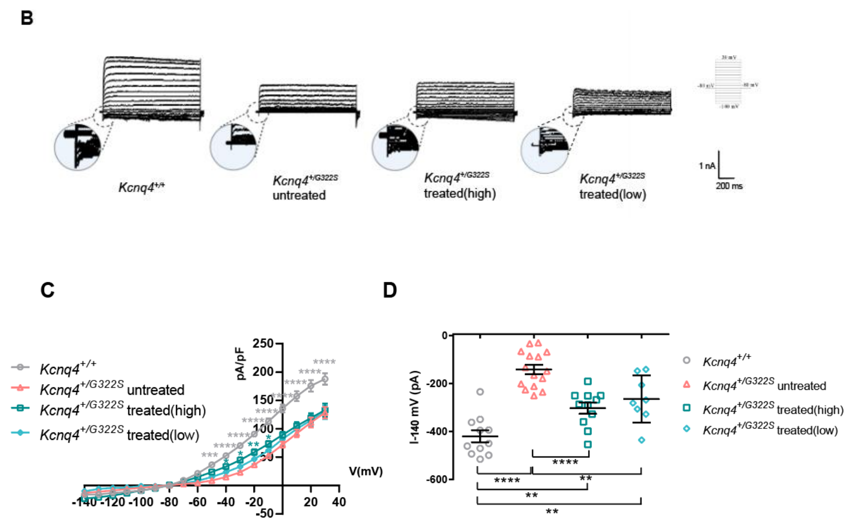

Figure 6. (B) Representative current traces from *Kcnq4*$^{+/+}$ controls, treated (3.64 × 1010 gc/mouse and 1.3 × 1010 gc/mouse), and untreated *Kcnq4*$^{+/G322S}$ mice. The transient inward current peak at -140 mV is highlighted. (C) Peak current−voltage (I−V) curves illustrating Kcnq4 channel function in *Kcnq4*$^{+/+}$ controls (n = 11 cells from 8 mice), treated (high dose n = 11 cells from 8 mice, low dose n=8 cells from 7 mice), and untreated (n = 15 cells from 10 mice) *Kcnq4*$^{+/G322S}$ mice. Statistical comparisons were made between treated and untreated *Kcnq4*$^{+/G322S}$ mice (dark teal for high-dose group, cyan for low-dose group) and between *Kcnq4*$^{+/+}$ and untreated *Kcnq4*$^{+/G322S}$ mice (gray). (D) Quantification of peak transient inward current at -140 mV in *Kcnq4*$^{+/+}$ controls (n = 11 cells from 8 mice), treated (high dose n = 11 cells from 8 mice, low dose n=8 cells from 7 mice), and untreated (n = 15 cells from 10 mice) *Kcnq4*$^{+/G322S}$ mice. Data are presented as mean ± SEM. Statistical analysis was performed using two-way ANOVA with Bonferroni's post hoc test. *$p < 0.05$; **$p < 0.01$; ***$p < 0.001$; ****$p < 0.0001$; ns: not significant.

Given that KCNQ4 mutations may disrupt membrane polarization, potentially leading to chronic depolarization, functional impairment, and ultimately cell death, we have incorporated an analysis of the resting membrane potential (RMP) in OHCs from treated and untreated heterozygous mutant mice. We found that the RMP of untreated mutant OHCs was significantly more depolarized than that of wild-type controls, with an increase of approximately 25%, confirming the deleterious impact of the mutation on cellular membrane polarization. Notably, high-dose treatment resulted in a significant rescue of the RMP, inducing an approximately 17% hyperpolarizing shift relative to untreated controls. In contrast, the low-dose group exhibited only a modest hyperpolarizing trend that did not reach statistical significance (revised Figure 6E). Given that P9–P14 represents an early developmental window, the full functional impact of the low-dose regimen may not yet be realized, raising the possibility of greater recovery at later stages. The above findings are described in detail in the revised manuscript (lines 354–358).

E

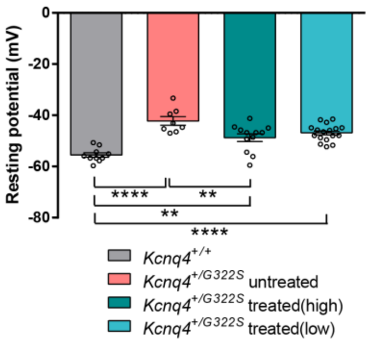

Figure 6. (E) Quantification of rest membrane potential in *Kcnq4*$^{+/+}$ controls (n = 10 cells from 5 mice), treated (high dose n = 12 cells from 6 mice, low dose n=17 cells from 10 mice) and untreated (n = 8 cells from 4 mice) *Kcnq4*$^{+/G322S}$ mice. Data are presented as mean ± SEM. Statistical analysis was performed using one-way ANOVA with Bonferroni's post hoc test. *$p < 0.05$; **$p < 0.01$; ***$p < 0.001$; ****$p < 0.0001$; ns: not significant.

5. In Figure 5D, how was the SGN survival quantified? The areas of Rosenthal's canal shown in the confocal images are consistently smaller in the untreated mice than the other two groups. I would suggest the authors count SGNs in a specified area which will make the counts more consistent and non-biased.

Response: We appreciate the reviewer's insightful feedback regarding our SGN quantification. We acknowledge that the control of the cross-sectional area of Rosenthal's canal was insufficiently rigorous in our initial analysis, and we sincerely apologize for this oversight. To address this,we have now selected consistent anatomical regions across all experimental groups and transitioned from reporting raw SGN counts to SGN density (SGNs/mm^2) (revised Figure 5D, E). This methodological refinement ensures a more standardized and rigorous evaluation, effectively minimizing potential biases arising from variations in sectional area. The detailed conclusions based on this revised analysis are elaborated in our previous response, and the revised manuscript has been updated accordingly (line 313-320).

6. In Figure 5F, the authors showed that the treated mutant mice had increased afferent/efferent nerve fibers compared to non-treated mutants. Yet, the images seem to suggest that at the apex and middle turns of the cochlea, the non-treated mutant seem to have greater number of the afferent/efferent nerve fibers compared to the treated mutants.

Response: While the NF200 signal in untreated mutant mice may appear superficially more prominent or 'dense' in certain regions, we interpret this as a pathological manifestation rather than a sign of superior preservation. Our reasoning is as follows:

Pathological Progression of Degeneration: Neurofilament heavy chain (NF200) primarily labels Type I afferent fibers and a subset of efferent fibers, with Type I afferents

being the most critical component for auditory transmission (indicated by red dashed boxes). In the absence of hair cells, the loss of trophic support triggers a degenerative sequence in these fibers: transitioning from an orderly arrangement to chaotic sprouting, pathological swelling, and ultimately retraction or complete disappearance.

Disorganized Sprouting vs. Functional Innervation: The untreated group clearly exhibits this pathological state. The swollen fibers and disorganized sprouting (spatially diffuse signals) create a false impression of increased density. However, this is a dysfunctional state, as evidenced by the localized disappearance of fibers in regions where IHCs are lost (indicated by red arrows)

Structural and Functional Rescue: In contrast, the nerve fibers in treated mutant mice are highly organized and bundled, maintaining integrated innervation with the surviving hair cells, particularly the IHCs. This refined morphology represents a healthy innervation pattern (revised Figure 5F). The above findings are described in detail in the revised manuscript (lines 320–328).

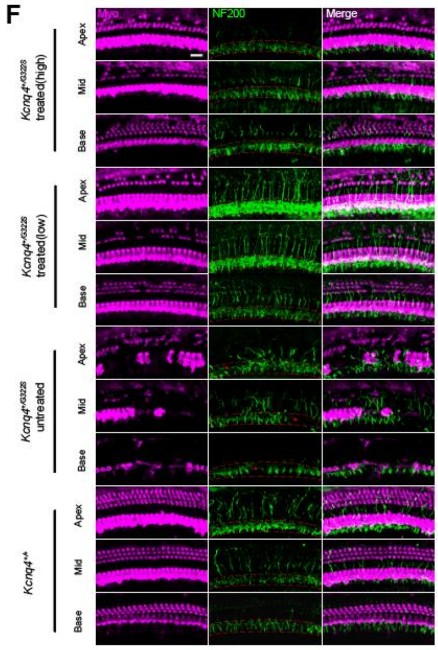

Figure 5. (F) Representative immunofluorescence images of afferent/efferent nerve fibers from *Kcnq4*$^{+/+}$, treated (3.64 × 10^{10} gc/mouse and 1.3 × 10^{10} gc/mouse), and untreated *Kcnq4*$^{+/G322S}$ mice . The red dashed box demarcates the region of afferent nerve fibers, while the red arrow indicates a focal loss of innervation. Scale bar, 20μm.

7. In figure 6, how did the authors know which OHCs in the treated mutant mice were transduced with AAVs and underwent successful base editing? The sample sizes provided in the figure legend, do they represent the number of mice used for each experiment or the number of OHCs recorded?

Response: We thank the reviewer for this critical question. Due to the limited packaging capacity of the AAV vector, we were unable to incorporate a fluorescent reporter into our

base-editing construct to directly trace transduced cells, so we were not able to pre-select a successfully edited OHC before recording. But our immunohistochemistry data using AAV-ie-EGFP demonstrated that this specific capsid achieves over 85% transduction efficiency in OHCs across all cochlear turns (revised Figure EV3A-C). Furthermore, our dual-AAV co-transduction assays confirmed a high probability of both vectors entering the same cell (revised Appendix Figure S3). This suggests that the vast majority of patched OHCs likely received the therapeutic components.

However, We acknowledge that beyond transduction, successful editing is further contingent on recombination efficiency and the inherent editing potency of the base editor. Consequently, the treated cochlea inevitably exists in a “mosaic” state, where a subset of OHCs is fully rescued while others remain at various stages of repair or unedited. By performing unbiased random sampling rather than targeting only 'ideal' cells, our data more accurately reflects the overall therapeutic landscape and correlates more closely with the hearing recovery observed in ABR thresholds. To minimize the impact of individual variability on the population-level analysis, we maximized the sample size to the fullest extent permitted by experimental feasibility,thereby enhancing the stability of these findings.

Here, “n” denotes the number of recorded OHCs. For enhanced data transparency, we have specified the number of mice from which these cells were derived alongside each “n” value, as reflected in the revised legend for Figure 6.

<u>Referee #2 (Comments on Novelty/Model System for Author):</u>

appropriate mouse model, straight forward for first line of preclinical proof of concept

<u>Referee #2 (Remarks for Author):</u>

EMBO Molecular Medicine - Referee Report
Title: Base editing rescue of dominant Kcnq4 p.G322S hearing loss in mice (Kong et al.)
Summary & significance
Kong et al. report dual-AAV delivery of split ABE8e to correct a dominant Kcnq4 p.G322S mutation that models human DFNA2 (KCNQ4 p.G321S). In the organ of Corti they achieve approximately 22-30% genomic correction (peaks around 40%) and approximately 25-44% cDNA correction within three weeks, alongside sustained functional rescue: best ABR threshold gains reach about 49 dB at 32 kHz, with low-dose effects persisting to 32 weeks. Morphology and physiology corroborate rescue (hair cells, spiral ganglion neurons, afferent/efferent fibers; partial KCNQ4 current restoration in outer hair cells). The work substantially advances mutation-correction strategies for dominant hearing loss and is likely of high interest to the field.
Major strengths
• Robust in vivo editing for the inner ear with clear benchmarking against prior rates, and careful quantification at DNA and cDNA levels.
• functional data (4-32 kHz ABR/DPOAE)

• Dose-response insight (early high-dose benefit but faster decline; superior durability at low dose).
• Anatomical/physiological correlates (hair cells/spiral ganglion neuron survival; nerve fiber density; outer hair cell KCNQ4 current).
• Initial safety/off-target assessment, including predicted DNA off-targets (HTS, GUIDE-seq) and transcriptome-wide RNA A-to-I analysis.

Response： We thank the reviewer’ comments.

Overall recommendation
Major revision. The study is exciting and timely, with convincing efficacy. Addressing the points above-especially a deeper, data-driven analysis of why rescue is limited (co-transduction/reconstitution, editing mosaicism, disease kinetics), strengthening off-target/biodistribution safety, and reporting/availability-will substantially enhance the manuscript's rigor and translational value.

Major points
1) Scope and mechanism of the incomplete/limited rescue
The functional gains are impressive but not complete or uniform over time and frequency. The Discussion touches on delay vs. durability and hair cell degeneration, but the manuscript would benefit from a deeper, quantitative analysis of potential limiting steps:
• Vector biology & delivery
- Despite excellent reporter transduction (inner hair cell > 98%; outer hair cell 86-99% by turn), editing efficiencies at the target locus are lower; please discuss bottlenecks beyond cell entry (e.g., AAV uncoating, nuclear entry, expression timing).

Resonse: We thank the reviewer for this constructive suggestion. While high reporter transduction reflects efficient cellular entry and detectable transgene expression, successful genome editing requires additional post-entry steps, which can be rate-limiting in post-mitotic cochlear hair cells. These steps include endosomal escape and trafficking, nuclear import and AAV uncoating, (for ssAAV) second-strand synthesis, and sufficient, sustained nuclear expression of the base editor and gRNA (Nonnenmacher & Weber, 2012). Consequently, impairments in AAV uncoating, hindered nuclear entry of the viral genome, suboptimal protein expression, or inefficient nuclear localization can all reduce the ultimate editing efficiency. Additionally, target-locus accessibility and the cellular DNA repair context remain critical determinants. Collectively, these stages establish an “efficacy threshold” for the editor that is substantially higher than that for a fluorescent reporter. Since fluorescent proteins can be detected even at minimal expression levels, reporter transduction primarily serves as a binary indicator of AAV entry, whereas editing efficiency constitutes a high-threshold quantitative evaluation. Furthermore, because base editors are typically delivered via dual-AAV vectors, the efficiencies of co-transduction and recombination are pivotal factors (as discussed in subsequent responses). In summary, AAV entry represents only the initial prerequisite; achieving effective genome editing

requires overcoming multiple downstream biological barriers. We have added these contents in the revised manuscript (lines 472–479).

-Clarify co-transduction and intein reconstitution rates in vivo for N + C halves (split ABE). In vitro, splitting reduced activity by about 21%. Can you estimate cochlear N ∩ C co-delivery/recombination from dual-fluor or barcoded reporters, or reference literature rates, to help explain the gap between transduction and editing? Also please compare to other dual-AAV approaches

Response: We appreciate the reviewer's inquiry regarding the disparity between transduction and editing efficiencies. While our dual-fluorescent AAV assays demonstrated high co-transduction rates—exceeding 90% in OHCs across all cochlear turns and approaching approximately 100% in IHCs—we have additionally included dual-AAV co-transduction experiments, which confirmed a high probability of both vectors entering the same cell (revised Appendix Figure S3). Nevertheless, we fully acknowledge that it is essential to distinguish cellular entry from functional genomic modification. The final editing efficiency is governed by a series of downstream bottlenecks, with the reconstitution of the split N- and C-terminal fragments being one of the most pivotal factors.

Our study utilizes an intein-mediated reconstitution strategy, which assembles two protein fragments into a complete protein through trans-splicing. This approach is widely adopted due to its characteristic high efficiency and rapid kinetics (Zettler *et al*, 2009). The reconstitution efficiency of this system depends on several factors, including the specific type of intein, the protein split site, the quality of vector preparation, and the expression balance between the two terminals. For instance, Ferreira et al. (Ferreira *et al*, 2023) demonstrated that the reconstitution efficiency of Cfa and Gp41-1 can be twice that of Npu and DnaE, and that high-quality virus preparation can significantly enhance protein reconstitution. Furthermore, Tang et al. (Tang *et al*, 2023) found that the intein-Rma combined with the S818/S1114 split site yielded the highest efficiency when splitting the otoferlin protein, further highlighting the importance of intein selection and split-site design. Tornabene et al. (Tornabene *et al*, 2019) also revealed the critical impact of N/C-terminal stability on reconstitution efficiency.

Given that our study employs the Npu intein, Brovin et al. (Brovin *et al*, 2024) explicitly noted that Npu-intein mediated GFP assembly yields were reduced by approximately 25% compared to direct expression. Our data showed that the editing efficiency of split-ABE8e decreased by about 21% compared to single-plasmid transfection, suggesting an Npu-intein reconstitution efficiency of approximately 75%–80%. Additionally, our "cell screening" results showed that even with single-plasmid transfection of ABE8e, the editing efficiency in successfully transfected cells was less than 50%. This indicates that, beyond cellular transduction, factors such as the intrinsic potency of the editor and chromatin accessibility at the target locus may also constrain the overall editing efficiency. Therefore, transduction efficiency cannot be equated with editing efficiency. Rigorously speaking, high transduction efficiency is a prerequisite for high

editing efficiency, but it is not the sole condition.

In addition to intein-mediated strategies, dual-AAV reconstitution predominantly employs overlapping (homology-based) reconstitution at the DNA level, dual-AAV trans-splicing at the RNA level, or hybrid designs combining both. The overlapping strategy uses a homologous overlap sequence to promote reconstitution via homology-dependent recombination/annealing (e.g., SSA/HR-like pathways), while NHEJ may also contribute and can be error-prone. Dual-AAV trans-splicing places a splicing donor (SD) on the 5′ fragment and a splicing acceptor (SA) on the 3′ fragment, enabling formation of a full-length mRNA (and thus full-length ORF/protein) after concatemerization and spliceosome-mediated processing. Because cochlear hair cells are post-mitotic and canonical HDR/HR is cell-cycle constrained, purely overlap-based reconstitution can be variable; therefore TS/hybrid (and more recently intein-based protein reconstitution) are commonly explored in this context.

Theoretically, a hybrid strategy combining overlap and splicing can enhance reconstitution efficiency. However, it increases vector design complexity (often necessitating extensive engineering and optimization) and, independently, consumes additional AAV genome space, thereby reducing the effective payload available for the therapeutic sequence. Its performance may not always surpass pure TS. For example, Al-Moyed et al. reported a higher fraction of correctly reconstituted otoferlin-positive IHCs with TS than with the hybrid design (~30% vs. ~19%, based on N- and C-terminal double labeling) (Al-Moyed *et al*, 2019). In contrast, intein-mediated protein reconstitution avoids DNA-junction indels associated with end-joining and bypasses aberrant RNA splicing products; it can be efficient once both fragments are co-expressed and co-localized. A head-to-head comparison by Xun et al. (Xun *et al*, 2026) showed that the intein strategy achieved higher efficiency, longer-lasting expression, and superior functional rescue than RNA-based approaches in the DFNB9 context, highlighting its broad potential in the dual-AAV delivery landscape. We have added these contents in the revised manuscript (lines 479–488).

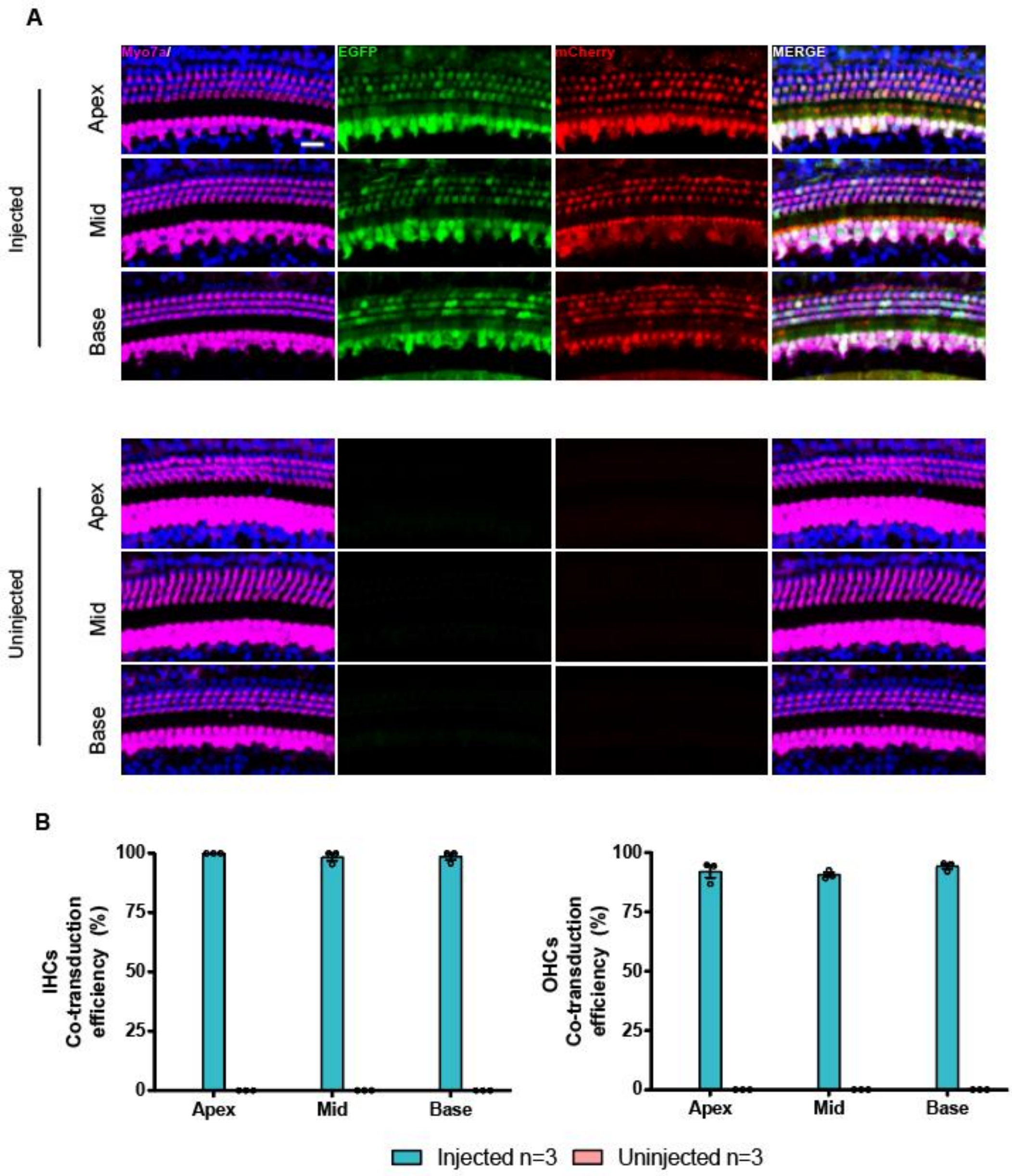

Appendix figure S3. (A) Immunofluorescence images *Kcnq4*$^{+/+}$ mice injected with AAV-ie-EGFP and AAV-ie-mcherry and uninjected controls. (B) Quantification of co-transduction efficiency of IHCs and OHCs in the injected (n=3) and uninjected (n=3) *Kcnq4*$^{+/+}$ mice. Data are presented as mean ± SEM.

• Editing pharmacodynamics

- ideally provide a timeline model for onset of editor expression vs. progression of hair cell loss, especially in basal vs apical turns. This could explain the delayed yet durable low-dose rescue and why early cell loss may be irreversible before editing acts.

Response: To characterize the temporal kinetics of ABE8e expression and the resulting

base-editing efficiency, we performed RT–qPCR on RNA harvested from the organ of corti in wild-type mice at 3, 7, 14, and 21 days post-administration of high-dose AAV. Our results demonstrate that ABE8e transcript levels increased progressively over this period, with a marked acceleration occurring at day 14. Consistently, base-editing outcomes assessed in homozygous mutant mice at parallel time points revealed a time-dependent increase in editing efficiency, reaching 33% at day 21—a nearly threefold elevation compared to the 10.5% observed at day 3 (revised Appendix figure S2C, D). These findings align with the established observation that AAV-mediated transgene expression typically ramps up during the initial weeks post-delivery, reaching a peak or plateau at approximately 2-4 weeks (Hollidge et al, 2022; Lange et al, 2016; Reimsnider et al, 2007). Notably, editing efficiency inherently exhibits a cumulative profile over time; this phenomenon is particularly pronounced in post-mitotic cells, such as cochlear hair cells, where the lack of cell division prevents the dilution of viral genomes and allows for a sustained, gradual increase in editing events over weeks to months（will be discussed in subsequent response).

To define the temporal window of hair-cell degeneration, we performed morphological analyses at P8 and P14. At P8, when KCNQ4 expression is first initiated (Kharkovets et al, 2000), both homozygous and heterozygous mutant mice maintained a full complement of hair cells. However, profound OHC degeneration was triggered shortly thereafter. By P14, homozygous mutants showed substantial OHC depletion, with losses reaching 53% (apex) and 42% (mid). Heterozygous mice followed a similar but attenuated trajectory, showing 23% and 12% reductions in the apical and middle turns, respectively (revised Appendix figure S2A, B). Our findings highlight a critical temporal disparity between the rapid onset of pathology and the progressive accumulation of therapeutic effect. While ABE8e-mediated editing efficiency continued to rise at 14 days post-injection (i.e.,P17) without reaching a plateau, substantial OHC loss was already evident by P14. This therapeutic lag—where pathological progression outpaces the onset of gene-editing efficacy—likely accounts for the limited overall treatment benefit observed in the more severely affected cochlear regions. Notably, OHCs in the basal turn exhibited superior preservation in mutant mice; in heterozygotes, the OHC count at 4 weeks remained nearly indistinguishable from wild-type controls (revised Figure 1K,M). This relatively intact cellular environment provides a more favorable physiological substrate and an extended therapeutic window for intervention. Furthermore, both AAV transduction and editing efficiencies reached their peak in the basal turn (revised Figures EV3A,B and Appendix figure S5A), likely underpinning the sustained preservation of high-frequency hearing observed over the extended follow-up period (revised Figure 4). Collectively, these findings underscore that a fundamental prerequisite for achieving robust therapeutic efficacy is the requirement for the therapeutic payload to reach its functional threshold before the onset of irreversible structural degeneration in inner-ear tissues. Precise identification and exploitation of this “critical therapeutic window” remain essential for attaining more potent and durable treatment outcomes. We have added these contents in the revised manuscript (lines 132–134;488-497).

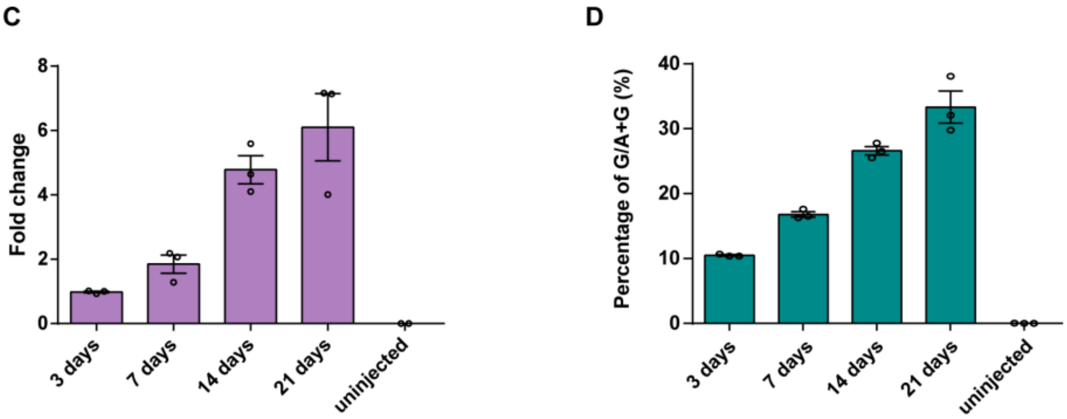

Appendix figure S2. (C) Time course of ABE8e expression in *Kcnq4*$^{+/+}$ mice at different time points following AAV injection (n=3 per group, 3.64 × 10^10 gc/mouse) and uninjected controls (n=2). (D) The editing efficiency of *Kcnq4*$^{G322S/G322S}$ mice at time points corresponding to (C) after AAV injection (n=3 per group, 3.64 × 10^10 gc/mouse) and uninjected controls (n=3). Data are presented as mean ± SEM.

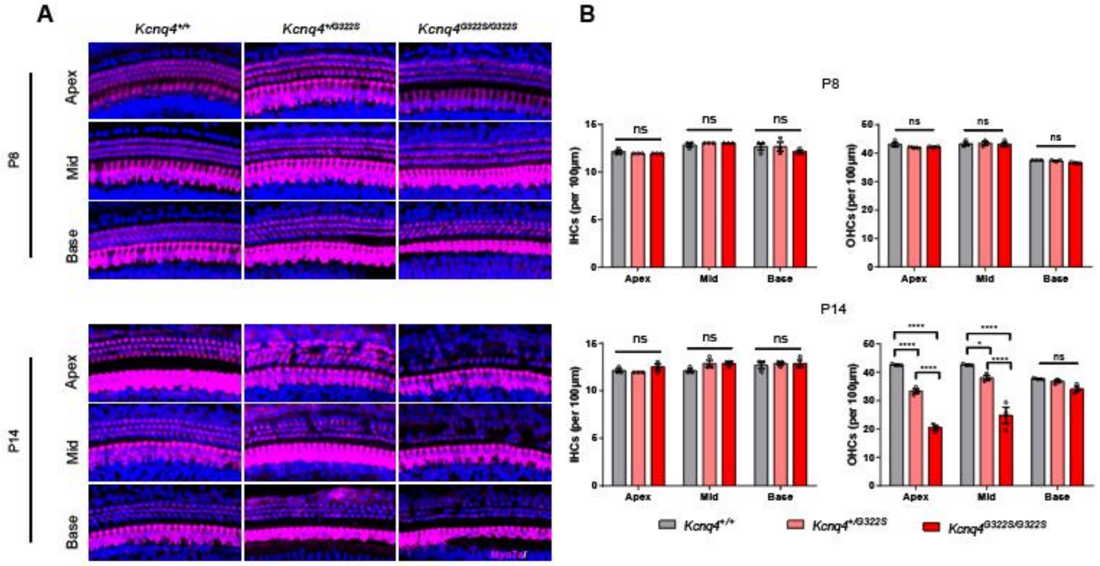

Appendix figure S2. (A) Immunofluoresence images of the organ of corti in *Kcnq4*$^{+/+}$, *Kcnq4*$^{+/G322S}$ and *Kcnq4*$^{G322S/G322S}$ mice at P8 and P14. (B) quantification of IHCs and OHCs in *Kcnq4*$^{+/+}$,*Kcnq4*$^{+/G322S}$ and *Kcnq4*$^{G322S/G322S}$ mice at P8 and P14 (n=3 per group at each time point). Data are presented as mean ± SEM. Statistical analysis was performed using two-way ANOVA with Bonferroni's post hoc test. *$p < 0.05$; **$p < 0.01$; ***$p < 0.001$; ****$p < 0.0001$; ns: not significant.

- If available, include longitudinal editing levels beyond 3 weeks to relate molecular correction to the ABR/DPOAE trajectories (weeks 4-32).

Response: To assess the long-term persistence and spatial distribution of genomic editing, we harvested the organ of Corti—including both turn-specific segments and whole-mount preparations—from 24-week-old heterozygous mice. Genomic DNA analysis revealed a distinct dose-dependent response and a tonotopic gradient in on-target editing efficiency. In both the high-dose and low-dose groups, the basal turn consistently exhibited the highest editing levels (27% vs. 18%), followed by the middle (20% vs. 14%) and apical turns (19% vs. 11%) (revised appendix Figure S5A). This spatial hierarchy aligns with the transduction profile of AAV-ie-EGFP, which also demonstrated peak efficiency in the basal region (revised Figure EV 3A,3B).

Furthermore, editing efficiency exhibited a cumulative temporal profile; at 24 weeks, the efficiencies measured in the whole organ of Corti were significantly higher than those observed at 3 weeks for both treatment regimens (high dose: 22% vs. 17%; low dose: 17% vs. 8%) (revised Appendix Figure S5A). Notably, the high-dose regimen produced measurable audiological improvements as early as the initial 4-week assessment, when the editing efficiency in heterozygous mice was approximately 17%. In contrast, the 24-week time point represented the “optimal efficacy window” for the low-dose group, at which editing efficiency reached approximately 17% in heterozygous mice and was associated with minimal hearing deterioration between 12 and 28 weeks. Collectively, these findings indicate that in heterozygous mice, an editing efficiency of ~8% at 3 weeks post-injection is insufficient to achieve a significant auditory benefit, whereas efficiencies approaching ~17% appear necessary to confer robust hearing improvement. Importantly, provided that cochlear immune activation is not exacerbated, higher levels of gene correction would be expected to translate into proportionally greater hearing preservation or recovery. We have added these results in the revised manuscript (lines 204–213;303-305).

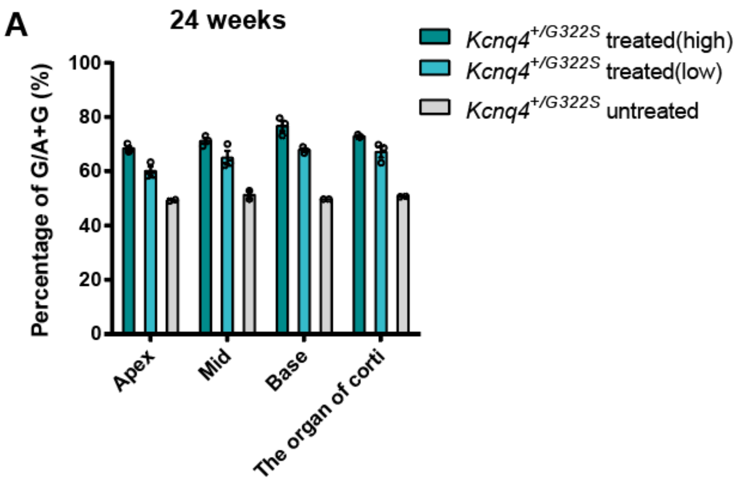

Appendix figure S5. (A) The percentage of G/A+G across apical, middle and basal turns, as well as in the whole organ of corti at DNA level in high-dose treated, low-dose treated (n=3 per group) and untreated *Kncq4*$^{+/G322S}$ mice at 24 weeks of age (n=2).

• Allelic context & mosaicism

- In heterozygotes the DNA/cDNA correction is modest (e.g., roughly 8-17% DNA, 2-8% cDNA above baseline). Please discuss how much cell-level correction is required to overcome dominant-negative effects and whether mosaic correction in critical nodes (e.g., basal outer hair cells) suffices for large ABR effects. Moreover, how could rescuing some hair cell might or might not slow the degenerative process

Response: We appreciate the reviewer's insightful question. When quantifying editing outcomes in heterozygous mice, we reported the absolute genomic editing efficiency, defined as the fraction of alleles harboring the A-to-G conversion among the total population of alleles at the target locus. In an idealized heterozygous context, the theoretical upper limit of this metric is 50%, as only the mutant allele is the target for correction. Consequently, the apparent editing efficiency in heterozygotes is systematically lower than that observed in homozygous mice.

For disease driven by dominant-negative variants, the requisite proportion of corrected cells—the 'therapeutic threshold'—is highly dependent on the underlying pathogenic mechanism. In our study, although OHC survival in the apical and middle turns reached approximately 50%, and basal turns showed near-complete preservation in 8 weeks (revised Figure 5A-C), we observed a progressive decline in auditory function (revised Figure 4) and ongoing hair-cell loss (revised Appendix figure S2 and S4). These findings suggest that in a 'mosaic-correction' setting, achieving robust and durable therapeutic benefits likely requires the correction of a substantial majority of the OHC population. Furthermore, since auditory function is integrated across the tonotopic axis of the cochlea, functional recovery in a specific region is primarily reflected in its corresponding frequency range (e.g., the basal turn for high frequencies). Consequently, achieving pan-cochlear rescue of hair cells remains the definitive objective for optimizing functional outcomes in gene therapy.

Regarding the ongoing degeneration of "rescued" hair cells and the continued decline in auditory function, we propose two plausible contributing mechanisms. First, alterations in the immune microenvironment: the progressive loss of unedited OHCs may sustain chronic inflammatory signaling and promote macrophage recruitment and activation. Such changes could create a hostile local milieu that secondarily compromises the long-term viability of neighboring, genetically corrected OHCs (Kaur *et al*, 2015; Pan *et al*, 2024). Second, the structural collapse of the organ of Corti: OHC loss is known to trigger extensive supporting-cell remodeling, including the expansion, displacement, and deformation of Deiters' cells and outer pillar cells. This process disrupts the cytoarchitecture of the sensory epithelium, ultimately leading to epithelial flattening or collapse (Taylor et al, 2012). Support for these mechanisms is also provided by the longitudinal auditory and hair-cell survival data. In the low-dose treatment group, hearing thresholds remained largely stable between 12 and 28 weeks of age, followed by a rapid deterioration between 28 and 36 weeks (revised Figure 4C, D, J; Figure EV5A–E). Notably, in a complementary analysis, we performed cochlear whole-mount preparations from low-dose–treated heterozygous mice at 32 weeks of age. At this time point, OHCs were almost completely absent across the cochlea, whereas iIHCs were still largely preserved in the basal turn. These observations suggest that the abrupt hearing decline

between 28 and 32 weeks is primarily attributable to the progressive and near-complete loss of OHCs. Subsequently, between 32 and 36 weeks, the complete absence of OHCs appears to precipitate further degeneration and eventual loss of IHCs (revised Appendix Figure S4). Taken together, these findings indicate that more effective and earlier correction of pathogenic cells—achieving preservation of a larger fraction of hair cells at early stages, while avoiding excessive immune activation—is likely to yield more robust and durable therapeutic outcomes. We have added these contents in the revised manuscript (lines 495–504).

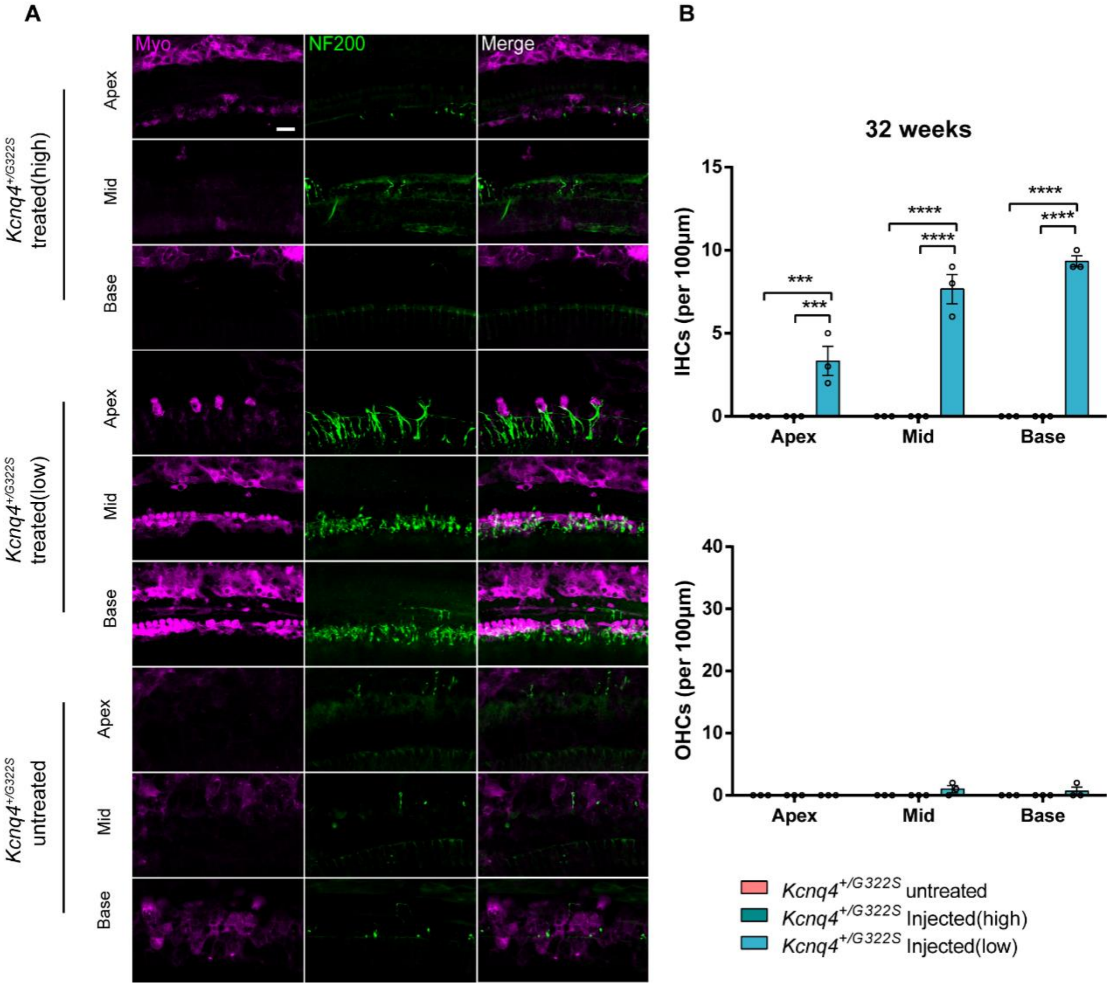

Appendix figure S4. (A) Immunofluoresence images of the organ of corti in high-, low-dose treated *Kcnq4*$^{+/G322S}$ mice and untreated controls at 32 weeks of age(the magenta represents HCs and the green represents efferent and afferent neural fibers). Scale bar, 20 μ m. (B) Quantification of HCs in two regimens treated *Kcnq4*$^{+/G322S}$ mice and untreated controls at 32 weeks of age(n=3 per group). Data are presented as mean ± SEM. Statistical analysis was performed using two-way ANOVA with Bonferroni's post hoc test. *p < 0.05; **p < 0.01; ***p < 0.001; ****p < 0.0001; ns: not significant.

• OHC functional rescue: Kharkovets et al., 2006 report on a depolarized resting potential of outer hair cells and argue that function of OHCs is hampered because of this before OHC loss due degeneration because of chronic depolarization. I think the manuscript

would benefit from reporting some current-clamp measurements of OHC resting potential in WT, Mut and Mut+rescue

Response: We thank the reviewer for the insightful suggestion. We recorded the resting membrane potentials (RMPs) of cochlear outer hair cells (OHCs) from heterozygous mice treated with different regimens, alongside wild-type controls, at P9–P14. Mutant OHCs exhibited significant depolarization (~25% increase) relative to wild-type controls, confirming ion-channel dysfunction (revised Figure 6E). Importantly, based on our supplementary analyses, the vast majority of OHCs in heterozygous mice remained intact between P8 and P14 (revised Appendix Figure S2A, B), consistent with the reviewer's point and with Kharkovets et al. (2006), who reported that disruption of RMP precedes hair-cell loss. While high-dose treatment effectively rescued this phenotype, producing a 17% hyperpolarizing shift, the low-dose group showed only a non-significant downward trend. This difference indicates that, at this stage, the high-dose regimen—but not yet the low-dose—achieves the therapeutic threshold required for effective RMP restoration. We have added these results in the revised manuscript (lines 342–347).

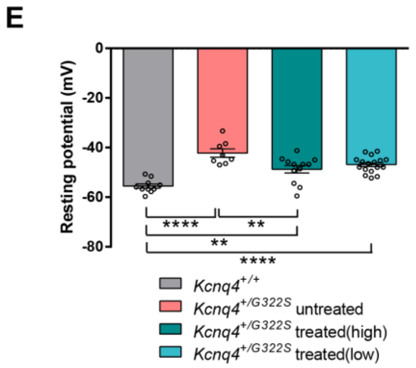

Figure 6. (E) Quantification of rest membrane potential in *Kcnq4*$^{+/+}$ controls (n = 10 cells from 5 mice), treated (high dose n = 12 cells from 6 mice, low dose n=17 cells from 10 mice) and untreated (n = 8 cells from 4 mice) *Kcnq4*$^{+/G322S}$ mice. Data are presented as mean ± SEM. Statistical analysis was performed using one-way ANOVA with Bonferroni's post hoc test. *$p < 0.05$; **$p < 0.01$; ***$p < 0.001$; ****$p < 0.0001$; ns: not significant.

• Tonotopic analysis

- Because DFNA2 begins basally, a brief turn-resolved correlation (editing vs. transduction vs. hair cell survival vs. thresholds) would strengthen the mechanistic link between tonotopic vulnerability and rescue magnitude.

Response: We thank the reviewer for this suggestion. As noted in our previous response, our turn-specific assessment of editing efficiency in 24-week-old heterozygous mice revealed that the basal turn consistently exhibited the highest editing levels (27% vs. 18% in high- and low-dose groups, respectively), followed by the middle (20% vs. 14%) and

apical turns (19% vs. 11%). This spatial hierarchy aligns with the transduction profile of AAV-ie-EGFP, which also demonstrated peak efficiency in the basal region (revised Appendix figure S5A). Furthermore, the superior editing efficiency in the basal turn likely facilitated enhanced OHC survival (revised Figure 5A,5B), ultimately resulting in lower high-frequency hearing thresholds (revised Figure 4A-H). These findings reinforce the mechanistic continuum linking transduction efficiency, editing levels, morphological preservation, and auditory recovery. We have added these results in the revised manuscript (lines 205-–213;266-268;303-308).

2) Dose-dependent decline and neuro-immune responses
The high-dose cohort shows earlier improvement but accelerated decline from around 16 weeks, whereas low-dose sustains gains. The cytokine/microglia/macrophage data suggest greater local immune activation at higher dose. Please:
• Expand on causality (editing toxicity vs. AAV load vs. editor overexpression). Could hit-and-run expression (self-limiting vectors, protein/RNP delivery)

Response: We appreciate the reviewer's insightful query regarding the potential mechanisms underlying the accelerated late-stage deterioration observed in the high-dose treatment group. We hypothesize that this phenomenon is driven by the interplay of three primary factors:

1. **Editing Toxicity: RNA Off-Target Effects**
Beyond catalyzing A-to-G conversions on genomic DNA, the TadA deaminase module within ABEs can engage RNA, inducing widespread, transcriptome-wide off-target RNA A-to-I editing (Richter *et al*, 2020; Zhou *et al*, 2019). Evidence suggests that at high exposure levels, frequent RNA off-target events can compromise cellular viability and pose in vivo risks (Zhang *et al*, 2026). Our transcriptomic analysis aligns with this, revealing a higher frequency of RNA A-to-I edits in the high-dose cohort (revised Figure EV4I; appendix Figure S5D-F). Over time, this cumulative RNA off-target activity likely imposes chronic stress on hair cell survival. Consequently, engineering ABE variants with reduced RNA off-target propensity—while maintaining a balanced dose-response—remains a critical strategy for mitigating this limitation (Li *et al*, 2021; Zhang *et al.*, 2026).
2. **Multi-Component Immunogenicity: Capsid, Genome, and Editor**
AAV-mediated delivery introduces several immunogenic cues:

Innate Sensing of Capsids: AAV capsids are recognized by innate immune receptors (e.g., TLR2), triggering downstream inflammatory signaling and cytokine production. Given the presence of resident macrophages in the cochlea, such activation can foster a pro-inflammatory microenvironment (Chan *et al*, 2022; Hosel *et al*, 2012; Ishibashi *et al*, 2023).
Genome-Mediated Response: Unmethylated CpG motifs in the AAV genome can be sensed by endosomal TLR9 (signaling via MyD88) to induce Type I interferons and inflammatory cytokines. Furthermore, cytosolic DNA-sensing pathways (e.g., cGAS–

STING–TBK1, with parallel NF-κB activation) may amplify inflammation in response to ITR-containing genomes or dsDNA forms (Chandler *et al*, 2019; Muhuri *et al*, 2021).

Adaptive Immune Risks: Since AAV-delivered editors are expressed long-term, degradation-derived peptides can be presented on MHC-I and recognized by CD8+ T cells (Najera *et al*, 2025). While macrophages predominate in the cochlea, resident populations of T, B, and NK cells exist (Liu & Xu, 2023), and injury may further recruit adaptive immune cells to the organ of Corti (Rai *et al*, 2020). Sustained exposure to this "inflammatory cytokine milieu" likely impairs hair cell metabolism and viability over time (Su *et al*, 2025; Sun *et al*, 2025).

Notably, recent evidence showing that immunomodulation (e.g., dexamethasone) can reduce macrophage recruitment underscores the pivotal role of immune management in inner-ear therapy (Wang *et al*, 2025a).

3. Perturbation of Cellular Proteostasis

High capsid loads may saturate the ubiquitin-proteasome system (UPS), creating a substantial proteostatic burden that potentially interferes with endogenous protein turnover (Kandel *et al*, 2024; Li *et al*, 2013). Simultaneously, high-level and sustained expression of exogenous proteins can elicit cellular stress responses; in some models, stress markers consistent with proteostasis disruption (including UPR/ER-stress–like signatures in certain contexts) have been observed alongside tissue injury. If these stressors chronically exceed a physiological threshold, adaptive responses may shift toward pro-apoptotic programs, compromising the long-term durability of the therapeutic effect (Kim *et al*, 2006; Lange *et al.*, 2016; Yasuno *et al*, 2024).

In summary, we speculate that high-dose administration provides substantial early benefit because the editing-driven repair initially outweighs these adverse influences. However, as cumulative toxicity and inflammation reach a "tipping point," hair cell loss accelerates.

With respect to potential solutions, two general directions may be considered.

Editor Optimization: Employing compact Cas9 orthologs (e.g., SaCas9, CjCas9 and SauriCas9) or increasing editing potency to enable dose reduction (Davis *et al*, 2022). However, this does not fully eliminate the risks of sustained editor expression, including off-target effects and chronic cellular stress.

Non-Viral Modalities: Transient delivery via mRNA-LNP, RNP, or VLPs offers "hit-and-run" kinetics, minimizing long-term off-target risks and immunogenicity (Hollidge *et al.*, 2022; Pardi *et al*, 2015). For instance, in our recent work on hereditary angioedema, LNP-mediated delivery outperformed AAV in editing efficiency and exhibited superior safety and off-target profile(Wang *et al*, 2025b), supporting the effectiveness and safety of non-viral delivery in certain disease contexts. While inner-ear delivery remains challenged by cell-type targeting and transduction efficiency (Liu *et al*, 2025), engineered VLP systems (Noh *et al*, 2025) have recently demonstrated enhanced in vivo editing performance compared to traditional AAV paradigms (AAV-Anc80L65) (Noh *et al*, 2022), offering a promising path forward for genetic hearing loss gene therapy. We have added these contents in the revised manuscript (lines 436–452).

• Include any hair-cell counts and synaptic metrics at late endpoints for high- vs low-dose

to link deterioration to pathology.

Respoonse： We appreciate the reviewer's suggestion and accordingly assessed late-stage cochlear morphology at 32 weeks post-treatment, with the relevant results presented in response to the preceding comment. At this time point, ABR measurements indicated profound deafness in both untreated and high-dose–treated heterozygous mice, whereas the low-dose group retained residual auditory responses at frequencies above 5.656 kHz (revised Figure 4D). Consistent with these functional outcomes, cochlear whole-mount immunofluorescence revealed a complete loss of both inner hair cells (IHCs) and outer hair cells (OHCs) in the untreated and high-dose cohorts. In contrast, although OHCs were nearly entirely depleted in the low-dose group, a subset of IHCs persisted, exhibiting a clear tonotopic gradient with higher survival in the basal turn and progressively reduced survival toward the apex. Notably, preservation of nerve fiber innervation at the IHC base was observed exclusively in the low-dose group, whereas both afferent and efferent fibers were fully degenerated in the other two cohorts (revised Appendix Figure S4). Collectively, these findings demonstrate that late-stage auditory deterioration closely parallels the progressive loss of hair cells and degeneration of cochlear nerve fibers, thereby reinforcing the internal consistency and reliability of our functional and morphological results. We have added these results in the revised manuscript (lines 259–263).

• Discuss aspects relevant for future clinical translation: dose selection and immune-modulatory strategies for the clinic.

Response: We appreciate the reviewer's insightful comments. Current clinical translation in the field of hereditary hearing loss has primarily focused on AAV-mediated gene-replacement therapy for *OTOF*-associated deafness. Across recent clinical reports, the per-ear dose for AAV-OTOF delivery typically spans from 5.6 x 10^11 to 1.5 x 10^12 vg, whereas Valayannopoulos et al. administered a substantially higher dose of 7.2 x 10^12 vg/ear (Lv *et al*, 2024; Qi *et al*, 2024; Qi *et al*, 2025; Valayannopoulos *et al*, 2025; Wang *et al*, 2024). While these studies generally reported no severe safety concerns, (the SAE described by Valayannopoulos et al. was deemed unrelated to the AAV delivery) evidence suggests that both systemic dissemination and immune activation follow dose-dependent kinetics. For instance, Wang et al. observed that bilateral administration correlated with elevated circulating AAV neutralizing antibody (NAb) titers (Wang *et al.*, 2024). Furthermore, Qi et al. identified a dose-dependent pattern of peripheral vector genome detection in cynomolgus macaques one day post-inner-ear injection (Qi *et al.*, 2024). Collectively, these observations establish a clear linkage between administered dose and the extent of systemic exposure.

Beyond dosage, immunogenicity is shaped by the AAV serotype and the specific transgene cargo. Base editors, in particular, introduce unique challenges, including potential editing toxicity and genomic/RNA off-target risks. Therefore, rigorous safety assessments in large animals or non-human primates (NHPs) remain indispensable for

establishing clinically informed dose-selection protocols.

AAV-associated immune risk remains a pivotal translational consideration. Lv et al. suggested that high baseline AAV NAb levels might be associated with suboptimal functional recovery (Lv *et al.*, 2024). Notably, Wang et al. demonstrated that excessive inflammatory responses following AAV delivery could threaten hair-cell survival; however, the administration of dexamethasone (DEX) showed promise in inhibiting macrophage recruitment, thereby enhancing both safety and therapeutic efficacy (Wang *et al.*, 2025a). The necessity of managing immune-mediated risks is further underscored by experiences in systemic gene therapy. For example, in a clinical trial of AAV9-SMN replacement for Spinal Muscular Atrophy (SMA) Type 1, a patient developed fatal thrombotic microangiopathy (TMA) driven by excessive complement activation. Despite the use of eculizumab (a C5 inhibitor) to suppress terminal complement signaling, the patient ultimately succumbed to infection and multi-organ failure (Guillou *et al*, 2022). These findings emphasize the critical need for systematic pre-treatment screening of baseline immune status, vigilant longitudinal monitoring, and the implementation of timely immunomodulatory interventions, including prophylactic immunosuppression where appropriate, to optimize the safety profile and durability of inner-ear gene therapies. We have added these contents in the revised manuscript (lines 523–539).

3) Off-target and biodistribution-request deeper analysis

The DNA off-target panel based on predicted sites was clean in cochlea and brain, but RNA-seq revealed dose-dependent A-to-I RNA editing. A few additions would substantially strengthen the safety case:

•Report genome-wide DNA off-targets beyond the predicted/GUIDE-seq panel if feasible (e.g., CHANGE-seq, DISCOVER-seq, CIRCLE-seq), or explicitly justify the current panel's coverage.

Response: We appreciate the reviewer’s insightful suggestion. In the original submission, we integrated in silico prediction (to nominate sgRNA-dependent off-target candidates) with GUIDE-seq (to capture genome-wide detection of Cas9-associated DSBs, including both sgRNA-dependent sites and non-canonical cleavage events), which together identified 12 potential off-target loci. Nevertheless, as the reviewer correctly noted, genome-wide off-target alterations may still be missed by these approaches. To address this concern, we additionally performed high-depth whole-genome sequencing (WGS; ~100× coverage) on cochlear tissues from mice following high-dose administration, enabling a more comprehensive and unbiased assessment of DNA-level off-target effects after treatment. WGS analyses revealed no significant increase in base substitution in injected mice compared with controls (revised Appendix Figure S6A). Moreover, the genomic and functional distributions of detected SNPs and indels—including coding versus non-coding regions, as well as exonic, intronic, and regulatory annotations—were likewise not significantly different between groups (revised Appendix Figure S6B). Collectively, these results provide additional evidence supporting a favorable genome-wide safety profile for this therapeutic strategy. We have added these results in

the revised manuscript (lines 233–237).

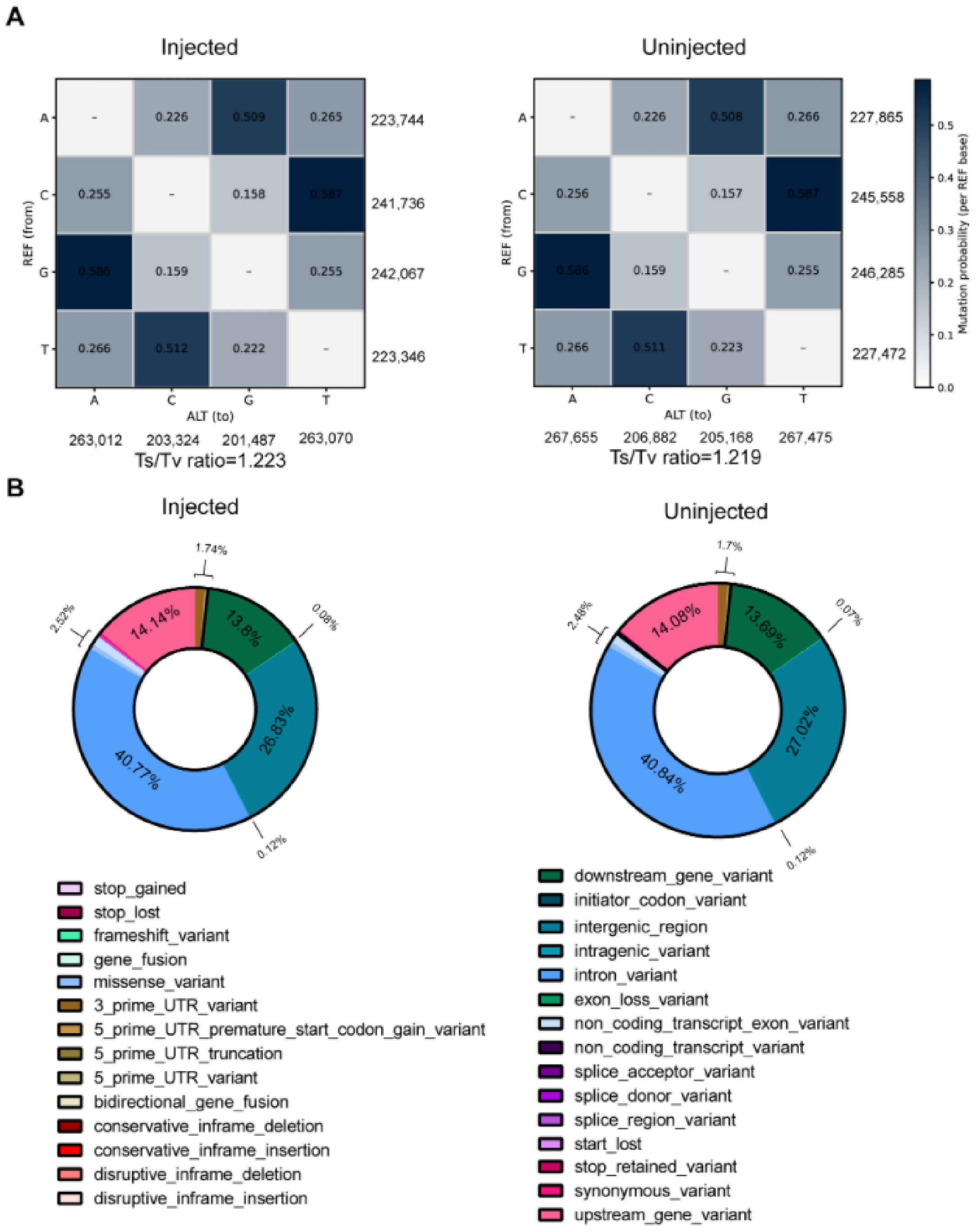

Appendix figure S6. (A) Base substitution matrix of SNPs between high-dose and uninjected *Kcnq4*$^{+/+}$ mice, Ts and Tv indicates transitions and transversions, respectively. (B) Distribution of SNPs and indels across functions and regions in high-dose, low-dose and uninjected *Kcnq4*$^{+/+}$ mice.

• Provide annotation of RNA edits (exonic vs. UTR/intronic; predicted consequence; overlap with ADAR hotspots) and an estimate of effect sizes at edited loci.

Response: We thank the reviewer for this helpful suggestion. To further interrogate the sequence-context preference of A-to-I editing events following ABE delivery, we overlapped the detected A-to-I variants with a curated set of endogenous deaminase (ADAR) hotspot loci (Picardi *et al*, 2017). As shown in Appendix Figure S5E, the high-dose cohort exhibited a markedly greater overlap with hotspot regions (202 and 218 sites, accounting for ~22–23% of all A-to-I calls) compared with the low-dose cohort (110 and 114 sites, ~12–13%) and uninjected controls (50 and 68 sites, ~10–13%). This dose-dependent enrichment at hotspot loci is consistent with increased cellular exposure to the TadA deaminase at higher vector doses, which may elevate deaminase-associated, guide-independent editing at intrinsically susceptible sequence contexts. Furthermore, Variant Effect Predictor (VEP) analysis revealed that these increased A-to-I variants were predominantly located within intronic regions (Appendix Figure S5F) and were largely classified as modifier variants with minimal predicted functional impact (Appendix Figure S5D). Collectively, these analyses indicate that the observed dose-dependent signal primarily reflects enrichment at endogenous ADAR hotspot loci with limited functional consequence, supporting an overall favorable transcriptome-wide safety profile at the early stage. Nevertheless, we acknowledge that high-dose administration may still trigger and progressively accumulate deleterious off-target editing events over an extended temporal horizon, which could ultimately compromise cochlear hair-cell integrity. As discussed in our earlier responses, this mechanism may partially account for the more rapid late-stage hearing deterioration observed in the high-dose group compared with the low-dose regimen. We have added these results in the revised manuscript (lines 239–243;).

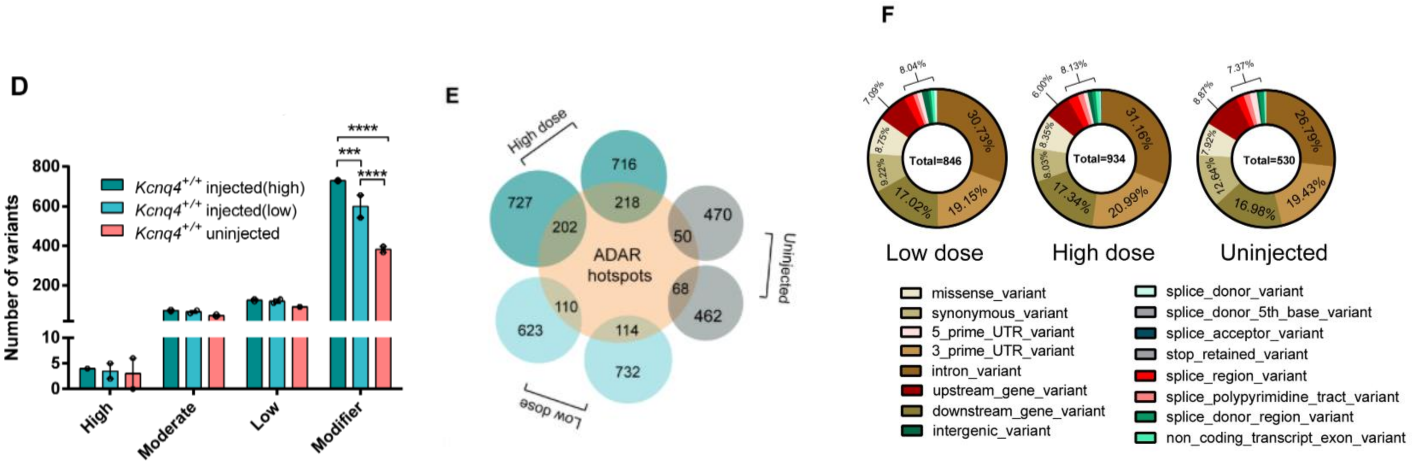

Appendix figure S5. (D) Analysis of A-to-I editing impact in high-dose, low-dose and uninjected *Kcnq4*$^{+/+}$ mice. (E) Overlap between A-to-I editing sites and ADAR hotspots across the high-dose, low-dose, and uninjected *Kcnq4*$^{+/+}$ mice. (G) Distribution of A-to-I editing sites across functions and regions in high-dose, low-dose and uninjected

Kcnq4$^{+/+}$ mice. Data are presented as mean ± SEM. Statistical analysis was performed using two-way ANOVA with Bonferroni's post hoc test. *p < 0.05; **p < 0.01; ***p < 0.001; ****p < 0.0001; ns: not significant.

• Because AAV-ie reporter showed expression in hippocampus and mild heart/liver, please clarify whether ABE components (not only EGFP) show extra-cochlear expression and whether off-target assays were performed in those tissues at the editor target sequence.
• State whether germline tissues were examined (or why not) given neonatal injections.

Response: We thank the reviewer for this insightful observation. We acknowledge that disparities in expression profiles may exist between AAV-ie-EGFP and AAV-ie-ABE8e due to variations in viral dosage, genomic cargo, and promoter elements. To address this, we quantified ABE8e transcript levels across various organs in homozygous mice three weeks post-injection using RT–qPCR. Consistent with our EGFP data, the brain, heart, and liver emerged as the top three off-target organs for ABE8e expression. Interestingly, upon evaluating on-target editing efficiencies, the liver exhibited a substantially higher editing rate (~15%) compared to the brain (~3%) and heart (~1%) (revised appendix figure S5B,C).

We propose that this phenomenon is driven by the physiological characteristics of neonatal mice. At the time of injection (P3), the blood–labyrinth barrier (BLB) is not yet fully mature, possessing high permeability that allows AAV particles to escape into the systemic circulation (Xu *et al*, 2024). As the organ receiving the largest total blood supply, the liver likely sequesters a significant portion of these leaked AAV particles. However, during subsequent development, rapid hepatocyte proliferation leads to the dilution of episomal AAV genomes, while immune clearance (e.g., via CD8+ T cells) (Kumar *et al*, 2024) further suppresses transcript levels. This is consistent with Ishibashi et al., who reported that AAV genomes detected in the liver three days post-inner-ear-injection became nearly undetectable by day 28 (Ishibashi *et al*, 2025). Crucially, since base editing is initiated as early as three days post-injection, the 'founder' hepatocytes are permanently modified. Even as the AAV vectors are diluted or cleared over time, the edited status is inherited by progeny cells, thereby consolidating the final editing outcome. As demonstrated by the study of Han et al. (Han *et al*, 2024), the systemic distribution profiles of different AAV serotypes vary significantly. To minimize editing activity in off-target organs, besides employing tissue-specific promoters, the screening of AAV serotypes is also a crucial step.

Furthermore, to assess the potential risk of germline transmission, we evaluated ABE8e expression and editing efficiency in gonadal tissues. Our results showed negligible ABE8e transcript levels and no detectable on-target editing in either the testes or ovaries (revised appendix figure S5B,C). These data underscore the relative biosafety of our localized delivery approach and its minimal risk of heritable genetic modification. We have added these results in the revised manuscript (lines 221–230).

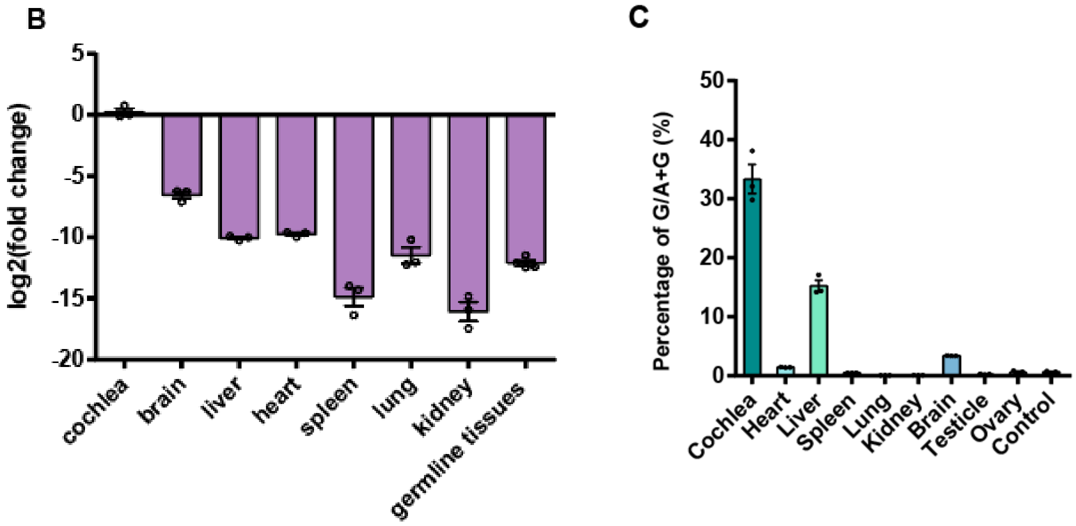

Appendix figure S5. (B) RT-qPCR quantification of ABE8e expression across major organs in high-dose treated *Kcnq4*$^{G322S/G322S}$ mice 3 weeks post injection, normalized to cochlear levels (germline tissues, *n* =4;other organs, *n* = 3). (C) A to G editing efficiency across major organs in high-dose treated *Kcnq4*$^{G322S/G322S}$ mice 3 weeks post injection (n=3 per group).

4) Statistics, design, and reporting

• You note blinding for ABR/DPOAE thresholding; please explicitly report randomization, litter/ear as unit of analysis, sex as a biological variable, and how you handled capping (ABR > 90 dB set to 100 dB) in statistical tests.

Response: Thank you for this suggestion. We have revised the Methods section to explicitly describe randomization, the experimental unit, and sex (Line 718-722 in revised manuscript). Briefly, animals were drawn from multiple litters, and pups within each litter were randomly assigned to experimental or control groups, with animals selected across different litters to avoid litter-specific bias. The right ear (injected ear) threshold of each mouse was measured for auditory analysis, and both male and female mice were included.

For ABR thresholds exceeding the maximum stimulus intensity, assigning a value of 90 dB SPL would render these observations indistinguishable from valid responses occurring exactly at that level. To facilitate visualization and statistical analysis, it is standard practice in the field to encode “no response” observations as the maximum tested level plus a fixed increment. Following established precedents—such as coding non-elicited responses as 95 dB (Hu et al, 2025), 100 dB (Jang et al, 2025), or 110 dB (Hong et al, 2026) —we assigned a value of 100 dB SPL to all thresholds exceeding our measurement limit. This convention was applied consistently throughout the manuscript to ensure statistical rigor and comparability across experimental cohorts.

• For longitudinal analyses, specify mixed-effects models or repeated-measures ANOVA choices and multiple-comparison control.

Response: To account for missing data arising from animal attrition during longitudinal monitoring, statistical analysis was performed using a mixed-effects model. This model is specifically designed to handle the unbalanced data structures inherent in repeated-measures designs. Post-hoc multiple comparisons were corrected using the Bonferroni method. Accordingly, the legend for revised Figure 4J has been revised to incorporate these statistical parameters.

5) Data availability

The current "available upon reasonable request" policy is below community expectations for a methods-heavy gene-editing study. Please deposit raw HTS/CRISPResso2 outputs, GUIDE-seq data, and RNA-seq FASTQs in public repositories with accession numbers included at acceptance (technical hold until accession numbers or active links are provided).

Response: We appreciate the reviewer’s suggestion.We have deposited the raw sequencing datasets into the NCBI Sequence Read Archive (SRA) under BioProject accession number: PRJNA1420045 (available at: https://dataview.ncbi.nlm.nih.gov/object/PRJNA1420045?reviewer=22tgv3pt90ur858kad82369co). The 'Data Availability' section of the revised manuscript has been updated accordingly to reflect this information.

Minor points and clarity edits

• Clarify whether bystander edits were fully excluded in vivo at the on-target window (you note none in vitro). Provide in vivo amplicon spectra.

Response: We thank reviewer’s suggestion. Accordingly, we have revised the corresponding subsections of the results as follows: “Notably, no bystander A to G editing was detected at either the genomic or the transcriptomic levels.” A schematic illustration of in vivo amplicon sequencing has been presented in revised Figure 3E.

• Include a simple schematic of editing window and PAM for the murine and human target to contextualize sgRNA choice and the two synonymous differences in the human guide.

Response: Done (revised Figure EV1A)

• Briefly compare editor classes (ABE8e vs newer compact/base-deaminase variants) for payload/precision trade-offs relevant to possible clinical translation.

Response: We selected ABE8e for its high efficiency in our proof-of-concept scenario.

We nonetheless agree with the reviewer that clinical translation is fundamentally constrained by in vivo delivery and safety concerns.

In this context, adopting compact or hypercompact Cas scaffolds can substantially reduce payload and render single-vector AAV delivery more feasible, thereby simplifying manufacturing and decreasing reliance on high doses and dual-vector co-transduction. Consistent with this rationale, compact ABE architectures built on smaller Cas9 orthologs (e.g., SaCas9, Nme2Cas9, CjCas9, and SauriCas9) have demonstrated robust in vivo editing in mice using single-AAV implementations explicitly designed around payload-driven constraints (Bamidele *et al*, 2024; Davis *et al.*, 2022; Kweon *et al*, 2023). Hypercompact systems are also emerging. For example, Cas12f-based ABEs have been positioned as AAV-friendly platforms to alleviate packaging bottlenecks (Kim *et al*, 2022). Importantly, "downsizing" typically introduces trade-offs, including altered PAM requirements and targeting range, as well as changes in editor geometry that can reshape editing windows and off-target landscapes. Thus, these backbones necessitate systematic, context-specific evaluation and optimization prior to their deployment in any therapeutic setting.

In parallel, the therapeutic application of base editors is often constrained by bystander editing within the activity window. To address this, substantial effort has been directed toward precision engineering of the TadA deaminase to narrow the effective window and mitigate bystander effects. Beyond the TadA-NW series (e.g., TadA-NW1/NW2), which compresses the editing window to ~4 nt while maintaining high on-target activity and reduced off-target profiles (Valdez *et al*, 2025), more extreme "ultra-narrow" variants have emerged. For instance, ABE9 utilizes structure-guided engineering to constrain A→G conversion to a 1–2 nt window, drastically minimizing undesired byproducts (Chen *et al*, 2023). Complementary strategies further enhance precision by leveraging motif selectivity (Shang *et al*, 2025) and/or the joint optimization of guide architecture (e.g., 3′-extended gRNAs) with deaminase engineering (Perrotta *et al*, 2025). We have added these discussion in the revised manuscript (lines 523–539).

• In Methods, add exact ABR/DPOAE parameter values in a supplementary table for reproducibility (stimulus timing, artifact rejection, and threshold criteria already described).

Response: Thank you for the reviewer's suggestion. We have detailed the parameter settings for ABR and DPOAE in revised Table EV6 to improve the reproducibility of the study.

Required citations to add:
• Kubisch et al., Cell, 1999 - cloning of KCNQ4 and mapping to DFNA2 (original description).
• Coucke et al., Human Molecular Genetics, 1999 - pathogenic KCNQ4 mutations in DFNA2 families.
• Al-Moyed et al., EMBO Molecular Medicine, 2018 - first proof-of-concept dual-AAV rescue of otoferlin in inner hair cells of Otof knockout mice.
• Vona et al., MedComm, 2025 - recent overview of preclinical and clinical inner-ear gene

therapy studies.

Response: These references have already been cited in the appropriate places.

Referee #3 (Comments on Novelty/Model System for Author):

Preclinical mouse models for human deafness are essential before considering moving to non-human primates and/or human clinical trials.

Referee #3 (Remarks for Author):

The work demonstrated base editing on a mouse model for human KCNQ4. A dual-AAV delivery approach with the ABE8e adenine base editor led to a correction efficiency of 22.2-29.8%. Given the promise of CRISPR base editing for gene therapy for multiple diseases, it is critical that this work be done on relevant mouse models for human deafness. A major challenge is lasting auditory restoration, to achieve a long duration for therapy in humans. The authors chose to attempt such therapy for an autosomal dominant form of deafness, KCNQ4, responsible for approximately 9.5% of this form of hearing loss. The results led to improved auditory threshold for at least 32 weeks, with a delay in degeneration of hair cells, spiral ganglion neurons, and afferent and efferent nerve fibers.

After screening their deafness cohort, a number of KCNQ4 variants were identified. A homozygous mouse model for one of these, c.964G>A (p.G322S), was created using CRISPR/Cas9-mediated knock-in technology, demonstrating hearing loss. A thorough study was done to evaluate the restoration of hearing in the mutants following high or low-dose AAV_ABE8e_N+C_322 or an empty AAV, including immunofluorescence for cochlear morphology, whole-cell voltage–clamp recordings, and a safety evaluation of inflammation and macrophage recruitment. Most compelling, with only an ~25% correction, the authors showed a dramatic ABR recovery (~50 dB), which was very efficient. The histology, immunofluorescence and electrophysiology all match well with this recovery.

Timing is critical and while Kcnq4 expression in OHCs begins at P8, there was a successful window of intervention seen from P9-14.

The in vitro screening of the ABE systems and sgRNAs was straightforward. For the in vivo correction, the viral dose was reasonable and matches an appropriate range (up to 5x10^10 vg/gc per mouse at p0).
Response： We thank the reviewer’ comments.

In the GFP and controls, the authors showed that dual AAV injection does not impair hearing in wild-type mice, and that AAV-ie-CMV-EGFP injection resulted in GFP

expression in the tissue. Have control groups been done, either the GFP vector alone or PBS/saline, to show no effect to controls and not only in untreated mice? If done, it would be relevant to show in supplementary data. This is, however, not essential moving forward.

Response: We thank the reviewer's suggestion. We have added an additional control group of PBS-injected WT mice, as shown in the revised figure EV3.

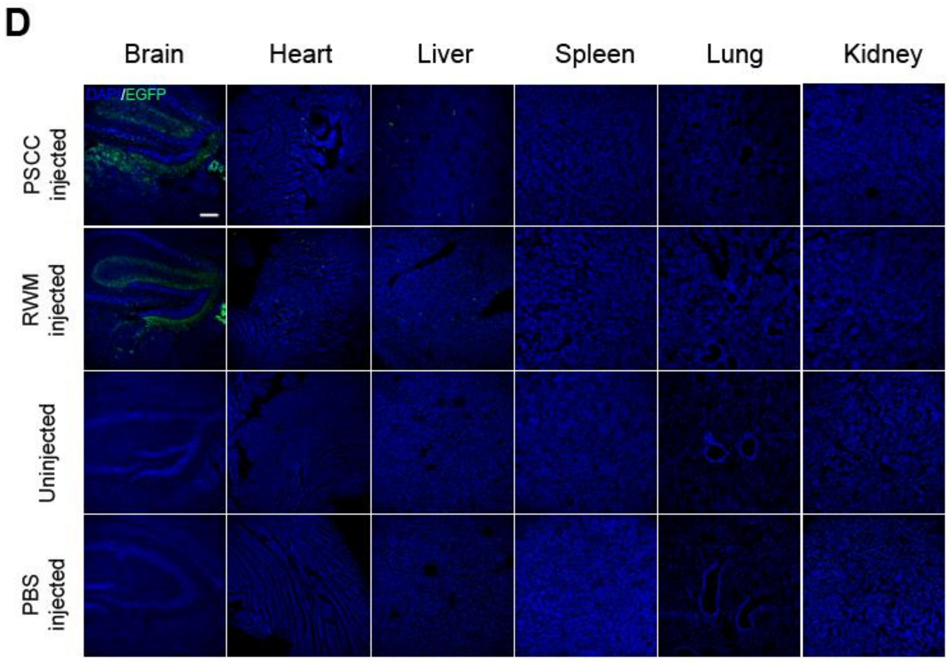

Figure EV3. (D) Representative immunofluorescence images of major organs from *Kcnq4*$^{+/+}$ mice three weeks after AAV-ie-CMV-EGFP administration via PSCC or RWM injection (1 × 10^{10} gc/mouse) , PBS -injected and uninjected controls.

In the off-target analysis, the authors saw a dose-dependent off-target RNA, though not DNA, editing. This finding has clinical implications and needs to be evaluated if this technique moves to the clinic with regards to safety. It is exactly for this reason that this preclinical model is essential.

Response: We thank the reviewer' comments. We have incorporated analyses of the positional distribution and predicted functional consequences of RNA A-to-I variants in our study. Additionally, we have included an unbiased, high-depth whole-genome sequencing (WGS) assessment to evaluate potential DNA-level off-target effects.

The high-dose group exhibited accelerated auditory threshold deterioration. Do the authors predict that the decline is due to immunologic (AAV tropism, macrophage infiltration), off-targets, or bystander edits?

Response: Response: We appreciate the reviewer's insightful query regarding the potential mechanisms underlying the accelerated late-stage deterioration observed in the high-dose treatment group. We hypothesize that this phenomenon is driven by the interplay of three primary factors:

1. **Editing Toxicity: RNA Off-Target Effects**

Beyond catalyzing A-to-G conversions on genomic DNA, the TadA deaminase module within ABEs can engage RNA, inducing widespread, transcriptome-wide off-target RNA A-to-I editing (Richter *et al.*, 2020; Zhou *et al.*, 2019). Evidence suggests that at high exposure levels, frequent RNA off-target events can compromise cellular viability and pose in vivo risks (Zhang *et al.*, 2026). Our transcriptomic analysis aligns with this, revealing a higher frequency of RNA A-to-I edits in the high-dose cohort (revised Figure EV4I; appendix Figure S5E-G). Over time, this cumulative RNA off-target activity likely imposes chronic stress on hair cell survival. Consequently, engineering ABE variants with reduced RNA off-target propensity—while maintaining a balanced dose-response—remains a critical strategy for mitigating this limitation (Li *et al.*, 2021; Zhang *et al.*, 2026).

2. **Multi-Component Immunogenicity: Capsid, Genome, and Editor**

AAV-mediated delivery introduces several immunogenic cues:

Innate Sensing of Capsids: AAV capsids are recognized by innate immune receptors (e.g., TLR2), triggering downstream inflammatory signaling and cytokine production. Given the presence of resident macrophages in the cochlea, such activation can foster a pro-inflammatory microenvironment (Chan *et al.*, 2022; Hosel *et al.*, 2012; Ishibashi *et al.*, 2023).

Genome-Mediated Response: Unmethylated CpG motifs in the AAV genome can be sensed by endosomal TLR9 (signaling via MyD88) to induce Type I interferons and inflammatory cytokines. Furthermore, cytosolic DNA-sensing pathways (e.g., cGAS–STING–TBK1, with parallel NF-κB activation) may amplify inflammation in response to ITR-containing genomes or dsDNA forms (Chandler *et al.*, 2019; Muhuri *et al.*, 2021).

Adaptive Immune Risks: Since AAV-delivered editors are expressed long-term, degradation-derived peptides can be presented on MHC-I and recognized by CD8+ T cells (Najera *et al.*, 2025). While macrophages predominate in the cochlea, resident populations of T, B, and NK cells exist (Liu & Xu, 2023), and injury may further recruit adaptive immune cells to the organ of Corti (Rai *et al.*, 2020). Sustained exposure to this "inflammatory cytokine milieu" likely impairs hair cell metabolism and viability over time (Su *et al.*, 2025; Sun *et al.*, 2025).

Notably, recent evidence showing that immunomodulation (e.g., dexamethasone) can reduce macrophage recruitment underscores the pivotal role of immune management in inner-ear therapy (Wang *et al.*, 2025a).

4. Perturbation of Cellular Proteostasis

High capsid loads may saturate the ubiquitin-proteasome system (UPS), creating a substantial proteostatic burden that potentially interferes with endogenous protein

turnover (Kandel *et al.*, 2024; Li *et al.*, 2013). Simultaneously, high-level and sustained expression of exogenous proteins can elicit cellular stress responses; in some models, stress markers consistent with proteostasis disruption (including UPR/ER-stress–like signatures in certain contexts) have been observed alongside tissue injury. If these stressors chronically exceed a physiological threshold, adaptive responses may shift toward pro-apoptotic programs, compromising the long-term durability of the therapeutic effect (Kim *et al.*, 2006; Lange *et al.*, 2016; Yasuno *et al.*, 2024).

In summary, we speculate that high-dose administration provides substantial early benefit because the editing-driven repair initially outweighs these adverse influences. However, as cumulative toxicity and inflammation reach a "tipping point," hair cell loss accelerates. We have added these results in the revised manuscript (lines 436–452).

Injections were on neonates and in the future, a juvenile or adult cohort of mice would add to the clinical relevance.

Response： We thank the reviewer' comments and fully agree with the reviewer's perspective. Currently, delivery platforms capable of efficiently transducing OHCs in adult mice remain extremely limited. In addition, the adult inner ear exhibits increased susceptibility to vector-associated toxicity, underscoring the need to develop delivery systems that combine high efficiency with minimal adverse effects. We are currently screening for AAVs capable of robustly infecting OHCs in adult mice. Once such AAVs are identified, we will prioritize implementing this therapeutic strategy in adult cohorts to further enhance its translational potential and facilitate eventual clinical application.

Finally, this is the most successful report of Kcnq4 base editing and correction of hearing in a mouse model for this human form of autosomal dominant.

response： We appreciate the reviewer's positive assessment of our work.

Essential to change:
In Figure 3A, use the word SACRIFICE, and not KILL

Response: Thanks for the reviewer's reminder. This has been addressed in the revised Figure 3A.

Sincerely,
On behalf of the authors,
Yingjie Zhang, Ph.D.
Institute of Rare Diseases
West China Hospital, Sichuan University
37 Guoxuexiang, Chengdu 610041, Sichuan, China
Email: yingjiezhang@scu.edu.cn

Reference

Al-Moyed H, Cepeda AP, Jung S, Moser T, Kugler S, Reisinger E (2019) A dual-AAV approach restores fast exocytosis and partially rescues auditory function in deaf otoferlin knock-out mice. *EMBO Mol Med* 11

Bamidele N, Zhang H, Dong X, Cheng H, Gaston N, Feinzig H, Cao H, Kelly K, Watts JK, Xie J *et al* (2024) Domain-inlaid Nme2Cas9 adenine base editors with improved activity and targeting scope. *Nat Commun* 15: 1458

Brovin A, Minskaia E, Sabantsev M, Chuvpilo S, Karabelsky A (2024) Protein trans-splicing: optimization of intein-mediated GFP assembly as a model for the development of gene therapy. *Front Bioeng Biotechnol* 12: 1488912

Chan J, Telang R, Kociszewska D, Thorne PR, Vlajkovic SM (2022) A High-Fat Diet Induces Low-Grade Cochlear Inflammation in CD-1 Mice. *Int J Mol Sci* 23

Chandler LC, Barnard AR, Caddy SL, Patricio MI, McClements ME, Fu H, Rada C, MacLaren RE, Xue K (2019) Enhancement of Adeno-Associated Virus-Mediated Gene Therapy Using Hydroxychloroquine in Murine and Human Tissues. *Mol Ther Methods Clin Dev* 14: 77-89

Chen L, Zhang S, Xue N, Hong M, Zhang X, Zhang D, Yang J, Bai S, Huang Y, Meng H *et al* (2023) Engineering a precise adenine base editor with minimal bystander editing. *Nature chemical biology* 19: 101-110

Davis JR, Wang X, Witte IP, Huang TP, Levy JM, Raguram A, Banskota S, Seidah NG, Musunuru K, Liu DR (2022) Efficient in vivo base editing via single adeno-associated viruses with size-optimized genomes encoding compact adenine base editors. *Nat Biomed Eng* 6: 1272-1283

Ferreira MV, Fernandes S, Almeida AI, Neto S, Mendes JP, Silva RJS, Peixoto C, Coroadinha AS (2023) Extending AAV Packaging Cargo through Dual Co-Transduction: Efficient Protein Trans-Splicing at Low Vector Doses. *Int J Mol Sci* 24

Guillou J, de Pellegars A, Porcheret F, Fremeaux-Bacchi V, Allain-Launay E, Debord C, Denis M, Pereon Y, Barnerias C, Desguerre I *et al* (2022) Fatal thrombotic microangiopathy case following adeno-associated viral SMN gene therapy. *Blood Adv* 6: 4266-4270

Han S, Xu Z, Wang S, Tang H, Hu S, Wang H, Guan G, Shu Y (2024) Distributional comparison of different AAV vectors after unilateral cochlear administration. *Gene Ther* 31: 154-164

Hollidge BS, Carroll HB, Qian R, Fuller ML, Giles AR, Mercer AC, Danos O, Liu Y, Bruder JT, Smith JB (2022) Kinetics and durability of transgene expression after intrastriatal injection of AAV9 vectors. *Front Neurol* 13: 1051559

Hong JW, Min H, Kim S, Song HG, Choi Y, Kim CH, Bok J, Gee HY (2026) Pharmacological chaperones mitigate noise-induced hearing loss by attenuating sustained PERK activation. *Proc Natl Acad Sci U S A* 123: e2421591123

Hosel M, Broxtermann M, Janicki H, Esser K, Arzberger S, Hartmann P, Gillen S, Kleeff J, Stabenow D, Odenthal M *et al* (2012) Toll-like receptor 2-mediated innate immune response in human nonparenchymal liver cells toward adeno-associated viral vectors. *Hepatology* 55: 287-297

Hu SW, Jeong S, Jiang L, Koo H, Wang Z, Choi WH, Zhu B, Seok H, Zhou Y, Kim MG *et al* (2025) PAM-flexible adenine base editing rescues hearing loss in a humanized MPZL2 mouse model harboring an East Asian founder mutation. *Nature communications* 16: 7186

Ishibashi Y, Sung CYW, Grati M, Chien W (2023) Immune responses in the mammalian inner ear and their implications for AAV-mediated inner ear gene therapy. *Hear Res* 432: 108735

Ishibashi Y, Zhu J, Gernoux G, Yu Y, Suh MJ, Isgrig K, Grati M, Olszewski R, Hoa M, Liang C *et al* (2025) AAV-mediated inner ear gene delivery triggers mild host immune responses in the mammalian inner ear. *Molecular therapy Methods & clinical development* 33: 101456

Jang SH, Roh JW, Oh KS, Joo SY, Kim JA, Kim SJ, Choi JY, Jung J, Kim Y, Bok J *et al* (2025) Antisense oligonucleotide therapy mitigates autosomal dominant progressive hearing loss in a murine model of human DFNA2. *Mol Ther* 33: 6479-6498

Kandel R, Jung J, Neal S (2024) Proteotoxic stress and the ubiquitin proteasome system. *Semin Cell Dev Biol* 156: 107-120

Kaur T, Zamani D, Tong L, Rubel EW, Ohlemiller KK, Hirose K, Warchol ME (2015) Fractalkine Signaling Regulates Macrophage Recruitment into the Cochlea and Promotes the Survival of Spiral Ganglion Neurons after Selective Hair Cell Lesion. *J Neurosci* 35: 15050-15061

Kharkovets T, Hardelin JP, Safieddine S, Schweizer M, El-Amraoui A, Petit C, Jentsch TJ (2000) KCNQ4, a K+ channel mutated in a form of dominant deafness, is expressed in the inner ear and the central auditory pathway. *Proceedings of the National Academy of Sciences of the United States of America* 97: 4333-4338

Kim DY, Chung Y, Lee Y, Jeong D, Park KH, Chin HJ, Lee JM, Park S, Ko S, Ko JH *et al* (2022) Hypercompact adenine base editors based on transposase B guided by engineered RNA. *Nat Chem Biol* 18: 1005-1013

Kim R, Emi M, Tanabe K, Murakami S (2006) Role of the unfolded protein response in cell death. *Apoptosis* 11: 5-13

Kumar SRP, Biswas M, Cao D, Arisa S, Munoz-Melero M, Lam AK, Pineros AR, Kapur R, Kaisho T, Kaufman RJ *et al* (2024) TLR9-independent CD8(+) T cell responses in hepatic AAV gene transfer through IL-1R1-MyD88 signaling. *Mol Ther* 32: 325-339

Kweon J, Jang AH, Kwon E, Kim U, Shin HR, See J, Jang G, Lee C, Koo T, Kim S *et al* (2023) Targeted dual base editing with Campylobacter jejuni Cas9 by single AAV-mediated delivery. *Exp Mol Med* 55: 377-384

Lange AM, Altynova ES, Nguyen GN, Sabatino DE (2016) Overexpression of factor VIII after AAV delivery is transiently associated with cellular stress in hemophilia A mice. *Mol Ther Methods Clin Dev* 3: 16064

Li C, He Y, Nicolson S, Hirsch M, Weinberg MS, Zhang P, Kafri T, Samulski RJ (2013) Adeno-associated virus capsid antigen presentation is dependent on endosomal escape. *J Clin Invest* 123: 1390-1401

Li J, Yu W, Huang S, Wu S, Li L, Zhou J, Cao Y, Huang X, Qiao Y (2021) Structure-guided engineering of adenine base editor with minimized RNA off-targeting activity. *Nat Commun* 12: 2287

Liu Y, Li L, Huang P, Zha D, Deng H (2025) Nanotechnology-enhanced gene therapy for hearing loss. *Nanoscale Horiz* 10: 2641-2667

Liu YC, Xu K (2023) Macrophage-related immune responses in inner ear: a potential therapeutic target for sensorineural hearing loss. *Front Neurosci* 17: 1339134

Lv J, Wang H, Cheng X, Chen Y, Wang D, Zhang L, Cao Q, Tang H, Hu S, Gao K *et al* (2024) AAV1-hOTOF gene therapy for autosomal recessive deafness 9: a single-arm trial. *Lancet* 403: 2317-2325

Muhuri M, Maeda Y, Ma H, Ram S, Fitzgerald KA, Tai PW, Gao G (2021) Overcoming innate immune barriers that impede AAV gene therapy vectors. *J Clin Invest* 131

Najera SS, Nicastri A, Bing S, Sajib AM, Ternette N, Mazor R (2025) AAV2 delivery of the saCas9 gene results in presentation of an HLA-A(*)02:01-restricted T cell epitope potent to induce T cell cytotoxicity. *Mol Ther Methods Clin Dev* 33: 101506

Noh B, Gopalappa R, Lin H, Gee HY, Choi JY, Kim HH, Jung J (2025) Engineered virus-like particles for in vivo gene editing ameliorate hearing loss in murine DFNA2 model. *Mol Ther* 33: 6449-6462

Noh B, Rim JH, Gopalappa R, Lin H, Kim KM, Kang MJ, Gee HY, Choi JY, Kim HH, Jung J (2022) In vivo outer hair cell gene editing ameliorates progressive hearing loss in dominant-negative Kcnq4 murine model. *Theranostics* 12: 2465-2482

Nonnenmacher M, Weber T (2012) Intracellular transport of recombinant adeno-associated virus vectors. *Gene Ther* 19: 649-658

Pan J, Wang K, Qu J, Chen D, Chen A, You Y, Tang J, Zhang H (2024) Activated tissue-resident macrophages contribute to hair cell insults in noise-induced hearing loss in mice. *Commun Biol* 7: 1078

Pardi N, Tuyishime S, Muramatsu H, Kariko K, Mui BL, Tam YK, Madden TD, Hope MJ, Weissman D (2015) Expression kinetics of nucleoside-modified mRNA delivered in lipid nanoparticles to mice by various routes. *J Control Release* 217: 345-351

Perrotta RM, Vinke S, Ferreira R, Moret M, Mahas A, Chiappino-Pepe A, Riedmayr LM, Mehra AT, Lehmann LS, Church GM (2025) Engineered base editors with reduced bystander editing through directed evolution. *Nat Biotechnol*

Picardi E, D'Erchia AM, Lo Giudice C, Pesole G (2017) REDIportal: a comprehensive database of A-to-I RNA editing events in humans. *Nucleic acids research* 45: D750-D757

Qi J, Tan F, Zhang L, Lu L, Zhang S, Zhai Y, Lu Y, Qian X, Dong W, Zhou Y *et al* (2024) AAV-Mediated Gene Therapy Restores Hearing in Patients with DFNB9 Deafness. *Advanced science* 11: e2306788

Qi J, Zhang L, Lu L, Tan F, Cheng C, Lu Y, Dong W, Zhou Y, Fu X, Jiang L *et al* (2025) AAV gene therapy for autosomal recessive deafness 9: a single-arm trial. *Nature medicine* 31: 2917-2926

Rai V, Wood MB, Feng H, Schabla NM, Tu S, Zuo J (2020) The immune response after noise damage in the cochlea is characterized by a heterogeneous mix of adaptive and innate immune cells. *Sci Rep* 10: 15167

Reimsnider S, Manfredsson FP, Muzyczka N, Mandel RJ (2007) Time course of transgene expression after intrastriatal pseudotyped rAAV2/1, rAAV2/2, rAAV2/5, and rAAV2/8 transduction in the rat. *Mol Ther* 15: 1504-1511

Richter MF, Zhao KT, Eton E, Lapinaite A, Newby GA, Thuronyi BW, Wilson C, Koblan LW, Zeng J, Bauer DE *et al* (2020) Phage-assisted evolution of an adenine base editor with improved Cas domain compatibility and activity. *Nat Biotechnol* 38: 883-891

Shang M, Li Y, Cao Q, Ren J, Zeng Y, Wang J, Gonzalez RVL, Zhang X (2025) A motif preferred adenine base editor with minimal bystander and off-targets editing. *Nat Commun* 16: 9153

Su Z, Chen Y, Liu Y, Cao J, Cui J, Chen H, Li Q (2025) Oxidative stress and inflammation combine to exacerbate cochlear damage and sensorineural hearing loss in C57BL/6 mice. *Front Neurosci* 19: 1563428

Sun Y, Zou S, Xu X, Xu S, Sun H, Tang M, Kong W, Chen X, He Z (2025) Inhibition of the cGAS-STING Pathway Reduces Cisplatin-Induced Inner Ear Hair Cell Damage. *Neurosci Bull* 41: 359-373

Tang H, Wang H, Wang S, Hu SW, Lv J, Xun M, Gao K, Wang F, Chen Y, Wang D *et al* (2023) Hearing of Otof-deficient mice restored by trans-splicing of N- and C-terminal otoferlin. *Hum Genet* 142: 289-304

Taylor RR, Jagger DJ, Forge A (2012) Defining the cellular environment in the organ of Corti following extensive hair cell loss: a basis for future sensory cell replacement in the Cochlea. *PLoS One* 7: e30577

Tornabene P, Trapani I, Minopoli R, Centrulo M, Lupo M, de Simone S, Tiberi P, Dell'Aquila F, Marrocco E, Iodice C *et al* (2019) Intein-mediated protein trans-splicing expands adeno-associated virus transfer

capacity in the retina. *Sci Transl Med* 11

Valayannopoulos V, Bance M, Carvalho DS, Greinwald JH, Jr., Harvey SA, Ishiyama A, Landry EC, Lowenheim H, Lustig LR, Manrique M *et al* (2025) DB-OTO Gene Therapy for Inherited Deafness. *The New England journal of medicine*

Valdez I, O'Connor I, Patel D, Gierer K, Harrington J, Ellis E, Caponetti SA, Sebra RP, Valley HC, Coote K *et al* (2025) A streamlined base editor engineering strategy to reduce bystander editing. *Nat Commun* 16: 8115

Wang H, Chen Y, Lv J, Cheng X, Cao Q, Wang D, Zhang L, Zhu B, Shen M, Xu C *et al* (2024) Bilateral gene therapy in children with autosomal recessive deafness 9: single-arm trial results. *Nature medicine* 30: 1898-1904

Wang X, Zhang L, Chen S, Xie L, Qiu Y, Kong C, Yin G, Kong W, Sun Y (2025a) Viral-Mediated Connexin 26 Expression Combined with Dexamethasone Rescues Hearing in a Conditional Gjb2 Null Mice Model. *Adv Sci (Weinh)* 12: e2406510

Wang Y, Kong Y, Zhang Y, He Y, Wang W, Lu Y, Meng J, Yuan H (2025b) Long-Term Attenuation of Vascular Hyperpermeability in a Hereditary Angioedema Mouse Model by Adenine Base Editing. *Allergy*

Xu X, Xu K, Chen F, Yu D, Wang X (2024) Mfsd2a regulates the blood-labyrinth-barrier formation and function through tight junctions and transcytosis. *Hear Res* 450: 109048

Xue Y, Tao Y, Wang X, Wang X, Shu Y, Liu Y, Kang W, Chen S, Cheng Z, Yan B *et al* (2023) RNA base editing therapy cures hearing loss induced by OTOF gene mutation. *Mol Ther* 31: 3520-3530

Xun M, Fan X, Wang H, Zhao J, Tang G, Zhang W, Hu S, Zhang L, Wang D, Chen Y *et al* (2026) Comparative analysis of RNA versus protein splicing in dual AAV-mediated gene therapy in a mouse model of DFNB9 deafness. *Mol Ther* 34: 203-215

Yasuno K, Watanabe R, Ishida R, Okado K, Kondo H, Iguchi T, Imaoka M, Tsuchiya Y (2024) Protein overexpression by adeno-associated virus-based gene therapy products in cardiomyocytes induces endoplasmic reticulum stress and myocardial degeneration in mice. *J Toxicol Pathol* 37: 139-149

Zettler J, Schutz V, Mootz HD (2009) The naturally split Npu DnaE intein exhibits an extraordinarily high rate in the protein trans-splicing reaction. *FEBS Lett* 583: 909-914

Zhang C, Chen Z, Cao J, Zhang Z, Li WK, Zhang X, Chen J, Liu J, Yuan Z, Gao F *et al* (2026) RNA inosine sensor-guided TadA mutational scanning for toxicity minimization of adenine base editors. *Mol Ther* 34: 380-396

Zhou C, Sun Y, Yan R, Liu Y, Zuo E, Gu C, Han L, Wei Y, Hu X, Zeng R *et al* (2019) Off-target RNA mutation induced by DNA base editing and its elimination by mutagenesis. *Nature* 571: 275-278

30th Mar 2026

Dear Dr. Zhang,

Thank you for the submission of your revised manuscript to EMBO Molecular Medicine. I am pleased to inform you that we will be able to accept your manuscript pending the following final amendments:

1) Figures: Please check Fig. EV3A Uninjected/EGFP images as they appear to be empty.
2) Author Checklist: Please make sure that all relevant fields are completed.
3) In the main manuscript file, please do the following:
- Please address all comments suggested by our data editors listed below:
o Figure legends:
1. Please note that the exact p values are not provided in the legends of figures 1B-I, M; 2D-F; 3B, C; 4A-H, J, K, L; 5B, C, E; 6C, D, E; 7A-C; EV4 A, B, I; EV5 M.
2. Please note that information related to n is missing in the legends of figures 1L, M .
3. Please note that the error bars are not defined in the legends of figures EV1 D, EV2 B.
- Please correct the order and the headings of the sections in the manuscript text to: Abstract / The Paper Explained / Introduction / Results and Discussion / Methods / Data Availability / Acknowledgements / Disclosure and Competing Interests Statement / References / Figure Legends / Tables / Expanded View Figure Legends
- Add callouts for Fig. 4I and Fig. 7C. Also make sure that figures and tables are called out in a sequential order. Currently, Table EV4 is called out before Table EV2 and Fig. EV4 before Fig. EV3D,E. Please correct.
- Remove information about BioRender from the Acknowledgement and add a dedicated "Graphics" section in the Methods, following this format:
Graphics:
(some of the... OR Figure #... OR synopsis) Graphics were created with BioRender.com.
- Remove Reagents Table from manuscript text and upload it as separate .doc file.
- In Methods, statistical paragraph should reflect all information that you have filled in the Authors Checklist, especially regarding randomization, blinding, replication.
- Indicate in legends exact n and exact p values, not a range, along with the statistical test used. To keep the figures "clear" some authors found providing an Appendix table Sx with all exact p-values preferable. You are welcome to do this if you want to.
- Author contributions: Please remove it from the manuscript and specify author contributions in our submission system. CRediT has replaced the traditional author contributions section because it offers a systematic machine-readable author contributions format that allows for more effective research assessment. You are encouraged to use the free text boxes beneath each contributing author's name to add specific details on the author's contribution. More information is available in our guide to authors:
https://www.embopress.org/page/journal/17574684/authorguide#authorshipguidelines
- In data availability statement leave only information about data deposited in public repositories. Please use the following format to report the accession number of your data:

The datasets produced in this study are available in the following databases:
[data type]: [full name of the resource] [accession number/identifier] ([doi or URL or identifiers.org/DATABASE:ACCESSION])

Please check "Author Guidelines" for more information.
https://www.embopress.org/page/journal/17574684/authorguide#availabilityofpublishedmaterial
4) Appendix: Please add table of contents with page numbers on the title page, correct nomenclature to "Appendix Figure S1" etc. throughout, remove yellow highlights and upload final version in PDF format.
5) The Paper Explained: Please add it to the main manuscript file.
6) Synopsis:
- Please check your synopsis text and image before submission with your revised manuscript. Please be aware that in the proof stage minor corrections only are allowed (e.g., typos).
7) As part of the EMBO Publications transparent editorial process (see our Editorial at http://embomolmed.embopress.org/content/2/9/329), EMBO Molecular Medicine will publish online a Review Process File (RPF) to accompany accepted manuscripts. This file will be published in conjunction with your paper and will include the anonymous referee reports, your point-by-point response and all pertinent correspondence relating to the manuscript. Let us know if you want to remove or not any figures from it prior to publication. Please note that the Authors checklist will be published at the end of the RPF.
8) Please provide a point-by-point letter INCLUDING my comments as well as the reviewer's reports and your detailed responses (as Word file).

I look forward to reading a new revised version of your manuscript as soon as possible.

Yours sincerely,

Zeljko Durdevic

Zeljko Durdevic
Senior Editor
EMBO Molecular Medicine

*** Instructions to submit your revised manuscript ***

*** PLEASE NOTE *** As part of the EMBO Publications transparent editorial process initiative (see our Editorial at https://www.embopress.org/doi/pdf/10.1002/emmm.201000094), EMBO Molecular Medicine will publish online a Review Process File to accompany accepted manuscripts.

In the event of acceptance, this file will be published in conjunction with your paper and will include the anonymous referee reports, your point-by-point response and all pertinent correspondence relating to the manuscript. If you do NOT want this file to be published, please inform the editorial office at contact@embomolmed.org.

To submit your manuscript, please follow this link:

https://embomolmed.msubmit.net/cgi-bin/main.plex

*****

When preparing your revised manuscript, please refer to our guidelines: https://link.springer.com/journal/44321/submission-guidelines#cms-Revised-submissions. We perform an initial quality control of all revised manuscripts before re-review; failure to include requested items will delay the evaluation of your revision.

We require:

1) A .docx formatted version of the manuscript text (including legends for main figures, EV figures and tables). Please make sure that the changes are highlighted to be clearly visible.

2) Individual production quality figure files as .eps, .tif, .jpg (one file per figure). For guidance, download the 'Figure Guide PDF': https://media.springernature.com/original/springer-cms/rest/v1/content/27825798/data/v1.

3) A .docx formatted letter INCLUDING the reviewers' reports and your detailed point-by-point responses to their comments. As part of the EMBO Press transparent editorial process, the point-by-point response is part of the Review Process File (RPF), which will be published alongside your paper.

4) A complete author checklist, which you can download from our author guidelines. Please insert information in the checklist that is also reflected in the manuscript. The completed author checklist will also be part of the RPF.

5) Please note that all corresponding authors are required to supply an ORCID ID for their name upon submission of a revised manuscript.

6) It is mandatory to include a 'Data Availability' section after the Materials and Methods. Before submitting your revision, primary datasets produced in this study need to be deposited in an appropriate public database, and the accession numbers and database listed under 'Data Availability'. Please remember to provide a reviewer password if the datasets are not yet public.

In case you have no data that requires deposition in a public database, please state so in this section. Note that the Data Availability Section is restricted to new primary data that are part of this study.

7) For data quantification: please specify the name of the statistical test used to generate error bars and P values, the number (n) of independent experiments (specify technical or biological replicates) underlying each data point and the test used to calculate p-values in each figure legend. The figure legends should contain a basic description of n, P and the test applied. Graphs must include a description of the bars and the error bars (s.d., s.e.m.).

8) At EMBO Press we ask authors to provide source data for the main manuscript figures. You will receive a separate email with

instructions for providing source data with your revised manuscript, including how to upload and organize the files.

9) Our journal encourages inclusion of *data citations in the reference list* to directly cite datasets that were re-used and obtained from public databases. Data citations in the article text are distinct from normal bibliographical citations and should directly link to the database records from which the data can be accessed. In the main text, data citations are formatted as follows: "Data ref: Smith et al, 2001" or "Data ref: NCBI Sequence Read Archive PRJNA342805, 2017". In the Reference list, data citations must be labeled with "[DATASET]". A data reference must provide the database name, accession number/identifiers and a resolvable link to the landing page from which the data can be accessed at the end of the reference.

10) We replaced Supplementary Information with Expanded View (EV) Figures and Tables that are collapsible/expandable online. A maximum of 5 EV Figures can be typeset. EV Figures should be cited as 'Figure EV1, Figure EV2" etc... in the text and their respective legends should be included in the main text after the legends of regular figures.

- For the figures that you do NOT wish to display as Expanded View figures, they should be bundled together with their legends in a single PDF file called *Appendix*, which should start with a short Table of Content. Appendix figures should be referred to in the main text as: "Appendix Figure S1, Appendix Figure S2" etc.

- Additional Tables/Datasets should be labeled and referred to as Table EV1, Dataset EV1, etc. Legends have to be provided in a separate tab in case of .xls files. Alternatively, the legend can be supplied as a separate text file (README) and zipped together with the Table/Dataset file.

11) The paper explained: EMBO Molecular Medicine articles are accompanied by a summary of the articles to emphasize the major findings in the paper and their medical implications for the non-specialist reader. Please provide a draft summary of your article highlighting

- the medical issue you are addressing,

- the results obtained and

- their clinical impact.

This may be edited to ensure that readers understand the significance and context of the research. Please refer to any of our published articles for an example.

12) Author contributions: You will be asked to provide CRediT (Contributor Role Taxonomy) terms in the submission system. These replace a narrative author contribution section in the manuscript.

13) A Conflict of Interest statement should be provided in the main text.

14) Every published paper includes a 'Synopsis' to further enhance discoverability. Synopses are displayed on the journal webpage and are freely accessible to all readers. They include a short stand first (maximum of 300 characters, including space) as well as 2-5 one-sentences bullet points that summarizes the paper. Please write the bullet points to summarize the key NEW findings. They should be designed to be complementary to the abstract - i.e. not repeat the same text. We encourage inclusion of key acronyms and quantitative information (maximum of 30 words / bullet point). Please use the passive voice. Please attach these in a separate file or send them by email, we will incorporate them accordingly.

Please also suggest a striking image or visual abstract to illustrate your article as a PNG file 550 px wide x 300 px high.

15) Include a Reagents and Tools Table as part of the Methods section, which can be downloaded from our author guidelines.

EMBO Molecular Medicine has a "scooping protection" policy, whereby similar findings that are published by others during review or revision are not a criterion for rejection. Should you decide to submit a revised version, I do ask that you get in touch after three months if you have not completed it, to update us on the status.

*Additional important information regarding Figures

Each figure should be given in a separate file and should have the following resolution:
Graphs 800-1,200 DPI
Photos 400-800 DPI
Colour (only CMYK) 300-400 DPI"

Figures are not edited by the production team. All lettering should be the same size and style; figure panels should be indicated by capital letters (A, B, C etc). Gridlines are not allowed except for log plots. Figures should be numbered in the order of their

appearance in the text with Arabic numerals. Each Figure must have a separate legend and a caption is needed for each panel.

*Additional important information regarding figures and illustrations can be found at https://media.springernature.com/original/springer-cms/rest/v1/content/27825798/data/v1

***** Reviewer's comments *****

Referee #1 (Comments on Novelty/Model System for Author):

I think this is a nice study which utilized base editor successfully to partially improve the auditory function in a new mouse model of DFNA2. The revised manuscript has sufficiently addressed my comments.

Referee #1 (Remarks for Author):

The authors have satisfactorily addressed my comments. Nice study!

Referee #2 (Remarks for Author):

I very much appreciate the scope and depth of the revisions. This was a strong MS already before and is further strengthened by the revisions, congrats.

The authors addressed the remaining editorial issues.

13th Apr 2026

Dear Dr. Zhang,

We are pleased to inform you that your manuscript is accepted for publication and is now being sent to our publisher to be included in the next available issue of EMBO Molecular Medicine.

Your manuscript will be processed for publication by EMBO Press. It will be copy edited and you will receive page proofs prior to publication. Please note that you will be contacted by Springer Nature Author Services to complete licensing and payment information.

You may qualify for financial assistance for your publication charges - either via a Springer Nature fully open access agreement or an EMBO initiative. Check your eligibility: https://link.springer.com/journal/44321/how-to-publish-with-us

Should you be planning a Press Release on your article, please get in contact with embo_production@springernature.com as early as possible in order to coordinate publication and release dates.

If you have any questions, please do not hesitate to contact the Editorial Office. Thank you for your contribution to EMBO Molecular Medicine.

Yours sincerely,
Zeljko Durdevic

Zeljko Durdevic
Senior Editor
EMBO Molecular Medicine

>>> Please note that it is EMBO Molecular Medicine policy for the transcript of the editorial process (containing referee reports and your response letter) to be published as an online supplement to each paper. If you do NOT want this, you will need to inform the Editorial Office via email immediately. More information is available here: https://link.springer.com/partners/embo-press/editorial-policies#Peer%20review

EMBO Press Author Checklist

Corresponding Author Name: yingjie zhang	
Journal Submitted to: EMBO molecular medicine	
Manuscript Number: EMM-2025-22608	

USEFUL LINKS FOR COMPLETING THIS FORM
The EMBO Journal - Author Guidelines
EMBO Reports - Author Guidelines
Molecular Systems Biology - Author Guidelines
EMBO Molecular Medicine - Author Guidelines

Reporting Checklist for Life Science Articles (updated January
This checklist is adapted from Materials Design Analysis Reporting (MDAR) Checklist for Authors. MDAR establishes a minimum set of requirements in transparent reporting in the life sciences (see Statement of Task: 10.31222/osf.io/9sm4x). Please follow the journal's guidelines in preparing your
Please note that a copy of this checklist will be published alongside your article.

Abridged guidelines for figures
1. Data
The data shown in figures should satisfy the following conditions:
- the data were obtained and processed according to the field's best practice and are presented to reflect the results of the experiments in an accurate and unbiased manner.
- ideally, figure panels should include only measurements that are directly comparable to each other and obtained with the same assay.
- plots include clearly labeled error bars for independent experiments and sample sizes. Unless justified, error bars should not be shown for technical
- if n<5, the individual data points from each experiment should be plotted. Any statistical test employed should be justified.
- Source Data should be included to report the data underlying figures according to the guidelines set out in the authorship guidelines on Data

2. Captions
Each figure caption should contain the following information, for each panel where they are relevant:
- a specification of the experimental system investigated (eg cell line, species name).
- the assay(s) and method(s) used to carry out the reported observations and measurements.
- an explicit mention of the biological and chemical entity(ies) that are being measured.
- an explicit mention of the biological and chemical entity(ies) that are altered/varied/perturbed in a controlled manner.
- the exact sample size (n) for each experimental group/condition, given as a number, not a range;
- a description of the sample collection allowing the reader to understand whether the samples represent technical or biological replicates (including how many animals, litters, cultures, etc.).
- a statement of how many times the experiment shown was independently replicated in the laboratory.
- definitions of statistical methods and measures:
  - common tests, such as t-test (please specify whether paired vs. unpaired), simple χ2 tests, Wilcoxon and Mann-Whitney tests, can be unambiguously identified by name only, but more complex techniques should be described in the methods section;
  - are tests one-sided or two-sided?
  - are there adjustments for multiple comparisons?
  - exact statistical test results, e.g., P values = x but not P values < x;
  - definition of 'center values' as median or average;
  - definition of error bars as s.d. or s.e.m.

Please complete ALL of the questions below.
Select "Not Applicable" only when the requested information is not relevant for your study.

Materials

Newly Created Materials	Information included in the manuscript?	In which section is the information available? (Reagents and Tools Table, Materials and Methods, Figures, Data Availability Section)
New materials and reagents need to be available; do any restrictions apply?	Not Applicable	

Antibodies	Information included in the manuscript?	In which section is the information available? (Reagents and Tools Table, Materials and Methods, Figures, Data Availability Section)
For **antibodies** provide the following information:<br>- Commercial antibodies: RRID (if possible) or supplier name, catalogue number and or/clone number<br>- Non-commercial: RRID or citation	Yes	Reagents and tools Table, Materials and methods

DNA and RNA sequences	Information included in the manuscript?	In which section is the information available? (Reagents and Tools Table, Materials and Methods, Figures, Data Availability Section)
**Short novel DNA or RNA including primers, probes**: provide the sequences.	Yes	Reagents and tools

Cell materials	Information included in the manuscript?	In which section is the information available? (Reagents and Tools Table, Materials and Methods, Figures, Data Availability Section)
**Cell lines:** Provide species information, strain. Provide accession number in repository **OR** supplier name, catalog number, clone number, and/**OR** RRID.	Yes	Reagents and tools Table
**Primary cultures:** Provide species, strain, sex of origin, genetic modification status.	Not Applicable	
Report if the cell lines were recently **authenticated** (e.g., by STR profiling) and tested for mycoplasma contamination.	Not Applicable	

Experimental animals	Information included in the manuscript?	In which section is the information available? (Reagents and Tools Table, Materials and Methods, Figures, Data Availability Section)
**Laboratory animals or Model organisms:** Provide species, strain, sex, age, genetic modification status. Provide accession number in repository **OR** supplier name, catalog number, clone number, **OR** RRID.	Yes	Reagents and tools Table
**Animal observed in or captured from the field:** Provide species, sex, and age where possible.	Not Applicable	
Please detail **housing and husbandry conditions**.	Yes	Materials and methods

Plants and microbes	Information included in the manuscript?	In which section is the information available? (Reagents and Tools Table, Materials and Methods, Figures, Data Availability Section)
**Plants:** provide species and strain, ecotype and cultivar where relevant, unique accession number if available, and source (including location for collected wild specimens).	Not Applicable	
**Microbes:** provide species and strain, unique accession number if available, and source.	Not Applicable	

Human research participants	Information included in the manuscript?	In which section is the information available? (Reagents and Tools Table, Materials and Methods, Figures, Data Availability Section)
If collected and within the bounds of privacy constraints report on age, sex and gender or ethnicity for all study participants.	Not Applicable	

Core facilities	Information included in the manuscript?	In which section is the information available? (Reagents and Tools Table, Materials and Methods, Figures, Data Availability Section)
If your work benefited from core facilities, was their service mentioned in the acknowledgments section?	Yes	Acknowledgments section

Design

Study protocol	Information included in the manuscript?	In which section is the information available? (Reagents and Tools Table, Materials and Methods, Figures, Data Availability Section)
If study protocol has been **pre-registered, provide DOI in the manuscript**. For clinical trials, provide the trial registration number **OR** cite DOI.	Not Applicable	
Report the **clinical trial registration number** (at ClinicalTrials.gov or equivalent), where applicable.	Not Applicable	

Laboratory protocol	Information included in the manuscript?	In which section is the information available? (Reagents and Tools Table, Materials and Methods, Figures, Data Availability Section)
Provide DOI OR other citation details if **external detailed step-by-step protocols** are available.	Not Applicable	

Experimental study design and statistics	Information included in the manuscript?	In which section is the information available? (Reagents and Tools Table, Materials and Methods, Figures, Data Availability Section)
Include a statement about **sample size** estimate even if no statistical methods were used.	Yes	Figure legends
Were any steps taken to minimize the effects of subjective bias when allocating animals/samples to treatment (e.g. **randomization procedure**)? If yes, have they been described?	Yes	Materials and methods
Include a statement about **blinding** even if no blinding was done.	Yes	Materials and methods
Describe **inclusion/exclusion criteria** if samples or animals were excluded from the analysis. Were the criteria pre-established?<br><br>If sample or data points were omitted from analysis, report if this was due to attrition or intentional exclusion and provide justification.	Not Applicable	
For every figure, are **statistical tests** justified as appropriate? Do the data meet the assumptions of the tests (e.g., normal distribution)? Describe any methods used to assess it. Is there an estimate of variation within each group of data? Is the variance similar between the groups that are being statistically compared?	Yes	Figure legends, Materials and methods

Sample definition and in-laboratory replication	Information included in the manuscript?	In which section is the information available? (Reagents and Tools Table, Materials and Methods, Figures, Data Availability Section)
In the figure legends: state number of times the experiment was **replicated** in laboratory.	Yes	Figures,Figure legends
In the figure legends: define whether data describe **technical or biological replicates**.	Yes	Figure legends,Materials and methods

Ethics

Ethics	Information included in the manuscript?	In which section is the information available? (Reagents and Tools Table, Materials and Methods, Figures, Data Availability Section)
Studies involving **human participants**: State details of **authority granting ethics approval** (IRB or equivalent committee(s), provide reference number for approval.	Not Applicable	
Studies involving **human participants**: Include a statement confirming that **informed consent** was obtained from all subjects and that the experiments conformed to the principles set out in the WMA Declaration of Helsinki and the Department of Health and Human Services Belmont Report.	Not Applicable	
Studies involving **human participants: For publication of patient photos**, include a statement confirming that consent to publish was obtained.	Not Applicable	
Studies involving experimental **animals**: State details of **authority granting ethics approval** (IRB or equivalent committee(s), provide reference number for approval. Include a statement of compliance with ethical regulations.	Yes	Materials and methods
Studies involving **specimen and field samples:** State if relevant **permits** obtained, provide details of authority approving study; if none were required, explain why.	Not Applicable	

Dual Use Research of Concern (DURC)	Information included in the manuscript?	In which section is the information available? (Reagents and Tools Table, Materials and Methods, Figures, Data Availability Section)
Could your study fall under dual use research restrictions? Please check biosecurity documents and list of **select agents and toxins** (CDC): https://www.selectagents.gov/sat/list.htm	Not Applicable	
If you used a select agent, is the security level of the lab appropriate and reported in the manuscript?	Not Applicable	
If a study is subject to dual use research of concern regulations, is the name of the **authority granting approval and reference number** for the regulatory approval provided in the manuscript?	Not Applicable	

Reporting

The MDAR framework recommends adoption of discipline-specific guidelines, established and endorsed through community initiatives. Journals have their own policy about requiring specific guidelines and recommendations to complement MDAR.

Adherence to community standards	Information included in the manuscript?	In which section is the information available? (Reagents and Tools Table, Materials and Methods, Figures, Data Availability Section)
State if relevant guidelines or checklists (e.g., **ICMJE, MIBBI, ARRIVE, PRISMA**) have been followed or provided.	Not Applicable	
For **tumor marker prognostic studies,** we recommend that you follow the **REMARK** reporting guidelines (see link list at top right). See author guidelines, under 'Reporting Guidelines'. Please confirm you have followed these guidelines.	Not Applicable	
For **phase II and III randomized controlled trials**, please refer to the **CONSORT** flow diagram (see link list at top right) and submit the CONSORT checklist (see link list at top right) with your submission. See author guidelines, under 'Reporting Guidelines'. Please confirm you have submitted this list.	Not Applicable	

Data Availability

Data availability	Information included in the manuscript?	In which section is the information available? (Reagents and Tools Table, Materials and Methods, Figures, Data Availability Section)
Have **primary datasets** been deposited according to the journal's guidelines (see 'Data Deposition' section) and the respective accession numbers provided in the Data Availability Section?	Yes	Data Availability Section
Were **human clinical and genomic datasets** deposited in a public access-controlled repository in accordance to ethical obligations to the patients and to the applicable consent agreement?	Not Applicable	
Are **computational models** that are central and integral to a study available without restrictions in a machine-readable form? Were the relevant accession numbers or links provided?	Not Applicable	
If publicly available data were reused, provide the respective **data citations in the reference list**.	Not Applicable	
